# 39th Annual European Brain and Behaviour Society Abstracts

**DOI:** 10.1155/2007/23250

**Published:** 2007-10-10

**Authors:** Edited by: Alessandro Treves, P. Paolo Battaglini, Leonardo Chelazzi, Mathew Diamond, Giorgio Vallortigara

**Affiliations:** ^1^SISSA, Trieste, Italy; ^2^University of Trieste, Italy; ^3^University of Verona, Italy

## Abstract

The EUROPEAN BRAIN AND BEHAVIOUR SOCIETY has held its 39th Annual General Meeting in Trieste, in the campus next to the Miramare castle and its park, co-hosted by SISSA, the International School for Advanced Studies, and ICTP, the Abdus Salam International Centre for Theoretical Physics. Alessandro Treves (SISSA) was the head and inspiration of the Local Organizing committee, supported by P. Battaglini, L. Chelazzi, M. Diamond and G. Vallortigara. All approaches relating brain and behaviour were represented at the meeting, which aimed to further expand the wide spectrum of previous EBBS AGMs, and to bring together integrative, system, cognitive, computational neuroscientists.

See also the societies home page: http://www.ebbs-science.org/.

We study molecular, cellular, and neuronal circuit mechanisms underlying acquisition, consolidation and retrieval of hippocampus-dependent memory in rodents. Our primary approach is to generate cell type and adult-restricted knockout mice and characterize them using multifaceted methods including molecular and cellular biology, in vitro and in vivo electrophysiology, confocal and two photon microscopy and behavioral tasks. The data obtained to date indicate that NMDA receptor-mediated synaptic plasticity in area CA1 plays a pivotal role in special and other hippocampus dependent learning and memory. The same receptors and synaptic plasticity in area CA3 are dispensable for the acquisition of reference memory, but play an important role in “pattern completion”—the ability to recall an entire experience with limited recall cues, as well as in one trial-based rapid learning. NMDA receptor function in dentate gyrus (DG) is also dispensable for reference memory, but is important in “pattern separation”, the ability to form distinct memories of similar events. These studies attest the power of this multifaceted—genetic, physiological and behavioral—approach in understanding mechanisms underlying cognition.

## Figures and Tables

**Figure 1:**
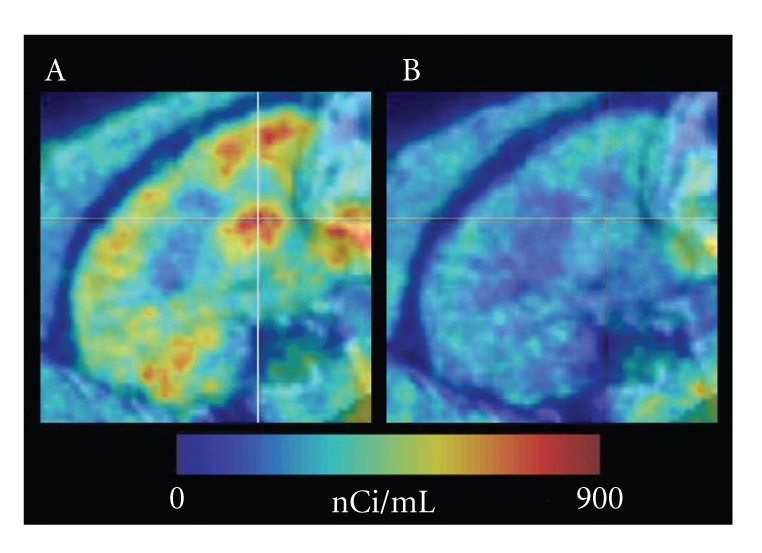
Averaged saggital baboon brain PET images of [11C]JHU75528 scans, displayed on aligned MRI images. Lines cross at the center of left putamen. The images are shown in the same color scale. (A) baseline; (B) blocking with Rimonabant (1 mg/kg, i.v.). Images display the putamen, frontal, parietal, and occipital cortices, and cerebellum, among other structures. In the baseline scan, the radioactivity accumulated predominantly to the putamen, frontal cortex, and cerebellum, and less to thalamus and pons.
