# Supplementary material for: 39th Annual European Brain and Behaviour Society Abstracts
Source: Neural Plast. 2007 Oct 10;2007:23250. doi: 10.1155/2007/23250 (PMC2366049; doi:10.1155/2007/23250)
Supplement: Related texts

# Abstract: SPACE AND TIME OF CORTICAL ACTIVITY

**Authors:** Moshe Abeles

**Affiliations:** Gonda Multidisciplinary Brain Research Center, Bar Ilan University, Ramat Gan, Israel

To understand how the cortex works, we need to define accurate scales in space and time. Anatomical and microscopic studies have parceled the cortex into different scales such as cytoarchitectonic areas, hyper-columns, mini-columns, single neurons, individual synapses, single channels, etc. Because neighbouring neurons can have very different properties, individual neurons appear to be the largest unit that can be used to understand cortical information processing mechanisms.

Activity can be measured by fMRI (or PET) on a time scale of seconds, by EEG (or MEG) on a time scale of a small fraction of a second, and by microelectrodes on a time scale of milliseconds or less. A single neuron can be part of different processes at different times. It remains unclear how fast a neuron can switch its functional properties. If such switching can be made on a ms time scale, fast and efficient computations can be made. We show that the temporal precision of cortical neurons can be better than 1 ms.

# Abstract: THE NEURAL BASIS OF LEARNING AND MEMORY MECHANISMS UNDERLYING DRUG ADDICTION

**Authors:** Barry J. Everitt

**Affiliations:** Department of Experimental Psychology, University of Cambridge, UK

Drug addiction is increasingly viewed as the endpoint of a series of transitions frominitial drug use, when a drug is voluntarily taken because it has reinforcing, often hedonic, effects through loss of control over this behaviour such that it becomes habitual and ultimately compulsive in nature. In this lecture I will discuss the evidence that these transitions depend upon interactions between pavlovian and instrumental learning processes and further, that the transition from an initial stage when drug seeking and taking represents a voluntary, goal-directed action, to a more habitual and compulsive mode represents a transition at the neural level from prefrontal cortical to striatal control over such behaviour. Experiments will be discussed that demonstrate the involvement of limbic corticostriatal systems in drug-seeking behaviour, emphasizing the importance of drug-associated stimuli acting as conditioned reinforcers. In addition, I will consider the possibility of reducing the motivational impact of drugassociated stimuli by disrupting the process of memory reconsolidation that point to a novel therapeutic approach to drug addiction by promoting abstinence and preventing relapse.

# Abstract: FROM SPEECH TO GENE: FOXP2 AND THE KE FAMILY

**Authors:** Faraneh Vargha-Khadem

**Affiliations:** University College London Institute of Child Health and Great Ormond Street Hospital for Children, London, UK

Fifteen of the 30 three-generational KE family members suffer from an orofacial and verbal dyspraxia arising from an underlying impairment in the rapid selection and accurate sequencing of orofacial movements most evident in speech. Structural neuroimaging in the affected members revealed bilateral abnormalities in a number of motor, and speech and language-related brain regions. Functional neuroimaging during verb generation and repetition tasks disclosed a distinctly atypical pattern of activation (i.e., diffuse, bilateral, and located predominantly in posterior cortical regions). Comparison between affected and unaffected family members indicated that the FOXP2 mutation is associated with significant underactivation in several regions that had been found to be morphologically abnormal, including the inferior frontal gyrus. Together, these structural and functional MR results provide a coherent explanation for the affected members' striking and persistent disorder.

The neuropsychological and neuroimaging findings will be related to the genetic basis of the disorder in the KE family and to that of other cases with mutations and/or deletions of the FOXP2 gene, and to the pattern of gene expression during embryological development in humans, mice and songbirds. The convergence of data from the behavioural, neuroimaging, and gene expression studies lead to a tentative model of the neuroanatomy of the speech and language system.

# Abstract: EARLY DAMAGE TO THE ORBITAL FRONTAL CORTEX IN MONKEYS ALTERS EMOTIONAL REACTIVITY, CHOICES GUIDED BY REWARD VALUE, AND SOCIAL BEHAVIOR

**Authors:** Jocelyne Bachevalier

**Affiliations:** Yerkes National Primate Research Center and Department of Psychology, Emory University, Atlanta, GA

**Background**: An increasing number of studies using a variety of experimental procedures in both animals and humans have demonstrated the significant contribution made by the orbital frontal cortex to the flexible monitoring of actions based on rewards processing. Yet, much remains to be discovered about the role played by this structure in the development of emotional responses and goal-directed behaviors, which are the prerequisites for the development of complex social behavior.

**Methods and Results**: Three studies will be presented investigating behavioral and cognitive changes following neonatal damage of the orbital frontal cortex in infant rhesus monkeys. The first study demonstrates that neonatal orbital frontal lesions alter the modulation of fear and defensive responses towards threatening social stimuli, indicating poor modulation of social stimuli. These findings were confirmed by a second study showing that these same neonatal lesions disrupted choice selection predicted by affective signals but not by visual signals conveying reward contingency. The last study investigated how the deficits in flexible monitoring of social cues will affect dyadic social interactions. The data indicated a lack of interests in initiating and maintaining social contacts with operated-controls in animals with neonatal orbital frontal lesions.

**Discussion**: These results are consistent with orbital frontal damage altering the complex and flexible monitoring of the reward values of emotional and social cues to select appropriate actions, even when the damage occurs in infancy. These functional alterations may in turn reduce general motivation to engage in social interactions. These experimental data shed some lights into the crucial role of this cortical area in developmental psychopathology.

# Abstract: OFF-LINE MEMORY PROCESSING, AS ASSESSED BY FUNCTIONAL NEUROIMAGING

**Authors:** Pierre Maquet

**Affiliations:** Cyclotron Research Centre, University of Liége, Belgium

Memory consolidation is the process by which fresh, initially labile, memories are reorganized into enduring stable memories. At the systems level, memory consolidation results in a progressive rearrangement of memories which can eventually be stored in circuits different from those in which they were initially encoded. For instance, declarative memories, originally heavily dependent on mesio-temporal structures, are thought to be gradually restructured in mature memories stored in more distributed cortical networks.

A growing body of data suggests that sleep is involved in the consolidation of declarative memories. We will provide evidence from fMRI studies that sleep deprivation hinders the plastic changes that normally occur during sleep and alters the brain responses subsequently recorded at retest. The basic mechanism underpinning memory consolidation seems to consist in the replay of firing sequences in neuronal populations involved in learning. The replay of firing sequences in neuronal populations involved in learning seems to underpin systems-level memory consolidation during sleep. Evidence for a replay of neuronal firing sequences has been collected in rodents during both NREM and REM sleep within the hippocampus, as well as in the neocortex. Consistent with data collected in rodents, we reported taskrelated experience-dependent increases in the hippocampal and neocortical activity in humans during NREM sleep following spatial learning.

An “offline” coordinated reactivation of distributed components of memory traces has also been observed during immediate post-training wakefulness in non human Primates and rodents. Likewise, using fMRI in humans, we were recently able to indirectly identify learning-related changes in spontaneous brain activity during wakefulness, by characterizing the modulation they impose on responses to an unrelated cognitive challenge.

# Abstract: SATELLITE MEETING TO THE EBBS 2007 CONFERENCE 15 SEPTEMBER 2007

**Authors:** Stress, Brain and Behaviour

**Affiliations:** Recent Developments on Pharmacology, Modelling and Translational Issues

The goal of this meeting is to shed new light on the mechanisms underlying the effects of stress on brain and behaviour with a focus on translational issues, novel behavioural and computational tools, and potential therapeutic approaches. In addition, an important goal of this meeting is to promote discussions.

# Abstract: I Translational Issues  THE NEUROBIOLOGICAL CONSEQUENCES OF STRESS-A TRANSLATIONAL APPROACH

**Authors:** E. Fuchs

**Affiliations:** Clinical Neurobiology Laboratory, German Primate Center, Göttingen, Germany

When Hans Selye formulated his stress theory about 70 years ago, stress was thought to have a merely endocrine character, and noxious stimuli of physical or chemical nature were the primary stressors discussed in those days. Subsequent research, however, demonstrated that psychological stimuli are also strong activators of the endocrine system and there was increasing evidence that ‘stress hormones’ such as adrenocortical steroids profoundly influence brain excitability. Today the stress response is regarded as an alarm system which is initiated whenever there is a discrepancy between what an organism is expecting and what really exists. Uncertainty, lack of information or loss of control induce alarm reactions whereas the presence of social support, information and control reduce the alarm reactions. Therefore, the stress response per se is not harmful or pathological in itself. Only when demanding, prolonged and sustained, body and brain homeostasis can be threatened, and health may be endangered. Since this is a consistent finding across species including man, it is important to understand the relationship between stressors and diseases. To refine our knowledge of the underlying processes neurobiological theories emerging from preclinical experimentation are tested on human subjects (bench to bedside) or vice versa where information obtained from investigations in human subjects can be used to improve our understanding of the biological principles (bedside to bench). Examples of this translational approach are presented and discussed in the first part of the Satellite Meeting starting with investigations in nonhuman primates and leading to investigations in human subjects. The intent is not only to summarize facts on the role of the brain in the stress response but also to promote discussion for future studies that will lead to the better mechanistic understanding of stress-related disturbances and their treatment.

# Abstract: NEUROPSYCHOLOGICAL ASSESSMENT IN NON-HUMAN PRIMATES: CONTRIBUTION TO UNDERSTANDING THE PHYSIOLOGICAL AND PATHOLOGICAL EFFECTS OF STRESS IN HUMANS

**Authors:** C. R. Pryce

**Affiliations:** Neuroscience Research, Psychiatry, Novartis Institutes for BioMedical Research, Novartis Pharma AG, Basel, Switzerland

Stress is an adaptive state in the short-term, and is associated with the aetiology and course of many diseases, including neuropsychiatric disorders such as depression and posttraumatic stress disorder, in the long-term. Neuropsychological processes that are enhanced by stress include motivation to avoid punishment, selective attention, and memory consolidation; and processes that are inhibited by stress include motivation for reward, behavioural inhibition, divided attention, executive functioning and memory recall. Because of the development of tasks based on non-verbal responses to visual stimuli, each of these neuropsychological processes can be studied using very similar paradigms in humans and nonhuman primates. This, combined with the high monkey-human homology in terms of (1) the physiology and neurobiology of stress, and (2) the neurobiological circuitry underlying behavioural processes, allows for reliable translation of findings between monkey and human. Examples of the study of effects of stress on neuropsychological functioning will be presented for the common marmoset, squirrel monkey and rhesus macaque, as will ideas for future research.

# Abstract: STRESS AND HUMAN EXPLICIT MEMORY: EFFECTS ON ENCODING, CONSOLIDATION AND RETRIEVAL

**Authors:** L. Nadel, S. Hoscheidt, E. Jackson, J. D. Payne

**Affiliations:** University of Arizona, Tucson, AZ, USA

The effects of stress on human memory was investigated in a series of behavioral experiments. We compared the impact of socially-induced stress on emotional and neutral material, mostly using variants of the Reisberg paradigm, in which subjects are presented narrative materials. Stress was induced either prior to encoding, after encoding (and hence during consolidation), or during retrieval. The impact of stress varied both as a function of the material being learned, and the stage of learning during which stress was induced. Under some conditions gender effects can be observed. We discuss the implications of these results for understanding how stress differentially influences multiple learning and memory systems.

# Abstract: SEROTONIN MODULATION OF MOTIVATION, EMOTION AND MEMORY IN HUMANS: HOW, WHEN AND WHY?

**Authors:** J. F. W. Deakin, I. M. Anderson, K. J. Lythe, M. E. Palm, R. Elliott

**Affiliations:** Neuroscience and Psychiatry Unit, University of Manchester, UK

Deakin and Graeff suggested that different 5HT subsystems are engaged by aversive events with two principal functions a) dorsal raphe nucleus (DRN) - 5HT2c projections to amygdala and basal ganglia serve to prevent the occurrence of future aversive events by motivating and directing avoidance behaviour and b)median raphe (MRN)- 5HT1A projections to hippocampus minimise the impact of current and past aversive events, by uncoupling acute stress responses and preventing rumination. We have used pharmacoMRI in volunteers and in antisocial and anxious individuals to test some of these ideas. Intravenous administration of the 5HT2c agonist mCPP evokes subjective anxiety and MRI responses in amygdala and caudate that are blocked by oral pre-treatment with mirtazepine, a 5HT2c antagonist. Tryptophan depletion reduces central 5HT neurotransmission and selectively reduced the detection of fear in face emotion processing. Increased 5HT release induced by citalopram has the opposite effect. These findings are predicted by the DRN-5HT2c fear hypothesis. However, amygdala fMRI responses evoked by fearful faces were attenuated rather than enhanced by iv citalopram pre-treatment. 5HT modulation of MRI activations evoked by go/no-go, reward, worry and social tasks and experiments in the clinical groups will be presented.

## References

[1] Deakin JFW, Graeff FG, Rodgers RJ (1991). Critique: 5-HT and mechanisms of defence. *Journal of Psychopharmacology*.

[2] Deakin JFW (1996). 5-HT, antidepressant drugs and the psychosocial origins of depression. *Journal of Psychopharmacology*.

[3] Lowry CA, Johnson PL, Hay-Schmidt A, Mikkelsen J, Shekhar A (2005). Modulation of anxiety circuits by serotonergic systems. *Stress*.

# Abstract: II Behaviour and Modelling  BEHAVIOUR AND MODELING

**Authors:** R. Dolan

**Affiliations:** Wellcome Trust Centre for Neuroimaging, UCL, London, UK

A fundamental principle in the organization of complex organisms is that of homeostasis. Perturbation in homeostasis provides a metric of the degree to which ongoing environmental interactions are stressful. The brains response to stress involves central cognitive repertoires (including appraisal, arousal, prediction, cognitive control) as well as peripherally mediated responses mediated via autonomic nervous and hormonal systems (a rapidly acting neural and a slow acting hormonal mechanism). Ideally both act in concert to integrate short and long term motivational needs in relation to current and, anticipated, future states of the environment. A maladaptive response to acute stress, or the emergence of chronic stress, is known to exert a profound negative impact on psychological and physical well-being leading to disorders such as depression, anxiety and cognitive under-function. Thus, key issues in understanding the impact of stress include the relationship between stress and aversive processing, the influence of neuro-humoural responses on neuronal plasticity, neurogenesis, as well as how stress interacts with individual genetic endowment. This mini-symposium will address each of these themes.

# Abstract: COMPUTATIONAL MODELS OF AVERSIVE PROCESSING: SEROTONIN, DOPAMINE AND STRESS

**Authors:** P. Dayan

**Affiliations:** Gatsby Computational Neuroscience Unit, University College London, UK

A wealth of evidence supports the notions that (i) the phasic activity of dopamine cells reports a temporally sophisticated prediction error for the delivery of rewards; that (ii) targets of these cells, including the amygdala and ventral and dorsal striatum, are involved in learning about and representing both appetitive predictions of future rewards and also the actions that will result in maximizing their delivery; and that (iii) there is a complex interaction between this scheme for the control of behaviour, which is associated with outcome-insensitive habits, and a model-based scheme, associated with outcome-sensitive or goal-directed actions, which appears to be realized in prefrontal structures. Despite an impressively extensive range of experimental findings, our understanding of the nature and realization of aversive processing appears to be rather less far advanced. One possibility is that serotonin might play a role as the sort of aversive opponent to dopamine that has been mooted and investigated from a psychological perspective. However, appetitive and aversive processing are far from being mirror images of each other psychologically, and indeed dopamine and serotonin, despite evidence for some mutually inhibitory interactions, are far from being mirror images of each other neurally. We will provide a computational examination of aversive processing in general, and opponency in particular.

# Abstract: A SYSTEMS-LEVEL MODEL OF STRESS EFFECTS ON COGNITION

**Authors:** J. B. Kim, T. J. Yoon, H. T. Blair

**Affiliations:** ^1^Department of Psychology and Programin Neurobiology & Behavior, University of Washington, Seattle, Washington, USA; ^2^Department of Psychology, University of California, Los Angeles, California, USA

Stress is a biologically significant social-environmental factor that plays a pervasive role in our lives, from impacting our daily behaviors to producing and exacerbating myriad physical and mental illness. An accumulating body of evidence from human and animal studies reveals that while the acute response to stress (i.e., heightened cognition) is an adaptive mechanism, exposures to uncontrollable (unpredictable and inescapable) stress can subsequently produce detrimental neurocognitive effects, particularly in the hippocampus. Rodent studies further indicate that stress impairs longterm potentiation (LTP), a leading candidate cellular mechanism of information storage, in the hippocampus. We have recently discovered that amygdala lesions/inactivation and prefrontal cortex lesions block and exacerbate, respectively, stress-induced impairments in hippocampal LTP and spatial memory. Moreover, single unit recording data indicate that stress alters the firing rate of place cells recorded from dorsal hippocampus, providing an empirical bridge between stress effects on synaptic plasticity and spatial memory. Based on these findings, we will present a conceptual model of the central stress mechanism (a neural-endocrine network comprising of amygdala, prefrontal cortex and glucocorticoids) regulating hippocampal functioning.

# Abstract: THE INTERACTION BETWEEN AFFECTIVE TRAITS AND STRESS IN LEARNING: EXPERIMENTS AND MODELLING

**Authors:** G. Luksys, C. Sandi

**Affiliations:** Brain Mind Institute, Swiss Federal Institute of Tchnology (EPFL), Lausanne, Switzerland

Acute stress regulates different aspects of behavioural learning through the action of stress hormones and neuromodulators. Stress effects depend on its type, intensity, timing, and the learning paradigm. However, animals with different affective traits (such as anxiety, impulsivity, and novelty reactivity) can be affected by stressors in different, sometimes even opposing ways. In the first part of the talk we will give an overview of rat and mouse experiments, showing a variety of examples, how affective traits may influence stress effects on learning and memory. Our animals, characterized prior to learning experiments, are tested on different tasks, where stress type, intensity, and timing are manipulated. In addition, we relate behavioural effects to levels of stress hormones (glucocorticoids), providing evidence that differential stress reactivity is really responsible for differences in learning. In the second part of the talk, we will describe how mouse behaviour can be formalized and studied using reinforcement learning (RL) models. In this framework, the effects of stress and genetic background on learning and memory can be attributed to differential dynamics of model meta-parameters (such as learning rate or exploitation-exploration factor), which are thought to be somehow related to activity of neuromodulators in the brain. We will provide the main results of RL meta-parameter studies and discuss benefits and perspectives of such approach.

# Abstract: III Pharmacology  STRESS IN DEPRESSION–PRIMARY CAUSE OR EPIPHENOMENON?

**Authors:** T. Steckler

**Affiliations:** Department of Psychiatry, R&D Europe, Johnson & Johnson PRD, Beerse, Belgium

Abnormal stress resilience has been considered as an important factor leading to depression and anxiety disorders. Corroborating evidence comes fromstudies as discussed by Oitzl et al., linking genetic vulnerability and stress exposure to the expression of fear-related behaviours in mice. Likewise, chronic stress has been shown to alter serotonergic function and gene expression related to neuroplasticity (Flugge and Abumaria), thought to play a role in depression and anxiety. Consequently, compounds targeting stress responsivity and HPA axis abnormalities have been proposed as potential novel therapeutic approaches with improved efficacy to treat depression and anxiety disorders (Thomson et al.). Fundamental to this idea is the hypothesis that abnormalities in stress systems represent a final common pathway in these disorders. Here we will discuss whether indeed this is the case. A full understanding of the relationship between stress reactivity and anxiety and depression is pertinent as this relationship will determine whether treatment strategies aimed at stress responsivity will be best positioned as monotherapy to treat the anxiety and depression, or whether they require co-medication with other drugs, whether their primary use will be during the acute phase, or whether they may be of prime benefit in preventing relapse. Further, by broadening the concept of altered stress reactivity beyond anxiety and depression, it can be expected that these types of compounds will also be of benefit in other disorders, including drug abuse, psychosis and sleep disorders.

# Abstract: IMPACT OF STRESS ON GENE EXPRESSION RELATED TO SEROTONERGIC NEUROTRANSMISSION: REGULATION BY THE ANTIDEPRESSANT CITALOPRAM

**Authors:** G. Flügge, N. Abumaria

**Affiliations:** Clinical Neurobiology Laboratory, German Primate Center and DFG Research Center, Molecular Physiology of the Brain (CMPB), University of Göttingen, Göttingen, Germany

It is well known that acute stress activates the serotonergic (5-HT) system and increases tryptophan hydroxylase (TPH) activity in the dorsal raphe nucleus (DRN). We studied the effects of chronic social stress and a chronic treatment with the specific serotonin-reuptake inhibitor (SSRI) citalopram on TPH expression in the DRN using a resident-intruder paradigm. Male Wistar rats were daily confronted with a dominant Lister Hooded rat during five weeks and received citalopram (via drinking water; 30 mg/kg/day) during four weeks starting on week 2 of the stress period. Citalopram normalized several stress-induced behavioral parameters, e.g. performance in the sucrose preference test ([1]).

Quantitative Real-time PCR and Western blotting confirmed previous data revealing strong TPH2 expression in the DRN but showed also that TPH1 is weakly expressed in this nucleus. Expression of the two TPH genes was found to be differentially regulated: TPH1 mRNA was increased more than three-fold by chronic stress and normalized by chronic citalopram. In contrast, TPH2 mRNA expression was not affected by stress but was downregulated by citalopram (to less than 50%) in both stressed and control animals. TPH protein was upregulated by stress exposure and the effect was prevented by citalopram. The SSRI had no effect on serotonin transporter mRNA but reduced 5-HT1A autoreceptor mRNA in stressed animals. These data shed new light on presumptive mechanisms underlying the beneficial effects of citalopram, and possibly other SSRIs, in rebalancing the central nervous 5-HT system. Moreover, synaptic vesicle protein 2b mRNA and protein were increased by chronic stress and normalized by the antidepressant indicating that the SSRI has a balancing effect on the neurotransmitter release machinery. Citalopram prevented the stress-induced upregulation of mRNA for CREB binding protein, an important factor in CREB-regulated transcription. In conclusion, our data demonstrate that in the rat DRN, citalopram blocks effects of chronic stress on distinct genes related to neurotransmitter release and neuroplasticity ([2]).

## References

[1] Rygula R, Abumaria N, Flügge G (2006). Citalopram counteracts depressive-like symptoms evoked by chronic social stress in
rats. *Behavioural Pharmacology*.

[2] Abumaria N, Rygula R, Hiemke C (2007). Effect of chronic citalopram on serotonin-related and stress-regulated genes in
the dorsal raphe nucleus of the rat. *European Neuropsychopharmacology*.

# Abstract: COGNITION AND EMOTION: ANTAGONIZING STRESS SYSTEM ACTIVITY IN A MOUSE MODEL OF STRESS RELATED DISORDERS

**Authors:** M. S. Oitzl, S. Dalm, V. Brinks

**Affiliations:** ^1^LACDR/LUMC Division of Medical Pharmacology, Leiden University, Leiden; ^2^SILS Center for Neuroscience, University of Amsterdam, Amsterdam, The Netherlands

Stress precipitates affective disorders like depression and anxiety. Genetic factors are implicated, but individual vulnerabilities depend on gene*life-events interactions. A fundamental question in biomedical research addresses the molecular mechanisms of stress system disorders. Glucocorticoid stress hormones acting via mineralocorticod (MR) and glucocorticoid receptors (GR) in the brain are in the center of interest. They modulate differential aspects of affective expressions and cognition; facilitating as well as impairing actions on memory have been reported. Based on animal models, I will discuss the action of corticosteroids and their antagonism. BALB/c and C57BL/6 mice with a distinct profile of central and peripheral markers of stress system activity will be used to demonstrate, that glucocorticoids and genetic make-up contribute in a distinct fashion to the expression of different fear behaviours, and the acquisition, consolidation and extinction of fear memories. The influence of chronic stress on emotional expressions, responses to novelty, learning and memory in C57BL/6 mice will be demonstrated in relation to the effects of the GR antagonist Mifepristone. Supported by grants of the Dutch Organization for Scientific Research NWO 015.01.076 and NWO-IRTG DN 95-420.

# Abstract: INNOVATIVE APPROACHES FOR THE TREATMENT OF DEPRESSION: TARGETING STRESS AND THE HPA AXIS

**Authors:** F. J. Thomson, M. Craighead, H. M. Marston, C. MacSweeney, S. Napier

**Affiliations:** ^1^Department of Molecular Pharamacology; ^2^Department of Pharmacology; ^3^Department of Chemistry, Organon, Laboratories Ltd, Newhouse, UK

Depression is a major cause of disability yet, in spite of over 50 years of research effort a substantial increase in the clinical efficacy of antidepressants has not been achieved. This observation is unsurprising, considering that all antidepressant drugs modulate neurotransmission as a common mechanistic endpoint. It is intriguing to speculate that improvement of drug efficacy may only emerge when pharmacological agents acting at nonmonoamine drug targets are developed. Further, our lack of understanding of the mechanisms underlying depressive illness is a major hurdle in our quest towards developing better antidepressants. Chronic exposure to stressful life events in combination with susceptibility factors is suggested to contribute to an individual developing depression. Exposure to stress results in activation of the hypothalamic pituitary adrenal (HPA) axis and hyperactivity of this system is the most consistently described neuroendocrine abnormality in depression. Irregularities include elevated cortisol concentrations, dysfunctional glucocorticoid receptor activity, blunted corticotrophin releasing factor (CRF) receptor-1 function and increased sensitivity to arginine vasopressin (AVP). Observations that clinical remission is associated with normalisation of HPA axis activity suggest that drugs that intervene with this system could have benefit for the treatment of depression. Recently, selective inhibitors of the vasopressin V1b receptor have emerged which, by counteracting vasopressinergic hyperdrive of the HPA axis, are proposed to have potential as a novel antidepressant agent. Here I will describe the pharmacology and preclinical behavioural profile of a novel, high affinity selective V1b receptor antagonist and speculate on the challenges for successful clinical application of such a drug.

# Abstract: SYMPOSIA S1: The retrosplenial cortex: revealing its role in memory and limbic system function

*Organizers*: John Aggleton (School of Psychology, Cardiff University, Cardiff, Wales, UK), Thomas Van Groen (Department Cell Biology, University of Alabama at Birmingham, Birmingham, Ala, USA)

Even though there is growing evidence that the retrosplenial cortex contributes to a variety of cognitive functions, most especially memory, the significance of the region has often been overlooked. The retrosplenial cortex is strategically placed anatomically as it is interconnected with the hippocampal region, anterior thalamic nuclei, and prefrontal cortex. For this reason, it may serve to link temporal, diencephalic, and prefrontal regions involved in memory. Consistent with this notion are clinical reports of memory dysfunction associated with retrosplenial cortex damage, while functional imaging studies have begun to reveal its importance for aspects of episodic memory. In spite of its strategic position, it is only in very recent years that its potential importance has emerged.

In this symposium, we will present complementary evidence from anatomical, lesion, gene expression, and functional neuroimaging studies that all underline the importance of this region for limbic function. Rodent studies highlight how the retrosplenial cortex is heterogeneous and will ultimately need to be subdivided functionally. Related research is revealing the unusual vulnerability of this region to brain insult.

Studies using functional MRI in humans are beginning to reveal its operating mechanisms, and its contribution to the dynamics of the brain's memory system.

- **Thomas Van Groen** (University of Alabama at Birmingham, Ala, USA) Anatomy of the anterior thalamus–retrosplenial system and its role in memory
- **Seralynne Vann** (Cardiff University, UK) Functional subdivisions within the rodent retrosplenial cortex
- **John Aggleton** (Cardiff University, UK) Evidence for cryptic pathology in the retrosplenial cortex and its potential contribution to memory disorders
- **Eleanor Maguire** (University College London, UK) What can functional neuroimaging studies in humans tell us about the functions of retrosplenial cortex?

# Abstract: ANATOMY OF THE ANTERIOR THALAMUSRETROSPLENIAL SYSTEM AND ITS ROLE IN MEMORY

**Authors:** T. Van Groen

**Affiliations:** University of Alabama at Birmingham

In the limbic system, many components such as the retrosplenial cortex and the anterior thalamic nuclei are heavily interconnected. Furthermore, these brain areas are part of the so-called Papez Circuit, which originally had been envisioned to play a role in emotions and emotional expression. Whereas components of the limbic system, such as the amygdala, are involved in emotions, the Papez circuit most likely only plays a minor role in emotions, however, in contrast it does play a major role in learning and memory, especially, spatial learning and memory. The caudal, midline limbic cortex, the retrosplenial cortex consists of three areas, retrosplenial granular a and b, and retrosplenial dysgranular cortex, whereas the anterior thalamus consists of four nuclei, the anterior dorsal, anterior ventral, anteriormedial nuclei and the laterodorsal nucleus. Each cortical area has its own specific connections with each of the four anterior thalamic nuclei, and most of these connections are reciprocal. Furthermore, the retrosplenial cortices are themselves interconnected, they project to the hippocampal formation, and the subicular cortex projects directly and indirectly, through the mamillary bodies, to the anterior thalamic nuclei, which project to the retrosplenial cortex, i.e., the Papez circuit. To better understand the functions of this circuit and its components, we and others, have done a series of learning and memory studies following lesions of components of this circuit. In simple terms, small lesions involving only one component of the system do not lead to measurable behavioral deficits, only lesions involving several parts lead to learning and memory deficits, with a direct correlation between lesion size and amount of spatial learning deficit. Together these data lead to the view that the retrosplenial cortex-anterior thalamus system form a network of parallel pathways, that can partially compensate for loss of one pathway.

# Abstract: FUNCTIONAL SUBDIVISIONS WITHIN THE RODENT RETROSPLENIAL CORTEX

**Authors:** S. D. Vann

**Affiliations:** School of Psychology, Cardiff University, Park Place, Cardiff CF10 3AT, UK

It is now recognised that the retrosplenial cortex plays a role in spatial memory. The rodent retrosplenial cortex is, however, an extensive structure and initial lesion studies may have underestimated the importance of the retrosplenial cortex by sparing the more caudal part of this region. Subsequent research has shown that the size of retrosplenial cortex lesions result is crucial in determining the extent of impairments on spatial memory tasks, and evidence from both lesion and immediate-early gene studies have implicated the entire rostral-caudal extent of this structure in spatial memory. In addition to rostro-caudal divisions within the retrosplenial cortex there are distinct cytoarchitectonic subregions within this structure with different patterns of connections. The two major subdivisions comprise granular and dysgranular retrosplenial cortex, with granular being separated further into granular a and b. Selective lesions of the dysgranular retrosplenial cortex impair allocentric performance of the radial-arm maze, consistent with the fact that this subregion is the primary recipient of visual inputs within the retrosplenial cortex. Finally, differential involvement of all the subregions within the retrosplenial cortex (granular cortex a, granular cortex b, dysgranular) at both rostral and caudal levels has been shown using immediate-early gene activation in normal rats performing a spatial working memory task. Together, these data highlight the functional heterogeneity of the retrosplenial cortex, consistent with the known anatomical connectivity.

# Abstract: EVIDENCE FOR CRYPTIC PATHOLOGY IN THE RETROSPLENIAL CORTEX AND ITS POTENTIAL CONTRIBUTION TO MEMORY DISORDERS

**Authors:** J. P. Aggleton

**Affiliations:** Cardiff Universit

There is growing evidence that the retrosplenial cortex is unusually vulnerable to the loss of its distal connections. One of its major afferent sources is the anterior thalamic nuclei complex. Previous studies have shown that large rostral thalamic lesions do not induce cell loss in the retrosplenial cortex, but reduce cytochrome oxidase levels and markers for acetyl choline. In a series of studies with rats we have found that the expression of the immediate-early genes (IEGs) c-fos and zif268 show a dramatic, and permanent, fall in retrosplenial cortex following selective, anterior thalamic lesions. This IEG loss, which was most marked in the superficial layers of the granular retrosplenial cortex (up to 90% loss), is found irrespective of the lesion method, strain of rat, and time post surgery. Subsequent in situ hybridrisation studies have confirmed the depletion of c-fos RNA in these superficial levels while microarray studies have provided a fuller picture of the retrosplenial cortex changes consequent to anterior thalamic damage. Electrophysiological studies of retrosplenial slices taken from rats with previous anterior thalamic lesions reveal a loss of plasticity (long term depression) in the local circuits within the superficial layers of granular retrosplenial cortex. These findings of cortical hypoactivity and a loss of plasticity not only indicate a covert pathology but may also be directly relevant for a number of neurological conditions (Korsakoffs disease, Mild Cognitive Impairment, Alzheimers disease) that are characterised by both memory loss and prominent pathology in sites connected with the retrosplenial cortex.

# Abstract: WHAT CAN FUNCTIONAL NEUROIMAGING STUDIES IN HUMANS TELL US ABOUT THE FUNCTIONS OF THE RETROSPLENIAL CORTEX?

**Authors:** E. A. Maguire

**Affiliations:** Wellcome Trust Centre for Neuroimaging, Institute of Neurology, University College London, UK

Often eclipsed by some of its more celebrated neighbours such as the hippocampus, the retrosplenial cortex has nevertheless maintained its standing as one of the key brain regions classically comprising the limbic system. Patients with retrosplenial cortex lesions suffer severe topographical and navigational deficits as well as, in some cases, amnesia. Moreover, neuroimaging studies have consistently observed activation in this structure during navigation, spatial, and episodic memory tasks. Despite such findings, however, determining the exact function of the retrosplenial cortex has proved difficult. The latest functional neuroimaging data will be reviewed, and possible theoretical frameworks considered that may shed some light on this mysterious brain region.

# Abstract: S2: Cannabinoids: what in vitro studies have not told us

*Organizers*: Gernot Riedel (University of Aberdeen, Aberdeen, UK), Robert E. Hampson (Department of Physiology & Pharmacology, Wake Forest University School of Medicine, Winston-Salem, NC, USA)

Recently, the food and drug agency (FDA) qualified a novel cannabinoid receptor antagonist from Sanofi-Aventis as approvable in the treatment of obesity and addiction and this has brought research into endocannabinoid function back into center stage. Recent advances in understanding the function of the endocannabinoid system in the brain, however, mainly arose from studies using in vitro, ex vivo, and cellbased assays. These, however, often fail to be confirmed by in vivo investigations, for example:

(1) In vitro slice work has now firmly established that cannabinoids contribute to depolarization-induced suppression of inhibition (DSI) in the hippocampus. However, recordings from freely moving animals show that induction protocol for DSI is nonphysiological and the phenomenon may not exist in a living animal, with the corollary that endocannabinoids may not contribute to this mechanism.

(2) Cell-based assays show that very low doses of cannabis (or synthetic agonists of cannabinoid receptors) are neurotoxic, while higher doses are neuroprotective. At the same time, low doses have little or no effect on memory formation although this would be expected given that hippocampal cells are highly vulnerable to this treatment. By contrast, high doses of cannabinoids that should protect neurons in fact lead to memory impairment.

These two examples highlight the need for a wider and more detailed analysis into the function of the endocannabinoid system using systemic approaches. Towards this end, we have selected four important areas of research from which a major contribution toward a richer understanding of cannabinoid function and potential treatments can be expected. These areas include cognition, physiology of cell ensembles, neuroimaging, and addiction. At the same time, these areas cannot be simply substituted by in vitro models.

- **Liana Fattore** (CNR Institute of Neuroscience, Cagliari, Italy) Endocannabinoids and addiction: a chance for some treat
- **Andrew Horti** (Johns Hopkins University, Md, USA) [11C]JHU75528, a PET radioligand for imaging of cerebral cannabinoid CB1 receptors
- **Robert E. Hampson** (Wake Forest University School of Medicine, Winston-Salem, NC, USA) The role of cannabinoids in neural ensemble responses to behavioral conditions
- **Gernot Riedel** (University of Aberdeen, Aberdeen, UK) Endocannabinoid function in cognition

# Abstract: ENDOCANNABINOIDS AND ADDICTION: A CHANCE FOR SOME TREAT

**Authors:** Liana Fattore, Maria Sabrina Spano, Paola Fadda, Walter Fratta

**Affiliations:** ^1^Institute of Neuroscience, National Research Council CNR c/o; ^2^Department of Neuroscience, University of Cagliari; ^3^Centre of Excellence “Neurobiology of Dependence”, Cagliari, Italy

Addiction is a complex neurobiological cycle, in which positive reinforcement exerted by the drug and the negative state of withdrawal drive the user to extremes to obtain the drug. Repeated drug use easily results in addiction where successful treatment is deterred by high incidence of relapse which remains the primary problem in treating drug dependence. Vulnerability to relapse after withdrawal is proposed to be the result of neuroadaptive processes within the brain which lead to impairment in the mechanisms that mediate the perceived value of the drug and the appearing of emotive changes such as anxiety and dysphoria during withdrawal. Although of some help, medications available to date do not completely prevent the probability of returning to compulsive drug-seeking, leaving patients vulnerable to drug use relapse. Thus, the identification of new pharmacological anticraving treatments for use in the maintenance of patients in a drug-free state is a priority issue. Over the last years, we and others demonstrated that the endocannabinoid system is crucially involved not only in the modulation of the reinforcing effects of most widely abused drugs but it also plays a key role in the regulation of relapsing episodes. Indeed, while cannabinoid CB1 receptor stimulation may elicit relapse not only to cannabinoid seeking but also to cocaine, heroin, alcohol and methamphetamine, this effect is significantly attenuated, if not fully prevented, by pretreatment with the CB1 receptor antagonist rimonabant. The conspicuous progress recently made in delineating the role of the endocannabinoid system in relapse to drug seeking has important consequences for human situations, since the risk of relapse in an abstinent individual may be significantly attenuated by CB1 antagonist preventive treatment.

# Abstract: [11C]JHU75528, A PET RADIOLIGAND FOR IMAGING OF CEREBRAL CANNABINOID CB1 RECEPTORS

**Authors:** A. G. Horti, H. Fan, H. T. Ravert, D. Holt, J. Hilton, A. Kumar, M. Alexander, A. Rahmim, A. F. Hoffman, C. R. Lupica, H. Kuwabara, D. F. Wong, R. F. Dannals

**Affiliations:** ^1^Department of Radiology, Johns Hopkins University, USA; ^2^NIH, NIDA, USA

Radioligands for positron-emission tomography imaging of cerebral cannabinoid receptors (CB1) are important for studying the role of CB1 in neuropsychiatric disorders, as well as for developing cannabinergic medications to treat these conditions. We developed a novel high affinity CB1 antagonist JHU75528 (JHU) with reduced lipophilicity. JHU displayed a higher binding affinity than that of SR141716 in the same assay (Ki = 15 nM vs. Ki = 40 nM). Extracellular electrophysiological recordings in rodent brain slices showed that JHU is a functional CB antagonist. Radiolabeled 11C-JHU readily entered the animal brain and specifically labeled CB1 with high binding potential (BP). The highest accumulation of radioactivity in the mouse brain occurred in the CB1-rich regions with the highest striatum-to-brainstem ratio (3.4) ever reported for a CB1 radioligand. Pre-administration of SR141716 inhibited 11C-JHU binding in all regions of the mouse brain examined, except the thalamus, a region with low density of CB1. A robust incorporation of radioactivity into the baboon brain was seen in a PET study after injection of 11C-JHU. The highest accumulation of radioactivity occurred in the putamen, frontal cortex and cerebellum and the lowest level of radioactivity was detected in the thalamus and pons. Injection of SR141716 reduced regional radioactivity concentrations in the regions with high and intermediate density of CB1 to the level in pons. Essentially a complete blockade in the CB1-rich brain regions after administration of SR141716 confirmed specific binding of 11C-JHU at CB1 receptors in vivo. The BP value of 11CJHU in baboon putamen obtained with the simplified tissue reference was 1.5. This value is substantially higher than that published for [123/124I]AM281 (lentiform nucleus, BP=0.2-0.4), the only available radioligand for CB1 tomographic imaging in humans. The overall results suggest that 11C-JHU holds promise as a successful CB1 radioligand for PET imaging in humans.

# Abstract: THE ROLE OF CANNABINOIDS IN NEURAL ENSEMBLE RESPONSES TO BEHAVIORAL CONDITIONS

**Authors:** R. E. Hampson

**Affiliations:** Wake Forest University School of Medicine, Winston-Salem, NC, USA

In vitro studies reveal that endocannabinoids mediate retrograde signaling between principal neurons and presynaptic GABAergic and glutamatergic inputs. Depolarization-induced Suppression of Inhibition (DSI) is an endocannabinoid- mediated feedback whereby hippocampal pyramidal neurons (as well as principal neurons from other brain areas) suppress activity of GABA neuron inputs. Likewise, DSE (suppression of excitation) occurs on glutamate neuron inputs in brain regions other than hippocampus.

In vivo studies show that exogenously applied cannabinoids do not produce effects predicted by the DSI mechanism. Instead, exposure to cannabinoids suppresses activity of hippocampal pyramidal neurons and alters (inhibits) ensemble responses to behavioral events critical to DNMS performance. While some inhibition may be consistent with DSE, only DSI has been shown to operate in hippocampus. Alteration of neural circuits involving connected brain areas do not account for the fact that local application of cannabinoids to hippocampus alone produce similar effects to systemic application. Likewise, exposure to the CB1 receptor antagonist Rimonabant, or endocannabinoid synthesis or reuptake blockers also fail to produce effects predicted by the in vitro findings. While exposure to cannabinoids suppresses working memory in behavioral tasks such as DNMS, modulating endocannabinoid actions via antagonists and reuptake blockers often fail to produce effects on working memory or the neural ensemble activity underlying such memory.

Therefore, in vivo studies have more to reveal about the role cannabinoids in shaping neural responses to complex stimuli than can be predicted by in vitro studies alone. While DSI and DSE likely contribute to the effects of cannabinoids in vivo, there is still a missing link between activation of cannabinoid receptors and cannabinoid effects on memory and behavior.

# Abstract: ENDOCANNABINOID FUNCTION IN COGNITION

**Authors:** G. Riedel

**Affiliations:** School Medical Science, University of Aberdeen, Aberdeen, UK

A large number of previous studies have revealed that cannabinoids and the endocannabinoid system (ECS) are involved in learning and memory processes. These effects are dependent on intrinsic and extrinsic factors of the subjects and are often difficult to dissociate from effects of cannabinoids on motor function, appetite, general motility, psychoactive experiences, reaction times etc. A more uniform and less variable result can be obtained by utilising in vitro systems to investigate the actions of the cannabinoid system. While many of these investigations produce interesting data, they contribute little to our global understanding of the function of the ECS.

Two examples are selected to highlight a) the discordance of in vivo and in vitro results with respect to memory formation, and b) the difference between global and local in vivo actions of the ECS.

(a) Long-term potentiation (LTP) is a model of synaptic plasticity which is widely held to reflect the cellular underpinnings of memory formation. In hippocampal slices, cannabinoids impair LTP. We tested the synthetic cannabinoid receptor (CR) antagonist WIN 55,212-2 intraperitoneally in doses of 1–3 mg/kg and it impaired spatial learning in the water maze. WIN presumable acted via hippocampal CRs since micro-pump injectedWIN also impaired learning (8.9 ng/day). The same doses, however, reduced paired-pulse depression (PPD) of CA1 neurons ex vivo and enhanced long-term potentiation (LTP) in slices excised from treated animals. It would have been predicted based on in vivo results, that LTP was reduced in WIN-treated animals so that learning was impaired.

(b) AM281 is a selective antagonist for the neuronally expressed CRs. Hippocampal slices treated with CR antagonists show normal or reduced LTP. When administered systemically, AM281 impaired spatial learning in the water maze. At the same time, there was no effect of CR antagonists on paired-pulse depression or LTP when given alone. Local administration of AM281 into the hippocampus, however, facilitated learning and prolonged/protected spatial memory, similar to previous work.

Data presented suggest that extrapolation of in vitro results to predict in vivo effects of cannabinoids and the ECS is difficult, and that even systemic administration of drugs may provide only limited information as to the specific role of localised populations of CRs.

# Abstract: S3: Avian brain and cognition

*Organizers*: Toshiya Matsushima (Hokkaido University, Sapporo, Japan) and Onur Güntürkün (Ruhr-Universität Bochum, Bochum, Germany)

A common notion has been that birds' brains are simple, or so scientists thought and taught for many years. But that notion has increasingly been called into question. We now recognize that the avian brain is as complex, flexible, and inventive as any mammalian brain, and this has been reflected in the recent adoption of a more accurate nomenclature that also reflects a new understanding of the anatomies of bird and mammal brains. The aim of the symposium is to bring together some avian neurocognitive scientists that work on topics which are currently a focus of interest for scientists working with mammals. The arguments selected will include neuroeconomics (Toshiya), space and number cognition (Vallortigara), the convergent evolution of prefrontal brain structures in birds and mammals (Güntürkün), and the evolution of cognitive traits and brains as shaped by ecological pressures (Lefebre). As a whole, the papers will provide a concise state-of-the-art of the recent advancement on avian brain and cognition and a direct comparison with theories and methods which are in use in mammals.

- **Onur Güntürkün** (Ruhr-University Bochum, Germany) The convergent evolution of prefrontal “cortices” in birds and mammals
- **Louis Lefebvre** (McGill University, Montréal, Canada) Behavioural flexibility and selection for enlarged brains
- **Toshiya Matsushima** (Hokkaido University, Sapporo, Japan) Economical decision making in chicks: brain mechanisms meet foraging ecology
- **Giorgio Vallortigara** (University of Trieste, Italy) Space and number sense in newborn chicks

# Abstract: THE CONVERGENT EVOLUTION OF PREFRONTAL ‘CORTICES’ IN BIRDS AND MAMMALS

**Authors:** O. Güntürkün

**Affiliations:** Department of Biopsychology, Faculty of Psychology, Ruhr-University Bochum, Germany

Both mammals and birds are able to flexibly organize their behavior over time. Inmammals, the mental operations generating this ability are called executive functions and are associated with the prefrontal cortex. The corresponding structure in birds is the Nidopallium caudolaterale. Anatomical, neurochemical, electrophysiological, and behavioral studies show both structures to be highly similar. Since the avian forebrain displays no lamination that corresponds to the mammalian neocortex, lamination does not seem to be a relevant requirement for higher cognitive functions. Since all other aspects of the neural architecture of the mammalian and the avian prefrontal areas are extremely similar, degrees of freedom to create different neural architectures that generate prefrontal functions seem to be very limited.

# Abstract: BEHAVIOURAL FLEXIBILITY AND SELECTION FOR ENLARGED BRAINS

**Authors:** L. Lefebvre

**Affiliations:** Department of Biology, McGill University, Montréal, Québec, Canada

Contemporary neuroscience focuses on lab research of molecular mechanisms controlling brain functions in model systems, as well as imaging studies of localized areas involved in cognitive tasks, mainly in humans. Neuroecology, the branch of neuroscience that examines the evolution of brains and cognition in naturalistic settings, has recently focused on large comparative studies at broad neural levels like the whole brain or pallium. Is neurecology compatible with the rest of contemporary neuroscience? I will review recent results, using innovation rate as the main operational measure of cognition in the wild. Innovation rate is a good measure of behavioral flexibility, which is associated with reduced mortality, higher colonisation potential, greater species and subpecies diversity, as well as a greater ability to adapt to seasonal environments. Overall, the results suggest (1) convergent evolution in birds and primates, (2) positively correlated variation in different types of cognitive measures, which is compatible with domain-general intelligence, and (3) similar pressures of resource distribution and abundance on social and asocial cognition. The last two results suggest that comparative studies at the level of the whole brain are valid, even if specific cognitive abilities are controlled by localized centres and networks.

Funded by NSERC.

# Abstract: ECONOMICAL DECISION MAKING IN CHICKS: BRAIN MECHANISMS MEET FORAGING ECOLOGY

**Authors:** T. Matsushima

**Affiliations:** Laboratory of Animal Behavior and Intelligence, Department of Biology, Faculty of Science, Hokkaido University, Japan

Optimal foraging theory (Charnov 1976, Stephens & Krebs 1986) predicts that hypothesized omniscient/rational foragers evaluate each prey item by its profitability (or the ratio of energetic gain per handling time) in order to maximize the long-term gain rate. The underlying cognitive processes are basically identical to those of the impulsive choice of an immediate-small food item (the prey item) against a latelarge alternative (the lost opportunity). The foragers should therefore be able to precisely anticipate the profitability and control the level of impulsiveness accordingly. To address these issues, we examined weeks-old domestic chicks in operant behaviors, in which each of colored cue beads was associated with a food reward of certain amount (A), delay (D: either temporal or spatial) and consumption cost (C); both D and C were given by the period of time required for approaching and consuming the food item, respectively. Concurrent binary choice tests suggested a currency (V) as given by V = A/(D + C), somehow reminiscent to the Mazur's formulation of subjective value. Localized lesions of the nucleus accumbens (core) in ventral striatum enhanced the impulsiveness without impairments in the amount based choices, suggesting a shift toward proximity-dominant choices with a biased stress on D. Localized lesions of the arcopallium intermedium in the “cortex” analogue (association area with massive descending projections), on the other hand, caused costaversive choices with a biased stress on C. The lesion effects were doubly dissociated, suggesting that these two factors are distinct not only in their ecological relevance but also in their neural substrates. Understanding the neuro-cognitive mechanisms of choice could enable us to bridge among disciplines in ecology, economics and neuroscience.

# Abstract: SPACE AND NUMBER SENSE IN NEWBORN CHICKS

**Authors:** G. Vallortigara

**Affiliations:** Department of Psychology and B.R.A.I.N. Centre for Neuroscience, University of Trieste, Trieste, Italy

Studies on human infants, focused on the ontogenetic origins of knowledge, provided evidence for a small set of separable systems of core knowledge dealing with the representation of objects, number and space. We investigated core knowledge systems in comparative perspective, making use of the domestic chick as a model system and filial imprinting as a key to animal mind. We discuss evidence showing precocious abilities in the chick to represent (i) the cardinal and ordinal/sequential aspects of numerical cognition and (ii) the distance, angle, and sense relations among extended surfaces in the surrounding layout. Some of the abilities associated with core knowledge systems of number and space were observed in the absence (or with very reduced) visual experience.

# Abstract: S4: Automatic processing of emotion: role in higher cognitive processes

*Organizer*: Pascale Gisquet-Verrier (Université Paris-Sud, Orsay, France)

There is a general agreement to consider that cognition is supported both by implicit and explicit processes. Nevertheless, interactions between implicit and explicit processes remain largely unknown and are probably underestimated. Over the last few years, compelling evidence indicates that emotion driven by exteroceptive stimuli has a determinant influence on higher cognitive processes and particularly on memory processes. This symposium is aimed at illustrating this statement through different approaches coming from cognitive psychology and behavioural neuroscience, in animals and in human.

- **Pascale Gisquet-Verrier** (Université Paris Sud, Orsay, France) Neural basis of emotional cue-induced memory retrieval facilitative effect in rats
- **Magali Seassau** (Université Paris Sud, Orsay, France) Emotion can modulate memory retrieval in humans
- **Ray Dolan** (University College London, UK) Emotional influences on decision making
- **Adam Anderson** (University of Toronto, Canada) Emotion, attention and episodic memory

# Abstract: NEURAL BASIS OF EMOTIONAL CUE-INDUCED MEMORY RETRIEVAL FACILITATIVE EFFECT IN RATS

**Authors:** P. Gisquet-Verrier

**Affiliations:** CNRS UMR 8620, Université Paris Sud, 91400 Orsay, France

In the every day life, environmental cues play a determinant role in promoting memory retrieval. In rats, exposure to training-related cues is able to reactivate the associated memory, to reinstate disrupted memories, and to improve retention performance. Using a brightness avoidance discriminative task, we showed that exposure to training related cues has both behavioural and neural consequences. At a behavioural level, retrieval cues improve the subsequent retention performance. While the nature of the most efficient cue to promote retrieval varies according to the age of memory, their facilitative effects on retention performance have the same time development and can be reproduced in an appetitive version of the task. At a neural level, we showed that exposure to retrieval cues is accompanied by a release of plasmatic ACTH and activates brain structures including several amygdala and hypothalamic nuclei, the nucleus accumbens and the insular cortex. In addition, the retrieval cue facilitative effect requires the integrity of the anterior cingulate and the prelimbic part of the medial prefrontal cortex (mPFC) but not the hippocampus. In addition, the effects of the retrieval cues can be blocked by various treatments, including injections of a CRF antagonist (CP- 154,526), of the non-selective opiate antagonist naloxone. Exposure to retrieval cues is accompanied by dopamine release within the prelimbic cortex, and D1 antagonist infusion in this area abolishes the facilitative effect of retrieval cues. Interestingly, the processes involved are closed from those mediating cue-induced relapse in drug-seeking and cue-driven PTSD syndrome. It is proposed that exposure to environmental cues always activates an amygdalo-corticostriatal neural circuitry which is the support of memory reactivation, whatever the valence and intensity of the memory. The potential function and dysfunction of such emotional cueinduced memory retrieval will be further discussed.

# Abstract: EMOTION CAN MODULATE MEMORY RETRIEVAL IN HUMANS

**Authors:** Magali Seassau, Richard Levy, Pascale Gisquet-Verrier

We investigated whether emotion may exert a direct influence on the efficacy of memory retrieval. Subjects were submitted to an unexpected recognition test of affective pictures after an encoding phase during which theses pictures were associated with neutral background pictures. At the recognition phase, the background pictures were used as primes under implicit condition. The prime were either the background picture initially associated with the affective picture at the encoding phase or a background picture initially associated with another affective picture of the same valence or of a different valence. Four intervals of retention were used and subjects were randomly assigned to one of four groups according to the interval of retention. Reaction times were found shorter when the emotional picture was primed by a background initially associated with a picture sharing the same emotional valence. The above effect was particularly marked for positive emotional pictures. In addition, this effect varied according to the interval of retention: After short retention intervals (one hour), background pictures were more effective to facilitate the recognition of the specific affective pictures to which they were associated to, whereas after long intervals (two weeks), background pictures facilitated the retrieval of emotional pictures sharing the same emotional valence than the picture initially associated with the background picture. These results demonstrate that a neutral stimulus may acquire an emotional valence as a result of its pairing with an emotional stimulus. They further show that these background pictures can subsequently be used as primes to retrieve the specific emotional information or to retrieve emotionally congruent information, depending on the length of the interval of retention. The latter phenomenon may be seen as an adaptive process to facilitate a forthcoming response for which the emotional valence, but not the specific content, can be anticipated.

# Abstract: EMOTION, ATTENTION, AND EPISODIC MEMORY

**Authors:** A. K. Anderson

**Affiliations:** University of Toronto, Department of Psychology

The influences of emotion on attention and memory are difficult to separate both in nature and in empirical study. For instance, the viewing of a gruesome roadside accident is at once both highly attention-grabbing and memorable. This functional correlation is also supported by the neuroanatomy of emotional influences on attention and memory, which highlight the common central role of the amygdala. That the amygdala is critical to both reflexive attention to and later memory for emotional events suggests the emotional enhancement of memory (EEM) may depend upon the emotional enhancement of attention (EEA) during initial encoding. In a series of studies we show that EEA and EEM are behaviorally and neurally dissociable. We provide evidence demonstrating 1) that the reflexive recruitment of EEA need not result in EEM; 2) the amygdala is involved in both EEA and EEM, but only the former recruits the hippocampal formation, which correlates with EEM and 3) emotional events result in retrograde EEM for prior neutral events, suggesting it is the altered consolidation of rather than attention to emotional events that resulting in long term EEM. In sum, these studies suggest that reflexive attention to and later memory for emotional events depend on dissociable neurocognitive processes, hypothetically reflecting the distinct contributions of the central and basolateral nuclei of the human amygdala.

# Abstract: S5: Neural representation of objects in the inferior temporal cortex

*Organizers*: Pietro Berkes and Yasser Roudi (Gatsby Computational Neuroscience Unit, London, UK)

Inferior temporal (IT) cortex plays a major role in visual object recognition, and thus is crucial for visual processing in the brain. A critical first step in understanding the underlying computational principles in IT is understanding how visual information is coded by neuronal activity. Central questions in current research include the representation of multiple objects, the distributed/single-neuron nature of the code, and the relation between neural activity and perception (are shapes that are perceived as similar represented similarly in IT?). Another key issue concerns invariant representation: what is the degree of invariance of neuronal responses to changes in object attributes and shape, and how is this invariant representation learned?

These questions are difficult to answer because they depend crucially on hard-to-control factors such as previous visual experience and degree of attention. Nevertheless, over the past several years, there has been considerable experimental and theoretical progress in answering them.

The symposium includes contributions by researchers discussing these issues from experimental and theoretical view points and presenting recent progress made in the field.

- **Rufin Vogels** (Laboratorium voor Neuro- en Psychofysiologie, Leuven, Belgium) Representation of perceived shape similarity in macaque inferior temporal cortex
- **James J. DiCarlo** (MIT, Boston, Mass, USA) The role of visual experience in supporting invariant visual object representations in primate Inferior Temporal cortex
- **Edmund T. Rolls** (University of Oxford, UK) Invariant object recognition in the ventral visual system
- **Laurenz Wiskott** (Humboldt University Berlin, Germany) Is slowness a learning principle of the visual system?

# Abstract: REPRESENTATION OF PERCEIVED SHAPE SIMILARITY IN MACAQUE INFERIOR TEMPORAL CORTEX

**Authors:** R. Vogels

**Affiliations:** Laboratorium voor Neuro- en Psychofysiologie

Macaque inferior temporal (IT) cortex is the end station of the ventral visual stream. It is well known that many IT neurons respond selectively to visual shapes. We have conducted a series of studies in which we used artificial shapes that differed systematically along a small number of dimensions and measured the responses of IT neurons of awake monkeys to these parameterized sets of shapes. In particular, we examined the relationship between physical shape similarity, perceived shape similarity (as measured in psychophysical studies in humans and monkeys subjects) and the shape tuning of single macaque IT neurons. I will demonstrate that, firstly, the response of IT neurons to various shapes depends on shape similarity locally in shape space. Secondly, the IT response modulation that is obtained when varying shape correlatesmore strongly with perceived than with physical shape similarity. Thirdly, categorization learning significantly affects the representation of shapes in IT such that the neurons respond more similarly to shapes belonging to the same than to a different learned category. In conclusion, we suggest that the shape representation in IT exceeds a mere representation of physical shape similarity by reflecting perceived shape similarity and emphasizing shape differences that are relevant for categorization.

# Abstract: THE ROLE OF VISUAL EXPERIENCE IN SUPPORTING INVARIANT VISUAL OBJECT REPRESENTATION IN PRIMATE INFERIOR TEMPORAL CORTEX

**Authors:** J. DiCarlo

**Affiliations:** McGovern Institute for Brain Research, MIT

Although object recognition is fundamental to our behavior and seemingly effortless, it is a remarkably challenging computational problem. Our goal is a mechanistic understanding of how the primate brain accomplishes this remarkable feat. Specifically we seek to understand how sensory input is transformed from an initial neuronal population representation (essentially a photograph on the retina), to a new, remarkably powerful form of population representation—one that can directly support object recognition. We are currently focused on patterns of neuronal activity in the highest levels of the ventral visual stream (primate inferior temporal cortex, IT) that may directly underlie recognition. Understanding the creation of the IT representation by transformations carried out along the ventral visual processing stream is the key to understanding visual recognition. In this talk, I will review our results on the spatial and temporal ability of the IT population representation for supporting position- and scale-tolerant recognition. Although several mechanistic hypotheses may explain the remarkable tolerance properties of the IT representation, one of the most intriguing and unexplored is the possibility that visual experience plays an important role in developing such tolerance. I will present results from our ongoing studies aimed at testing this hypothesis using neurophysiology, human psychophysics, and monkey fMRI. These studies illuminate the role of the IT representation in supporting visual object recognition, and provide new constraints on the mechanisms that might produce that representation. Our goal is to use this understanding to inspire artificial vision systems, to aid the development of visual prosthetics, to provide guidance to molecular approaches to repair lost brain function, and to obtain deep insight into how the brain represents sensory information in a way that is highly suited for cognition and action.

# Abstract: INVARIANT OBJECT RECOGNITION IN THE VENTRAL VISUAL SYSTEM

**Authors:** E. T. Rolls

**Affiliations:** University of Oxford, Department of Experimental Psychology

In the primate temporal cortical visual areas, the representation of objects is frequently invariant with respect to position, size and even view. The distributed neuronal representation of object identity uses encoding based on the number of spikes, with little contribution of stimulus-dependent synchrony, and with almost independent information conveyed by different single neurons, so that the encoding capacity of the system is very high. A multistage feed-forward architecture with convergence and competition at each stage is able to learn invariant representations of objects including faces by use of a Hebbian synaptic modification rule which incorporates a short memory trace (0.5 s) of preceding activity. This trace rule enables the network to learn the properties of objects which are spatio-temporally invariant over this time scale. A new learning principle utilises continuous spatial transformations to compute invariant representations. It has been found that in complex natural scenes, the receptive fields of inferior temporal cortex neurons shrink to approximately the size of an object, and are centred on or close to the fovea. It is proposed that this provides a solution to reading the output of the ventral visual system, for primarily the object that is close to the fovea is represented by inferior temporal visual cortex neuronal activity. The effect is captured in models that use competition to weight the representation towards what is at the fovea. Some inferior temporal cortex neurons in these conditions have asymmetric receptive fields about the fovea, so that the location of the face with respect to the fovea, and multiple faces, can be represented in a scene. The model has been extended to account for covert attentional effects such as finding the location of a target object in a complex scene, by incorporating modules to represent the dorsal visual system, backprojections, and short term memory networks in the prefrontal cortex to keep active the representation of the object of attention, and does not require temporal synchronization to implement binding. The model has also been extended to a theory of how invariant global motion such as rotation is computed in the dorsal visual system.

## References

[1] E. T. Rolls LJ (2008). *Memory, Attention, and Decision-Making*.

# Abstract: IS SLOWNESS A LEARNING PRINCIPLE OF THE VISUAL SYSTEM?

**Authors:** L. Wiskott, M. Franzius, P. Berkes, H. Sprekeler

**Affiliations:** Institute for Theoretical Biology, Humboldt University Berlin, Germany

Different representations of our visual environment vary on different time scales. Retinal responses vary quickly because they are very sensitive to saccades or object motion while representations in the inferior temporal cortex (IT) display a large degree of invariance and therefore vary more slowly. Turning this argument around leads to slowness as a learning principle. By learning input-output functions that generate slowly varying output signals, units become invariant to frequently occurring transformations, such as translation, rotation, or illumination changes. We argue that this is an effective mechanism by which IT could learn its invariant representations. Interestingly the same principle also leads to a number of complex-cell receptive-field properties even though invariance does not seem to be such an issue so early in the visual system. Some of the simulation results presented here are complemented by analytical results obtained with variational calculus.

# Abstract: S6: Of rodents, monkeys and men: how experience shapes brain and behavior

*Organizer*: Katharina Braun (Otto von Guericke University Magdeburg, Germany)

*Chair*: Francesca Cirulli (Department of Cell Biology and Neuroscience, Rome, Italy)

Whereas the basic wiring of the mammalian central nervous system is genetically programmed, its fine tuning throughout phases of infancy and childhood is highly dependent on experience. Experience during time windows of elevated synaptic plasticity “imprints” templates of limbic circuits and determines their functional capacity throughout life. Although the impact of early environment on the development of behavior is known for centuries and although our insight into the molecular principles of brain development has increased exponentially in the past decade, these sets of knowledge have not yet been conceptually linked. Understanding the interplay between early experience and the neuron's molecular machinery will guide us to understand that early childhood presents itself as an investment opportunity for our society, and that it takes a well-functioning family and educational system to grow a brain.

- **Fotini Stylianopoulou** (University of Athens, Greece) Effects of earlymanipulations on brain plasticity in rodents
- **Jörg Bock** (Otto von Guericke University Magdeburg, Germany) Impact of pre- and postnatal stress on the establishment of limbic synaptic circuits
- **Steven J. Suomi** (NICHD, Poolesville, Md, USA) How gene-environment (G x E) interactions can shape biological and behavioral development in rhesusmonkeys and other primates
- **Petra Hüppi** (Hôpital des Enfants, Genève, Switzerland) Endocrine disruptors in brain development: effects of adverse prenatal conditions on rat cerebral development: a quantitative proton magnetic resonance spectroscopy (1h-mrs) and histopathology analysis

# Abstract: EFFECTS OF EARLY MANIPULATIONS ON BRAIN PLASTICITY IN RODENTS

**Authors:** F. Stylianopoulou

**Affiliations:** Laboratory of Biology-Biochemistry, University of Athens, Athens, Greece

Early experiences have profound effects on the development of adult behavior. However the underlying neurobiological mechanisms still remain elusive. In an effort to address this issue we employed two experimental paradigms as models of early experiences: A. “neonatal handling”, which is known to affect the programming of the stress response, and B. Spatial learning during the neonatal period using the mother as a positive or negative reinforcer. Neonatal handling affects both emotional and cognitive adult behavior, often in a sexual dimorphic manner. In males it has a beneficial effect, providing them with a greater capacity to actively cope with chronic stressors, while in the females it increases their susceptibility to express “depressive” behavior. A cellular correlate of these behavioral results is a handling-induced change in 5HT1A receptors, with an increase in the males and a decrease in the females. Furthermore, in males handling results in increased ability, as assessed by the Morris water maze, for spatial learning and memory after stress, while it has no such effect in females. Handling results in increased GR and BDNF levels in males, and in the females it increases turnover in the basal forebrain cholinergic system. In the second paradigm in which contact with the mother is used as either a positive or a negative reinforcer, rat pups, both those denied contact (frustrated) and those allowed contact as continuous reward (non-frustrated) learn a T-maze after four days of training (postnatal day 10–13). Although, both experimental groups acquire the spatial memory task, it appears that they employ different cognitive strategies, with non-frustrated rats following procedural learning. On the other hand “frustrated” pups show increased activation of the hippocampus and in adulthood improved recall of spatial learning in the Morris water maze.

# Abstract: IMPACT OF PRE- AND POSTNATAL STRESS ON THE ESTABLISHMENT OF LIMBIC SYNAPTIC CIRCUITS

**Authors:** J. Bock, K. Braun

**Affiliations:** Institute of Biology, Otto von Guericke University Magdeburg, Germany

The way in which experience sculpts the developing neural circuitry is one of the most intriguing questions in developmental neurobiology. Recent evidence suggests that epigenetic factors affect the development of the brain and behaviour in a much more pronounced way than was previously appreciated. However, the cellular mechanisms of such experience-driven developmental events are far from understood. In particular, it is not clear in which way neural changes induced by environmental factors are correlated with certain developmental time windows. Our studies reveal that stressful pre- and postnatal events dramatically influence neuronal development particularly in prefrontal and limbic brain areas. We also found that the direction and magnitude of these dendritic and synaptic alterations is determined by the developmental stage during which an adverse event is encountered. Moreover, the observed experience-induced neuronal changes occur in a highly region- and sex-specific manner. In summary, our results indicate that the synaptic circuits within prefrontal and limbic regions, which play a critical role in emotional regulation, learning and memory formation, are adapted to the animal's pre- and early postnatal environment and therefore may shape the network capacities for species-specific behavioural and cognitive functions in adolescence and adulthood.

# Abstract: S7: The role of the orbitofrontal cortex in adaptive behaviour

*Organizers*: Giorgio Coricelli (Institut des Sciences Cognitives, Bron, France) and Francesco P. Battaglia (Swammerdam Institute for Life Sciences, Universiteit van Amsterdam, The Netherlands)

Cognitive neuroscience is beginning to integrate factors such as preferences, emotion, and social context in the study of decision making, using a range of mutually informative approaches from behavioral experiments, neurophysiology, and functional neuroimaging. The present symposium adopts such a neuroeconomics perspective on decision making, asking how humans and other animals represent values and consequently influence their choices. The orbitofrontal cortex has important reciprocal connections with cortical and subcortical areas of the brain, thus appears to be at the interface of emotion and cognition. Converging neuroscientific evidences show how the orbitofrontal cortex is involved in representing the relative reward values (i.e., preferences), and the affective value of reinforcers, thus playing a fundamental role in complex and adaptive behaviour. Lesions in this brain area determine severe impairments in individual and social decision making. The symposium is aimed to discuss the unique and integrative role of the orbitofrontal cortex in adaptive behaviour, reporting evidence across species based on the use of neurophysiological and neuroimaging methodologies.

- **John O'Doherty** (California Institute of Technology, Calif, USA) fMRI studies on the role of the orbitofrontal cortex in decision making
- **Antoine Bechara** (University of Southern California, Calif, USA) Decision-making after frontal lobe injuries
- **Matthew Roesch** (University of Maryland, Baltimore, Md, USA) The impact of time-discounted reward on neural activity in orbitofrontal cortex and ventral tegmental area
- **Camillo Padoa-Schioppa** (Harvard University, Cambridge, Mass, USA) Economic choice and the Orbitofrontal Cortex

# Abstract: FMRI STUDIES ON THE ROLE OF THE ORBITOFRONTAL CORTEX IN DECISION MAKING

**Authors:** John P. O'Doherty

**Affiliations:** ^1^Computation and Neural Systems Program; ^2^Division of Humanities and Social Sciences, California Institute of Technology, Pasadena, CA 91106

Damage to the orbitofrontal cortex has long been known to lead to impairments in the adaptive control of behavior in order to obtain rewards or avoid punishers. Yet, the underlying functional contribution of OFC that can lead to such an impairment is still poorly understood. A key debate in the literature is whether orbitofrontal cortex is involved exclusively in encoding associations between stimuli and rewarding outcomes (stimulus-outcome learning), or whether this region is also involved in learning the relationship between an individual's own actions and the consequences of those actions (response-outcome learning). The majority of single-unit neurophysiology studies have tended to support the stimulus-outcome view, although some recent studies in rat OFC have found preliminary evidence for responseoutcome coding. Here, I will present results from two human fMRI studies that directly address this issue. I will provide evidence that neural activity in human OFC during instrumental responding for rewards is sensitive to the value of the associated outcome, and to the contingency (or correlation) between an individual's responses and the outcomes received, which together are the key behavioral characteristics of response-outcome learning. These data suggest that human OFC, particularly its medial aspect guides rewardrelated behavior by virtue of encoding associations between a specific action and its associated outcome.

# ABSTRACT DECISION-MAKING AFTER FRONTAL LOBE INJURIES

**Authors:** Antoine Bechara

For a long time, the prefrontal cortex has been considered a “non-functional” brain area, and understanding its function has lagged behind nearly all other areas. This is no longer true since appreciation of the vital role that this brain region plays in adaptive behaviors, and especially decision-making, is now evident more than ever. This presentation is to highlight the recent progress that has been made in this area of research. Decision-making is a term often referred to in the psychological literature as one of the “executive functions”, which play a role in managing (like an executive) other cognitive functions, such as memory, attention, and language. Considerable research efforts have been directed towards differentiating various processes of executive functions, but much of this effort in the past has focused on the dorsolateral prefrontal cortex (DLPC) sector. Here I will review findings that address decision-making and its link to the ventromedial prefrontal cortex (VMPC). Decision-making impairments as encountered in neurological and psychiatric patients are costly in terms of individual human suffering and in financial terms. In addition to its obvious value in advancing fundamental knowledge in neuroscience, understanding the neural mechanisms of decision-making is likely to have important practical consequences, including the understanding and management of neuropsychiatric disorders such as addiction, as well as the management of a considerable number of elderly suffering from a decline in cognitive functions critical for decision-making, in spite of relatively intact memory and general intellect, which impact real-life matters that are important to themselves and their family.

# Abstract: THE IMPACT OF TIME-DISCOUNTED REWARD ON NEURAL ACTIVITY IN ORBITOFRONTAL CORTEX AND VENTRAL TEGMENTAL AREA

**Authors:** M. R. Roesch

**Affiliations:** Departments of Anatomy and Neurobiology, University of Maryland School of Medicine

Animals discount the value of a delayed reward. This behavior presumably requires them to form expectations for rewards and to detect when those expectations are violated. Evidence from recording studies suggests that orbitofrontal cortex (OFC) is responsible for the former function, whereas the latter function is mediated by midbrain dopamine neurons (DA). However few studies have examined the impact of time-discounted reward on these functions. We recorded neurons fromrat VTA and OFC in a choice task during which we manipulated the relative value of the reward by changing the size of or time to reward delivery over the course of several trial blocks. In each block, rats biased behavior towards the more valued reward, i.e., short delays and large rewards. Consistent with the proposal that DA neurons signal prediction errors, we found that reward-related activity of DA neurons in VTA increased significantly whenever the value of the reward available increased and then declined as the rat learned to expect that reward. As neural responses to the rewards waned, responses to odor cues predicting the more valued reward emerged. Signaling of prediction errors in individual neurons was not modulated by response direction and co-varied with delay and reward size manipulations. This pattern of signaling clearly differed from reward-related activity in OFC, which tended to develop as the rats learned to expect reward. This was particularly evident when the timing of the reward changed; delay- selective OFC neurons showed anticipatory and reward-related activity that strengthened during the block as the rats learned when to expect the delayed reward. Additionally encoding of delayed reward in OFC was modulated by response direction and did not covary with encoding of reward size. These data are consistent with the proposal that OFC maintains detailed information about the current value of expected outcomes, whereas VTA signals when those expectations have been violated.

# Abstract: ECONOMIC CHOICE AND THE ORBITOFRONTAL CORTEX

**Authors:** C. Padoa-Schioppa

**Affiliations:** Department of Neurobiology, Harvard Medical School

Economic choice is the behavior observed when individual select one of many available options solely based on subjective preferences. Behavioral evidence suggests that economic choice entails two mental processes: values are first assigned to the available options, and a decision is subsequently made between these values. With respect to the underlying brain mechanisms, numerous reports show that lesions to the orbitofrontal cortex (OFC) lead to choice deficits in various domains, and imaging studies indicate that OFC activates when people make choices. In my talk, I will review evidence from single cell recordings linking OFC more specifically to valuation. Individual neurons in OFC encode the value monkeys assign to different beverages when they choose between them. These neurons encode economic value as a subjective quantity, and they also reflect value transitivity. Most importantly, neurons in OFC encode economic value per se, not as a modulation of sensory or motor processes. This trait distinguishes the value representation in OFC from that observed in other brain areas. That OFC neurons encode economic value independently of visuomotor contingencies has profound implications for possible psychological models of economic choice. It suggests that economic choice is fundamentally choice between goods (“good-based” model) rather than choice between actions (“action-based” model).

# Abstract: S8: Episodic-like memory in animals

*Organizer*: Alexander Easton (University of Durham, Durham, UK)

Episodic memory has long been considered to be a peculiarly human phenomena, with the conscious recall of past events beyond the ability of nonhuman species. However, recent work has shown that nonhuman animals do have a form of memory that resembles episodic memory: episodic-like memory. In the last two years, a number of groups have advanced our knowledge of this area by developing simple but effective tasks that allow us to explore animals recollection of past events including the memory of what happened, where it happened, and when it happened, or in which context it happened. A number of these speakers are brought together in this symposium to discuss recent experimental methods in this field as well as findings from these studies about the neurobiology of episodic memory. For the first time, this brings together work from a number of laboratories whose research has implications not just for studies of memory mechanisms in animals, but also for understanding the mechanisms of memory and memory loss in humans.

- **Ekrem Dere** (Heinrich-Heine-University, Düsseldorf, Germany) NMDA receptors and episodic-like memory in the rodent
- **Mark Good** (Cardiff University, UK) Context-dependent object recognition memory: Assessment of age-related memory impairments in rats and mice
- **Madeline Eacott** (University of Durham, UK) Familiarity and recall in rats: Memory for objects and events
- **Emma Wood** (University of Edinburgh, UK) What, where and which: the role of the hippocampus in context specific episodic-like memory

# Abstract: NMDA RECEPTORS AND EPISODIC-LIKE MEMORY IN THE RODENT

**Authors:** E. Dere

**Affiliations:** Institute of Physiological Psychology, Heinrich-Heine-University Düsseldorf, Düsseldorf, Germany

Rats and mice are attracted by novel objects. Exploration of a novel object leaves a complex memory trace akin to human episodic memory. In rodents, episodic-like memory (ELM) can be inferred from behavior which indicates the remembrance of the content (“what” kind of object was presented), place (“where” was this object placed) and temporal context (“when” was the object presented) after an unique episode. Most importantly, rodents behave as there is an interaction between temporal- and spatial information in their ELM, suggesting that they have established an integrated memory for unique experiences comprising “what”, “where” and “when” information. N-methyl-D-aspartate receptors (NMDA-R) have been implicated in some forms of hippocampal synaptic plasticity and learning and memory. The NMDA-R is well suited to associate multiple features of an event to represent an episode or event. We have asked whether NMDA-R modulation by d-cycloserine (DCS), a glycine-site agonist and cognitive enhancer, has promnestic effects on ELM under sub-optimal learning conditions, induced by either stress or pro/retroactive memory interference. Mild acute stress induced by an i.p. saline injection prior to the learning trials disturbed ELM in rats. DCS (15-mg/kg, i.p.) ameliorated this stress-induced deficit, but was not able to fully restore ELM. Using an experimental protocol designed to detect promnestic drug effects in mice, we found that DCS, can induce ELM under conditions where animals regularly fail to establish an ELM. Mice treated with DCS (20-mg/kg, i.p.) both remembered the temporal order in which two different objects have been encountered, their spatial position, and showed the predicted type of interaction between temporal and spatial information typical for ELM. In conclusion, it appears that the NMDA-R is critically involved in ELM formation in rodents. Supported by DFGgrants DE 1149/1-1 and DE 1149/1-2 to ED.

# Abstract: CONTEXT-DEPENDENT OBJECT RECOGNITION MEMORY: ASSESSMENT OF AGE-RELATED MEMORY IMPAIRMENTS IN RATS AND MICE

**Authors:** Mark A. Good

**Affiliations:** School of Psychology, Cardiff University, Cardiff, UK

The ability to form a representation of the spatial (where) and temporal (when) features of an event is thought to rely upon the medial temporal lobe region. In order to test this hypothesis we have developed a simple object recognition memory procedure in which both rats and mice show a preference for exploring an object that was presented earlier in a sequence and in a novel location relative to other objects that possess only one of these features. Rats with lesions of the hippocampus show a pronounced impairment in this form of object memory while leaving a context-independent form of object memory intact. Here we examine the representational processes that underlie this form of memory and in addition consider whether context-dependent object recognition memory in rodents is sensitive to age-related changes in hippocampal function.

# Abstract: FAMILIARITY AND RECALL IN RATS: MEMORY FOR OBJECTS AND EVENTS

**Authors:** M. Eacott

**Affiliations:** University of Durham

Episodic memory has been claimed to be unique to humans. However, we have been examining rats ability to demonstrate memory for complex events. Based on this evidence we have proposed a novel model of episodic-like memory which involves an integrated, flexible memory for objects (what), their location (where) and on which occasion they were experienced (which).Using this approach, termed what-where-which, we have demonstrated that rats can show recall-like memory for what-where-which that is dependent on the fornix. In contrast, we have shown in the same task that familiarity-based memory is intact, showing for the first time direct behavioural evidence for a dissociation between recall-like and familiarity-based memory in the rat.

# Abstract: WHAT, WHERE AND WHICH: THE ROLE OF THE HIPPOCAMPUS IN CONTEXT SPECIFIC EPISODIC-LIKE MEMORY

**Authors:** E. R. Wood, R. F. Langston

**Affiliations:** Centre for Cognitive and Neural Systems, University of Edinburgh, Edinburgh, UK

It has been proposed that recognition memory tasks can be solved by two different processes: hippocampus-independent familiarity judgements of the prior occurrence of items, and hippocampus-dependent episodic recollection of items together with the contexts in which they occur (Aggleton & Brown, 1999). The recent development of tasks for rats that assess recognition of objects together with contextual information such as where they occurred, when they occurred, and in which context they occurred by the other speakers in this session provide a useful tool for testing this idea. One prediction of the dual process model is the hippocampus should be required for context-dependent, but not for context-independent recognition. To test this, we have used the what, where and which task developed by Eacott and Norman (2004), which requires integrated memory for objects, their spatial locations, and their background contexts, as well as a variety of control tasks. Large, bilateral hippocampal lesions result in impaired performance on the OPC task, while leaving object recognition, object-place (OP) recognition and object-context (OC) recognition intact. These data suggest that the hippocampus is not required for all forms of context-dependent recognition memory, and that the OPC task may depend on hippocampusdependent processes that are not required for recognition of object-place and object-context configurations. Therefore, the underlying processes, rather than the information content per se, may determine whether the hippocampus is required for recognition memory. I will then go on to describe the effects of discrete lesions of the CA1 and CA3 subfields of the hippocampus on performance of the these tasks. Our data suggest that the CA1 is required for memory for integrated object-place-context information, whereas the CA3 region may play a less critical role. These data will be discussed in the light of current theories of hippocampal contributions to episodic memory.

# Abstract: S9: Attention-deficit/hyperactivity disorder (ADHD) in childhood: the Behaviour and the Neurobiology

*Organizers*: Terje Sagvolden (University of Oslo, Norway) and Bob Oades (University Clinic of Child and Adolescent Psychiatry, Essen, Germany)

Recently, interdisciplinary investigations involving collaboration between clinical research and the basic neurosciences have thrown new light on the widespread developmental dysfunctions grouped together as childhood attentiondeficit/ hyperactivity disorder (ADHD). The high heritability of the disorder has been related to a number of features defining monoaminergic function. In turn, the success of catecholaminergic medications and animal models of various symptoms (e.g., in the spontaneously hypertensive rat) point not only to the neurochemical bases underlying deficient attentional control and impulsivity in “hyperactive children,” but also crucially to their altered responsiveness to reinforcement (e.g., delay aversion). In parallel, indices of monoaminergic function have been related in neuroimaging studies to the activation (or lack thereof) in patients' brain regions (e.g., the frontal lobes) involved in important executive and reward functions. The addition of neurophysiological measures to such investigations has helped illustrate the nature of the delayed developmental processes of potential etiological importance. Illustrating the interactions beween these levels of analysis, an explanation of the underachievement of children with ADHD in terms of their high intraindividual variability of response organization will be proposed; this is a theory based on neuron-glia interactions. But we emphasize that recent and ongoing studies of these interdisciplinary interactions will be illustrated in each of the four presentations.

- **Heidi Aase** (Norwegian Institute of Public Health, Oslo, Norway) From model to practice—experimental studies of basic characteristics of the behaviour of animal models and of children with AD/HD
- **Terje Sagvolden** (University of Oslo, Norway) Animal models of ADHD—aspects of their behavior, genetics and neurobiology
- **Katya Rubia** (King's College London, UK) Functional imaging abnormalities in Attention Deficit Hyperactivity Disorder and effects of Methylphenidate
- **Bob Oades** (University Clinic, Essen, Germany) Glia energy supply: a neuro-physiological explanation for intra-individual variability and maturation delays in ADHD

# Abstract: FROM MODEL TO PRACTICE EXPERIMENTAL STUDIES OF BASIC CHARACTERISTICS OF THE BEHAVIOUR OF ANIMAL MODELS AND OF CHILDREN WITH AD/HD

**Authors:** Aase Heidi, Sagvolden Terje

**Affiliations:** ^1^Norwegian Institute of Public Health, Division of mental health, Oslo, Norway; ^2^University of Oslo, Department of Physiology, Norway

The behaviour of children with Attention-Deficit / Hyperactivity Disorder (AD/HD) is characterized by low predictability of responding. Low behavioural predictability is one way of operationalizing intra-individual AD/HD-related variability. AD/HD-related variability may be caused by inefficient behavioural selection mechanisms linked to altered reinforcement and extinction processes, as suggested by the recently published dynamic developmental theory (DDT) of AD/HD. DDT argues that AD/HD is a basic neurobehavioural disorder, probably caused by dysfunctioning dopamine systems. For establishing AD/HD as a neurobehavioural disorder, findings from studies conducted in Western countries should be replicated in other cultural populations. Also, predictions about basic behavioural mechanisms should be shown in animal models for the disorder.

The present paper will discuss validation criteria for animal models and show data from a translational and crosscultural research programme aiming to bridge the gap between neurobiology and behaviour. It is shown that similar behavioural characteristics may be found across cultures and across species, supporting the conclusion that AD/HD is a neurobiologically based disorder. Further, the data indicate that some of the intra-individual variability may be related to altered learning mechanisms in AD/HD.

# Abstract: ANIMAL MODELS OF ADHD ASPECTS OF THEIR BEHAVIOR, GENETICS AND NEUROBIOLOGY

**Authors:** Terje Sagvolden, Steve Faraone, Frank Middleton, Tania Dastc

**Affiliations:** ^1^University of Oslo; ^2^SUNY Upstate Medical University, NY

**Background**. An ideal animal model should be similar to the disorder it models in terms of etiology, biochemistry, symptomatology, and treatment. Animal models provide several advantages over clinical research: simpler nervous systems, easily interpreted behaviors, genetic homogeneity, easily controlled environment, and a greater variety of interventions. Attention-deficit/hyperactivity disorder (ADHD) is a neurobehavioral disorder of childhood onset that is characterized by inattentiveness, hyperactivity, and impulsiveness. Its diagnosis is behaviorally based; therefore, the validation of an ADHD model must be based in behavior. An ADHD model must mimic the fundamental behavioral characteristics of ADHD (face validity), conform to a theoretical rationale for ADHD (construct validity), and predict aspects of ADHD behavior, genetics, and neurobiology previously uncharted in clinical settings (predictive validity). Spontaneously hypertensive rats (SHR) fulfill many of the validation criteria and compare well with clinical cases of ADHD.

**Methods**. ADHD-like behavior was tested with a visual discrimination task measuring overactivity, impulsiveness and inattentiveness. Gene expression was measured by extracting mRNA from the tissue and hybridising the mRNA sample to the 25-mer probes on Affymetrix GeneChips.

**Results**. The SHR showed striking overactivity and poorer sustained attention. WKY and Sprague-Dawley rats showed a normal behavior. Wistar rats deviated significantly from the other control groups. Genetic analyses showed several changes in monoamines as well as other systems. Discussion. The present results indicate that overactivity and impulsiveness are caused by imbalances in other neuromodulators than those causing poor sustained attention. Behavioral phenotyping of controls is essential in animal model studies of ADHD. WKY, but not Sprague-Dawley and Wistar rats, are appropriate controls.

# Abstract: FUNCTIONAL IMAGING ABNORMALITIES IN ATTENTION DEFICIT HYPERACTIVITY DISORDER AND EFFECTS OF METHYLPHENIDATE

**Authors:** K. Rubia, A. B. Smith, R. Halari

**Affiliations:** Department of Child Psychiatry, Institute of Psychiatry, King's College London, UK

This paper will review the current evidence for functional magnetic resonance imaging (fMRI) abnormalities in children and adolescents with Attention Deficit Hyperactivity Disorder (ADHD) and show the effects of stimulant medication on neural networks of cognitive functioning. The most consistent fMRI findings in ADHD have been a reduced activation in predominantly right frontal and caudate brain regions during tasks of inhibitory and cognitive control. Preliminary evidence from our cross-sectional functional imaging studies suggests that ADHD might be characterised by a maturational delay of normal task-specific neurofunctional activation. Recent evidence from our and other labs has in addition suggested more widespread functional abnormalities in ADHD in parietal and temporal brain regions during visual-spatial attention functions. We will review these recent findings and show evidence that temporal lobe abnormalities during attention allocation in ADHD is related to their typical behavioural abnormality of increased intra-subject response variability. Lastly, the effects of the psychostimulant drug Methylphenidate will be shown on brain activation in medication-nave ADHD children during several tasks of cognitive control. During all tasks, Methylphenidate appears to enhance taskunspecific, generic striatal and cingulate neural networks that mediate attention and motivation rather than normalising task-specific prefrontal brain abnormalities. Overall, the findings suggest that ADHD is associated with a more immature recruitment not only of fronto-striatal brain regions during cognitive control, but also of parieto-temporal brain regions in relation to typical behavioural features such as inattention and behavioural dispersion. Furthermore, Methylphenidate does not normalise task-specific prefrontal abnormalities, but seems to modulate cognitive functions by enhancing compensatory, generic and task-unspecific subcortical attention networks.

# Abstract: GLIA ENERGY SUPPLY: A NEURO-PHYSIOLOGICAL EXPLANATION FOR INTRA-INDIVIDUAL VARIABILITY AND MATURATION DELAYS IN ADHD

**Authors:** R. D. Oades

**Affiliations:** Biopsychology Group, University Clinic for Child and Adolescent Psychiatry, Essen, Germany

Descriptions of potential endophenotypes for attentiondeficit/hyperactivity disorder (ADHD) have referred to right hemisphere function and impulsivity among others. Here it is proposed that intra-individual behavioural variability has broad explanatory value. Symptoms are expressed often or frequently or pretty much and performance on laboratory tests varies markedly over brief time periods (milliseconds to seconds).

A new explanation of the phenomenon suggests there is a deficiency of the energy supply to rapidly firing cortical neurons1. Central to the mechanism may be the glutamate-stimulated lactate shuttle under astrocytic control and modulated by monoaminergic input. In situations requiring continual responses to externally paced stimuli the rapid firing of neurons must be maintained: but in ADHD, and in related conditions, there may be deficient ATP production, a slow restoration of ionic gradients across neuronal membranes and delayed neuronal firing often resulting in the subject going off-task. Further, the proposed insuffient glial supply of lactate may also affect CNS development on the time scale of months to years. This deficiency in the oligodendrocytes may lead to impaired fatty acid synthesis and an uneven myelination of axons as the children grow up. Reduced glycolysis and lactate supply can reflect under-stimulation of catecholamine receptors and be moderated by current pharmacotheraputic strategies.

The hypothesis can be tested with neuroimaging (DTI), EEG recording (coherence), biochemical indicators (compromised glial function) and animal models. The prevalence of genetic markers for factors that regulate energy metabolism, astrocyte function and myelin synthesis should be investigated. If confirmed the relevant mechanisms offer a range of new targets for genetic moderation and therapeutic intervention.

## References

[1] Russell VA, Oades RP, Tannock R (2006). Response variability in attention-deficit/hyperactivity disorder: a neuronal and glial energetics hypothesis. *Behavioral and Brain Functions*.

# Abstract: S10: Hippocampal/cortical interactions in spatial behaviors

*Organizer*: Etienne Save (CNRS, Université de Provence, Marseille, France)

Spatial behaviors in animals depend on a distributed brain network. The function of the hippocampus in this network is crucial but requires interactions with a number of cortical areas. A recent discovery have revealed the existence of “grid cells” (cells with firing fields forming a grid structure) in the entorhinal cortex which constitute the major cortical input to the hippocampus. Marianne Fyhn will present an overview of the grid cells studies and Torkel Hafting some new data about the interactions between the entorhinal grid cells and the hippocampal place cells. The existence of a specific connection between the hippocampus and the prefrontal cortex suggests a functional interaction between the two structures. A recent study have suggested that the prefrontal cortex contributes to goal encoding (Etienne Save). The forth presentation will consider the interaction between the hippocampus and the cortex in the perspective of the dynamics of spatial memory processes (Bruno Bontempi).

- **Marianne Fyhn** (Centre for the Biology of Memory, NTNU, Trondheim, Norway) Spatial representation in the entorhinal cortex
- **Torkel Hafting** (Centre for the Biology of Memory, NTNU, Trondheim, Norway) Interactions between entorhinal grid cells and hippocampal place cells
- **Etienne Save** (CNRS, Université de Provence, Marseille, France) Goal encoding in prefrontal cells and hippocampal place cells
- **Bruno Bontempi** (CNRS, Université Bordeaux 1, Talence, France) Hippocampal-cortical interaction during spatial memory processing

# Abstract: SPATIAL REPRESENTATION IN THE ENTORHINAL CORTEX

**Authors:** M. H. Fyhn

**Affiliations:** Centre for the Biology of Memory, Norwegian University of Science and Technology, Norway

The ability to find ones way is dependent on the integration of information about position, direction, and distance. This is likely to rely on a distributed brain network where the medial entorhinal cortex (MEC) plays a central role. By recording single units from MEC in behaving rats we have revealed a topographically organized neural map of the spatial environment in MEC. The key unit of the map is the ‘grid cell’ which fires selectively at multiple spatial locations forming a triangular grid pattern spanning the surface of the environment. The map is anchored to external landmarks, but persists in their absence, suggesting the grid cells may be part of a generalized, path-integration based map of the spatial environment. Grids of neighbouring cells share a common orientation and spacing, but their vertex locations are offset relative to each other. Grid cells show a topographic organization with grid spacing increasing from less than 30 cm dorsally to several meters ventrally. Grid cells are found in all four principle cell layers of MEC and they intermingle with head-direction cells in all layers except layer II. ‘Conjunctive cells’ with conjoint head- direction and grid cell properties exist in layers III to VI. Simultaneously recorded grid-, conjunctive- and head-direction cells respond coherently to environmental manipulations, such as a rotation of the enclosure. Grid cells show a preserved temporal and spatial firing relation to each other across environments. Simultaneously recorded grid cells at different locations of MEC shift, rotate and expand in concert between environments even when grid spacing and grid orientation are different. Our results imply that MEC operates as an integrated structure for accurate positional calculations. The fact that realignments were similar between recording locations suggests that the entorhinal map may show a coherent representation of space that extend across areas of different grid scales and grid orientations.

# Abstract: INTERACTIONS BETWEEN ENTORHINAL GRID CELLS AND HIPPOCAMPAL PLACE CELLS

**Authors:** T. F. Hafting

**Affiliations:** Centre for the Biology of Memory, Norwegian University of Science and Technology, Norway

In the medial entorhinal cortex (MEC) neurons with positional, directional, and metric information constitutes a context- independent spatial map of the environment. The hippocampal place code is context specific and responds to changes in the sensory or motivational input as either a full reorganization of the place code (global remapping), or as a selective change of firing rates (rate remapping). To determine whether the hippocampal population response originates within the hippocampus or upstream in the grid-cell network of the MEC, we recorded spike activity in these areas under conditions that gave either global or rate remapping in the hippocampus. Global remapping in CA3 was always accompanied by a coherent offset in the grid of simultaneously recorded MEC neurons. The strong temporal contiguity of hippocampal remapping and entorhinal grid shifts suggests that the two phenomena are mechanistically related. During rate remapping in CA3, grid cells in MEC showed no offset or rotation, and the firing rate distribution remained unaltered. Thus, it is likely that rate remapping is of intrahippocampal origin. The entorhinal cortex and hippocampus have reciprocal connections suggesting an integrated unit with bidirectional influence. It has been suggested that stored representations in the hippocampus anchor the grid to the environment. Temporal inactivation of the hippocampus by local infusion of muscimol dramatically reduced the firing frequency of grid cells, and the grid patterns almost vanished from the time-averaged rate maps, while simultaneously recorded head-direction cells from layer III were not affected. These results suggest that entorhinal grid fields are dependent on functional interactions with the hippocampus. It remains to be elucidated, however, whether the hippocampal output exerts a general excitatory drive on the grid cells or if grid stability depends on stored representations in the hippocampus.

# Abstract: GOAL ENCODING IN PREFRONTAL CELLS AND HIPPOCAMPAL PLACE CELLS

**Authors:** E. Save, V. Hok, P.-P. Lenck-Santini, B. Poucet

**Affiliations:** ^1^Lab Neurobiology and Cognition, CNRS, Aix-Marseille Universités, Marseille, France; ^2^Neuroscience Center, Dartmouth Medical School, Lebanon, USA

Spatial navigation depends on a distributed brain network of cortical and subcortical structures. The hippocampus is thought to play a key role in this network because it contains place cells. Although place cells signal an animal's location, an additional mechanism is needed to specify the animal's goal. However, whether and how the brain encodes goal location have not been demonstrated so far. The prefrontal cortex is a potential candidate to such a function since it has been proposed to be involved in the organization of spatial behaviour and it receives a direct projection from the hippocampus. To investigate this hypothesis, we recorded medial prefrontal cortex neurons from rats performing a continuous place navigation task. In this task, rats had to locate an unmarked goal zone by using an environmental cue and wait there for 2 s to trigger an overhead feeder. A single pellet was then released and could end anywhere in the arena after dropping. The rat had to leave the goal zone and forage to retrieve the pellet. We found that a substantial proportion of neurons in the prelimbic and infralimbic areas had firing fields. Prefrontal firing fields were larger and noisier than place cell firing fields and were concentrated in explicit goal locations (goal zone, pellet-dropping zone). We also recorded hippocampal place cells as rats performed the same task. We discovered that in addition to their main firing field, place cells displayed a selective discharge while the rat was at the goal zone waiting for pellet release. Because it was unlikely that such activity resulted from a specific behavioral state, we propose that this is a signal of the animal's correct location at the goal. Overall, the results suggest that the prefrontal cortex and the hippocampus are involved and cooperate in goal encoding. Support by CNRS, MENRT (ACI Neurosciences Intégratives et Computationnelles).

# Abstract: HIPPOCAMPAL-CORTICAL INTERACTIONS DURING SPATIAL MEMORY PROCESSING

**Authors:** B. Bontempi

**Affiliations:** Centre de Neurosciences Intégratives et Cognitives, CNRS UMR 5228, Université Bordeaux 1, Talence, France

It is now well established that the formation of declarative memories involves changes in synaptic plasticity within structures of the medial temporal lobe, including the hippocampus. However, our group and others have demonstrated previously that the hippocampal formation has a time-limited role in long-term memory storage, such that extrahippocampal structures eventually become capable of independently supporting the retrieval of remote memories. In other words, the hippocampus does not store remote memories, yet what happens beyond the hippocampus remains unclear. This issue has been the subject of intense investigation and debate in the field of Cognitive Neuroscience, but to date, no convincing evidence as to the identity, mechanisms and putative interactions between memory systems underlying remote memory storage and retrieval have emerged. To address this issue, we have conducted brain imaging experiments using (14C)2-deoxyglucose mapping and analyses of changes in expression of activity-dependent genes in mice submitted to recent and remote spatial memory testing. Our findings show that memory processing initially requires a hippocampal-cortical dialogue, ultimately enabling structured cortical networks to mediate recall of remote memories. Morphological changes, including the synthesis of novel synapses in cortical networks also occurs as memories progressively mature. However, the neocortex does not simply serve as a passive storage site but is also able to actively integrate new memories depending on the organisation and status of pre-existing knowledge. The prefrontal cortex in particular may play a crucial role in integrating information from distributed cortical networks and modulating hippocampal activity during memory recall. These findings will be discussed in the context of current models of memory consolidation.

# Abstract: S11: New insights in the development of cortical netwarks

*Organizers*: Alessandro Vercelli (Pharmacology and Forensic University of Turin, Turin, Italy) and Ferdinando Rossi (University of Turin, Turin, Italy)

The construction of neural circuits in cortical structures involves complex developmental processes, including sequential generation of different neuron categories, their homing in precise cortical layers, and the formation of highly patterned local and distant connections. All these features emerge from largely unknown interactions between genetically determined mechanisms and influences from the external world. The symposium is devoted at discussing new insights in some specific aspects of cortical development, including gene expression patterns that define specific neuron phenotypes and populations, cellular interactions that determine the morpho-functional identity of cortical areas or nodules, integration of neurons in cortical layers and circuits, wiring of long-distance projections, and local inhibitory connections. In particular, we aim at bringing together and comparing complementary experiences drawn from the study of the ontogenesis of major cortical structures in the mammalian brain, the cerebral and cerebellar cortex.

- **Zoltan Molnar** (University of Oxford, UK) Towards the classification of projection neurons in layer V and subplate
- **Alessandro Vercelli** (University of Turin, Italy) Development and plasticity of cortical pyramidal neurons
- **Marion Wassef** (CNRS/ENS, Paris, France) Cell interactions involved in the development of functional modules in the cerebellar cortex
- **Ferdinando Rossi** (University of Turin, Italy) Specification and integration of inhibitory interneurons in the cerebellar cortex

# Abstract: TOWARDS THE CLASSIFICATION OF PROJECTION NEURONS

**Authors:** Amanda Cheung, Zoltán Molnár

**Affiliations:** Department of Human Anatomy and Genetics, University of Oxford, South Parks Road, Oxford, OX1 3QX

Pyramidal neurons in layer V of rodent cerebral cortex are of two main types (Larkman and Mason, 1990). One type has a thick apical dendrite extending into layer I with a prominent terminal tuft, produces distinctive initial bursts of action potentials in response to current injection and projects out of the telencephalon to the superior colliculus, pontine nuclei and spinal cord. The other type has a slender apical dendrite which does not reach layer I, arborises in layers II-IV without a terminal tuft, does not burst, and projects to other cortical areas, including the contralateral hemisphere. These neurons are generated at similar times, and with the exception of their projections, are initially indistinguishable, having tufted apical dendrites reaching layer I and no bursting firing pattern. Towards the end of the first postnatal week the corticocortical cells selectively lose their apical tufts (Koester and O'Leary, 1992) and it is not until 14 days postnatally that the first corticotectal cells develop the burst-firing characteristic (Kasper et al., 1994; Christophe et al., 2005). Our experiments aimed at detecting and characterizing causal relationships between target finding, somatodendritic differentiation, physiological specification and gene expression patterns using the different layer V projection neurons in the rodent as a model system.

Previous and recent studies have isolated molecules which serve as molecular markers for layer V neurons; Otx- 1, a transcription factor expressed in layers V/VI, specific to type I (Frantz et al., 1994; Weimann et al., 1999); SMI-32, N200 and FNP-7 which are neurofilaments only expressed in type I neurons (Voelker et al., 2004); ER81, an ETS transcription factor expressed in both neuronal cells types (Hevner et al., 2003; Yoneshima et al., 2005); Lmo4, a LIM domain-containing protein known to be expressed in layers II/III and V (Bulchand et al., 2003) which is a specific marker for type II neurons (Arlotta et al., 2005); CTIP2, a gene of unknown function specifically expressed in layer V type I neurons (Arlotta et al., 2005).

Co-localisation studies onOtx-1 and ER81 suggested that the two markers are not expressed within the same postnatal layer V neurons; moreover ER81 is also expressed in some type II layer V pyramidal neurons (Hevner et al., 2003; Yoneshima et al., 2005). Although ER81 and N200 have been shown to label type I projection neurons in layer V, recent co-localisation studies suggests that the two molecules are not co-expressed within the same cells (Rolph et al. 2005). Retrograde labelling and immunohistochemistry for the two markers revealed that although ER81 and N200 both expressed in type I layer V neurons, they are never coexpressed in the same projection neurons, suggesting that there are at least two distinct neurochemical subpopulations within type I layer V pyramidal cells (Rolph, et al., 2005; A. Cheung, C. Voelker, R. Rolph, T. Jessell and Z. Molnar unpublished observations). An important aim of these studies is to be able to unify molecular classification with other aspects of layer V neuronal classification in adult and during development. Potential molecular markers for layer V neurons are continually being found and the correlation of these markers to other aspects of neuronal phenotype will not only result in a more comprehensive classification of layer V projection neurons, but also in the better understanding of the genetic and epigenetic programs of cortical neuronal differentiation and cortical circuit formation.

# Abstract: DEVELOPMENT AND PLASTICITY OF CORTICAL PYRAMIDAL NEURONS

**Authors:** A. Vercelli

**Affiliations:** Department of Anatomy, Pharmacology and Forensic Medicine, University of Torino, Italy

Pyramidal neurons represent the 70% of cortical neurons, and are different from each other for laminar position, pattern of projection, morphological and electrophysiological features. Their development depends on genetic and epigenetic factors, and developmental errors can lead to diseases such as epilepsy and mental retardation. A subpopulation of pyramidal neurons lose their apical dendrite at the end of development and gives origin to spiny stellate neurons. Another one is characterized by a shortened apical dendrite, whereas others by a long apical dendrite reaching the first cortical layer. Rho-GTPases are involved in pyramidal neuron development. In particular, Citron K is involved in cytokinesis and in the development of dendritic arborisation, whereas Citron N is involved in spine formation. Genetic manipulation of their genes can lead to gross alterations in pyramidal neurons and their dendrites. Pyramidal neurons are dependent on their laminar position and on local factors in their dendritic arborisation, and pyramidal neurons projecting to different targets differ for morphology and electrophysiology. They participate in the vertical organisation of cerebral cortex in modules, both in cortical columns and minicolumns. Minicolums are conserved through species, individuals and cortical areas, but their composition is multifarious. They are characterized by groups of pyramidal neurons located in different cortical layers whose apical dendrites bundle together: they possibly reflect aspects of neuronal neurogenesis, and are elctrophysiologically linked by gap junctions in the early phases of cortical development. Pyramidal neurons in the same dendritic bundle send axons which fascicle together, and project to the same target, either cortical or subcortical. The organisation of minicolumns therefore is targetrelated. It is disrupted in several diseases such as autism and Alzheimer disease. Support: Compagnia di San Paolo.

# Abstract: CELL INTERACTIONS INVOLVED IN THE DEVELOPMENT OF FUNCTIONAL MODULES IN THE CEREBELLAR CORTEX

**Authors:** P. C. Grimaldi, A. Coiffic, A. Belmer, F. Rossi, M. Wassef

**Affiliations:** ^1^Regionalisation nerveuse, CNRS/ENS UMR8542 Paris; ^2^Department of Neuroscience, University of Torino

During development, the GABAergic interneurons (GI) of the cerebellar and cerebral cortices derive from populations of proliferative subventricular progenitors that also give rise to oligodendrocytes. Because the bHLH transcription factor Mash1 is involved in the differentiation of the GI and oligodendrocytes destined to populate the cerebral cortex, we investigated its function in GI differentiation in the cerebellum. By comparing the generation of parvalbumin labeled GI in heterochronic transplants of Mash1−/− and wild type embryonic cerebellum, we found that the GI/Purkinje cell ratio is sharply decreased in the mature cerebellar cortex in the absence of Mash1 function. Accordingly the expression of Pax2, a marker of immature GI, is decreased in Mash1 mutant E18.5 embryos compared to wild type littermates. The level of Mash1 expression differs between the GI and oligodendrocyte lineages. By comparing wild type, Mash1−/− and Mash1 electroporated embryos, we are currently investigating how the level of Mash1 expression controls delamination of subventricular progenitors from the cerebellar neuroepithelium and the fate choice between oligodendrocytes and GI.

It has been shown recently that inhibition is organized in parasagittal zones in the molecular layer of the cerebellar cortex. Our observations indicate that GI distribution in the molecular layer of the cerebellum depends on the function of Rig1/Robo3. Variations in inhibition could result from a modulation of the activity of the Slit/Robo/Rig pathway resulting in an uneven distribution or a regional modulation of the functional properties of the GI.

# Abstract: SPECIFICATION AND INTEGRATION OF INHIBITORY INTERNEURONS IN THE CEREBELLAR CORTEX

**Authors:** F. Rossi

**Affiliations:** Department of Neuroscience, University of Turin, I-10126 Turin, Italy

The different neuronal phenotypes that populate the cerebellar cortex are generated according to a precise spatiotemporal schedule, in which projection neurons precede local interneurons. Glutamatergic neurons develop from the rhombic lip, whereas GABAergic types originate from the ventricular neuroepithelium. Progenitors in these germinal layers are committed towards specific phenotypes already at early ontogenetic stages. Transplantation experiments show that postnatally proliferating precursors exposed to the heterochronic environment of the embryonic cerebellar primordium are unable to adopt the identities of projection neurons (Purkinje cells or deep nuclei neurons), suggesting that the sequence of phenotype generation results from the progressive restriction of progenitor cell developmental potential. GABAergic interneurons derive from a subset of ventricular zone cells, which migrate in the white matter and proliferate up to postnatal life. During this period, different interneuron categories are produced according to an insideout sequence, from the deep nuclei to the molecular layer. Progenitors for these interneurons heterochronically transplanted to embryonic or postnatal cerebella achieve a high degree of integration in the recipient cortex and deep nuclei, and acquire GABAergic interneuron phenotypes appropriate for the host age and engraftment site. Therefore, contrary to other cerebellar types, which derive from fate-restricted precursors, GABAergic interneurons are produced by a common pool of progenitors, which maintain their full developmental potentialities up to late ontogenetic stages and adopt mature identities in response to local instructive cues. In this way, the numbers and types of inhibitory interneurons can be set by spatio-temporally patterned signals in order to match the functional requirements of developing cerebellar circuits.

# Abstract: S12: GABA, the Amygdala, and Mood and anxiety disoredrs

*Organizers*: Gal Richter-Levin (University Haifa, Haifa, Israel) and Oliver Stork (Sch Med, Otto-Von-Guericke University, Magdeburg, Germany)

GABAergic neuromodulation in the brain is usually associated with regulating the level of excitability of the principal excitatory networks of activity that are considered to process information within the brain and to form memories. Abnormal GABAergic neuromodulation is typically associated with epileptic activity and epilepsy whereas affective disorders are traditionally linked to abnormal modulation by monoamines.

However, accumulating data suggest a role for abnormal GABAergic neuromodulation in affective disorders. Furthermore, possible interactions between GABAergic agents and monoamines have recently been suggested.

The proposed symposium brings together four researchers whose complementary research, ranging from more basic neuroscience to biological psychiatry research, is aimed at elucidating evidence supporting a role of GABAergic neuromodulation in brain malfunction associated with affective and anxiety disorders.

- **Maria F. M. Braga** (Uniformed Services University of the Health Sciences, Bethesda, Md, USA) Stress-induced alterations in GABAergic transmission in the amygdala: Implications for the etiology and treatment of depression
- **Graziano Pinna** (University Illinois, Chicago, Ill, USA) Fluoxetine (FLX) and norfluoxetine (NFLX) regulate contextual fear responses and aggression via activation of neurosteroidogenesis
- **Oliver Stork** (Otto-von-Guericke University, Magdeburg, Germany) Molecular view on GABAergic function in the amygdala—relevance to anxiety and mood disorders
- **Gal Richter-Levin** (University of Haifa, Israel) Alteration of amygdalar GABAA Receptor? Subunit maturation in a rat model of mood and anxiety disorders

# Abstract: FLUOXETINE (FLX) AND NORFLUOXETINE (NFLX) REGULATE CONTEXTUAL FEAR RESPONSES AND AGGRESSION VIA ACTIVATION OF NEUROSTEROIDOGENESIS

**Authors:** Graziano Pinna

**Affiliations:** Psychiatric Inst., Univ. Illi Chic, Chicago, IL

Social isolation (SI) of male mice lasting longer than three weeks enhances the intensity of aggressiveness and the magnitude of contextual fear. These changes are associated with the downregulation ($\tilde{5}0\%$ ) of 5*α*-reductase type I (5*α*-RI) protein and mRNA expression and a reduction ($\tilde{5}0\%$) of allopregnanolone (Allo) levels in the olfactory bulb, frontal cortex, and limbic structures. Here, we investigated whether pharmacological manipulation (up-or downregulation) of brain Allo content changes in the opposite direction in association with fear conditioning and aggression. To increase Allo brain levels, we administered the S-isomers of the selective brain steroidogenic stimulants (SBSS) FLX and NFLX, which are several-fold more potent than their respective Risomers in upregulating Allo brain levels (PNAS 101: 6222, 2004). The EC50 of S-FLX (0.80 *μ*mol/kg s.c.) and S-NFLX (0.15 *μ*mol/kg s.c.) required to normalize brain Allo content in SI mice is respectively 10–50 times lower than the EC50 required for inhibit serotonin reuptake (PNAS 101: 6222, 2004). At concentrations that failed to inhibit serotonin reuptake but increased neurosteroid biosynthesis, S-FLX (0.5 to 1.9 *μ*mol/kg s.c.) and S-NFLX (0.09 to 0.9 *μ*mol/kg s.c.) administered to SI mice reduced by 30–40% the duration of contextual fear to an electro-shock stimulus (3 shocks at 2 mA delivered 24 hrs before the test) and the intensity of aggressiveness. Hence, these effects are related to neurosteroid biosynthesis and not to serotonin reuptake. Further, in group-housed mice, we depleted Allo levels by 60 to 80% in the hippocampus, amygdala, olfactory bulb, and frontal cortex by administering 10 to 80 *μ*mol/kg s.c. of the potent selective 5.-RI inhibitor (17beta)-17-[bis (1-methylethyl) amino carbonyl)] androsta-3,5diene-3-carboxylic acid (SKF 105,111). At these doses, this drug elicits an increased contextual fear conditioning intensity similar to that observed in SI mice. These pharmacological manipulations of neurosteroid biosynthesis may allow the identification of inhibitory synaptic circuits directly linked to fear and aggression.

# Abstract: MOLECULAR VIEW ON GABAERGIC FUNCTION IN THE AMYGDALA—RELEVANCE TO ANXIETY AND MOOD DISORDERS

**Authors:** O. Stork

**Affiliations:** Department of Molecular Neurobiology, Institute for Biology, and Institute for Physiology, Otto-von-Guericke University, Magdeburg

Local interneurons that use gamma amino butyric acid (GABA) as a transmitter play a critical role in information processing in the amygdala and the control of affective behaviour. These neurons comprise a highly heterogeneous population of cells further characterised by the expression of particular neuropeptide co-transmitters and specific neuromodulatory innvervation. Although together accounting only for 10–20% of the total neuron population in the amygdala, GABAergic interneurons exert a profound control over the activity of amygdala projection neurons, information flow within the amygdala and its integration in extended network activity patterns. We employed a combination of molecular, behavioural and system physiology approaches to identify gene products that are involved in control of such emotions through the GABAergic interneurons in the amygdala, and to characterise their contribution to cellular and network processes underlying affective behaviours. In particular, we investigated the role of the key enzyme in GABA synthesis, glutamic acid decarboxylase (GAD), as well as neuropeptide co-transmitter in GABAergic neurons, such as somatostatin, in these processes. Firstly, gene expression analysis indicated a involvement of these factors in specific aspects of fear memory consolidation. Secondly, mutant mice with deficits in different GAD isozymes (GAD65 and GAD67) or somatostatin revealed their differential involvement in the postnatal development of the GABAergic system in the amygdala, as well as the expression of anxiety-like and mood-related behaviours. And thirdly, in further gene expression and cell culture studies we identified a novel antidepressant-sensitive signalling pathway that is likely to control gene expression in GABAergic interneurons of the amygdala.

# Abstract: ALTERATION OF AMYGDALAR GABAA RECEPTOR *α*; SUBUNIT MATURATION IN A RAT MODEL OF MOOD AND ANXIETY DISORDERS

**Authors:** G. Richter-Levin, S. Jacobson-Pick, K. Rosenblum

**Affiliations:** University of Haifa; University of Haifa; University of Haifa

Profound evidence indicates that GABAARs are important in the control of the physiological response to stress and anxiety. The GABAAR are best distinguished by their type of.; subunit. The alpha 2, 3, 5 subunits are predominantly expressed in the brain during the embryonic and early postnatal period, whilst alpha1 are most prominent during later developmental stages. We have shown before that Juvenile stress impairs coping behavior in adulthood. The present study examined the short and long-term effects of Juvenile stress on GABAA subunit expression in both the amygdala and hippocampus. At juvenileness (d 26-28), either elevated platform stress or variable stress were applied. The open field, startle response test, and the plus maze were used to assess anxiety level alterations 24 hours or 1 month following the exposure to Juvenile stress. Following the behavioral assessment, we quantitatively determined the level of expression of alpha1, 2 and 3 in the hippocampus and amygdala. Abnormal pattern of alpha1, 2 and 3 subunits expression was found mainly in the amygdala one month, but not 24 hours after the exposure to juvenile stress. These results suggest that juvenile stress induces a faulty maturation of the GABAergic system, particularly in the amygdala.

# Abstract: S13: Novel vistas on brain dopamine

*Organizer*: Carmen Cavada (Université Autónoma de Madrid, Madrid, Spain)

The goal of the symposium is to present current, timely concepts on the location, mechamisms of action, and consequences of dysfunction of dopamine in the brain. This goal will be directly addressed by the first three speakers. M. A. García-Cabezas is a young licensed pathologist who has done his Ph.D. thesis in C. Cavada's lab working on the distribution of dopaminergic axons in the primate thalamus. This will be the main focus of his presentation. It will be preceded by a brief summary presentation of the brain dopaminergic systems. A. A. Grace has made important contributions on dopamine release mechanisms, which will be the core of his contribution. J. A.Obeso is a reputed neurologist specializing in Parkinson's disease who will address the effects in humans of dopaminergic dysfunction in the motor domain and beyond.

Professor Hornykiewicz's contribution represents the golden finale to the symposium. His personal account on the discovery of the neuropathology of Parkinson's disease is not only delightful; it is most educational and stimulating. Professor Hornykiewicz is still quite active in research and is a brilliant speaker.

- **Miguel Ángel García-Cabezas** (Université Autónoma de Madrid, Spain) Dopamine reaches further brain domains: the innervation of the monkey and human thalamus
- **Anthony A. Grace** (University of Pittsburgh, Pa, USA) Tonic and phasic dopamine system regulation of information flow
- **José A. Obeso** (University of Navarra, Spain) Motor and behavioral consequences of abnormal dopaminergic stimulation in humans
- **Oleh Hornykiewic** (Medical University of Vienna, Austria) The discovery of brain dopamine deficit in Parkinson's disease: the story of an eyewitness

# Abstract: DOPAMINE REACHES FURTHER BRAIN DOMAINS: THE INNERVATION OF THE MONKEY AND HUMAN THALAMUS

**Authors:** M. A. García-Cabezas, M. A. Sánchez-González, B. Rico, P. Martínez-Sánchez, M. Garzón, C. Cavada

**Affiliations:** ^1^Departamento de Anatomía, Histología y Neurociencia, Facultad de Medicina, Universidad Aut.noma de Madrid, Madrid, Spain; ^2^Instituto de Neurociencias de Alicante, Universidad Miguel Hernández-CSIC, Sant Joan d'Alacant, Spain

The existence of significant dopamine innervation in the primate thalamus has been ignored until recently probably because of the reported scant dopamine innervation in the rat thalamus. We recently defined the thalamic dopaminergic system in primates using immunohistochemistry against dopamine and the dopamine transporter (DAT) in monkey and human brains, as well as neural tracing studies in monkey brains. The system arises from numerous dopaminergic cell groups in the hypothalamus and brainstem, and selectively targets numerous thalamic nuclei. The most densely innervated are the midline limbic nuclei, the mediodorsal and lateral posterior association nuclei, and the ventral lateral and ventral anterior motor nuclei. The rat dorsal thalamus holds only sparse dopamine innervation; it is mainly located in the mediodorsal, paraventricular, ventral medial, and ventral lateral nuclei. The reticular nucleus is notably innervated in both primates and rats. The main targets of DAT-expressing axons in the macaque mediodorsal nucleus are thalamic interneurons as revealed by ultrastructural analysis. Perhaps the marked expansion of the dopamine innervation in the primate versus the rodent thalamus is related to the presence of a sizable interneuron population in the primate. Considering the distribution of dopaminergic axons in the primate thalamic nuclei, we propose that thalamic dopamine may play a role in attention, emotion, cognition, sleep regulation, complex somatosensory and visual processing, and motor control. The thalamic dopaminergic system could also be related to the pathophysiology of Parkinson's disease and schizophrenia, and may be a relevant site of action for some drugs of addiction.

# Abstract: TONIC AND PHASIC DOPAMINE SYSTEM REGULATION OF INFORMATION FLOW

**Authors:** A. A. Grace, S. B. Floresco, Y. Goto, D. L. Lodge, A. R. West

**Affiliations:** Departments of Neuroscience, Psychiatry and Psychology, University of Pittsburgh, Pittsburgh, PA, USA

Increasing evidence suggests that the dopamine (DA) system is functionally compartmentalized, with DA release occurring via two processes: a steady-state tonic DA system that controls the low levels of DA present in the extrasynaptic space in the striatum, and a rapid, high amplitude intrasynaptic phasic release. Our data show that the tonic DA system is dependent on the population activity of the DA neuron population, which is the proportion of spontaneously firing DA neurons that is controlled by a pathway originating in the ventral subiculum of the hippocampus. In contrast, the phasic DA release is determined by burst firing of DA neurons in response to behaviorally salient stimuli; this response is regulated by glutamatergic afferents from the pedunculopontine tegmentum that innervate ventral tegmental DA neurons. Our studies have shown that the tonic and phasic dopamine system work together to regulate information flow within the nucleus accumbens. Thus, tonic DA is found to selectively inhibit prefrontal cortical afferent input to the accumbens via D2 receptor stimulation, whereas phasic DA preferentially increases limbic ventral subicular inputs via a D1-mediated process. Therefore, increases in tonic and phasic DA release would shift information flow away from prefrontal cortical drive and towards a limbic predominance. Such a shift is reflected in the behavior of the animal, with disruption of phasic limbic drive interfering with acquiring a task, and disruption of tonic prefrontal cortical input leading to perseverative behavior. Repeated cocaine treatment was found tomimic the effects of limbic predominance, which we propose underlies the focus on drug-seeking behavior while interfering with the ability of the prefrontal cortex to switch to more behaviorally effective strategies.

# Abstract: MOTOR AND BEHAVIORAL CONSEQUENCES OF ABNORMAL DOPAMINERGIC STIMULATION IN HUMANS

**Authors:** J. A. Beso

**Affiliations:** University of Navarra

There may be several human disorders associated with abnormalities of the dopaminergic (DAergic) system. This may include schizophrenia and psychosis, Gilles de la Tourette syndrome and restless leg syndrome. However, direct proof of DAergic dysfunction is only available for Parkinson's disease(PD). Here, neuronal degeneration of dopamine (DA) cells in the substantia nigra is associated with profound reduction of striatal dopamine. Replacement of such deficit with levodopa is associated with very high levels of intracerebral DA levels eventually giving rise to excessive DAergic stimulation. Patients with PD represent therefore, an opportunity to study the consequences of two extreme situations, i.e., severe DAergic deficit and excessive DA availability. The former (i.e., untreated PD) is associated with reduced movement capacity (bradykinesia) and rigidity as well as reduced initiative and depression. The latter (i.e., levodopa treated PD) leads to excessive involuntary movements or dyskinesias, behavioral abnormalities and psychosis.

These different clinical expressions mediated by changes in DA availability probably represent activation of several cortico-basal ganglia-thalamo-cortical circuits. Thus, they represent at the same time a clinical challenge but also a chance to better understand the organization of the dopaminergic system.

# Abstract: THE DISCOVERY OF BRAIN DOPAMINE DEFICIT IN PARKINSON'S DISEASE: THE STORY OF AN EYEWITNESS

**Authors:** O. Hornykiewicz

**Affiliations:** Center for Brain Research, Medical University of Vienna, Vienna, Austria

Dopamine (DA) was first synthesized in the laboratory in 1910 and characterized pharmacologically as a weak sympathomimetic agent. Following the discovery in 1938 of the enzyme dopa decarboxylase and the demonstration that in animal tissues this enzyme catalyzed the formation of DA from the amino acid 3,4-dihydroxyphenylalanine (L-DOPA), DA was assigned the role of an intermediate metabolite in the biosynthesis of noradrenaline and adrenaline in the body. In 1957 the occurrence of DA in the mammalian, including human, brain was discovered. In 1959 DA was shown to be highly concentrated in the nuclei of the basal ganglia. This striking localisation, together with earlier observations by various authors showing that the DA precursor L-DOPA caused motor overactivity, antagonized the reserpine “tranquilization”, increased brain catecholamine levels, and replenished the brain DA depleted by reserpine, suggested that DA might be involved in the basal ganglia control of movement and the “reserpine parkinsonism”. Combining these various data into a single concept, in 1959 a study of DA in the brain of patients with Parkinson's disease (PD) was started in Vienna, demonstrating in 1960 a severe striatal DA deficit, specific for PD. This discovery led directly to the DA replacement concept with L-DOPA and in 1961 to the first highly successful clinical trials with L-DOPA in PD patients. In 1963 the DA deficit in the PD substantia nigra was demonstrated, paving the way for studies on the existence of the nigrostriatal DA pathway and the recognition that the loss of nigral DA neurons was the cause of the striatal DA deficit in PD. Although brain DA's path to recognition was not always smooth, today it is generally recognized that the DA/L-DOPA/PD story is one of the greatest success stories of modern neuroscience.

# Abstract: S14: Is the neocortex fundamentally multisensory? A look at the evidence

*Organizers*: Christoph Kayser (Max Planck Institute for Biological Cybernetics, Tübingen, Germany) and Asif Ghazanfar (Princeton University, Princeton, NJ, USA)

Sensory neurobiology is traditionally investigated one modality at a time, though it has long been known that real world behaviour is mediated by integrating information from multiple sensory sources. A number of recent results suggest that the neocortical underpinnings of this multisensory integration reach beyond association cortices and into early sensory cortical areas. This symposium will explore the role of multiple senses in driving behavior and how the integration of multiple senses is mediated by neocortical operations in both human and nonhuman primates.

Four experts will be brought together to present their recent work and discuss how understanding multisensory integration sheds light on fundamental questions related to sensory processing and large-scale interactions in the brain. The proposed speakers investigate this phenomenon using complementary methods–from fMRI and EEG to electrophysiology and behavior–and using complementary data from both humans and monkeys.

Together, they will show that the pervasiveness of multisensory influences on sensory perception and on all levels of cortical processing will force neurobiologists to reconsider the practice of thinking about brain and behavior in unisensory terms.

- **Micah Murray** (University of Lausanne, Switzerland) Multisensory interactions redefine functional cortical organization in humans
- **Nicholas P. Holmes** (INSERM Unite 864, Bron, France) Body, brain & space: Multisensory perception by eyes, hands, & tools
- **Uta Noppeney** (Max Planck Institute for Biological Cybernetics, Tübingen, Germany) Audio-visual interactions within the cortical hierarchy
- **Christoph Kayser** (Max Planck Institute for Biological Cybernetics, Tübingen, Germany) Multisensory integration in early auditory areas

# Abstract: MULTISENSORY INTERACTIONS REDEFINE FUNCTIONAL CORTICAL ORGANIZATION IN HUMANS

**Authors:** M. M. Murray

**Affiliations:** ^1^Electroencephalography Core, Center for Biomedical Imaging, University of Lausanne, Lausanne and Geneva, Switzerland; ^2^Neuropsychology and Neurorehabilitation Service, Centre Hospitalier Universitaire Vaudois and University of Lausanne, Switzerland; ^3^Radiology Service, Centre Hospitalier Universitaire Vaudois and University of Lausanne, Switzerland

Traditional models of sensory processing contend that sensory information is analyzed in relative independence before being combined at higher-order “multisensory” regions. This talk will first present findings in humans using EEG, fMRI, and TMS that instead support a model wherein there are direct interactions between low-level, nominally “unisensory” regions. In particular, new methodological advances in hemodynamic and electrical neuroimaging are revealing multisensory convergence and interactions within primary cortices and during the initial 100 ms of sensory processing. Second, it will be shown how multisensory processes are providing a better understanding of the likely functional organization of cortical areas. This will be shown for the case of spatial processing, ongoing brain responses, and also for memory functions. In particular, multisensory experiences enrich our memories, despite only single-trial exposure, and influence ongoing sensory processes at extremely early latencies in areas traditionally considered unisensory in their function. Multisensory experiences are registered by the brain even when of no immediate behavioral relevance and can be used to categorize ongoing experiences. Episodic and semantic factors contributing to this efficacy are likewise discussed that reveal a possible object' rule of multisensory effects. Multisensory integration in early auditory cortex.

# Abstract: BODY, BRAIN & SPACE: MULTISENSORY PERCEPTION BY EYES, HANDS, & TOOLS

**Authors:** N. P. Holmes

**Affiliations:** Espace et Action, INSERM Unite 864, Bron, France

The sensory interface between the body and the outside world is defined through the integration of visual, tactile, and proprioceptive information. In the first part of my talk, I will show how the felt location of our hands is computed via a weighted sum of visual and proprioceptive position information. Seeing our hand displaced from its true location (using mirrors and rubber hands) has compelling effects on its felt location, and can significantly bias the trajectories of subsequent movements. In the second part, I show, using fMRI, that perceiving distant vibrotactile stimuli via a hand-held tool relies substantially upon visual inputs and visual reference frames. Furthermore, activity in low-level visual cortex covaries significantly with vibrotactile discrimination performance. Together, this work shows that visual information about the body, and about peripersonal space, directly influences proprioceptive and tactile representations. Body and space in the brain are inherently multisensory.

# Abstract: AUDIO-VISUAL INTERACTIONS WITHIN THE CORTICAL HIERARCHY

**Authors:** U. Noppeney

**Affiliations:** Max-Planck-Institute for Biological Cybernetics

To interact effectively with our environment, the human brain integrates information from multiple senses into a coherent percept. Neurophysiological and functional imaging studies have revealed multi-sensory interactions in a widespread neural system encompassing subcortical structures, putative ‘unisensory’ and higher order association cortices. Combining fMRI and psychophysics, we investigated where and how different types of sensory features are combined within the cortical hierarchy. We presented subjects with object pictures and sounds while factorially manipulating the relative informativeness of the auditory and visual modalities. While low level spatio-temporal interactions were found within Heschl's gyrus, higher order object features were integrated within the superior temporal sulci (STS) bilaterally. Consistent with the law of inverse effectiveness, the multisensory interactions in STS were primarily suppressive for intact, but (super)additive for degraded stimuli. These distinct modes paralleled behavioral indices of multi-sensory enhancement showing the greatest multisensory benefit for degraded stimuli. In conclusion, the human brain integrates information that is abstracted from its sensory inputs atmultiple levels of the cortical hierarchy. The operational mode of audio-visual integration is dictated by the informativeness of the auditory and visual modalities.

# Abstract: MULTISENSORY INTEGRATION IN EARLY AUDITORY CORTEX

**Authors:** C. Kayser

**Affiliations:** Max-Planck-Institute for Biological Cybernetics

An increasing body of literature from functional imaging, electrophysiology and anatomy provides compelling evidence that merging of sensory information not only occurs in higher association areas, but also in lower sensory regions. To investigate early cross-modal interactions in detail, we use the macaque auditory cortex as model and employ a combination of high-resolution imaging (fMRI) and electrophysiological recordings. In the imaging data, few voxels respond to non-auditory stimulation alone, but many show cross-modal interactions in the form of supra-linear enhancement; i.e., the multimodal response exceeds the linear superposition of the unisensory responses. This effect is reliably found at the caudal end and along the lateral side of the secondary auditory cortex, and can be localized to the medial and caudal belt and caudal parabelt regions. This interaction obeys the classical rules for sensory integration: it occurs only for temporally coincident stimuli and follows the principle of inverse effectiveness (integration is stronger for less effective stimuli). Complementary electrophysiological recordings demonstrate that the imaging results are nicely paralleled by similar findings in the low frequency local field potentials. Individual neurons, however, often show the opposite effect and exhibit a decreased response when a visual stimulus is presented simultaneously with a sound. This audio-visual depression occurs with a time lag of about 40–80 ms, and for a wide range of simplistic and naturalistic stimuli.

Altogether, our results clearly support the notion that early sensory cortices are susceptible to modulation by different senses. However, for individual neurons these effects are subtle and can be better detected at the level of population responses. Future studies need to resolve where exactly this cross-modal input originates and how it aids the auditory system to segregate our complex acoustical environment.

# Abstract: POSTERS  Theme A: Sensory & motor systems  SPATIOTEMPORAL CLASSIFICATION OF FACIAL EXPRESSION AND IDENTITY REPRESENTATIONS USING HIGH-RESOLUTION IMAGING

**Authors:** N. Furl, J. Mourão-Miranda, N. Weiskopf, R. J. Dolan

**Affiliations:** ^1^Wellcome Trust Centre for Neuroimaging, University College London, London, UK; ^2^Institute of Psychiatry, Kings College London, London, UK

To what extent are visual category representations localised or distributed? And how does experience with a category change this representation? Functional magnetic resonance imaging (fMRI) studies of visual coding predominantly rely on mass-univariate general linear models (GLMs) to identify regions where localised activations show domain-specific preferences for categories such as faces, nonface body parts, places, objects. In contrast, distributed activation patterns whose profiles accurately discriminate visual categories have been identified by a growing number of studies. We discriminated distributed brain activity patterns corresponding to facial identity and expression categories using multivariate pattern classification (often characterised as “mental state decoding”). At high resolution (1.5 mm isotropic voxel size), we scanned the ventral stream as participants viewed blocks of faces. For each block, one of seven identities was repeated (while expressions and viewpoints changed) or one of seven expressions was repeated (while identities and viewpoints changed). Ventral stream regions (including the fusiform gyrus) showed adaptation selective to repetitions of either expression or identity categories, indicating category information in the temporal response dynamics. We capitalised on such category-selective dynamics associated with adaptation by using a spatiotemporal multivariate classifier, which uses both spatial and temporal information.

# Abstract: FUNCTIONAL ORGANIZATION OF THE VISUAL CORTEX DURING ANTICIPATORY AND STIMULUS-DRIVEN ATTENTION

**Authors:** A. Wróbel, W. Bogdan, M. Bekisz, J. Smyda

**Affiliations:** Nencki Institute of Experimental Biology, Warsaw, Poland

We have previously shown that an attentional mode of the visual system is characterized by enhanced beta frequency activity in local field potential recordings as compared to a situation requiring auditory attention. This beta activity appears in the primary visual cortex of behaving cats regardless of whether attention is evoked by a visual stimulus or by an anticipatory paradigm [1, 2]. In order to reveal the cortical activation pattern in both behavioral situations, we measured amplitudes of potentials (EPs) evoked from 18 recording sites (all located at deep layer 4) in eight cats (four trained in either paradigm) by electrical stimulation of the optic chiasm. We found that

- 200 ms periods of enhanced beta activity were followed by EPs of increased amplitudes during anticipation, and by EPs of smaller amplitudes during stimulusinduced attention as compared to control EPs—which implied that direction of polarization across the cortical depth was opposite in either situation;
- a correlation calculated between raw beta signals recorded in the anticipatory situation from neighboring electrodes in the visual cortex was similar irrespective of a modality of the task (visual or auditory), but highly varied when attention was induced by a visual (instead of auditory) stimulus.

We suggest that the idle beta oscillatory rhythm observed in the primary visual cortex during nonvisual situation changes towards a specific pattern of beta synchronization during attentive seeing. The anticipatory paradigm results in a depolarization of superficial layers over a large cortical area whereas the stimulus-driven attention sets a temporary activated mosaic of functional columns, as needed for a current visual scan.

## References

[1] Bekisz M, Wróbel A (2003). Attention-dependent coupling between beta activities recorded in the cat's thalamic and cortical
representations of the central visual field. *European Journal of Neuroscience*.

[2] Wróbel A, Ghazaryan A, Bekisz M, Bogdan W, Kamiński J (2007). Two streams of attention-dependent *β* activity in the striate recipient zone of cat's lateral posterior-pulvinar complex. *Journal of Neuroscience*.

# Abstract: COMPLEX AND SIMPLE CELLS ARE IDENTITY AND ATTRIBUTE VARIABLES IN A GENERATIVE MODEL OF NATURAL IMAGES

**Authors:** P. Berkes, R. Turner, M. Sahani

**Affiliations:** Gatsby Computational Neuroscience Unit, London, UK

A promising approach to the study of representation in the visual system is based on the idea that neuronal response properties reflect an internal model of the statistical structure of the environment, and can thus be derived or learnt from the structure and parameters of a statistical model of sensory observations, following the Helmholtzian notions of perception as an inferential process. Here, we present a generative model of visual input based on the fundamental coupling between the identity of an object or feature and its attributes. In our model, observations are generated from a set of binary identity variables, representing the presence of a visual feature.

The appearance of each feature is modelled by amanifold that represents its episodic pose, and is parametrised by a set of attribute variables.

When the model is applied to natural videos, the resulting attribute manifolds are spanned by a small set of Gabor wavelets with similar position, orientation, and frequency, but with different phase.

The activity of attribute variables thus resembles that of simple cells in the primary visual cortex (V1). Identity variables indicate the presence of a feature irrespective of its position on the underlying manifold, and are therefore insensitive to the phase of the input. Their behaviour is thus similar to that of complex cells. The properties of the variables' receptive fields are found to be consistent with physiological data from V1.

This generative model makes explicit an interpretation of complex and simple cells as elements in the segmentation of a visual scene into independent features along with a parametrisation of their episodic appearance. It also indicates their possible role in a hierarchical system that extracts progressively higher-level contents, starting from simpler, low-level features.

# Abstract: FORGETTING RATE OF TOPOGRAPHICAL KNOWLEDGE IN HUMANS IN ABSENCE OF RECONSOLIDATION

**Authors:** C. L. Latini, C. Thinus-Blanc, M. P. Nesa, G. C. Geminiani, P. Péruch

**Affiliations:** ^1^Dipartimenti di Psicologia, Università degli Studi di Torino, Torino, Italy; ^2^INSERM U-751 Epilepsie & Cognition, Faculté de Médecine de la Timone, Marseille, France

Topographical knowledge is necessary for efficient navigation toward unseen goals. Route knowledge corresponds to the memory trace of the sequence of landmarks encountered along a specific route, while survey knowledge allows direct access to the global layout of an environment. In normal humans, the few available data on the retention of topographical memory show no systematic decline over months or years. In the present experiment, two groups of participants elaborated route and survey knowledge during navigation in the same complex virtual environment before performing two route tasks (estimating the number of turns and the route distance between landmarks) and two survey tasks (pointing to unseen landmarks and estimating the straightline distance between landmarks). Both groups were tested immediately after learning and three months later, while one group was also tested one week and one month later (repeated testing). Following the first testing session, performance was similar in both groups. On subsequent sessions, while performance of the repeated tested group remained stable, it decreased significantly in the nonrepeated tested group, especially on route tasks. Survey knowledge was more resistant to decay probably because of its abstract structure. These data are the first to reveal a substantial and selective decline of topographical memory in humans, occurring only if there is no possibility to reactivate knowledge along successive testing phases, that is, in the absence of reconsolidation.

# Abstract: MODULATION OF AUDITORY RESPONSES BY THE PREVIOUS HISTORY OF STIMULATION IN THE AWAKE ANIMAL

**Authors:** J. M. Abolafia, M. M. Arnold, R. Vergara, M. V. Sanchez-Vives

**Affiliations:** Instituto de Neurociencias de Alicante, Universidad Miguel Hernández-CSIC, Spain

Sensory responses, even in primary sensory areas, are deeply influenced by the previous history of stimulation. Thus, repetitive stimulation often induces adaptation to the stimulus and a decreased response. On the other hand, in an awake animal, previous exposure to stimuli may vary how that stimulus is experienced: it can be attended, feared, highly expected or neglected according to its acquired meaning. To what extent does this higher processing of a simple stimulus alter the responses of single-neuron primary sensory cortices? In order to answer this question and to characterize how repetitive stimulation shapes auditory cortical responses, we recorded from A1 and A2 in awake behaving rats. Recordings were obtained with chronically implanted tetrodes that allowed us to identify the recordings of up to 5-6 single units simultaneously. To study adaptation, two identical white noise stimuli were delivered with different intervals ranging from 50 ms to 8 s. Single neuron's recording revealed that the response to a sound is influenced by sounds delivered even seconds earlier, the second one usually yielding a weaker response (see Figure 1). Adaptation occurred mainly in two time scales: one of hundreds of ms and another one of seconds (<10 s), the time course of adaptation and its recovery being determined by the intensity of the first stimulus of the pair. Our results suggest that adaptation to repetitive stimulation does not differ from the one that can be evoked with similar protocols in other preparations of the auditory cortex, therefore it seems to depend strictly on bottom-up mechanisms. On the other side, we have observed modulation of auditory responses depending on the preceding sequence of sounds and thus the expectancy of certain stimuli. We hypothesize that the activation of top-down mechanisms is underlying this phenomenon. 

## Figures and Tables

**Figure 1:** 

# Abstract: EXPLORING THE F-DTI HYPOTHESIS

**Authors:** G. Garbin

**Affiliations:** University of Trieste, Trieste, Italy

Diffusion tensor imaging (DTI) is an MR technique that allows the visualisation the white matter bundles by identifying the free mean path directions of the molecules of water in the brain volume. In functional MRI (fMRI), the different behaviour of oxy-haemoglobin and deoxy-haemoglobin in a magnetic field is used to identify functional areas on the brain cortex. The two techniques can be applied separately on a same subject to identify the white matter bundles that connect separated functional areas on the cortex. Nevertheless, using this combination of the two techniques gives as result a certain number of bundles and does not allow to identify which fibers of the white matter are indeed responsible for signal transportation during a specific task. A different approach in integrating fMRI and DTI may be found in a technique to show the activity in the white matter bundles rather than in the vessels near the target neurons. In this work, the hypothesis of obtaining fMRI-like activations from DTI acquisitions is explored. Indeed, fMRI takes into account blood oxygenation variations, while DTI is a technique to measure the behaviour of the molecules of water in an anisotropic mean. The presented hypothesis comes from the consideration that the action potentials that are generated during a specific task may affect the mean diffusivity of water molecules in the axons, and this may be revealed by a confrontation of images of fractional anisotropy obtained with a normal DTI protocol as if the images belonged to an fMRI session. This communication refers to two experimental MR-DTI acquisitions and postacquisition processing that used the same algorithms that compete to fMRI. The two experiments were made using a 1.5T tomograph and two different stimuli: in both cases, the correlation of the fractional anisotropy images with an event-related time pattern shows at least one identified variation in the white matter response near the interested cortical area.

# Abstract: PERCEPTUAL GROUPING IN FISH: GLOBAL AND LOCAL VISUAL PROCESSING

**Authors:** L. Frassine, V. A. Sovrano, V. Truppa, A. Bisazza

**Affiliations:** ^1^Department of General Psychology, University of Padua, Padua, Italy; ^2^Institute of Cognitive Sciences and Technologies, CNR, Rome, Italy

Different visual grouping principles may operate in different species among vertebrates. Adult humans show a global bias in visual grouping, whereas nonhuman primates show mixed results depending on the density of the elements in the stimuli. This study analyzes global versus local preferences in perceptual grouping of hierarchically organized compound stimuli in red tail split fins (*Xenotoca eiseni*). Fish were trained to discriminate between a circle made of small circle elements and a cross made of small cross elements. They were then tested for choice between a circle made of small crosses and a cross made of small circles. Fish showed a global bias in testing, irrespective of the density of the elements in the stimuli. The results were not due to a lack of attention for local elements during the training, because when fish were tested with the local elements in isolation, a small disc and a small cross, they chose in accordance with the stimulus of training. These results thus suggest that the “global precedence effect” (Navon effect) is not a peculiar human characteristic and that grouping processes for object identification may be different among vertebrates, probably linked to the ecology and the evolutionary adaptation of different species.

# Abstract: RECOGNITION OF OBJECTS FROM IMPOVERISHED STIMULI IN NEWLY HATCHED VISUALLY NAïVE CHICKS (GALLUS GALLUS)

**Authors:** E. Mascalzoni, L. Regolin

**Affiliations:** Department of General Psychology, University of Padova, Italy

We investigated the ability to recognise a three-dimensional object from two-dimensional cues in a highly visual animal species, the domestic chick, taking advantage of the ecological procedure of filial imprinting. Newly hatched visually naïve chicks were imprinted for 2 days to a solid object and then required to choose between two 2D images, the shadow of the familiar object and the shadow of an object never seen before, in either a static or rotating condition. In Experiment I, stimuli were selected to maximise differences in their projected shadows; in Experiment II, the objects had controlled dimensions so that the testing images would differ minimally. Results showed that chicks are able of discriminating between familiar and novel objects on the basis of their cast shadows and revealed that the motion of the solid stimuli affects this process, being crucial for discrimination of stimuli that differ minimally. In Experiment III, test stimuli were point-of-light patterns moving coherently and producing the perception of a solid shape only when in motion. In these conditions, it seems that chicks were not able to recognise the familiar solid, probably because of the high level of generalisation required by test stimuli which had lost all features of their corresponding solids. For this reason, in Experiment IV, chicks were imprinted and tested with point-light displays. Results suggested that chicks can discriminate the two test stimuli from one another. In conclusion, it seems that domestic chickens, at a very early stage of development, are able to recognise familiar objects by 2D impoverished stimuli. Such recognition is sensitive to fine differences whenever motion cues are provided (i.e., stimuli rotation, which allows temporal integration of the different visual perspectives). Moreover, chicks showed being able of object recognition from point-light displays requiring the sheer extraction of structure-from-motion.

# Abstract: A NEW PARADIGM FOR THE STUDY OF THE ROLE OF THE WHISKER INFORMATION

**Authors:** T. Gener, R. Reig, M. V. Sanchez-Vives

**Affiliations:** Instituto de Neurociencias de Alicante, Universidad Miguel Hernández-CSIC, Spain

The objective of this study was to explore a paradigm that would allow a temporary deprivation of whisker information which could be used both in awake behaving and in anesthetized preparations. With that aim we characterized topical application effect of lidocaine to the whiskers base. Extra and intracellular barrel cortex recordings from a ketamineanesthetized rat were obtained and the response to a puff of air delivered to the whiskers adjusted to evoke a response of 40–100 microV (extracellular) and 2–10 mV (intracellular). Lidocaine was locally applied in different forms (cream, local injection, aerosol, liquid drop) and concentrations (2–10%). The response to an air puff (3–10 ms) delivered to the whiskers was averaged over 50–60 trials (5s ISI) in 16 rats. Local application of lidocaine induced a decrease in the evoked response amplitude (see Figure 1). Even when local injection of lidocaine induced blockade of the responses, we ruled out this form of application since an injection at the snout may be stressful for an awake animal. The application of a liquid drop of lidocaine (0.4 ml, 10%) was found to induce a reliable blockade, which a recovery time of 3–5 hours. The decrease in the response was a constant proportion of the sensory evoked amplitude. To conclude, topical application of liquid lidocaine applied at whiskers base can induce a temporary, reliable and reversible deprivation of whisker information. This approach can therefore be used to investigate the role of whisker information and the short term plasticity induced by its deprivation. Preliminary results indicate that this sensory deprivation has an impact on the integration of whisker information in other cognitive processes in awake animals.

## Figures and Tables

**Figure 1:** (a) Absolute value of local field potential amplitude in barrel cortex (one point represents 60 responses). (b) Simultaneous recording extra (top) and intracellular (bottom) during lidocaine application.

# Abstract: NEURONAL RESPONSES TO VISUAL STIMULI IN AUDITORY CORTICAL AREAS OF MONKEYS PERFORMING AN AUDIO-VISUAL DETECTION TASK

**Authors:** C. Cappe, G. Loquet, P. Barone, E. M. Rouiller

**Affiliations:** ^1^Department of Medicine, University of Fribourg, Switzerland; ^2^Centre de Recherche Cerveau et Cognition (UMR 5549) CNRS, Toulouse, France

On the psychophysical point of view, as compared to unimodal stimuli, multisensory integration leads to improvement of perceptive threshold, as reflected by a decrease of reaction time and better performance in sensori-motor tasks. While such effects have been largely reported for human subjects in auditory-visual recognition tasks, few data are available in behaving monkeys engaged in similar protocols. Multisensory integration is believed to take place mainly in higher order cortical areas. On a behavioral point of view, we have investigated the interaction between auditory and visual stimuli in monkeys. Moreover, the present study aimed at exploring the mechanisms underlying multisensory integration at the level of single neuron during a multisensory motor task in a cortical region considered as unimodal, a dimension that cannot be assessed in human subjects. Two adult macaque monkeys were trained to execute a visuo-auditory detection task. The animals had to generate a motor response in a reaction time paradigm whenever a visual, an auditory or a visuo-auditory signal was presented. By varying the intensity of the individual auditory and visual stimuli we have observed that, near threshold, the bimodal condition had a significant facilitatory effect on reaction times and stimulus detection; this effect disappeared at higher intensities. Electrophysiological recordings were derived from single neurons in the auditory cortex and adjacent zones in the posterior bank of the lateral sulcus. Different types of neuronal responses were observed. Some neurons responded only to auditory stimuli whereas, somewhat surprising, other neurons in the auditory cortex were influenced also by visual stimuli. The auditory cortex contains neurons which respond both to auditory and visual stimuli, confirming other recent studies which suggest that multisensory convergence can occur at low cortical level.

# Abstract: SUPERIOR OCCIPITAL LOBE IS INVOLVED IN PLANNING OF REACHING MOVEMENTS: EVIDENCE FROM TRANSCRANIAL MAGNETIC STIMULATION

**Authors:** P. Busan, F. Monti, M. Semenic, P. P. Battaglini

**Affiliations:** ^1^BRAIN Center for Neuroscience, Department of Physiology and Pathology, University of Trieste, Italy; ^2^Clinical Neurophysiological Unit, University Hospital of Trieste, Italy

We investigated parieto-occipital functionality of the left hemisphere with transcranial magnetic stimulation (TMS) during planning of reaching movements under visual guidance. Ten right-handed healthy subjects were asked to keep their eyes closed while a small metal target was positioned on a horizontal surface in the peripersonal space in front of them. The position was chosen within a choice of three: straight forward, 45° left, 45° right. Subjects were asked to open their eyes at a bell ring and to reach the target with their right hand always keeping foveal view in straight forward position. TMS was applied randomly along repetitions of the task. The instant of TMS pulse was set at 50% of the reaction time from go signal to hand movement. In comparing tasks with or without TMS, we found significantly shorter reaction times (*p* < 0.0087) in left superior occipital lobe (SOL) only. No significance was found in performing the same tasks on other 4 closed parieto-occipital control points, investigated in the same way. Movement time was also measured, never showing significance differences among conditions. Moreover there was not a lateralized effect of target position on reaction times, in fact no differences were evident in SOL between them in the same trial. These results suggest a role of SOL in very early planning of reaching movements. It could be involved in target/movement selection operations. SOL could be part of a planning system that probably includes a human homologue of monkeys area V6A.

# Abstract: EYE TRACKING INVOLVES A COVERT MOTOR PLAN FOR AN AIMING MOVEMENT OF THE ARM: A TRANSCRANIAL MAGNETIC STIMULATION (TMS) STUDY

**Authors:** L. Falciati, T. Gianesini, C. Maioli

**Affiliations:** Department of Biomedical Sciences, University of Brescia, Italy

When we make an aiming movement towards a moving visual object, eye-hand coupling is of paramount importance for accurate motor performance. Some studies suggested that both gaze and manual tracking systems are driven by a common command signal. However, it has never been demonstrated that a motor plan for the arm is produced even when the object is tracked by the eyes alone. By applying TMS to the motor cortex, we show for the first time that ocular tracking is linked to changes in the excitability of the corticospinal system (CSS) of the relaxed upperlimb, as estimated from the amplitude of the motor evoked potentials recorded in contralateral hand and wrist muscles. Subjects had to track with the eyes a target moving at a constant velocity of 10°/s along the horizontal meridian. During each trial, single-pulse TMS was randomly delivered either 100 ms before target motion or during the smooth pursuit (SP) eye movement, triggered by the EOG signal at gaze eccentricities of 5°, 10° or 15°. Changes in excitability consisted in an overall inhibition of the arm CSS, which was modulated in a highly specific manner in the different muscles, depending on the direction of SP eye movements. The observed modulation in CSS excitability was found to be compatible with a motor plan encoding an aiming movement of the hand towards the same target tracked by the eyes. In addition, excitability changes are contingent upon upper-limb posture, as they are present only with a pronated forearm, but not with a supinated hand position. Results demonstrate that, if the arm is held in a congruent postural configuration, tracking a moving object always entails a coordinated motor plan, which involves both gaze and hand movements. Active inhibitory mechanisms are activated in order to prevent an overt arm movement, whenever a manual tracking is not requested. Our data provide strong evidence in favour of the existence of a common drive to both eye and hand tracking systems.

# Abstract: TEMPORAL AND SPATIAL DYNAMICS ON THE CONTROL OF THE REACHING MOVEMENT

**Authors:** D. Bertogna, R. Budai, R. Blanco, P. Battaglini

**Affiliations:** ^1^BRAIN Center, Department of Physiology and Pathology, University of Trieste, Trieste, Italy; ^2^Department of Neurology, University of Udine, S. M. Misericordia Hospital, Udine, Italy

Many researches have shown a complex activation of several cortical areas belonging to a widely distributed frontoparietal network during the planning and the control of reaching movement. Although significant achievements in characterizing processes and structures of the reaching movements, the degree to which visually-directed movements are planned and controlled during execution is still matter of debate. Recent studies have indicated that the motor plan, assembled prior to the onset of movement, does not unfold unaltered but is updated continuously by internal feedback loops. This study aimed at evaluating the temporal and spatial dynamics of such cerebral network during the control of a reaching movement by the recording of event-related potentials (ERPs) in 9 healthy subjects performing a typical targetshift task. Subjects were asked to reach and touch a spot in the centre of a touchscreen. Just after movement onset, the target could stay in the same position or shift either at 20% or 40% of the total reaching trajectory (SHIFT condition). The target could randomly shift towards one of six other positions, 3 for right and 3 for left hemifield. NO-SHIFT condition represented the control condition. The analysis of the event-related dynamics showed a “phase-resetting” phenomenon of alpha, beta1 and beta2 bands, just after the shift of the target. The early phase synchronization was evident on shift condition for both hemifields and for both shift delays. It was absent on the control condition. From source analysis, it emerged a multiple synchronized activity of the fronto-parieto-occipital network, a constant activation of posterior regions during all the task and a controlateral involvement of motor regions with regard to the shift direction. Data show a strong parallel activation of fronto-parieto-occipital network during the execution of the visuomotor task and underline the forward proprieties regarding the online control of the reaching movements.

# Abstract: REINFORCING MALE SEXUAL PHEROMONES ARE NON-VOLATILE CHEMOSIGNALS DETECTED BY THE VOMERONASAL ORGAN

**Authors:** J. Martínez-Ricós, C. Agustin-Pavon, E. Lanuza, F. Martinez-García

**Affiliations:** ^1^Department of Functional Biology, Faculty of Biology, Universitat de València; ^2^Department of Cell Biology, Faculty of Biology, Universitat de València

Bedding that has been soiled by male mice contains sexual pheromones that are innately attractive and reinforcing to females [1]. Since these attractive sexual pheromones are non volatile, we decided to check if they are detected by the vomeronasal organ by testing whether electrolytic lesions of the accessory olfactory bulb (AOB) of naïve females (reared in the absence of male odours) affect their attraction to male-soiled bedding. To do so, we used two-choice preference tests in which we compared the time that sham and AOB-lesion female mice spent exploring intact- vs castrated-male soiledbedding (Experiment 1).

To ensure that olfactory function was not affected by AOB lesions we also performed habituation-dishabituation tests (Experiment 2) in both the sham-operated and AOBlesioned females.

Statistical analysis of the resulting measures showed that sham females preferred bedding soiled by intact males to that soiled by castrated males, whereas AOB-lesioned females did not. Moreover, AOB-lesioned and sham operated females showed comparable olfactory sensitivity and discrimination, since they were able to discriminate two odorants in habituation-dishabituation tests. Therefore, the lack of preference for male-soiled bedding displayed by AOBlesioned females cannot be attributed to altered olfactory function.

This confirms that the reinforcing male sexual pheromones, which have been shown to be non-volatile, are detected by the vomeronasal organ of the females.

## References

[1] Martínez-Ricós J, Agustín-Pavón C, Lanuza E, Martínez-García F (2007). Intraspecific communication through chemical signals in female mice:
reinforcing properties of involatile male sexual pheromones. *Chemical Senses*.

# Abstract: BEHAVIORAL DISORDERS AFFECTING COGNITIVE PERCEPTION AND THE TRAINING INDUCED BY THE PARENTAL ANXIETY

**Authors:** D. Bensoltana, B. Asselah

**Affiliations:** Neurosciences, Algres, Algeria

The bond parental-child set a paramount weaving creating their significant interactive relation; it is the stage of evolution and the installation; various structures and components: neurophysiological and sensory. The majority of the investigations give the cause of the neuroses to this “phase of construction which is childhood”; characterized by the elementary period of training; access to the rudimentary factors memorizing the lexicon. Any factor stressing according to its intensity; deteriorate the degree in performance, and affects cognitive faculties brought into play; This is translated biensur by harmful behavioral disorders. And explains a brittleness of maturation which instead of installing the constructive elements there; span yourself towards the disaster. Because parents delivered to them same unconsciously; during the scandalous situations panics torturing and of the traumatic times without return do not envisage; that they induce with their children with the detriment of their childhood (transmission of the stress related to the fear of with the confrontation of the danger of the one of the two parents and in particular the mother). In order to determine the undeniable impact of the stress environmental on the humorale attitude and behavioral and its mode of action on visual perception and the mnemonic retention [hyper.]. We made a etiologic study on the pathol anxiety while basing ourselves on a comparison of the groups of children affected by stressing factors; subjected to tests of the behavioral and psychic evaluations detecting the disorders and by the recording of their movements of the eyes during the reading their imp acts on the saccadic ocular sizes. The results confirm that visual perception coding information thus models the cognitive answers continuations to the afférences; that the stress of with the attachment generates typical characterial functional instabilities determining control and the personality and disturbing the schooling.

# Abstract: THE INFLUENCE OF ANANDAMIDE ON THE ENDOMOR-PHIN-1-INDUCED ANTIHYPERALGESIA

**Authors:** G. Horvath, G. Tuboly, G. Benedek

**Affiliations:** Department of Physiology, Faculty of Medicine, University of Szeged, Hungary

Several studies have proved synergistic interaction between opioid- and cannabinoid drugs, but the effects of coadministration of their endogenous ligands were not investigated. The goal of this study was to determine the antinociceptive interaction of endogenous ligands, endomorphin-1 and anandamide, acting opioid and cannabinoid receptors, respectively at spinal level. After obtaining institutional ethical approval, intrathecal catheters were implanted into male Wistar rats. A paw withdrawal test was used for nociceptive testing in carrageenan-induced (2 mg) thermal hyperalgesia in male Wistar rats. The drugs were injected intrathecally alone or in combinations (0.01–10 *μ*g endomorphin-1 and 1.5–100 *μ*g anandamide). Both substances by themselves caused a dose-dependent antihyperalgesic effect, but endomorphin-1 had higher potency, and the largest dose of anandamide caused painful behavior too. The coadministration of 30 g anandamide with lower doses of endomorphin-1 (0.01, 0.1 and 0.3 *μ*g) and 10 *μ*g anandamide with 0.01 endomorphin-1 caused potentiated antihyperalgesia, while other combinations were not more effective than endomorphin-1 alone.

In conclusion, combinations of these ligands in special dose-ranges furnish potentiated antihyperalgesia. A possible mechanism of the antinociceptive interaction of these endogenous ligands might be a complex coactivation of opioid and cannabinoid receptors. Since anandamide activates TRPV1 receptors too, the net effect after their coadministration is due to the complex changes.

# Abstract: HYPERCOLUMNS VS. PINWHEELS

**Authors:** M. Valverde, V. Braitenberg

**Affiliations:** Max Planck Institute for Biological Cybernetics, Tübingen, Germany

“Optical imaging” maps of the visual cortex after systematic application of variously oriented visual stimuli provide an opportunity to test different hypotheses on the distribution of orientation sensitive neurons over the surface of the cortex. Rectilinear “slabs” of uniform orientation, as postulated in some earlier models, are not supported by the evidence. What is compatible with the optical imaging maps is the arrangement of neurons with different orientation around centers, regularly spaced at distances of about 0.5 mm in a hexagonal array. According to the model proposed by [3], the orientations to which the neurons are sensitive should be arranged either radially, or, more likely, like the tangents [1] of circles around said centers, whereby in either case twice the same orientation occurs in opposite positions of the “hypercolumn” thus defined. The centers of the hypercolumns very likely coincide with the so-called cytochrome oxidase “blobs” which are spaced at the same distance. The fact that within these “blobs” orientation tuning of cortical neurons becomes undefined [4], makes the array of orientations around these centers less spectacular, and indeed other interpretations of the coloured maps produced by optical recording were put forward. So-called “pinwheels” stole the show, that is centers around which neurons with different orientation sensitivity crowd with the colours representing their orientation clashing without interposed indifferent regions. In these pinwheels each of the different orientations occurs only once as you go full circle around their center. They most likely correspond to the corners between the hypercolumns in their hexagonal array, and the different orientations within one “pinwheel” most likely belong to three different hypercolumns that meet there [2].

The distinction between the two entities, orientation hypercolumns and pinwheels may sound academic but becomes crucial when one endeavours to underpin orientation specificity of cortical neurons with schemes of neuronal interactions at the elementary level. The accompanying illustration should help the reader to partake in this discussion.

## Figures and Tables

**Figure 1:** Color encoding of a “hypercolumn” (inset 1) and interpretation of an optical imaging picture from [5]. Both, “hypercolumns” (1, 3) and “pinwheels” (2) can be found (arrows point to examples). A detailed analysis of such pictures is compatible with the assumption that the number of pinwheels is twice the number of hypercolumns and that the distance between hypercolumn centers corresponds to the distance between blob centers.

## References

[1] Braitenberg V, Peters A, Jones EG (1985). Charting the visual cortex. *Cerebral Cortex: Volume 3: Visual Cortex*.

[2] Braitenberg V, Aertsen A, Braitenberg V (1992). How ideas survive evidence to the contrary: a comment of data display and modelling. *Information Processing in the Cortex: Experiments and Theory*.

[3] Braitenberg V, Braitenberg C (1979). Geometry of orientation columns in the visual cortex. *Biological Cybernetics*.

[4] Livingstone MS, Hubel DH (1984). Anatomy and physiology of a color system in the primate visual cortex. *Journal of Neuroscience*.

[5] Obermayer K, Blasdel GG (1993). Geometry of orientation and ocular dominance columns in monkey striate cortex. *Journal of Neuroscience*.

# Abstract: READING THE GRIDS: LEARNING, PLACE-FIELD FORMATION AND PATTERN-SEPARATION

**Authors:** G. Papp, A. Treves

**Affiliations:** ^1^SISSA, International School for Advanced Studies, Trieste, Italy; ^2^Center for the Biology of Memory, Norway University of Science and Technology, Trondheim, Norway

The hippocampus includes three main fields, DG, CA3 and CA1, all receiving their main inputs from entorhinal cortex (EC). In terms of neural activity, the prominent firing pattern of the CA areas are the place-fields that in usual experimental enclosures tend to have single peaks. Recent studies have demonstrated “place” units with multiple peaks in DG, and with a regular triangular grid-like firing pattern in medial EC (mEC). Several computational studies have tried to show how the place-fields of the CA areas may form from triangular grids. What seems to emerge is that by just combining different frequencies and orientations the result tends to be multiple fields. This points to the need for a mechanism that chooses from the many possible peaks provided by the mere combination of EC grids. In our studies, we examined the role DG and lateral EC (lEC) plays in learning and place-field formation.

We used a simplified hippocampal model network consisting of DG, CA3 and CA1. The inputs to the network came from both mEC and lEC. In mEC we modeled the firing rates of cells as place-fields arranged on a triangular grid, with some noise added. In lEC, we supposed cells with large, unimodal fields. We characterized our network two fold by measuring its learning abilities by percent correct localization performance before and after learning, and by the number of resulting place-fields. We showed that learning was important in both increasing the performance of the network and decreasing the number of resulting place-fields. Inputs from mEC were found to increase percent correct localization both before and after learning. Simulated lesioning of DG decreased post training percent correct localization of the network, an effect that was found to be reversed by considering more mEC inputs. DG lesions, however, inevitably decreased the ability of the network to set up independent representations for correlated environments.

# Abstract: PERCEPTION OF OBJECT UNITY IN FISH

**Authors:** V. A. Sovrano, A. Bisazza

**Affiliations:** Department of General Psychology, University of Padua, Padua, Italy

Fish (*Xenotoca eiseni*) were trained to discriminate between a complete and an amputated disk. Thereafter, the fish performed test trials in which hexagonal polygons were either exactly juxtaposed or only placed close to the missing sectors of the disk in order to produce or not produce the impression (to a human observer) of an occlusion of the missing sectors of the disk by the polygon. In another experiment, fish were first trained to discriminate between hexagonal polygons that were either exactly juxtaposed or only placed close to the missing sectors of a disk, and then tested for choice between a complete and an amputated disk. In both experiments fish behaved as if they were experiencing visual completion of the partly occluded stimuli. These findings suggest that the ability to visually complete partly occluded objects may be widespread among vertebrates, possibly inherited in mammals, birds and fish from early vertebrate ancestors.

## Figures and Tables

**Figure 1:** Subicular PC response to size change and barrier insertion. Unit a scaled its firing field across all arenas. Unit b replicated its firing in the four rooms across repeated conditions.

# Abstract: SCALING AND MODULATION OF SUBICULAR PLACE CELLS FIRING FIELDS BY SPATIAL STRUCTURE

**Authors:** J. R. Brotons-Mas, N. Montejo, T. Gener, S. M. O'Mara, M. V. Sanchez-Vives

**Affiliations:** ^1^Instituto de Neurociencias de Alicante, Universidad Miguel Hernández-CSIC, San Juan de Alicante, Spain; ^2^Department of Psychology, Institute of Neuroscience, Trinity College Dublin, Ireland; ^3^LORIA-Campus Scientifique, Bâtiment C-BP 239-54506 Vandoeuvre-lès-Nancy Cedex, France

Studies on subicular place cells (SPC) have shown that these neurons could extrapolate spatial representation across different arena shapes and sizes possibly by using arena boundaries to set their firing [1, 2] Similarly, a model developed for hippocampal PC firing proposes that PC could be modulated by boundary vector cells (BVC) set to fire at a certain distance and angle bearing to the arena boundaries behaving in a similar way as subicular PC [3]. Although some characteristics of SPCs are known, it is not clear how they code for other extreme changes in size or systematic barrier insertion. Here we recorded from SPC in multiple arena size: small (50 × 50 cm), medium (100 × 100 cm) and large arenas (150 × 150 cm). Later, the medium arena open field was transformed in a four equal size communicated subchambers by inserting 4 barriers. Out of the 129 cells recorded in the size experiment 4 different response patterns were identified: Perfect scaling units (54%), which firing field location and size were equivalent across conditions. Imperfect scaling units (4%), similarly located but different relative size. Remapping scaling units (26%) scaled their firing fields but remapped across conditions. Size sensitive units (16%) presented firing only in some arenas. With respect to the 65 recorded units in the barrier-multiple chamber room, 92% replicated their firing at least in 2 chambers, 5% displayed plasticity across all 4 rooms and 3% stopped firing in the 4-room arena. Results suggest that SPC can scale up and down their firing fields from small to quite large arenas and that barrier insertion generates similar firing structure in different similar sub-chambers. The subiculum could be mainly coding for geometry and size of the spatial context, similarly to the BVC proposed for hippocampal neurons.

## References

[1] Sharp PE (1999). Subicular place cells expand or contract their spatial firing pattern to fit the size of the environment
in an open field but not in the presence of barriers: comparison with hippocampal place cells. *Behavioral Neuroscience*.

[2] Sharp PE (1997). Subicular cells generate similar spatial firing patterns in two geometrically and visually
distinctive environments: comparison with hippocampal place cells. *Behavioural Brain Research*.

[3] Barry C, Lever C, Hayman R (2006). The boundary vector cell model of place cell firing and spatial memory. *Reviews in the Neurosciences*.

# Abstract: NETWORK DYNAMICS OF INFEROTEMPORAL CORTEX DURING CATEGORICAL PROCESSING OF CONTINUOUSLY MORPHED NATURAL IMAGES

**Authors:** A. Akrami, Y. Liu, A. Treves, B. Jagadeesh

**Affiliations:** ^1^Cognitive Neuroscience, SISSA, Trieste, Italy; ^2^Department of Physiology and Biophysics, University of Washington, Seattle, USA

Neural codes for familiar visual stimuli may reflect distinct attractor states, possibly implemented in the dynamics of inferior temporal (IT) cortical networks, thought to be the long-term memory store for complex visual representations. An attractor network is endowed with the ability to converge towards pre-established representations, usually the one most closely correlated with some aspects of the inputs. To determine whether single units in the IT cortex of a behaving monkey exhibit such convergence, we recorded their responses during a classification task on visual stimuli prepared by gradually morphing between pairs of familiar images, one of which was chosen to elicit a particularly strong response (the effective image, while the other was simply less effective). Individual units show a variety of non-linear responses to successive morphs. On average, however, the initial response reflects the distance along the morphing dimension, the “physical changes”, while later, after stimulus offset, the responses elicited by the effective image and by its neighboring morphs were observed to converge as if only the natural category determined the firing level, for effective morphs. Such evolution of the response could reflect internal network dynamics in IT, perhaps underlying classification performance, induced either by attractor states or by firing rate adaptation or both. Attractor neural network simulations seem to discard a potential contribution of firing rate adaptation, and point at the removal of afferent inputs as the trigger for dynamical network convergence.

# Abstract: DO SPATIAL CONSTRAINTS INFLUENCE SEARCH BEHAVIOR IN THE PRESENCE OF CEREBELLAR LESIONS?

**Authors:** F. Foti, D. Cutuli, L. Mandolesi, P. De Bartolo, L. Petrosini

**Affiliations:** ^1^Department of Psychology, University of Rome “La Sapienza”, Rome, Italy; ^2^IRCCS Fondazione Santa Lucia, Rome, Italy; ^3^Faculty of Motor Sciences, University of Naples “Parthenope”, Naples, Italy

Recently, within the topic of spatial functions, an increasing interest is arisen in the analysis of search strategies in the presence of environmental constrains. This aspect was widely investigated in many spatial tasks, such as the radial maze, in which search behavior is constrained by the intrinsic structure of the apparatus. Conversely, the studies of unconstrained explorative behavior allow examining how search strategies modify according to environment features. The present study was aimed to investigate how search behavior could be modified in different spatial configurations in the presence of cerebellar lesions provoking marked exploration deficits. Namely, many studies demonstrated that cerebellar networks are specifically involved in the acquisition of the procedural components of different spatial tasks. Hemicerebellectomized (HCbed) and control rats were tested in a search task in which they had to visit nine food trays spatially arranged in four independent configurations: a cross, a 3 × 3 matrix, three clusters of three trays each and a circle configuration. The aim of the task was to catch the nine rewards avoiding visits to already depleted trays. When the effects of the four configurations were taken into account, it appeared that in the cross configuration both groups exhibited the lowest number of errors as well as a delay in performing the first error. Although no exhaustive searches were observed in any configuration, in all animals performances improved, as the sessions went by. However, HCbed rats exhibited lower search performances, displaying a significantly higher number of errors, long pathways to visit the trays, earlier occurrence of first error, reduced sequence of correct visits. The present data support the role of cerebellum in the acquisition of novel exploration strategies. Furthermore even in the presence of a cerebellar lesion, structural affordances of the environment appear to influence the construction of search strategies.

# Abstract: HIPPOCAMPAL ACTIVITY IN RATS DURING TACTILE DISCRIMINATION BEHAVIORS

**Authors:** P. M. Itskov, M. E. Diamond

**Affiliations:** Cognitive Neuroscience, SISSA, Trieste, Italy

Rats and mice palpate objects with their whiskers to generate tactile sensations. The present work is aimed at understanding the nature of the representation of the texture-related signals in the hippocampus. We recorded neuronal activity from the CA1 region of hippocampus while rats used their whiskers to discriminate between a rough and a smooth texture. We have found the cells in the hippocampus respond selectively to different aspects of the task, for example, touch, reward delivery. Some cells showed robust discrimination between 2 different textures, for example, their firing rate was significantly modulated during the contact with the texture or immediately after the contact. We then checked the texture related activity in a second task where rats had to discriminate between 3 different textures but they had to give the same response for 2 textures, that is, they had to identify all 3 textures and classify two of them as belonging to the same category. A small proportion (about 5% of recorded cells) showed different responsemagnitude for 2 textures that were in the same response class, while most of the cells (about 25% of all recorded cells) showed response levels specific for the texture class rather than for the individual texture identity. We speculate that a minority of cells in the hippocampus represent tactile information and while the majority of cells tend to represent the actual meaning of the texture or response class rather than the exact texture properties.

# Abstract: HIPPOCAMPAL MOSSY FIBRE DISTRIBUTION CORRELATES WITH MIGRATORY DISTANCE WITHIN AND ACROSS BAT SPECIES

**Authors:** I. Amrein, D. Dechmann, I. Drescher, H. Lipp

**Affiliations:** Institute of Anatomy, University of Zürich-Irchel, Switzerland

Variations in the extent of the terminal fields of the hippocampal mossy fiber projection have been shown to correlate with the animals reactivity towards external and internal (motivational) stimuli. The hypothesis predicts large intra- and infrapyramidal mossy fibers for animals in need of stable ongoing behaviour in spite of interfering stimuli, as we would expect for long distance migratory animals. To test this, we analyzed bats with differences in habitat shift or true migratory behaviour. For this, the mossy fiber fields were analyzed within and among bat species using the ratio of intra and infrapyramidal mossy fibres (IIP-MF) to suprapyramidal mossy fibres (SP-MF). Bats (9 wild-living species) have significantly smaller IIP- projections than do mice. In bats, the species with a maximum migration distance of 1440 km (Vespertilio murinus, particoloured bat) has significantly larger IIP-MF than do all other bats and shows an IIP/SP ratio within the corresponding range in mice. In bats, Spearman Rank correlation shows a positive association of the ratio of IIP/SP mossy fibres to habitat shift or migratory distance (*p* < 0.001, Rho 0.531). These results are strengthened by the observation that subadult, hence migratory naïve Vespertilio murinus show significantly smaller mossy fibers in the hilus and IIP projection field than adults. None of the other species show MF growth in connection to age and experience. These results, however, have to be re-evaluated, as Vespertilio murinus is the only long-distance migratory species in this sample. Hence, our prediction of a correlation between MF size and reactivity measured as migratory behaviour has been confirmed within the order of Chiroptera. Future research will integrate other mammalian orders and ecological factors such as social system, and will aim to include more species.

# Abstract: THE LONG-LASTING EFFECT OF SOCIAL ISOLATION AND SUBCHRONIC KETAMINE TREATMENT ON PAIN THRESHOLD

**Authors:** G. Tuboly, G. Horvath, G. Benedek

**Affiliations:** Department of Physiology, Faculty of Medicine, University of Szeged, Szeged, Hungary

Clinical studies have proved that schizophrenia is accompanied by hypoalgesia. Relevant animal models are of decisive importance in the study of psychiatric diseases. It is well known that subchronic treatment with ketamine or social isolation induces schizophrenia related alterations, which are ameliorated by clinically used neuroleptics. The purpose of this study was to test nociceptive responses after singly housing conditions and/or subchronic ketamine treatment.

At weaning (21–23 days) male Wistar rats were either group housed (5–6; GR. I and II) or isolated (GR. III and IV) for 20 days. Starting 6 days later (30 days old rats), animals (Gr. II and IV) were injected with 30 mg/kg ketamine daily for 14 days. Control rats (Gr. I. and III.) received saline. The tail-flick latency tests were performed at 21st, 35th and 42nd days at 48 and 52 °C.

The tail-flick latency significantly increased during the time in all groups and at both temperatures. Both at 21st and 42nd days applying 48 °C we found that housing condition significantly increased TF latency, whereas ketamine treatment had no effect on reaction time. The effect of ketamine + single housing condition did not differ from the saline + single housing conditions. There were no significant differences between the groups at 35th day. In contrast, at 52 °C there were no significant differences between the four groups at any time points.

Our study suggests that isolation but not ketamine treatment has effect on acute heat pain sensitivity. Since the low temperature activates mainly the C-fiber, while the high temperature the A*δ*-fibers, we suppose that this treatment disturbs primarily the C-fiber linked pain pathways.

# Abstract: A POSSIBLE INVOLVEMENT OF CEREBELLAR LEPTIN RECEPTOR IN THE BEHAVIOR OF PUBERTAL RATS

**Authors:** C. Koros, G. Boukouvalas, E. Kitraki

**Affiliations:** Lab of Histology, Athens University Medical School, Athens, Greece

Leptin is considered to play a fundamental role in various CNS functions, beyond the control of metabolism. Its receptor is widely expressed in the cerebellum. This implies a role of leptin in regulating cerebellum mediated tasks, including locomotion and spatial learning. Imbalanced diets are known to alter leptin levels in the periphery. In this study, we examined two experimental groups of 6-week old male Wistar rats, which followed a different dietary protocol from weaning to puberty. The high fat (HF) fed group received a diet enriched (45% kcal) in saturated fat and the low fat (LF) fed group an isocaloric low fat (10%kcal) diet. Animals were tested in the open field and Morris water maze tests. After sacrifice, we evaluated the expression of leptin receptor in the cerebellum by western blotting. Open field test revealed a less active spontaneous motor activity of the HF group, since the moving/standing ratio was decreased compared to the LF group in a statistically significant way. In learning the water maze task, HF group had lower latencies to locate the hidden escape platform, compared to LF animals. This might imply an enhanced spatial learning and navigation ability for the HF rats. Swimming velocities did not differ between the groups. Leptin receptors were statistically reduced in the HF group, possibly reflecting a down regulation shift due to the increased circulating leptin. Our results support an involvement of leptin signaling in the cerebellum, in behavioral aspects including locomotion and spatial learning. Although a role of leptin in regulating the aforementioned behaviors has been documented for the limbic system, cerebellum remains a strong candidate target for the non-metabolic actions of leptin in the CNS.

# Abstract: Theme B: Learning & memory (animal)  RAT NAVIGATION TO A VISIBLE MOVING GOAL IN MORRIS WATER MAZE

**Authors:** D. Klement, K. Blahna

**Affiliations:** Neurophysiology of Memory and Computational Neuroscience, Institute of Physiology, Academy of Sciences, Prague, Czech Republic

We studied navigation of rats (*n* = 7) to a visible moving goal in a circular water maze (190 cm in diameter). The goal was moving along the wall of the maze. The direction of the movement (clockwise, counter-clockwise) and the speed (slow, fast) changed pseudorandomly between trials but they remained constant within trials to make it possible to predict future locations of the goal. The fast speed of the goal was faster than the maximal swimming speed of the rats. We analyzed 45 slow trials (the goal moved slowly) and 33 fast trials (the goal moved fast) of trained rats. The rats used different goal-approaching strategies. In the case of the slow trials, the rats most often (62%) followed the goal until they reached it. In the remaining slow trials and in all the fast trials, the rats swum ahead of the goal motion. Some of these trajectories (slow trials: 61%, fast trials: 49%) were straight suggesting that the rats predicted the goal movement and estimated the place of collision.

# Abstract: REPREZENTATION OF MOVING OBJECT IN DIFFERENT CHARACTER OF ENVIRONMENT IN RAT

**Authors:** B. Karel, O. Barbora, K. Daniel

**Affiliations:** ^1^Department of Neurophysiology of memory and computation neuroscience, Academy of Science of Czech Republic, Prague, Czech Republic; ^2^Department of Zoology, Faculty of Science, Charles University, Prague, Czech Republic

We studied processing of spatial information represented by distant moving object in rat. For study, we used handled Long-Evans rats (*n* = 10). Animals were trained in two rings circular arena. The inner ring of arena was full circle (*d* = 0.82 m), it was a space where rats were tested inside, external ring was circular belt (length 0.4 m), where object (black cylinder: latitude = 0.4 m, high = 0.8 m) were offered. Both inner and external circles were separated by transfluent plexiglass wall (high = 60 cm), both arena rings were movable in clock-wise or counter clock-wise directions, at speeds of 1 turn per minute. Animals were trained avoid of shock sector (60°) denoted by object moving in clock-wise direction. All parameters of experiment was conformable to AAPA test [1] where rats avoided of similar sector denoted by room or arena frame.

In first part of experiment rats were trained to avoid moving sector inside the stable arena designated by object moving with the external ring. Number of entrances (Figure 1) was **30.4** (SEM = 1.3) in day 1. Second day animals significantly decreased number of entrances to **21.7** (SEM = 1.3, *p* = 0.014), this behavior remain stable in all experimental days. In day 8 was object removed and rats avoided only moving sector alone. Generally we didn't found no change of ability to avoid the sector with or without denoted of object, number of entrances **23.7** didn't changed (SEM = 1.4, *p* = 0.71 for day 7 to day 8). Next experimental day 9 object were returned again to the external ring. On last experimental day 10 were character of arena movement changed. Moving object became stable in room and in opposite, inner arena with rat had been rotated. Behavior notably changed, number of entrances statistically decreased in comparison to all days to **10.1** (SEM = 2.7, *p* = 0.002 for day 1 to day 10, *p* = 0.004 for day 7 to day 10). We also found statistically significant differences in total path length (Figure 2) between last 10 to all days (*p* = 0.008 for day 1 to day 10, *p* = 0.00001 for day 7 to day 10).

Our preliminary study had shown that processing information of distal moving object is for stable room frame in rat more difficult than processing of similar information among object which is stable in room frame and arena with rat which is moving. Next step is found importance of this kind of landmark in more cue controlled experiment.

## Figures and Tables

**Figure 1:** Number of entrances to avoided sector during ten experimental days. Themain differences are between 1-th a 2-th days and between 10-th and first days, when character of moving arena had changed.

**Figure 2:** Total length of trajectory path. Note difference between 10-th day when character of moving arena had changed and remain experimental days.

## References

[1] Bureš J, Fenton AA, Kaminsky Y, Rossier J, Sacchetti B, Zinyuk L (1997). Dissociation of exteroceptive and idiothetic orientation cues: effect on hippocampal place cells and place navigation. *Philosophical Transactions of the Royal Society of London Series B*.

# Abstract: BEHAVIORAL EFFECTS OF 6 MONTH ORAL ALUMINUM EXPOSURE AT ONE YEAR OF AGE IN A APP695 TRANSGENIC MICE

**Authors:** D. Ribes, P. Vicens, J. Domingo, M. Colomina

**Affiliations:** ^1^Department of Psychology, “Rovira i Virgili” University, Tarragona, Spain; ^2^Laboratory of Toxicology and Environmental Health, “Rovira i Virgili” University, Reus, Spain

In rodents, the studies on neurotoxicity and neurobehavior of oral aluminum (Al) have not provided yet enough evidence to establish if this metal is a potential risk factor for neurodegenerative disorders. Because mice and rats are not especially sensitive to Al, in the present study we investigated the neurobehavioral effects of Al in a transgenic mouse model of Alzheimer disease. Transgenic 2576 mice and their respective wild controls were fed with rodent chow supplemented with Al lactate at 0 or 11 g/kg during six months. At one year of age, several tests were carried out. A functional observational battery showed differences between groups in the arousal and hand reaction. Results in openfield showed a general effect of Al in the time spent in the center of the open. Moreover, Al exposed mice were more active in the center of an open field when compared with non exposed mice. The two compartment light/dark test, showed general effects of animal type on latency to the light compartment and time spent in the dark compartment, tg mice spent more time in the dark compartment and showed a higher latency to enter the light compartment. Moreover a general effect of Al was observed in time spent in the dark and in the number of entries to the light compartment. Al exposure tens to increase activity in both transgenic and wild mice, this increase is higher in transgenic mice. Results in water maze test showed a general effect of animal type in acquisition, and an interaction between Al treatment and animal type. Differences between groups were observed during the whole acquisition period. In general terms aluminum treatment improve acquisition in wild mice and impair acquisition in transgenic treated mice. However, retention was neither affected by animal type nor by aluminum exposure.

# Abstract: ADULT HIPPOCAMPAL NEUROGENESIS IN ST8SIAIV/P- ST-KNOCKOUT MICE

**Authors:** M. Zerwas, T. Escudé, H. Halley, R. Gerardy-Schahn, C. Rampon

**Affiliations:** ^1^Centre de Recherches sur la Cognition Animale, CNRS UMR 5169, Toulouse, France; ^2^Abteilung Zelluläre Chemie, Medizinische Hochschule Hannover, Germany

Adult hippocampal neurogenesis is now a well-established fact in the mammalian brain. Although the steps from the generation of new cells to their integration into the existing network are gradually being unveiled on the cellular level, their functional role is still controversially being discussed. However, evidence is emerging that adult newborn cells play a role in some types of learning and memory processes, a prominent example being hippocampally-dependent spatial memory. The carbohydrate polysialic acid (PSA), uniquely associated with the neural cell adhesion molecule NCAM, is predominantly found in the developing brain. Nevertheless, its expression persists in brain regions of plasticity, such as the hippocampus, during adulthood. Here, young newborn neurons display PSA-NCAM. Furthermore, levels of PSANCAM are transiently increased following hippocampally-dependent learning. This strongly suggests the implication of PSA in both adult neurogenesis and cognitive processes. Moreover, knockout (ko) mice lacking the enzyme polysialyl transferase ST8SiaIV/PST and hence practically devoid of PSA in adulthood, have recently been shown to be impaired in the morris water maze for reversal learning, a test for hippocampally-dependent spatial memory. We examined the hippocampal neurogenic potential in 6 monthold male ST8SiaIV/PST-ko mice. At this age the amount of PSA-NCAM is reduced by 75% in mutant mice compared to controls. Cell proliferation in the hippocampus was investigated using the cell cycle marker Ki67. With doublecortin, a marker of immature neurons, the window of the impact of PSA lack was further confined. Using BrdU, we determined the survival rate of 30 day-old newborn cells. Stereological analysis of these immunostainings allowed us to detect differences in hippocampal neurogenesis in ST8SiaIV/PST-ko mice compared to their wild type littermates.

# Abstract: ARE NEW HIPPOCAMPAL NEURONS INVOLVED IN THE RETRIEVAL OF LONG-TERM MEMORY IN THE ADULT MOUSE?

**Authors:** S. Trouche, C. Guissard, P. Roullet, B. Bontempi, C. Rampon

**Affiliations:** ^1^Centre de Recherches sur la Cognition Animale, UMR 5169, Toulouse, France; ^2^Centre de Neurosciences Intégratives et Cognitives, UMR 5228, Bordeaux, France

The finding of neurogenesis in the adult hippocampus has favored the idea that this continuous neurogenic potential might subserve cognitive functions. Studies have shown that spatial learning, which heavily relies on the hippocampus, can enhance the survival of newborn neurons. Conversely, reduction of neurogenesis by irradiation impairs long-term memory retrieval but not acquisition. However, the question remains as to whether new neurons born before learning may be recruited into dentate gyrus circuits by participating in long-term spatial memory retrieval. Adult C57BL/6J mice were injected over 1 day with bromodeoxyuridine (BrdU, 3 × 100 mg/kg, ip) and were trained one week later in the Morris water maze (MWM). Training consisted in 24 trials over 1 day (fixed platform location, various starting points). BrdU-labelled cells were 9 days-old at the time of training, an age when these cells exhibit functional plasticity. A single probe test was given 30 days after training. To isolate nonspecific aspects of the procedure, control groups included “swimmers”, yoked-control mice, “cue-learners” trained in a hippocampus-independent version of the task and “homecage controls”. Mice were sacrificed 2 hours after the probe test and immunohistochemistry of the activity-dependent gene zif268 was used to quantify neurons processing spatial memory during retention testing. Phenotype of the surviving BrdU cells was established using NeuN, a marker of mature neurons. Our results show that survival of newborn neurons is increased by spatial learning compared to swimmers. A significant proportion of new neurons expressed Zif268 following long-term spatial or cue memory retrieval. Our data suggest an early functional recruitment of newborn neurons into neuronal networks involved in long-term spatial memory retrieval.

# Abstract: BEHAVIORAL EFFECTS OF ADULT MICE EXPOSED TO PERFLUOROOCTANE SULFONATE (PFOS)

**Authors:** S. Fuentes, M. Colomina, P. Vicens, J. Domingo

**Affiliations:** ^1^Department of Psychology, “Rovira i Virgili” University, Tarragona, Spain; ^2^Laboratory of Toxicology and Environmental Health, “Rovira i Virgili” University, Reus, Spain

Perfluorooctane sulfonate (PFOS) is an organic pollutant with wide consumer and industrial applications. Recent findings of its global distribution, environmental persistence, presence in humans and wildlife and adverse health effects in laboratory animal studies have generated considerable interest in this compound. Although numerous human and wildlife biomonitoring studies have been conducted, not much is known about its behavioral effects. Therefore, in the present work we assessed the effects of PFOS on behavior after one month of exposure. Thirty adult mice were divided in three groups (*n* = 10). Animals were dosed by gavage 0, 3 or 6 mg PFOS/kg/day for four weeks. After the treatment period, mice were evaluated for several skills by testing motor and sensory function with a functional observation battery (FOB), general activity and exploratory behavior in an openfield and learning and memory in a water maze task. One week after behavioral testing, all animals were deeply anesthetized and blood from cava vein collected and centrifuged in order to obtain serum for corticosterone analysis by RIA. No general effects were observed in the FOB. Activity in the open-field was similar in all the groups, the only observed differences were: group PFOS 3 spent less time in the center and group PFOS 6 performed a reduced rate of vertical activity. Concerning the effects of PFOS in the water maze, all animals learned the task, but no effect of group was observed during the acquisition. In the retention test, Control group presented a reduced swim velocity within the target quadrant, which may indicate that they did recognize the target location. These results suggest that PFOS exposure induce behavioral effects in adult male mice.

# Abstract: SPATIAL LEARNING DURING THE NEONATAL PERIOD USING THE MOTHER AS A POSITIVE OR NEGATIVE REINFORCER

**Authors:** M. Dimitropoulou, A. Stamatakis, T. Panagiotaropoulos, F. Stylianopoulou

**Affiliations:** Laboratory of Biology-Biochemistry, University of Athens, Athens, Greece

During the neonatal period mother-infant interaction mediates all environmental stimulation of the pups. When mother contact is used as either a positive or a negative reinforcer, rat pups, both those denied contact (frustrated) and those allowed contact as continuous reward (non-frustrated), can learn a T-maze after a four day training (postnatal day 10–13). Although, both experimental groups acquire the spatial memory task, it appears that non-frustrated rats follow procedural learning processes. Frustrated pups, on the other hand, which show an increased activation of the hippocampus, employ a cognitive strategy that still remains elusive. In an effort to address this issue, we subjected them to two different memory probe trials where the mother was either absent, or placed in the reverse position to that during training. In the absence of the mother animals spent most time in the start position of the T-maze, while in the reverse probe trial they stayed the longest in the armof the maze that led to their mother. However detailed analysis of the path followed by the pups revealed that most of them initially turned towards the arm where their mother was during the training sessions (“target arm”), indicating that they had acquired the spatial information. Furthermore, a positive correlation was found between performance during learning and time spent in the “target arm”. The above results show that, in spite of the frustrative non-reward training, animals learn the T-maze, but, at the same time, the presence of the mother provides the motivation essential for the execution of the motor program of approach by the pups.

# Abstract: MAMMILLARY BODY PROJECTIONS TO THE ANTERIOR THALAMIC NUCLEI ARE NECESSARY FOR NORMAL IMMEDIATE-EARLY GENE EXPRESSION IN RETROSPLENIAL CORTEX

**Authors:** M. M. Albasser, S. D. Vann

**Affiliations:** Cardiff University, UK

The mammillary bodies (MBs) are centrally placed within the Delay and Brion circuit, receiving dense afferents from the hippocampal formation, via the fornix, and projecting in turn to the anterior thalamic nuclei, by way of the mammillothalamic tract (MTT). Lesions of the MTT produce persistent deficits on spatial memory tasks comparable with those seen after MB lesions. While memory impairments found after MTT lesions are assumed to be due to the disruption of the MB projections to the anterior thalamus, it is not known whether disconnecting these structures may have wider effects on more distal brain regions. In the present study, the quantification of immediate-early gene (IEG) expression was used as a marker of neuronal activity in animals that had received either bilateral lesions of the MTT or sham surgery. Rats were trained on a forced-choice version of the radialarm maze task but on the final test day rats performed the task in a novel room in order to increase demands on spatial encoding. MTT lesions markedly reduced IEG expression in the retrosplenial cortex, another component of the Delay and Brion circuit, and a structure also implicated in spatial memory. The present results are consistent with previous studies as reduced retrosplenial IEG expression has also been reported following lesions of both the hippocampus and anterior thalamic nuclei. However, while the hippocampus and anterior thalamus both have dense, direct connections with the retrosplenial cortex, the MTT lesion effects are indirect and are, therefore, even more striking. Together, these results highlight both the importance of the integrity of the Delay and Brion circuit for normal retrosplenial activity and the need to interpret MTT lesion effects not solely in terms of the mammillary body-anterior thalamic disconnection.

# Abstract: ENCODING OF THE TOPOLOGY OF SPACE BY HIPPOCAMPAL PLACE CELLS

**Authors:** A. Alvernhe, T. Van Cauter, E. Save, B. Poucet

**Affiliations:** Laboratoire de Neurobiologie de la Cognition, Université de Provence/CNRS, Marseille, France

Hippocampal place cells are pyramidal cells that display location-specific activity, that is, fire when the animal is in a specific location in the environment (called the place field of the cell). A remarkable property of place cells is their sensitivity to environmental modifications. For example, changing the shape or color of the apparatus induces a reorganization of place fields, a phenomenon called remapping.Here, we examine how CA1 place cells respond to a topological change in the environment. Place cell activity was recorded while rats alternated between two food-rewarded goals in an Mmaze. Following an initial recording session, the topology of the maze was modified by removing an inner wall, therefore generating a shortcut. The results show that place cells that had fields close to the removed wall remapped (i.e., changed their location, shape, or firing rate) whereas most place cells that had fields at a distance from the removed wall remained stable. These results are compatible with the notions of local (restricted to the changed region) and partial (only a part of the place cell population is affected) remapping. Such remappings can be interpreted in the light of the attractor network theory of hippocampal function, in which the connexions between attractor networks generated during learning reflect the time sequence of visited regions, and therefore the topology of space.

# Abstract: FROM PILOTING TO MAP NAVIGATION- A PROPOSED EXPERIMENTAL MODEL OF SPATIAL REPRESENTATION

**Authors:** R. Avni, D. Eilam

**Affiliations:** Dept. Zoology, Tel-Aviv University, Israel

We present a hierarchal model and supporting data on spatial representation during exploration and navigation, based on successive phases of spatial information processing: 1) Piloting (Sequential processing): egocentric space representation is constructed by traveling from one landmark to the next, changing (reprocessing) heading direction between travel segments; 2) Orienting (Parallel processing): space is represented in relation to a fixed place that is a terminal for repeated sequential processing that varies, yet starts and ends at that location, thus heading direction ultimately points to the fixed location; 3) Navigating (Continuous processing): space representation (“map”) is constructed by orienting (continuously updating heading direction) in relation to several places (landmarks). Each phase is based on spatial information gained in previous phases, thus reflecting a progress to a higher level of information processing. Empirically, we so far demonstrated a transition from sequential to parallel processing in the exploratory behavior of gerbils. When tested in an unfamiliar dark environment, gerbils first explored the open field using a looping mechanism: traveling slowly in an undulating path that forms a loop by returning to a previously visited place. In looping, the gerbils rarely returned more than once to a previously visited place, with no apparently fixed heading direction, as if behavior is not anchored to the environment. Rather, they seem to shift from one location (landmark) to the next. Gradually, the gerbils shifted from looping to a home base mechanism, spending increasingly greater time in one place and repeatedly returning to it. Exploration became organized in relation to a fixed place, with varying paths that share a heading direction computed in relation to the same place (the home base). We suggest this as a shift from piloting (looping) to orienting (home base behavior), reflecting a change from momentary to fixed heading direction.

# Abstract: THE ROLE OF GLUTAMATE RECEPTOR SUBTYPES WITHIN THE PREFRONTAL AND PERIRHINAL CORTICES IN ASSOCIATIVE RECOGNITION MEMORY

**Authors:** G. R. Barker, E. C. Warburton

**Affiliations:** MRC Centre of Synaptic plasticity, Department of Anatomy, University of Bristol, Bristol, UK

Experimental evidence has suggested that the perirhinal cortex (PRH) and the medial prefrontal cortex (mPFC) play a crucial role in recognition memory based upon in objectplace associative discriminations, and that these two cortical regions operate within a functional neural network (Barker et al. 2007). The present study sought to examine the importance of glutamatergic and cholinergic receptor neurotransmission in these cortical regions for performance of an object-in-place discrimination task. Selective AMPA/kainite, NMDA or muscarinic receptor antagonists were infused into the PRH or mPFC via chronically implanted via bilateral guide cannulae prior to acquisition in an object-in-place task. The behavioral effects of such infusions were compared with the effects of unilateral infusions into both PRH and mPFC in either the same or contralateral hemispheres. Infusions of CNQX (an AMPA/kainate receptor antagonist) into the PRH, mPFC or both regions in contralateral hemispheres impaired short-term object-in place performance. Infusions of scopolamine (a muscarinic receptor antagonist) into the mPFC, PRH or both regions in contralateral hemispheres impaired short-term and long-term object in place performance. Infusions of AP5 (an NMDA receptor antagonist) into the mPFC impaired long-term and shortterm object-in-place memory, while infusions of AP5 into the PRH, or into both the PRH and mPFC in contralateral hemispheres impaired short-term but not long term objectin place-memory. These results indicate the importance of both glutamatergic and cholinergic neurotransmission for discriminations based upon object-place associations. Further these results support the hypothesis that the PRH and mPFC interact within the same hemisphere to process associational recognition memory information.

# Abstract: THE INVOLVEMENT OF CORTICAL M1 CHOLINERGIC RECEPTORS IN VISUAL RECOGNITION AND PERCEPTION

**Authors:** S. J. Bartko, B. D. Winters, L. M. Saksida, T. J. Bussey

**Affiliations:** Experimental Psychology, University of Cambridge UK

Recent studies have indicated the involvement of muscarinic cholinergic receptors in the perirhinal cortex (PRh) in object recognition memory. However, the specific role of muscarinic receptor subtypes in object recognition and complex perceptual processing remains unclear. In the present study we examined the specific involvement of m1 cholinergic receptors in PRh in spontaneous object recognition memory, as well as a perceptual task—simultaneous oddity discrimination. In the first experiment, rats received counterbalanced intra-PRh infusions of either saline or the m1 receptor antagonist pirenzepine 15 minutes before a given object recognition trial. The retention delay between sample and choice phases was either 0-s or 3 hr. Pre-sample infusions of pirenzepine significantly impaired object recognition memory with a 3-hr retention delay compared to the same rats on saline trials. Pirenzepine did not, however, cause an impairment when a 0-s retention delay was utilized. In the second experiment, we examined whether pirenzepine would affect performance in an oddity discrimination task in which all objects were presented simultaneously; the perceptual similarity of the stimuli varied according to three different conditions (easy, medium, and hard). A new group of rats received pre-trial intra-PRh infusions of pirenzepine or saline. Pirenzepine significantly impaired oddity discrimination performance compared to saline in the hard condition, but not in the easy and medium conditions. This result suggests that difficult perceptual tasks require m1 receptors in PRh. Collectively, these findings suggest an important role for m1 receptors in PRh for memory acquisition and for the perceptual processing of complex stimuli. The delay- and perceptual difficulty-dependant nature of the pirenzepine-induced deficits suggests thatm1 receptor-mediated information processing in PRh is critical for demanding mnemonic and perceptual functions.

# Abstract: TRANSITIONS IN BEHAVIORALLY CORRELATED ACTIVITY IN MEDIAL PREFRONTAL NEURONS OF RATS ACQUIRING AND SWITCHING STRATEGIES IN A Y-MAZE

**Authors:** F. P. Battaglia, M. Khamassi, A. Peyrache, V. Douchamps, P. Tierney, S. Wiener

**Affiliations:** ^1^SILS-APCN Universiteit van Amsterdam, The Netherland; ^2^CNRS-College de France, France

In order to elucidate prefrontal mechanisms for cross-modal strategy shifts, PL/IL neurons were recorded in rats performing a Y maze task based on the Wisconsin Card Sorting Task used to test for prefrontal deficits in neurological patients. On each trial one of the arms was selected by random to be lit. However the rat was only rewarded at the arm on the right. Once the rat acquired this ‘go right’ strategy (ignoring lighting of the arms) it was then required to, only on the basis of absence or presence of reward, to shift strategies and go to the lit arm, whether it was to the left or right. (Once acquired this was followed by ‘go left’ and ‘go dark’ contingencies, etc.) Of the 1894 cells with mean firing rate greater than 0.3 Hz analysed in 5 rats in 108 recording sessions, 259 showed abrupt changes in activity profile during the course of a recording session. These changes included overall firing rate changes or appearance (or disappearance) of a behaviorally correlated activity. In 91 neurons, these transitions in cell activity corresponded to changes in task contingency imposed by the experimenters, while in 143 cases the activity changes accompanied shifts in behavioral strategy spontaneously made by the animal and 25 other neurons showed both effects. In previous recordings of rats switching strategies (albeit with trigger cues) [1] we observed comparable changes in responses in prefrontal afferent zones of the nucleus accumbens, but not hippocampal place cells which also project to accumbens [2]. This is consistent with the hypothesis that this striatal ‘set-shifting’ activity is mediated at least in part by prefrontal projections.

## References

[1] Shibata R, Mulder AB, Trullier O, Wiener SI (2001). Position sensitivity in phasically discharging nucleus accumbens neurons of rats alternating between
tasks requiring complementary types of spatial cues. *Neuroscience*.

[2] Trullier O, Shibata R, Mulder AB, Wiener SI (1999). Hippocampal neuronal position selectivity remains fixed to room cues only in rats alternating
between place navigation and beacon approach tasks. *European Journal of Neuroscience*.

# Abstract: BEHAVIORAL ANALYSIS OF CYCLIN D2 KNOCKOUT MICE, LACKING ADULT HIPPOCAMPAL NEUROGENESIS, WITH AND WITHOUT EXPOSURE TO CRANIAL IRRADIATION IN CLASSICAL PARADIGMS AND THE INTELLICAGE

**Authors:** N. Ben Abdallah, R. K. Filipkowski, P. Jaholkowski, L. Kaczmarek, H. P. Lipp

**Affiliations:** ^1^University of Zurich, Anatomy Institute, Zurich, Switzerland; ^2^Department of Molecular and Cellular Neurobiology, Nencki Institute, Warsaw, Poland

Low doses of X-rays are used to non-invasively analyze the function of neurogenesis in the adult rodent hippocampus. Previously, this approach showed a role of hippocampal neurogenesis in learning and memory, particularly with the Morris water-maze and fearconditioning. However, such behavioral impairment may arise from combined effects of neurogenesis and other radiation-induced brain changes. To address this question, we investigated the effects of a cranially focused low dose of radiation on behavioral performance. We used Cyclin D2 knockout mice, which lack adult hippocampal neurogenesis. Mice were exposed to 10 Gy of Xrays, a dose known to induce hippocampal-dependent cognitive and learning deficits. Classical behavioral tests were the Morris water-maze, open-field, and fear conditioning. We also monitored the behavior of the mice in the IntelliCage (NewBehavior, AG), an automated home cagelike device. Results were:

1. reduced activity of the cyclin D2 knockout mice, regardless of the radiation exposure, observed in the IntelliCage through the number of visits to the drinking corners during the first hour (*p* = 0.0007) as well as the whole session (*p* = 0.043) of a short adaptation phase
2. impaired long-term memory retention of the mice genetically lacking adult neurogenesis, regardless of whether or not they had received radiation, as evidenced by the Morris water maze (*p* < 0.0001).

Exposure to radiation significantly reduced long-term memory retention in WT-mice to levels similar to those of KO-mice. The later effect argues against claims that radiation effects are not specific to adult neurogenesis. These results show a similarity between cognitive changes induced by genetically and radiation-induced ablation of adult neurogenesis, suggesting a phenomenological specificity of the effects of radiation on hippocampal neurogenesis and performance in hippocampus-dependent behaviors.

# Abstract: C-FOS EXPRESSION IN MAMMILLARY BODIES AFTER LEARNING IN A SPATIAL WORKING MEMORY TASK WITH RATS

**Authors:** E. Blanco, R. Miranda, A. Begega, L. J. Santin, J. L. Arias

**Affiliations:** ^1^Faculty of Psychology, University of Oviedo, Oviedo, Spain; ^2^Department of Psychology, University of Balearic Islands, Palma de Mallorca, Spain; ^3^Faculty of Psychology, University of Malaga, Malaga, Spain

Different studies suggest that mammillary bodies contribute to learning and memory. There are some evidences that showing a great implication of this region in allocentric spatial processing by rats. Although the involvement of the mammillary bodies in memory processes has been shown, the relevance of these nuclei has remained poorly understood. In the present study, rats were trained on a spatial working memory task that could be solved by place strategy. Male and female rats performed a spatial orientation tasks in the holeboard, which permits assessment of working memory. The holeboard apparatus consists of an open-field with a 16-hole floor insert. Across trials, animals have to learn that 4 holes of 16 are baited. Configuration of baited holes was changed trial to trial. The aim of this study was to assess the effect of spatial training with working memory demands to solve the task, related to c-fos expression in the mammillary bodies. Rats readily acquired this task within 4 days at two trials per day (sample-retention), but no differences between sexes were found. One hour after training, the number of c-Fos positive neuronal nuclei was quantified in the mammillary body region (medial mammillary nucleus [MMn], lateral mammillary nucleus [LMn] and supramammillary nucleus [SuM]). The results shown that c-Fos immunoreactivity (IR) was significantly higher in the SuM of males and females, and in the MMn of males trained than in control rats. On the other hand, no sex differences were shown between males and females trained rats in c-Fos-IR. In conclusion, our results suggest that the regions studied by immunohistochemical analysis of the c-Fos protein play a different role in spatial working memory.

# Abstract: A NEURAL NETWORK MODEL FOR GRID CELLS: COHERENCE IN THE POPULATION DYNAMICS

**Authors:** E. Cerasti, A. Treves

**Affiliations:** SISSA, Triesta, Italy

An important feature of grid cells in mEC is that they present firing fields that remain coherent among themselves across changes in the environment, even if the individual firing fields change (Fyhn et al. 2007). Such coherence is expected if the triangular grid firing maps are taken, as postulated by recent models (Fuhs & Touretzky, 2006), to result from connectivity that reflects physical distance between units in mEC, perhaps during an initial phase of development (Mc- Naughton et al., 2006).

An alternative model (Treves et al., SfN 2005) for the formation of the grid pattern views it as produced by two factors: the need to represent continuous space and the effect of adaptation in firing rates. The spatial features of the grid are then determined by the time course of firing rate adaptation and by the mean locomotion speed; and they are not related to the connectivity pattern. We asked whether in this alternative model the grid pattern is indeed established and a coherent population dynamics also emerges.

In order to answer this question we performed simulations of a population of recurrently connected units; unfamiliar environments are explored and units receive input information about spatial features in the environments. A grid arrangement seems to emerge and the coherence across cells in each environment is found to be greater than the stability of each cell across different environments.

# Abstract: TRACKING THE ROOTS OF HUMAN MENTAL RETARATION: COGNITIVE IMPAIRMENTS IN GDI1 KNOCKOUT MICE ARE ASSOCIATED WITH ANOMALOUS SYNAPTIC VESICLES

**Authors:** V. Bianchi, H. Lipp, D. Toniolo, P. D'Adamo

**Affiliations:** ^1^Dulbecco Telethon Institute at DIBIT-HSR, Milan, Italy; ^2^Dept. Neuroanatomy and Behaviore, Zurich University, Zurich, Switzerland; ^3^DIBIT-HSR, Milan, Italy

GDI1, one of the genes responsible for human X-linked Mental Retardation, encodes alfaGdi, one of the proteins controlling the activity of the small GTPases of the Rab family, in intracellular vesicle trafficking. A mouse mutant deficient in Gdi1 was generated. The phenotype of the Gdi1 KO mice may be ascribed to alterations in a subset of Rab GTPases that were found concentrated on intracellular vesicle membranes, possibly in their GDP bound inactive form, and a reduced concentration in their soluble form. Electrophysiological analysis of the KO mice revealing that slow repetitive stimulation induced short-lasting impairments of synaptic transmission: a post-tetanic depression was observed in mutant mice suggesting depletion of the available SV pool. EM analysis was performed on P10, P23, and P90 hippocampus of Gdi1 KO mouse mice, to monitor SV density. SV counting revealed a significant reduction of the SV density in mutant mice in all stages analyzed. A detailed study of their learning and behaviour capacity showed a specific cognitive impairments detected in two tests: the 8-arms radial maze and the trace fear conditioning, both dependent on a functionally intact hippocampus and on the ability to form short-term memory. We showed that in the trace fear conditioning test, the Gdi1 KO mice were impaired in forming the association between CS and US if they were separated by a short time interval. When the ITI between each CS-US pairing was more than 30 minutes KO mice associate the two stimuli as well as the WT mice. In the 8-arms radial maze test, Gdi1 KO mice showed specific impairment in working memory formation, but they exhibit intact working memory when the arms were reduced to 4 or 6, and also when we pretrained them on 4- arms and then tested on 8-arms. It is likely that while mass training eventually caused depletion of the SV pool, during a longer ITI the KO mice can restore the SV pool required for efficient synaptic transmission and shortterm memory formation and processing. All these data suggest that lack of Gdi1 in the brain of KO mice alters steps controlling formation and maintenance of the pool of SV through intracellular trafficking possibly through Rab activity.

# Abstract: LATERALIZATION OF SOCIAL LEARNING IN THE DOMESTIC CHICK (*GALLUS GALLUS*): LEARNING TO AVOID

**Authors:** J. N. Daisley, O. Rosa-Salva, G. Vallortigara, L. Regolin

**Affiliations:** ^1^Department of General Psychology, University of Padova, Via Venezia 8, Padova, Trieste, Italy; ^2^Department of Psychology, University of Trieste, Via S. Anastasio, 12, Trieste, Italy

Social learning allows individuals to acquire information about their environment whilst reducing the risks associated with engaging in novel experiences. At least some social behaviours are influenced by cerebral lateralization [1]. In the present study we used the domestic chick (*GALLUS GALLUS*) as a model since light stimulation of eggs before hatching is known to produce brain lateralizations, and hence behavioural asymmetries, for several visual tasks in this bird [2].

We employed a one-trial passive avoidance learning task as a simple and quick tool to determine whether the ability of young domestic chicks to avoid pecking a noxious substance having seen a conspecific's reaction is influenced by brain lateralization. We placed 2-day old chicks, coming from eggs maintained in the light (“lateralized”) or in the dark (“nonlateralized”) with a conspecific social demonstrator (either of the same or different lateralized state) separated by a piece of wire mesh. One of a pair of chicks (the demonstrator) was allowed to peck a small red bead coated in a bitter-tasting substance, methylanthranilate (MeA). The other chick (the observer) was able to see the demonstrator's reaction but was not given the opportunity to peck the bead. 30 min later both demonstrator and observer were given the opportunity to peck a dry red bead followed by a white bead. Successful discrimination was determined by pecking at the white bead but avoiding the red bead. A control group received a dry red bead at both training and testing.

Initial results show that chicks in all groups could learn the avoidance task. It appears that lateralization state of both observer and demonstrator chicksmay interact in such a way as to suggest that differential social interactions are occurring between these birds.

## References

[1] Deng C, Rogers LJ (2002). Social recognition and approach in the chick: Lateralization and effect of visual experience. *Animal Behaviour*.

[2] Rogers LJ, Andrew RJ (2002). *Comparative Vertebrate Lateralization*.

# Abstract: BREAKING UP THE GRID CELL PATTERN IN THE HIRPIN MAZE

**Authors:** D. Derdikman, M. Fyhn, T. Hafting, A. Tsao, M. B. Moser, E. I. Moser

**Affiliations:** Centre for the Biology of Memory, Norwegian University of Science and Technology, 7489 Trondheim, Norway

During locomotion, mammals update their position with respect to a fixed point of reference by processing inertial cues, proprioceptive feedback and stored motor commands generated during locomotion. One question that arises is, what is the extent to which path integration and familiar visual cues cooperate to optimize navigational performance. The discovery of grid cells in the medial entorhinal cortex (Hafting et al., Nature, 2005) has opened a new field of research. Grid cells fire when the rat is running in a box, and is in any one of a number of locations that are arranged in a regular hexagonal grid.The grid cells are likely to be key components of a brain mechanism that constantly updates the rat's sense of its location, even in the absence of external sensory input. We recorded spike activity in grid cells in the entorhinal cortex and in the hippocampus while rats run for food in a large open field and in a hairpin-maze of the same dimensions. The hairpin maze was used to force the rat to cover the whole twodimensional box while performing a one-dimensional task. As expected, triangular grid fields emerged in the open field. In the hairpin maze, the grid structure broke up into repeating patterns dictated by the hairpin turns. Firing fields emerged at similar distances between the start and end of each linear segment of the maze. The results suggest that the boundary conditions of the grid may be set by the walls which restrict the path of the rat; thus, similar paths in different positions may evoke replicating grid-field representations. The observations imply that the grid-cell representation in the one-dimensional case is not simply a projection of a two-dimensional representation.

# Abstract: EFFECTS OF METRIC TRANSFORMATIONS OF AN ARRAY OF LOCAL LANDMARKS ON THE ORIENTATION STRATEGIES OF THE DOMESTIC CHICK

**Authors:** T. Pecchia, C. Castella, A. Della Chiesa, G. Vallortigara

**Affiliations:** ^1^Dept. of Psychology, University of Trieste, Italy; ^2^Inst. of Neuroscience, Trinity College, University of Dublin, Ireland; ^3^BRAIN Centre for Neuroscience, University of Trieste, Italy

Chicks can be trained to ground scratch over sawdust on the floor of an enclosure in order to gain a previously buried food reward. Using this method, it has been shown that chicks can retrieve the goal location by relying on the global geometry of the enclosure as well as on local landmarks. However, little is known on how chicks encode the spatial relationship between a goal and the local orienting cues. To investigate this issue, we performed two experiments. In the first experiment, chicks were trained to locate food in the centre of a square-shaped arena in the presence of two landmarks which were spaced 25 cm apart along one of the walls of the enclosure. After training, chicks were tested binocularly (BIN) and in monocular conditions (RE: right-eye; LE: left-eye) after enlarging or contracting the distance separating the landmarks. At test, BIN and LE still searched for food at the centre of the arena, irrespective of the array transformation. RE chicks relied more on local cues, moving toward the axis of the configuration when metric transformations occurred. In the second experiment, chicks were trained to locate food with respect to the landmarks only (i.e., food was located in different positions within the enclosure but in a fixed spatial relationship with respect to landmarks). LE-chicks were more scattered in searching behaviour when compared to the other two groups. BIN-and RE-chicks approached the array along its axis when contracted, whereas they approached closely the points defined by vector of orientation taken out by single landmark when the array was expanded. These data suggests that chicks, under certain circumstances, may be able to encode the geometrical properties of a landmark configuration.

# Abstract: ANTERIOR BUT NOT LATERAL THALAMIC NUCLEI LESIONS IMPAIR ALLOCENTRIC SPATIAL MEMORY

**Authors:** M. Wolff, S. J. Gibb, J. C. Dalrymple-Alford

**Affiliations:** ^1^Van der Veer Institute for Parkinson's and Brain Research, Christchurch, New Zealand; ^2^Department of Psychology, University of Canterbury, Christchurch, New Zealand

Medial thalamic damage is a common cause of severe memory disruption in humans. Both the anterior thalamic nuclei (AT) and the intralaminar thalamic nuclei (ILn) have been suggested as primary sites of diencephalic injury underlying learning and memory deficits, but their respective roles have yet to be resolved. The present study explicitly compared three memory tasks in rats with selective neurotoxic lesions either to the AT or the lateral medial thalamic (LT) nuclei; the LT lesions included the rostral ILn. Learning to escape from a Morris water maze using a visible platform that moved position across trials was rapidly acquired by both lesion groups. Consistent with previous studies, the AT group exhibited an impairment in the standard memory task, when the platform was now hidden in a fixed position beneath the surface of the water, but the LT group performed as well as Sham rats, thus providing a clear dissociation between the influence of AT and LT lesions in allocentric spatial reference memory. The final task used a radial-arm water maze, using any three arms on each trial, to test the acquisition of egocentric spatial reference memory for a left or right body turn; both lesion groups performed as well as the Sham group. The lack of deficits in LT rats on this last task contrasted with previous findings reporting a detrimental effect of LT lesions in a working memory egocentric task. This study highlights the relative importance of the AT in mediating allocentric spatial memory and provide an additional dissociation between AT and LT lesions.

# Abstract: EFFECTS OF SCOPOLAMINE IN THE RAT PRELIMBIC CORTEX IN CONSOLIDATION OF RELATIONAL AND IMPLICIT MEMORY TASKS

**Authors:** A. Carballo-Márquez, A. Vale-Martínez, G. Guillazo-Blanch, N. Boix-Trelis, M. Martí-Nicolovius

**Affiliations:** Institut de Neurociencies, Universitat Autonoma de Barcelona, Barcelona, Spain

Previous findings indicate that muscarinic receptor activation is involved in the consolidation of several tasks and that cholinergic input to the prefrontal cortex may modulate mnemonic processes. Evidence from our laboratory showed that pretraining muscarinic blockade with scopolamine in the prelimbic cortex disrupted learning of an odorodor associative memory task showing characteristics of relational memory, the social transmission of food preference. Nevertheless, the role of muscarinic receptors of particular prefrontal areas, such as the prelimbic cortex, in consolidation processes has not been completely specified. Experiment 1 determined whether the blockade of muscarinic cholinergic receptors in prelimbic cortex affects consolidation of the socially transmitted food preferences. Experiment 2 examined the effects of the same treatment on consolidation of a tone-shock associative implicit learning paradigm, the two-way active avoidance task. In both experiments, bilateral infusions of scopolamine hydrobromide (20&956 g/side) were administered immediately after training, and a reten tion test was carried out 24 hours later. The results showed that the blockade of muscarinic receptors significantly impaired retention of the relational memory task shown by the lower preference for an odor-cued food that had been learned 24 hours previously, but not the implicit task, where both groups show a similar number of correct avoidance responses. Such data suggest that muscarinic transmission in the prelimbic cortex is important for early memory formation of a relational socially-transmitted food preference task, but not of an implicit instrumental avoidance conditioning.

# Abstract: PARAFASCICULAR NUCLEUS INVOLVEMENT IN ACQUISITION, EXTINCTION AND REVERSAL LEARNING OF AN ODOR-REWARD ASSOCIATIVE TASK

**Authors:** I. Villarejo-Rodríguez, G. Guillazo-Blanch, A. Vale-Martínez, A. Carballo-Márquez, M. Quiroz-Padilla, M. Martí-Nicolovius

**Affiliations:** Institut de Neurociències, Universitat Autònoma de Barcelona, Barcelona, Spain

The parafascicular nucleus (PF) of the thalamus links brainstem arousal systems to cerebral cortical and basal ganglia networks involved in cognitive processes. Thus, the PF neurons seem to be strategically positioned to exert a powerful influence by supplying the optimal activation needed for selecting and/or encoding critical information. Previous studies carried out in our laboratory reported detrimental effects of pretraining bilateral PF lesions in retention and retrieval of an odor-reward associative task (ODT) after changing task contingencies, without affecting ODT acquisition. These PF lesion-induced deficits were attributed to the disruption of the appropriate prefrontal dynamics to show cognitive flexibility. The present study sought to further elucidate the specific cognitive functions related to PF nucleus. We evaluated the effects of PF lesions in the acquisition, extinction and the reversal learning of an implicit odor-reward association task (ODT). Pretraining bilateral N-methyl-D-aspartate (0.15 M) lesions were made one week before ODT acquisition (1 session, 5 trials). A single extinction session or a reversal session (5 trials) was conducted 24 hours after ODT acquisition. Latency for the correct responses and number of errors were recorded to obtain a performance score. Present data suggest a differential effect of PF lesions depending on the specific cognitive functions assessed. Considering anatomical and functional data, we suggest that the PF influence might be exerted by setting or maintaining the activation of prefrontal circuitry needed for displaying behavioral flexibility.

# Abstract: EFFECTS OF VOLUNTARY PHYSICAL EXERCISE ON ANXIETY, EXPLORATORY BEHAVIOR AND LEARNING AND MEMORY: DIFFERENTIAL EFFECTS DEPENDING ON EXERCISE INTENSITY

**Authors:** S. García-Capdevila, M. Torras-García, I. Portell-Cortés, M. Coll-Andreu, D. Costa-Miserachs

**Affiliations:** Department of Psicobiologia i Metodologia CC.SS., Instituto de Neurociències, University of Autònoma de Barcelona, Barcelona, Spain

Physical exercise can affect cognitive function as anxiety, learning and memory. Most studies show a decrease in anxiety related behaviors, but some find an increase. Regarding learning and memory, a facilitation of hippocampaldependent tasks is the most common result. This effect has been related with an increase on the expression of neurotrophic factors (BDNF) in hippocampus. A poorly understood aspect in these effects is the “dose”. It has been approached using treadmills at different speeds or durations. However, this forced exercise generally involves stressing procedures that can mask the effect of exercise. In the present experiment, we studied the effect of four weeks of voluntary exercise on a running wheel, upon behavior on an elevated plusmaze, an open field, and upon two different learning tasks: an object recognition task, that relies on perirhinal cortex and hippocampus, and a two-way active avoidance task, that relies mainly on the striatum where exercise do not increase BDNF expression. Subjects were young Wistar rats maintained by pairs in the homecage. Data were analyzed considering either the subjects as a whole or divided in three different groups: low runners (LR), high runners (HR) and very high runners (VR). We found that exercise increased most anxiety-like behaviors, especially in LR. Exploration behaviors were decreased by exercise, especially in VR. Regarding learning and memory, exercise modulated the object recognition task, but had no effect on the two-way active avoidance task.Unexpectedly, exercise modulated object recognition in a U-inverted manner, that is, LR were facilitated, whereas HR and VR were impaired. We conclude that exercise increase defensive behaviors, decrease the motivation to explore and modulate learning and memory depending on the neural circuit involved in the task. The intensity of exercise seems to be a crucial variable in these effects.

# Abstract: POST-TRAINING EPINEPHRINE IMPROVES MEMORY FOR OBJECT AND PLACE RECOGNITION, IN RATS

**Authors:** P. Jurado-Berbel, M. Torras-García, D. Costa-Miserachs, M. Coll-Andreu, I. Portell-Cortés

**Affiliations:** Department of Psicobiologia i Metodologia CC.SS., Institute Neurociències, University of Autònoma de Barcelona, Barcelona, Spain

Extensive evidence indicates that memory consolidation can be improved by posttraining injections of epinephrine. This effect has been observed mainly in tasks with emotionally arousing information, such as aversive and some appetitive tasks. The present experiment investigated whether posttraining epinephrine (0.01 mg/kg) can also improve memory consolidation for emotionally neutral tasks. Specifically, we studied the effect of post-training epinephrine (0.01 mg/kg) on memory 24 or 48 hr after the acquisition session of a standard object recognition task (Ennaceur and Delacour, 1988), a spontaneous task that does not require food or water deprivation, or any negative reinforcer. We also examined whether the same effect can be observed when dissecting two components of this task: the “what” component (24 h memory for the object itself) and the “where” component (24 h memory for the place where the object was located in the acquisition session). The results showed that all the groups recognized the object previously explored in the standard version of the task (both at 24 and at 48-hours retention), and they also recognized the familiar object and the location of it in the “what” and “where” component versions, respectively. This suggests that present task can be considered an implicit form of episodic memory. We also found that epinephrine enhanced standard object recognition memory either on the 24 and 48-hr retention tests. Moreover, epinephrine also improved memory for the “what” and “where” components of this episodic-like memory. Our results indicate that epinephrine can modulate memory for emotionally neutral tasks, as well as for emotionally arousing tasks.

# Abstract: INTRACRANIAL SELF-STIMULATION FACILITATES SPATIAL LEARNING AND MEMORY IN THE MORRIS WATER MAZE

**Authors:** J. Ruiz-Medina, I. Morgado-Bernal, D. Redolar-Ripoll, L. Aldavert-Vera, P. Segura-Torres

**Affiliations:** Unitat de Psicobiologia. Institut de Neurociències. Universitat Autònoma de Barcelona, Spain

Leaning and memory improvement by post-training intracranial self-stimulation has been observed mostly in implicit tasks, such as two-way active avoidance, in rats. Our laboratory has also shown that post-training self-stimulation can facilitate flexible expression of a learned response in a T-maze delayed alternation task. Here we wanted to know if posttraining intracranial self-stimulation is also able to facilitate a spatial learning and memory task in the Morris water maze. Four different experiments were run with Wistar rats. In each of them subjects were given at least 5 training sessions, one daily, consisting of two-minute duration trials. Starting from a variable position, rats had to swim in a 2 m-diameter pool until meeting a hide platform signalled by a cue located over the wall of its opposite site. Each daily session was followed by an immediate treatment of intracranial self-stimulation. Control subjects did not receive the self-stimulation treatment. In the four successive experiments rats were given eight, five, three and one trial per session, respectively. Temporal latencies and trajectories to meet the platform were measured for each subject. A strong and consistent facilitation of performance was observed from the second session onward when the rats were given only one daily trial. We did not observed differences between experimental and control groups in the other three experiments with larger amount of daily training. Besides confirming our previous suggestion that self-stimulation could facilitate explicit or relational memory, this finding also agree with our hypothesis stating that posttraining self-stimulation accelerates memory consolidation.

# Abstract: HIPPOCAMPAL GENE EXPRESSION IN INTRACRANIAL SELF-STIMULATION (ICSS) IN RATS

**Authors:** G. Huguet, L. Aldavert, E. Kádár, S. Pena, I. Morgado, P. Segura

**Affiliations:** ^1^Department of Biologia, Universitat de Girona, Girona, Spain; ^2^Department of Psicobiologia, Universitat Autònoma de Barcelona, Barcelona, Spain; ^3^Department of Biología, Universidad de Puerto Rico, San Juan, Puerto Rico

The purpose of our study is to contribute knowledge on the molecular mechanisms through which intracranial self stimulation (ICSS) on the medial forebrain bundle (MFB) is able to facilitate different kinds of learning tasks (classical conditioning, inhibitory and active avoidance, T-maze delayed alternation or Morris water maze). ICSS is a harmless and easily administrable technique that, besides being capable of facilitating learning and memory, could compensate some cognitive deficit caused by aging or by brain damage. Some of the facilitative ICSS effects have been attributed to structural changes (increase in dendritic intersection and length, spine density or in the number of excrescences) on brain areas related to learning and memory. Nevertheless, little is known about its molecular and functional mechanisms. In the ICSS procedure, the experimental subjects are taught to self-stimulate by pressing a lever with electrical current (at intensities ranging from 10 to 250 microA) by chronically implanted electrodes in MFB. By using high-density oligonucleotide arrays, we profiled gene expression in memory and learning-related brain regions of rats after ICSS treatment (2500 trains of ICSS at the optimal intensity of each rat) compared to control rats (with electrode implanted but without stimulation). Our analysis show more than 250 transcripts differentially expressed in the hippocampus. We want to report a small group of genes related to the increase of activity, ex. Kfl 15 a glucose transporter transcription factor, glycerol-3-phosphate 1, sarcomeric mitochondrial creatine kinase (Ckmt2) and Per 2. And we want to stand out an extended group of genes involved in signal transduction, most of them reported previously to be related with neural plasticity, LTP or learning; among the upregulated: c-Fos, PACAP, cart and Plcl1 and Pde1a in the downregulated ones. Our results provide a set of candidate genes to be related to the learning-enhancement properties of ICSS.

# Abstract: A BALANCED MEMORY NETWORK

**Authors:** Y. Roudi, L. E. Peter

**Affiliations:** Gatsby Computational Neuroscience Unit, UCL, UK

It is generally believed that persistent activity-the ability of a network to maintain its activity in the absence of inputunderlies working memory. Two salient experimental features of persistent activity are low firing rates and high variability. Existing models, however, do not elucidate how these are achieved. Here we propose a model that does. Based on this model, we find that only two condition are needed to ensure low, irregular firing: during learning, changes in the synaptic weights should be much smaller than the background weights, and the fraction of neurons selective for a memory should be above some threshold. When these are satisfied, parameters that support low, irregular firing can be found with very little fine tuning of network parameters. We also find that the number of memories that can be stored in such a network is proportional to the number of connections per neuron.

# Abstract: MALE AND FEMALE C-FOS EXPRESSION IN SPATIAL WORKING MEMORY

**Authors:** M. Méndez-López, M. Méndez, L. López, J. L. Arias

**Affiliations:** Laboratory of Psychobiology. School of Psychology. University of Oviedo, Spain

Several studies have shown sex differences in cognitive skills performance like spatial learning. These differences had been related to the gonadal steroids action on brain regions including components of Papezs circuit. According to this, neuronal expression of c-Fos is useful to investigate brain substrates related to spatial memory in males and females. The aim of this work was to assess the c- Fos immunoreactivity of anterior thalamic nuclei, dorsal hippocampus and medial and lateral mammillary bodies after training wistar rats (30 days) of both sexes in the Morris Water Maze. Two control groups of each sex were used to rule out the effect of swimming and handling on the c-Fos immunoreactivity measures. The training protocol consisted in a matching to sample procedure with one session per day of two equal trials, probe and retention, that changed throughout days. The behavioural results showed that both males and females were able to adquire the task with differences in speed adquisition. Males spent 4 sessions to reach the established criterion, forty session retention latency was significantly smaller than the probe (*P* < 0.033) and than first session retention (*P* = 0.005). However, females needed 8 sessions to reach significant reduction on retention latency related to probe trial (*P* < 0.001) and retention in session 1 (*P* = 0.002). Task related increase in c-Fos immunoreactivity was different between sexes in anterior thalamic nuclei and dorsal hippocampus. In males, significant increased was found in dorsal, ventral and medial anterior thalamic nuclei, but only anteroventral increase was described in females. Females hippocampal changes in c-Fos occurred in CA3 region and dentate gyrus but only CA3 was involved in males. Contrast to this, the medial and lateral mammillary bodies showed significant increase in both sexes. The results suggest that this spatial learning is differentially processed in male and female brain.

# Abstract: WHAT IS THE ROLE FOR THE PRELIMBIC-INFRALIMBIC AREA IN RULE SHIFTING: INVOLVEMENT IN UPDATING?

**Authors:** C. Oualian, P. Gisquet-Verrier

**Affiliations:** CNRS UMR 8620, Université Paris Sud, 91400 Orsay, France

A great deal of evidence indicates that the prelimbicinfralimbic (PL/IL) area of the rodent medial prefrontal cortex is implicated in behavioural flexibility, demonstrated by experiments investigating the effects of PL/IL dysfunction on rule shifting. However, PL/IL involvement largely depends on the type of rule shift, and the kind of PL/IL dysfunction. Here, the contribution of PL/IL was investigated following lesions and after specific injections of a dopamine D1 receptor antagonist, in a shift from a visual to a response strategy. Rats were trained first to enter the lighted arm of a Ymaze in order to get a food pellet and one day later to give always the same body turn, independently of the light location. In every case, rats were trained until a criterion of 10 consecutive correct responses. Ibotenic acid PL/IL lesions had no effect on performance or the ability to adapt to the new rule. These results contrast with those obtained when PL/IL dysfunction was induced by D1 antagonist. Rats with cannula in PL/IL were trained as in the previous experiment and received SCH 23390 PL/IL injections (1 *μ*L; 1 *μ*g) just before the rule shift. Unexpectedly, this treatment facilitated the shift from the visual to the response strategy. Analysis of the strategies used to solve the task revealed that this facilitative effect resulted from an inability for the subjects placed in the same environment to use previous information when required to learn a new rule. This result which suggests that PL/IL is involved in the capacity to update initially acquired memory, could also account for other performance disruption resulting from PL/IL dysfunction (extinction, drug reinstatement). Experiments accounting for the differential effects of the lesions versus the dopaminergic blockade are under investigation.

# Abstract: FUNCTIONAL MAPPING USING FOS IMMUNOHISTOCHEMISTRY OF THE NEONATAL RAT BRAIN DURING SPATIAL LEARNING UNDER FRUSTRATIVE NON-REWARD OR CONTINUOUS REINFORCEMENT

**Authors:** A. Diamantopoulou, A. Stamatakis, T. Panagiotaropoulos, E. Karantoni, F. Stylianopoulou

**Affiliations:** Lab of Biology-Biochemistry, University of Athens, Athens, Greece

Previous results from our laboratory using mother contact as a positive or negative reinforcer in a T-maze have shown that both frustrated (pups denied contact with the mother) and non-frustrated (rewarded by unrestricted contact) rats showed improved performance over training during postnatal days 10–13. The aim of the present study was to assess the brain areas involved in learning under frustrative conditions versus reward, using Fos immunoreactivity for functional mapping. Increased number of Fos immunopositive cells were found in every area studied for both frustrated and nonfrustrated rats compared to controls (naive animals). Frustration resulted in increased number of Fos immunopositive cells in the prefrontal cortex and the hippocampus, while in the striatum the number of Fos immunopositive cells was higher in the pups receiving continuous reward. These results indicate that non-frustrated rats receiving continuous reward develop a procedural-type learning process, as revealed by activation of the basal ganglia. On the other hand, frustrative non-reward leads to a behavioural state of increased vigilance and appraisal of environmental stimuli with concomitant activation of the prefrontal cortex and the hippocampus. It is known that the prefrontal cortex is responsible for decoding the reward (and punishment) value of primary reinforcers and for what is called stimulusreinforcement association learning; Moreover, it represents the components responsible for triggering the error-detection neurons that respond during frustrative non-reward. The hippocampus, which is also more activated in the frustrated than the nonfrustrated animals, plays a role in information processing decoding contextual changes and conflict between inherent drives and environmental stimuli.

# Abstract: DISSOCIATION OF ENTORHINAL AND PREFRONTAL CONTRIBUTIONS TO TRACE FEAR CONDITIONING

**Authors:** F. Esclassan, E. Coutureau, G. Di Scala, A. R. Marchand

**Affiliations:** ^1^Centre de Neurosciences Intégratives et Cognitives, CNRS UMR 5228, Univ. Bordeaux, 33405 Talence, France; ^2^Centre de Recherches sur la Cognition Animale, CNRS UMR 5169, Univ. Paul Sabatier, Toulouse, France

In trace conditioning, a temporal gap between the conditioned (CS) and the unconditioned (US) stimulus must be bridged to allow an association between these two stimuli. We previously showed that this process does not critically rely upon mediation by contextual stimuli, and proposed that some persistent representation of the CS must be maintained throughout the trace interval. Cortical structures such as the medial prefrontal cortex (mPFC) and entorhinal cortex (EC) are likely substrates for persistent representations. Naïve Long-Evans rats with excitotoxic lesions to either the mPFC (prelimbic and infralimbic cortices) or the EC were submitted to a trace (30 s) or zero-trace (0 s) conditioning protocol with five pairings of a tone CS (4000 Hz, 10 s, 70 dB) and footshock (0.4 mA, 1 s).The tone responsewasmeasured in a distinctive context 48 h after conditioning. Rats submitted to EC lesions showed a selective attenuation of freezing to a trace-conditioned tone, whereas rats with neurotoxic lesions of the mPFC showed deficits in zero-trace conditioning and a non significant reduction in trace-conditioned responses. This indicates that trace conditioning involves a specific contribution of the EC but not the mPFC. The critical role of the EC in trace conditioning could involve persistent activities requiring cholinergic activation. This hypothesis was tested through microinjections into the EC of cholinergic agents prior to conditioning. Microinjections of the M1 muscarinic antagonist pirenzepine strongly attenuated traceconditioned responses while leaving delay responses unaffected. We conclude that the mPFC is not specifically involved in trace conditioning whereas the EC plays a major role through cholinergic-dependent activities.

# Abstract: FLEXIBLE BEHAVIOUR IN A TASK OF EPISODIC-LIKE RECALL IN THE RAT

**Authors:** A. E. Fitchett, M. Jimenez, L. Webster, A. Easton, M. J. Eacott

**Affiliations:** Department Psychology, Durham University, Durham, UK

Recent work has demonstrated that rats can recall episodiclike memories within an E-shaped maze. Each day, in the Emaze, animals are presented with two novel objects to explore at different locations in different contexts. Rats are then removed from the E-maze and habituated to one of these objects in a separate environment. Finally rats are returned to the E-maze where their behaviour is measured as the object towards which they first turn. In experiments to date the question has been asked such that animals should turn towards the non-habituated object. Here we present evidence that manipulations to the task procedure can modify the animals behaviour in a predictable and flexible way. We discuss these findings in relation to whether the task can be made more salient and whether time cues can be used rather than contextual ones.

# Abstract: EARLY DETECTION OF SPATIAL MEMORY DEFICITS DURING AGING IN MICE

**Authors:** F. Céline, P. Géraldine, G. Emilie, M. Jean, R.-R. Laure

**Affiliations:** ^1^Laboratoire de Physiologie de la Perception et de l'Action, UMR CNRS 7152, Collège de France, Paris; ^2^Laboratoire Développement et Vieillissement du Système Nerveux, UMR NPA 7102, CNRS et Université Paris 6, Paris

Spatial memory is one of the first functions impaired during aging. Using the Morris water maze, most studies have shown a navigation deficit at the age of 25–27 months in rodents eventually revealing a subtle deficit around 15 months. Our aim was to detect much earlier spatial memory deficit and to characterize them. Navigation toward an invisible goal involves two complementary processes: the acquisition of a spatial knowledge and the use of this spatial knowledge in order to elaborate the most direct path toward the goal. We hypothesized that early detection might be possible studying these navigation processes specifically.

Therefore, we compared 3, 10, 17 and 20-month-old mice in an aquatic maze with alleys, developed in our team: the “starmaze”. This maze allows precise characterization of the different navigation processes mentioned above. The aim of the task is to reach a hidden platform located in a fixed alley. We characterized the mices ability to localize the platform and to develop a direct path toward the goal. We also checked anxiety, activity, motor and visual mices ability.

The results revealed that from the age of 10 months, two subgroups can be detected: one group presented abilities comparable to 3-month-old mice and the other falling outside the range of the youngs performance. 10-month-old bad learners were all impaired in the ability to elaborate a direct path and 67 percent of them were also impaired in their ability to localize the goal. The proportion of bad learners rose with mices age; we respectively observed 17, 47 and 55 percents of bad learners at the age of 10, 17 and 20 months. These deficits were not correlated with anxiety, poor motor ability, activity or visual impairment. In conclusion, studying navigation processes, we were able to detect spatial memory deficits from the age of 10 months which were characterized by a deficit to elaborate a direct path sometimes associated with a deficit lo localize the goal.

# Abstract: THE POTENTIALS OF CANTOR CODING IN HIPPOCAMPAL CA1 PYRAMIDAL NEURONS

**Authors:** Y. Fukushima, M. Tsukada, I. Tsuda, Y. Yamaguti, S. Kuroda

**Affiliations:** ^1^Brain Science Institute, Tamagwa University, Sapporo, Japan; ^2^Research Institute of Electronic Science, Department of Mathematics, Hokkaido University; ^3^Department of Mathematics, Hokkaido University, Sapporo, Japan

A mathematical model has been proposed that predicts Cantor coding in the hippocampal CA1 (Tsuda and Kuroda, 2001). This prediction includes attractor dynamics in the associative network, which is a feature that many authors have implemented since Marr's theory of simple memory in the hippocampus (Marr, 1971). Following up on Tsuda and Kurodas theoretical proposal, we have tried to obtain Cantorlike patterns of activity in experiments with CA1 pyramidal neurons. Temporally associated and non-associated electrical stimulation trains were delivered to Schaffer collaterals, and membrane potentials were recorded using the patch-clamp recording method. We found that at least two trains of advance electrical stimulation affect the ensuing neuronal responses. The time course of the response patterns depends on whether the sequential electrical stimulation is sufficient to induce action potentials. Our results suggest that the potentials of pyramidal neurons in hippocampal CA1 show Cantor-like coding of successive spatiotemporal information. This process appears to have at least two modes, depending on the membrane potential.

# Abstract: CANTOR CODING OF TEMPORAL SEQUENCES IN HIPPOCAMPAL CA1 MODEL NEURONS

**Authors:** Y. Yamaguti, S. Kuroda, I. Tsuda, Y. Fukushima, M. Tsukada

**Affiliations:** ^1^Department of Mathematics, School of Science, Hokkaido University, Sapporo, Japan; ^2^Research Institute for Electronic Science, Hokkaido University, Sapporo, Japan; ^3^Brain Science Institute, Tamagawa Univ., Machida, Japan

propose a mathematical model for the hippocampal CA1 in order to investigate how the CA1 network encodes the spatio-temporal input sequences from CA3. Since CA1 pyramidal cells have less recurrent connections compared with CA3 cells, it may be thought that CA1 has different functional roles from CA3. One hypothesis is that CA1 would be involved in the information processing of temporal input sequences. The hypothesis of “Cantor coding” in CA1 has been proposed by Tsuda and Kuroda (2001, 2003). Cantor coding provides the scheme for encoding temporal sequences of events. It forms a fractallike hierarchical structure in the state space of neural dynamics. Here, in order for investigating the Cantor coding hypothesis, we constructed a model for CA1 which consists of conductance-based model neurons. In the model, it was assumed that the CA3 outputs temporal sequences of spatial patterns, each of which represents a single episodic event. The pyramidal cells in CA1 are assumed to receive such activity. The response of the CA1 cells to each input forms a state vector, which is viewed as an information representation of CA1. It is revealed that the states of CA1 were hierarchically clustered in a self-similar manner according to the similarity of input sequences. Furthermore, linear discrimination analysis showed that distinct input sequences of up to about five length were well encoded in the CA1 state space without overlaps. These results suggest that fractal-like structure encoding time-series is formed in state space of neural dynamics of CA1.

# Abstract: TOLUENE DESTROY LEARNING PROCESSES IN YOUNG RATS

**Authors:** L. Gelazonia, L. Chilachava, N. Melia, N. Japaridze, Dashniani, M. Zhvania

**Affiliations:** ^1^Department of Neuroanatomy; ^2^Department of Behavior and Cognition Function, Institute of Physiology, Tbilisi, Georgia

The experiments were carried out in 28 rats of two age groups. Total of 14 experimental rats (7- one-month old and 7- two-month old) were subjected to toluene intoxication during 40 days. Two days later after cessation of toluene intoxication the ability of exploratory behavior and habituation to the environment of control and experimental rats of different age groups was estimated in the open field. Regularities of motor response learning were studied under conditions of Morris water tank seven days later after the cessation of toluene intoxication of control and experimental rats of different age groups. The experiments were carried out in the medium poor in cues. In the process of training, the location of platform to the start place did not change. A proper time (latent time) for searching of platform place and trajectory of swimming in each trial were registered. The criterion of learning appears to be a direct swimming from the start place to the platform in the consecutive four trials. Thus toluene intoxication of one-month old snuffer rats induced a deterioration of motor response learning. Experimental animals reach a criterion of learning task on the 7th day of training, while control group animals perform the task on the 3rd day. As compared to experimental animals, an average indices of latent time were reliably low (U criterion of Wilcoxon-Mann Witney *P* = 0.05). One of the reasons for the deterioration of learning processes may be considered a hyperactivity of experimental animals, clearly manifested during their testing in the open field. Regularities of motor response learning and results of testing in the open field of two-month old snuffer rats and control ones do not differ. The difference in the results obtained in two different age groups is due to a high sensitivity of one-month old rats to the action of toluene or to suppression of compensatory mechanisms of toluene action in two-month old rats.

# Abstract: EXCITOTOXIC LESIONS OF THE RAT MEDIAL PREFRONTAL CORTEX RESULT IN AN AUGMENTED LATENT INHIBITION EFFECT

**Authors:** D. N. George, A. Duffaud, S. Killcross

**Affiliations:** School of Psychology, Cardiff University, Wales, UK

Repeated non-reinforced presentation of a stimulus results in the retardation of subsequent conditioning to that stimulus relative to a non-preexposed (NPE) control stimulus. This effect is referred to as latent inhibition (LI). It has previously been reported that electrolytic lesions of the medial prefrontal cortex (mPFC) do not disrupt LI [1], and that excitotoxic mPFC lesions do not result in abnormally persistent LI [2]; in each case an off-baseline conditioned emotional response (CER) procedure was employed. [3] also reported no effect of excitotoxic mPFC lesions on LI; although they used an on baseline CER procedure, they did not assess suppression of the appetitive baseline during conditioning but only during a single subsequent test trial. In the experiment reported here, we examined the effect of mPFC lesions on LI using an on-baseline CER procedure similar to that reported by [4]. The foot-shock US was sufficiently weak (0.4 mA, 0.5 s) to allow us to examine the acquisition of suppression over several conditioning trials, and the preexposure regime was designed to yield little or no LI effect in sham operated control animals in order to maximise the opportunity to observe an augmented LI effect in lesioned animals. As expected, we observed no difference in the rate of acquisition of conditioned suppression to the preexposed (PE) and NPE stimuli in normal rats, but significantly slower acquisition to the PE stimulus than to the NPE stimulus for rats with mPFC lesions. Possible implications of these results for the switching model of LI [5] are discussed together with speculation about the roles of the prelimbic and infralimbic cortices in LI.

## References

[1] Joel D, Weiner I, Feldon J (1997). Electrolytic lesions of the medial prefrontal cortex in rats disrupt performance on an analog of
the Wisconsin card sorting test, but do not disrupt latent inhibition: implications for animal models of schizophrenia. *Behavioural Brain Research*.

[2] Schiller D, Weiner I (2004). Lesions to the basolateral amygdala and the orbitofrontal cortex but not to the medial prefrontal cortex produce an abnormally persistent latent inhibition in rats. *Neuroscience*.

[3] Lacroix L, Spinelli S, White W, Feldon J (2000). The effects of ibotenic acid lesions of the medial and lateral prefrontal cortex on latent inhibition,
prepulse inhibition and amphetamine-induced hyperlocomotion. *Neuroscience*.

[4] Killcross AS, Robbins TW (1993). Differential effects of intra-accumbens and systemic amphetamine on latent inhibition using an
on-baseline, within-subject conditioned suppression paradigm. *Psychopharmacology*.

[5] Weiner I, Feldon J (1997). The switching model of latent inhibition: an update of neural substrates. *Behavioural Brain Research*.

# Abstract: EXAMINING THE EXPLORATION/EXPLOITATION TRADE-OFF IN RATS: THE INFLUENCE OF ACTION TYPE, BEHAVIOURAL STATE AND PROXIMITY TO REWARD

**Authors:** J. E. Haddon, S. Killcross

**Affiliations:** School of Psychology, Cardiff University, Wales, UK

An important aspect of decision making is choosing when to continue to pursue a current goal and when to switch to pursuing other goals. Here, we present a novel procedure in rats, which may afford a more direct insight into this issue. This procedure involves the use of a testing chamber in which the four walls are LCD screens, the displays of which are computer controlled. As such the displays can be changed to provide information to the animal about its own position (or state) within a particular behavioural chain. We examined the influence of post-training reinforcer devaluation on lever press and magazine responding during the different stages of a simple levermagazine behavioural chain following under- and over-training. Rats were trained in a Distal state to press a lever to transition to the Proximal state in which a food pellet was delivered. Magazine entry to collect reward resulted in transition back to the Distal state. As each state (Lever press/Food delivery) was accompanied by changes in the environment (Distal and Proximal contextual cues) we were able to explicitly examine the selection of particular actions at particular states within the behavioural chain. Following completion of three sessions (Under-trained) or ten sessions (Over-training) of lever press training, animals received 5-minute extinction tests in the proximal and distal states. Prior to extinction testing, half the animals received pairings of the reinforcer with injections of lithium chloride (Devalued) and half the animals received injections of saline (Non-devalued). The results suggest that the proximity between an action and reward, and between a behavioural state and reward, both influence the decision to explore or exploit different elements of a behavioural sequence. Moreover, this trade-off between the influence of the behavioural state and action changes across training. This novel procedure provides a useful tool for further examining action selection in rats.

# Abstract: DISSECTING EXPLORATORY BEHAVIOUR: THE USE OF CUE-BASED SEARCHING STRATEGIES, VIEW-MATCHING AND EGOCENTRIC GUIDANCE DURING MORRIS WATER MAZE ACQUISITION

**Authors:** D. Harvey, S. Commins

**Affiliations:** Department of Psychology, National University of Ireland, Maynooth, Ireland

The Morris water maze (MWM), initially described over 25 years ago, was heralded as a simple and effective paradigm for examining spatial learning and memory. Since then, many elaborate studies, spanning a wide spectrum of areas within neuroscience, have employed this straightforward task, demonstrating its broad versatility and ease of adaptation to many protocols. However, despite innumerable findings since its introduction, the precise intricacies on how rodents acquire information regarding the hidden platforms location remain ambiguous. Often, overarching navigational strategies are reported when documenting rodent performance in the pool. These include those termed egocentric (defining the relation of an object, goal, or location relative to the subject) or allocentric (defining the relation of an object, goal, or location relative to another location, where this object is independent of the subject). Indeed previous research has strongly implicated the use of allocentric processes in solving the MWM; particularly through the use of visual distal cues in forming spatial relations with the platforms location. The current study demonstrates how animals interact with these external visual cues during acquisition of the task. It is shown that several identified swimming strategies, strongly associated with the cues, in combination with initial view-matching and egocentric guidance, ultimately allows animals infer the hidden platforms location (via allocentric extrapolation) with training. Furthermore, hippocampal BDNF expression is also shown to closely correlate with this acquisition, elucidating its role in learning.

# Abstract: OVERTRAINING IN RATS DOES NOT LEAD TO THE ACQUISITION OF A PROCEDURAL STRATEGY IN THE MORRIS WATER MAZE

**Authors:** K. John, D. Mairead, H. Deirdre, K. Elizabeth, M. Vanessa, C. Sean

**Affiliations:** Department of Psychology, NUI Maynooth, Maynooth, Co. Kildare, Ireland

Navigation of the Morris water maze (MWM) is achieved by using a combination of allocentric and egocentric strategies. Rats will use allocentric strategies over egocentric strategies in a hierarchical fashion. Two experiments were conducted to investigate whether rats could be trained to use a procedural (egocentric) strategy to solve the MWM task in preference to an allocentric one. In experiment 1, rats (*n* = 30) were trained in the light with full access to the distal cues to find the hidden platform (fixed in the NE quadrant) from a fixed starting position (NW quadrant) over 4 days (4 trials/ day). Seven days post-acquisition animals were divided into 4 groups, retention was then tested by allowing them to swim for 60 seconds in the platformless pool. Groups one and two were both re-tested in the light but group one (controllight) was placed into the pool from the trained starting position (i.e., NW) and group two started the probe trial from a position 180° away from this (start-rotated (SR)- light). Groups three and four also consisted of a control and SR group but both of these groups were retested in the dark. It was expected that both SR groups would search less in the NE quadrant than the non-rotated groups and that the SRdark group would rely on a procedural strategy and search in the opposite quadrant (SW). There was no evidence of the rats adopting this strategy. Animals in the SR-light condition searched in the NE quadrant similar to the control-light group, while both dark groups remained in their respective starting position or swam around the pool in a thigmotactic fashion. To examine this further, a second experiment was conducted with the same four conditions but this time animals (*n* = 30) were trained for 12 days. Results indicated that despite overtraining there was again no evidence that rats adopted a procedural strategy. These findings would suggest that rats do not employ a procedural strategy when solving the MWM task but rely heavily on distal cues to form spatial relations. In addition, the idea of a hierarchy of searching strategies at an animals disposal is also questioned. Funding was provided by the SFI and IRCSET.

# Abstract: IMPAIRMENT IN A CUED VERSION MAY BE COMPENSATED BY PRE-TRAINING IN A SPATIAL VERSION IN THE MORRIS WATER MAZE IN A RAT MODEL OF PARKINSON'S DISEASE

**Authors:** E. C. Wietzikoski, S. Wietzikoski, M. H. C. Silva, M. M. Ferro, J. C. Pedrozo Jr., N. S. Canteras, C. Da Cunha

**Affiliations:** ^1^Laboratório de Fisiologia e Farmacologia do Sistema Nervoso Central, Departamentos de Farmacologia e Biquímica, UFPR, Curitiba, Brazil; ^2^Departamento de Anatomia, Instituto de Ciências Biomédicas-3, USP, São Paulo, Brazil

The bilateral intranigral infusion of 1 *μ*mol 1-methyl-4-phenyl-1,2,3,6-tetrahydropyridine (MPTP) in adult male Wistar rats caused a specific and partial loss of substantia nigra pars compacta (SNpc) dopamine neurons, a partial depletion of striatal dopamine, and a deficit to learn the intra-maze cued version of the Morris water maze. Pretraining the SNpc rats in the spatial version of the water maze or simply maintaining the animals on the water maze platform reversed this deficit. This improvement was even observed when the order of the extramaze cues presented to the rats during pre-training of the spatial version was changed during training of the intra-maze cued version. However, this deficit was not reversed either by maintaining the animals on the platform if the spatial cues were surrounded and covered with a curtain or by swimming sessions in the maze without the escape platform and the curtain. These findings suggest that none of the following elements alone, learned during the spatial task pre-training, could help SNpc rats learn the intra-maze cued task: improvement of swimming skills or knowledge of the existence of the escape platform; distance between the platform and the border of the pool; location of a particular extra-maze cue; relations among extra-maze cues. However, the simultaneous presence of the escape platform and extra-maze cues (irrespective of their relational configuration) during the pretraining sessions proved to be necessary for this improving effect to occur.

# Abstract: SPATIAL RELATIONAL LEARNING PERSISTS FOLLOWING NEONATAL HIPPOCAMPAL LESIONS IN MACAQUE MONKEYS

**Authors:** P. Lavenex, P. Banta Lavenex, D. G. Amaral

**Affiliations:** ^1^Department of Medicine, Faculty of Science, University of Fribourg, UC Davis, Switzerland; ^2^Department of Psychiatry and Behavioral Sciences, University of Fribourg, UC Davis, Switzerland

The hippocampus plays a central role in the acquisition of spatial representations of the environment, and consequently in contextual memory. This suggests that the neural substrates underlying spatial cognition might be essential to remember specific life episodes. Indeed, hippocampal lesions prevent spatial relational learning in adult rodents and monkeys, and result in profound amnesia in adult humans. Here, in contrast, we show that monkeys with neonatal hippocampal lesions learned new spatial relational information. Our experiments suggest that early hippocampal damage leads to functional brain reorganization enabling the acquisition of spatial information using brain regions that normally do not subserve this function.

# Abstract: OPERANT LEARNING AS A TOOL FOR BEHAVIOURAL SCREENING AND QTL MAPPING OF RECOMBINANT INBRED MICE

**Authors:** H. A. Malkki, S. E. Schumann, R. N. Joosten, C. M. Pennartz

**Affiliations:** Center for Neuroscience, Swammerdam Institute for Life Sciences, Faculty of Science, Universiteit van Amsterdam, P.O. Box 94084, Kruislaan 320, 1090 GB Amsterdam, The Netherlands

Our knowledge of the genome of humans and other mammalian species has been rapidly increasing, but relatively little is known yet about the functions of these genes in cognitive processes, such as learning and memory. The aim of the project is to elucidate the relationships between chromosomal loci and heritable behavioral traits related to memory and learning processes by mapping quantitative trait loci (QTLs) that correlate with behavioral-cognitive abnormalities, leading to the identification of genes which play a role in memory and learning processes and corresponding cognitive disorders. In order to find QTLs, a large number of recombinant inbred (BxD) mouse lines are screened for behavioral cognitive abnormalities using appetitive conditioning paradigms. The first step of training is a classical conditioning task in which the mice have to learn to associate a stimulus light with a reinforcer. In the next stage, an operant conditioning task, the mice have to learn to press a lever in order to get a reinforcer. According to the performance of the mouse lines tested so far (14 BxD mouse lines and 6 common inbred mouse lines including the parental lines, *N* = 6–12 per line), as well as heritability estimates, the acquisition of operant behaviour has remarkable variation across the recombinant inbred mouse strains: The performance of the parental lines after five training sessions was 68.9 + 4.8% correct trials relative to the total number of trials (mean + SEM) for C57 and 67.2 + 4% for DBA mice. The performance of the BxD mouse lines varied from 1.4 + 0.2% to 85 + 4.5%. The heritability estimate [1] for the performance was 15.5%, suggesting that performance in this task has a heritable component.

## References

[1] Isles AR, Humby T, Walters E, Wilkinson LS (2004). Common genetic effects on variation in impulsivity and activity in mice. *Journal of Neuroscience*.

# Abstract: NORADRENALINE INDUCES LONG-TERM SYNAPTIC DEPRESSION IN RAT PREFRONTAL CORTEX: INVOLVEMENT OF ALPHA1- AND ALPHA2-ADRENORECEPTORS, NMDA-RECEPTORS, AND MAP KINASES

**Authors:** A. Marzo, P. Vanhoutte, J. Caboche, S. Otani

**Affiliations:** Department of Neurobiology, CNRS-UMR7102 Neurobiology of Adaptative Processes, Paris, France

Prefrontal cortex (PFC) is critical for higher cognitive functions. Noradrenaline (NA) acts in the PFC during attention as well as stressful situation and may modulate the higher cognitive functions. We tested the effect of NA on glutamatergic synaptic transmission in rat PFC using intracellular recording from layer V pyramidal neurons. Bath-application of NA (20 microM, 12.5 min) induced acute synaptic depression (−26.9 ± 5.6%) that was followed by long-term depression (LTD) (−26.3 ± 7.4% 40 min after NA washout, *n* = 8). We characterized cellular mechanisms underlying this NA-induced LTD. First, NA-induced LTD involves the activation of alpha1 and 2-adrenergic receptors, since prazosin (10 microM, alpha1-antagonist) or idazoxan (5 microM, alpha2-antagonist) blocked this LTD (11.9 ± 12.6% *n* = 8 and 17.6 ± 7.9% *n* = 7, respectively). Second, induction of this LTD requires synaptic activation of NMDA-R, since DL-AP5 (100 microM), applied during NA application, blocked LTD (6.5 ± 11.1%, *n* = 8). Requirement of concurrent synaptic stimuli for LTD induction was suggested also by omission of test synaptic input during NA application which blocked LTD (3.7 ± 12.6%, *n* = 6). Third, this LTD involves PKC and extracellular signal-regulated kinase 1/2 (ERK1/2) cascades: application of specific PKC inhibitor RO31-8220 (0.4 microM) or ERK1/2 inhibitor PD98059 (20 microM) both blocked LTD (3.3 ± 5.8%, *n* = 7 and 9.6 ± 7.3%, *n* = 7, respectively). Postsynaptic inhibition of ERK1/2 by synthetic peptide injection also blocked LTD, suggesting that critical activation of ERK1/2 occurs postsynaptically (6.4 ± 4.7%, *n* = 6). Finally, activation of ERK1/2 was quantified by western blot analysis. In conclusion, NA induces NMDA-Rdependent LTD in rat PFC that involves the activation of PKC and postsynaptic ERK1/2. Future in vivo research would reveal whether this NAinduced LTD represents a pathological state induced under stressful condition or it is part of physiological mnemonic processes.

# Abstract: INDUCIBLE INHIBITION OF CREB PHOSPHORYLATION IMPAIRS MEMORY AND BLOCKS IN VIVO LONG TERM POTENTIATION IN MCREB MICE

**Authors:** S. Middei, C. Pittenger, A. Reynolds, S. M. O'Mara

**Affiliations:** ^1^Trinity College Institute for Neuroscience, Trinity College, Dublin, Ireland; ^2^Clinical Neuroscience Research Unit, Connecticut Mental Health Center, New Haven; ^3^Institute of Genetics, Trinity College, Dublin, Ireland

CREB is implicated in learning and memory, likely thorugh its effect on the mechanisms of long term synaptic plasticity. To investigate this hypothesis, we measured both in vivo long term potentiation and memory performance in a Contextual Fear Conditioning (CFC) paradigm. The study was performed using transgenic mice in which an inducible mCREB mutation had been introduced. The mutation blocks CREB activation within the cell nucleus thus altering the CREB to function (Sakai et al., 2002). When compared to control mice, mCREB mutants show impaired behavioral performance in the CFC task. The induction of long-term potentiation in the dentate gyrus in vivo was also altered in mCREB mice. Together, the data suggest that impaired memory in mCREB mice could be dependent on altered synaptic transmission within the hippocampus.

# Abstract: PERFORMANCE IMPROVEMENT IS ASSOCIATED WITH DIFFERENTIAL HIPPOCAMPAL INVOLVEMENT AND NARROWED NEURAL PARTICIPATION: IMMEDIATE-EARLY GENE IMAGING AND DYNAMIC HIPPOCAMPAL NETWORK ACTIVITY IN RATS

**Authors:** G. Poirier, E. Amin, J. P. Aggleton

**Affiliations:** School of Psychology, Cardiff University, Cardiff, UK

Contrary to the long-held tri-synaptic loop view, hippocampal subfield function relies on parallel inputs that drive functionally different roles. In order to test the engagement of hippocampal subfields in an intact brain during the acquisition of a spatial task, we used immunohistochemistry to examine the induction of the immediate-early gene Zif268 in an extended hippocampal system in the brain of rats (dentate gyrus, CA1, and entorhinal, postrhinal and retrosplenial cortices). Two groups of rats were tested in a hippocampal-dependent task in a radial-arm maze, each trained to a different extent, for either two or five sessions (Early or Late). Control groups were used, where each rat was yoked to an experimental rat, and obtained the same number of rewards, visited the same number of maze arms and spent a similar amount of time in the maze. Structural equation modeling analyses indicated dynamic network effective connectivity, which was masked by the lack of Zif268-positive cell count differences between the conditions. The engagement of hippocampal subfields in network activity displayed by the two trained groups was unequal. The tri-synaptic loop was bypassed with training, as the dentate gyrus specifically became disengaged and reduced its neural influence. The differential neural engagement of hippocampal subfields was supported by the finding of strong associations of performance measures with dentate gyrus Zif268 expression in the Early but not the Late training group, and conversely with CA1 Zif268 expression in Late but not the Early training group. The findings of the current experiment suggest both a dynamic role of hippocampal subfields in spatial memory function and a dynamic pattern of network activity, both evolving in parallel with task mastery.

# Abstract: IMMEDIATE EARLY GENE EXPRESSION IN SUBREGIONS OF THE RETROSPLENIAL CORTEX IN RESPONSE TO A SPATIAL WORKING MEMORY TASK

**Authors:** H. J. Pothuizen Helen, Davies Moira, John P. Aggleton, D. Vann Seralynne

**Affiliations:** School of Psychology, Cardiff University, Cardiff, UK

It is now recognized that the retrosplenial cortex is important for spatial learning and memory in rodents, consistent with the dense anatomically connections with other structures known to support this class of memory, that is, the hippocampal formation and the anterior thalamic nuclei. To date, lesion studies have been unable to define the specific role of the retrosplenial cortex in spatial memory which might be, in part, due to the majority of lesion studies treating the retrosplenial cortex as a unitary structure. The retrosplenial cortex can, however, be separated into two main subregions: “granular” and “dysgranular”. These subregions differ both in terms of their morphology and anatomically connectivity and it is, therefore, possible that they may also contribute differentially to spatial learning and memory. The present study used immediate-early gene (IEG) expression to ascertain the specific involvement of these separate subregions in spatial memory. Using IEGs as a marker of neuronal activity enabled us to examine all of the retrosplenial subregions within the same animals, as well as allowing further differentiation within these subregions. Male, Dark Agouti rats were either trained on a working memory task in an 8-arm radial maze or were “yoked” controls, matched for sensorimotor stimulation in the radial-maze but without the mnemonic component. c-fos and zif268 expression was quantified at different levels along the rostro-caudal axis of the granular a (Rga), granular b (Rgb) and dysgranular (Rdg) cortices. Although there was increased IEG expression in all retrosplenial subregions (regardless of rostro-caudal level) in the experimental compared to the control rats, the most pronounced difference was found in Rga. The present results implicate all subregions of the retrosplenial cortex in spatial working memory in normal rats, but also highlight a more specific contribution from retrosplenial granular a.

# Abstract: A CONSISTENT LEFT WARD BIAS IN SPATIAL TASKS IN AN ANIMAL MODEL

**Authors:** L. Regolin, R. Rugani, G. Vallortigara

**Affiliations:** Department of General Psychology, University of Padova, Italy; Department of Psychology and BRAIN Centre for Neuroscience University of Trieste, Italy

A leftward bias has been reported for adapted versions of the cancellation task in two species of birds (i.e., the pigeon and the domestic chicken, Diekamp et al., Current Biology, 2005), and for an adapted version of the line bisection task in domestic chicks (Regolin, Cortex, 2006). Left-right asymmetries were here investigated by training (for foodreinforcement) young domestic chicks (*N* = 21) to locate a certain element within a series of identical and aligned elements on the exclusive basis of its positional serial order. Birds were trained to locate the fourth in a series of ten elements positioned right in front of each chick (i.e., sagittally, so that the first element was the closest to the subject and the final element was the farthest) and then tested after the same array had been rotated by 90° (i.e., so that all elements were virtually equidistant from the subject). The test consisted in twenty consecutive trials. At test, both the fourth item from the left end and the fourth from the right end constituted correct choices, nevertheless 16 chicks significantly chose the fourth position from the left, while only 4 chicks responded preferentially on the fourth position from the right (and one chick selected both positions equally). It is plausible that a bias for preferentially attend to objects located in the left hemispace is at the base of such consistent series of observations; such bias could possibly relate to a right hemispheric dominance for spatial tasks in avian species.

# Abstract: HIPPOCAMPAL PLACE CELLS FIRING IN ZIF268 KO MICE

**Authors:** S. Renaudineau, B. Poucet, E. Save

**Affiliations:** Laboratory of Neurobiology and Cognition Marseille, France

Location-specific activity of place cells and long term potentiation (LTP), two mechanisms crucial for memory, were discovered in the hippocampus more than 25 years ago. How these two phenomena are related is still unclear. Recent studies have revealed the importance of the immediate early gene zif268 in both LTP and spatial memory. First, deletion of Zif268 in mice does not affect LTP induction and short term maintenance but impairs its long term maintenance. Second, Zif268 mutant mice have a long term, but not a short term, memory deficit in spatial memory tasks. Our purpose was to investigate place cell firing in Zif268 KO mice and examine its short and long term stability. Place cells were recorded as mice explored a highly familiar environment (square) and were exposed to a new environment (circle), a situation that usually induces formation of a new representation (remapping). Successive sessions were conducted to examine whether place cells in Zif268 1) Maintained stable firing field in the square (S1, S2, S4), 2) Remapped in the circle (S3), 3) Re-activated the circle representation after a 1H delay (short term memory), and 4) Re-activated the circle representation after a 24H delay (long term memory). We found that place cells in Zif268 maintained stable firing fields in the square (S1, S2) and remapped in the circle (S3). Surprisingly, a proportion of cells was unable to re-establish the familiar representation in the square afterwards (S4). Lastly, both short term and long term stability of firing fields were affected in Zif268. The results suggest that the Zif268 gene is involved in the formation and maintenance of place celldependent spatial memory.

# Abstract: INTERACTION OF ARENA SHAPE WITH OTHER CUES IN THE CONTROL OF PLACE CELL FIRING LOCATION IN A ROTATING RECTANGULAR ARENA

**Authors:** S. E. Rhodes, M. Good, R. C. Honey, S. Killcross

**Affiliations:** School of Psychology, Cardiff University, Cardiff, UK

Although place cell firing is governed by a variety of sensory cues (O'Keefe and Speakman 1987; Shapiro, Tanila et al. 1997), despite considerable research it is far from clear exactly how this is done. As a contextual cue, the shape of the environment can influence whether a place cell fires or not, with remapping seen between two differently shaped arenas in the same spatial location (Lever, Wills et al. 2002; Paz-Villagran, Save et al. 2004). However as a geometric cue, the positional control of place fields by arena shape is less clearly defined. Following training in an environment where the distal cues are stable relative to the orientation of the arena, there is evidence that distal cues dominate over arena shape in the control of place field location when the two are subsequently rotated relative to each other (Cressant, Muller et al. 2002). However there is evidence that in situations whereby distal cues are perceived as unstable, they gain less control over place field location relative to other cues (Knierim, Kudrimoti et al. 1995). In the current study, place cells were recorded in a rectangular arena that was rotated from trial to trial. The findings indicated that arena shape can control the position of place fields in a situation whereby distal cues and arena entry point were unstable across trials. Furthermore there is also evidence for interaction between arena geometry and other cues in the orientation of place fields in this rotationally symmetrical environment.

# Abstract: PREFERENCE FOR FACE-LIKE STIMULI IN AN ANIMAL MODEL

**Authors:** O. Rosa Salva, C. Romor, L. Regolin

**Affiliations:** Department of General Psychology, University of Padova, Padova, Italy

There has long been a debate as to whether infants preference for looking at face-like stimuli with respect to non facelike ones (Goren, Sarty and Wu, Pediatrics, 1975) is determined by the presence of an innate schematic representation of conspecific faces' appearance (CONSPEC, Morton and Jonhnson, Psychol review, 1991), or by the so called “up-down bias” (a bias that favours stimuli presenting more high contrast elements in their upper part with respect to the lower part, Simion et al., Developmental Sci, 2002). The present work investigated the presence of an innate preference for face-like schematic stimuli in two day-old domestic chicks, controlling for the role of the up-down bias. Subjects (*N* = 34) were completely naïve with respect to the arrangement of the internal faces features: they had no visual experience of any face prior to the experiment. During the test chicks were presented with two stimuli (a face-like stimulus and a non face-like one), both having more elements in the upper part of the figure (i.e., balanced with respect to the up-down bias) and the same symmetrical/asymmetrical pattern with respect to the vertical and horizontal axes. Chicks preferred to approach (X2 = 5.765; *p* = 0.016) and spent more time near the face-like stimulus (*t* = 3.525; *p* = 0.001), showing an innate preference for a schematic face-like configuration. This is consistent with what has previously been shown in studies on infants face perception. Support from: PRIN 2005 - PROF. MARIO ZANFORLIN.

# Abstract: INTERFERENCE OF SPATIAL TASKS ON EMOTIONALLY AROUSING INFORMATION IN RODENTS AND HUMAN

**Authors:** D. Saraulli, M. Aceti, C. Rossi-Arnaud, M. Costanzi, V. Cestari

**Affiliations:** ^1^Istituto di Neuroscienze del CNR – Sezione di Roma, via del Fosso di Fiorano, 64 – 00143 Roma, Italy; ^2^Facoltà di Scienze della Formazione, Università LUMSA, piazza delle Vaschette, 101 – 00193 Roma, Italy; ^3^“La Sapienza”, Facoltà di Psicologia, Università di Roma, via dei Marsi, 78 – 00185 Roma, Italy

In the present study three experiments investigated the effects of spatial interference on memory retention for information associated with emotional stimuli. In the first experiment CD1 mice were submitted to an inhibitory avoidance task (IA) with a pre-training interfering task (place object recognition task; PORT) administration. Tests were carried out 3 sec (working memory, WM), 30 min (short-term memory, STM), and 24h (long-term memory, LTM) after training. Results showed that PORT administration significantly impaired WM and STM for IA. Interestingly, LTM was not affected by the interfering task. In the second experiment CD1 mice with bilateral lesions of dorsal hippocampus (hippo) or medial prefrontal cortex (mPFC) were submitted to the same interfering procedure. Results showed that mPFC lesions rescued the impairing effects exerted by PORT administration on WM and STM for IA, while the hippocampal lesions impair both STM and LTM, suggesting a complex relationship between hippocampus and mPFC in mediating the effects exerted by spatial interference on memory. In the third experiment in which objects were associated to neutral and emotional pictures. Tapping was used as a spatial interfering task during stimuli presentation. Tests were carried out both immediately and 24h after training. Results showed that tapping impaired performance both immediately and 24h after learning, although an improving effect of emotional pictures on object relocation was observed in the test carried out 24h after training. Taken together, our results suggest that both in humans and mice (1) spatial interfering task administration affect spatial working memory; (2) the association between emotional stimuli with spatial information decreases the effects exerted by the spatial interference on long-term memory.

# Abstract: MODULATION OF SPATIAL AND STIMULUS-RESPONSE LEARNING STRATEGIES BY STRESS AND CORTICOSTEROIDS IN MICE

**Authors:** L. Schwabe, H. Schächinger, M. S. Oitzl

**Affiliations:** ^1^Department of Clinical Physiology, Institute of Psychobiology, University of Trier, Trier, Germany; ^2^Division of Medical Pharmacology, LACDR/LUMC, University of Leiden, Leiden, The Netherlands

Previous findings indicated that psychosocial stress administered prior to training in a spatial task that could be acquired by spatial and stimulus-response strategies modulates the employed learning strategy of humans in a manner that favours nucleus caudate-based stimulus-response learning over hippocampus-based spatial learning (Schwabe et al., 2007). We hypothesized that the glucocorticoid stress hormones are the modulatory mechanism. Male C57BL/6j mice (*n* = 12/group) were either left untreated, received corticosterone (250 *μ*g/kg s.c.) or restraint stress (10 minutes) 30 minutes before performing a circular hole board task. Mice had to find one exit hole which could be located by both, a spatial and a stimulusresponse strategy (6 trials, ITI 15 min). Trial 7 revealed the used learning strategy. Twenty-four hours later another three trials were given. On day 1, all untreated mice employed a spatial strategy, while 25 percent of the corticosterone and restraint group used a stimulusresponse strategy (*p* < 0.05). Furthermore, untreated mice had significantly shorter latencies to the exit hole than corticosterone-injected and restrained mice (*p* < 0.01). On day 2, strategy and latency to the exit were comparable, indicating good memory performance. We conclude that (a) the stress hormone corticosterone modulates the use of spatial and stimulus-response learning strategies, (b) stress and stress hormones affect both the used strategy and the latencies to the exit hole, as spatial learners of the corticosterone and restraint group had longer latencies than those of the untreated group, and (c) acute stress and corticosterone affect the learning strategy and latency during acquisition without changing memory consolidation and retrieval.

# Abstract: BEHAVING IN GEOMETRIC MAZES WITHOUT A GEOMETRIC MODULE: A MODEL OF INTERACTION BETWEEN VISION, PATH INTEGRATION AND NAVIGATION STRATEGIES IN RODENTS

**Authors:** D. Sheynikhovich, W. Gerstner

**Affiliations:** Laboratory of Computational Neuroscience, Federal Institute of technology (EPFL), Lausanne, Switzerland

Behavioral and neurophysiological experiments suggest that the geometric arrangement of walls in the environment plays an important role in rodent navigation. Although nongeometric information can be used to identify a goal location, disoriented animals seem to relymostly on the environmental shape when relocating previously encountered food. Using a computational model of rat navigation, we propose and test a hypothesis that the influence of environment geometry can be explained by the use of a hippocampus-dependent ‘locale’ navigation strategy in combination with a view-based reorientation mechanism. The model can also use a stimulus-response ‘taxon’ strategy in the same environment, in which case the influence of geometry is decreased. Based on these results we argue that behavioral decisions previously attributed to a ‘geometric module’ can be explained by the interaction between the locale and taxon strategies, both based upon snapshots of the environment, that is without explicit representation of geometric cues.

# Abstract: BOMBINAS (BOMBINA ORIENTALIS) GO FOR MORE

**Authors:** G. Stancher, G. Vallortigara, L. Regolin

**Affiliations:** ^1^Department of Psychology and BRAIN Centre for Neuroscience, University of Triest, Italy; ^2^Department of General Psychology, University of Padua, Italy

When placed in front of two different sets of edible items, animals spontaneously (i.e., without the need of a specific training) tend to chose the larger quantity, performing an adaptive choice consistent with the predictions of the “optimal foraging strategy” theory. The ability to ‘go for more’ has been recognized in evolutionarily remote species, such as fish and salamanders as well as in human infants and in non-human primates. Choice of the larger amount can be considered as a rudimentary mathematical ability, although it is still unclear its relation to the systems of categorization of experiences from which, in humans, the concept of number arises. We investigated, in seven adult subjects of Bombina orientalis, spontaneous choices for different numerosities of Tenebrio molitor larvae (1 vs 2; 2 vs 3; 3 vs 4; 4 vs 8) as a first evidence of numerical discrimination ability within this species. Subjects could freely approach any of the two groups of larvae that were placed in front of them. The data showed that bombinas discriminated the two quantities and chose the larger amount when faced with sets of 1 vs 2 (*p* = 0.0009) and 2 vs 3 (*p* = 0.0489), but not when the paired numbers were 3 vs 4 (*p* = 0.4816). Though, four items was not the absolute limit to discrimination as, in the case of 4 vs 8 mealworms, when the ratio between the two numerosities was large enough, bombinas were capable of significantly choosing the larger set (*p* = 0.0059). These results are consistent with those obtained in salamanders and also show striking similarities with the performance of monkeys and human infants. Overall, in a comparative perspective, the existence of an archaic non-linguistic systems for numerical discrimination seems plausible. Whether the cognitive mechanisms at the basis of the observed abilities are the same in all the species remains at present an open issue.

# Abstract: DUAL EFFECTS OF TAURINE SUPPLEMENTATION ON VISUAL DISCRIMINATION TASK IN MICE

**Authors:** R. Suge, N. Hosoe, M. Furube, T. Yamamoto, A. Hirayama, S. Hirano, M. Nomura

**Affiliations:** ^1^Department of Physiology, Saitama Medical University, Saitama, Japan; ^2^Department of Internal, Toho University Sakura Medical Center, Chiba, Japan; ^3^Department of Physiology, Tokyo Dental College, Chiba, Japan; ^4^Laboratory of Radioisotopes, Tokyo Dental College, Chiba, Japan; ^5^The Nukada Institute for Medical and Biological Research, Chiba, Japan

Taurine, 2-aminoethane-sulphonic acid, found in high concentrations in the central nervous system of mammals, has been shown to be essential for the development. There were several reports that taurine supplementation in early life induced neuronal and behavioural changes. In this study, the effects of taurine supplementation at different times on visual discrimination task in mice were examined. Male mice were assigned into four groups by the supplementation periods. (i) Lifelong: Taurine dissolved in distilled water (0.12%) was provided as drinking water. In the perinatal to early post-natal period, the taurine was given to through subjects' mothers. After the weaning subject uptake the taurine by itself as the drinking water by the end of the experiment. (ii) Pre-weaning: Subjects were exposed to the taurine by the weaning, (iii) Post-weaning: from after the weaning. (iv) Control: Subjects were drinking distilled water throughout the experiment. For acquisition of the task, the Lifelong group needed longer period than the Control group. In the Post-weaning group, conversely, mice learned the task quicker than the control. The Pre-weaning group showed no difference compared with the control. Thus, the retardation of the task acquisition occurred with the supplementation in early and adult life. At the same time, the taurine supplementation after weaning improved the visual discrimination learning. We suggest that timing of the supplementation affected the effect of the taurine supplementation in adult on the learning and the perinatal-early postnatal period might be “sensitive period” for the retardation of learning.

# Abstract: ROLE OF PARIETAL CORTEX OF THE RAT IN TWO TASKS INVOLVING DYNAMIC ENVIRONMENT

**Authors:** J. Svoboda, P. Telensky, J. Bures

**Affiliations:** ^1^Institute of Physiology of Czech Academy of Sciences, Prague, Czech Republic; ^2^Psychiatric Centre Prague, Prague, Czech Republic; ^3^Department of Zoology, Faculty of Science, Charles University, Prague, Czech Republic

Most spatial tasks require animals to organize their behavior in relation to stable environmental cues. However, cue manipulations such as rotation or transition allow to assess their control exerted over behavioral processes and to examine underlying brain mechanisms. We used Long-Evans rats with lesion of parietal cortex in two behavioral tasks taking place in dynamic environment. Both of them were conducted on elevated circular arena (*d* = 80 cm), in 20 min daily sessions. At first, the rats were trained 6 days in Robot Avoidance Task (RAT), in which the rat must keep safe distance at least 25 cm from a programmable robot to avoid a mild footshock. The robot was programmed to move straigth forward (15 cm/s) until it hit the wall, then it waited for 15 s, turned 1800 to 90 degrees, and ran again. Two weeks after the first experiment, food-deprived rats were trained to asymptotic performance in a modified version of Place Avoidance (PA) task to search for randomly dispersed pellets while avoiding a small sector on the arena. Since the sector is not directly perceivable rats can remember its position either in coordinate frame of the arena, or in frame anchored to cues outside the arena. By slow arena rotation under extinction condition, we may examine which of these two frames well-learned rats prefer. Results showed that parietal rats (*n* = 8) were slightly but not significantly facilitated (*p* = 0.10) in learning RAT when compared to controls (*n* = 8). In addition, they did not differ in locomotion nor in thigmotaxis. The PA task revealed that parietal rats chose arena frame more frequently than controls, though both groups learned the task at the same rate. Our results do not support data of other authors obtained in static environment tasks that suggested parietal cortex would play a role in processing proximal spatial cues.

# Abstract: ENTORHINAL CORTEX LESIONS PRODUCE DELAY-DEPENDENT DEFICITS IN HABITUATION BUT NOT IN REACTION TO SPATIAL CHANGE IN AN OBJECT EXPLORATION TASK

**Authors:** T. Van Cauter, B. Poucet, E. Save

**Affiliations:** Laboratory of Neurobiology and Cognition UMR 6155, CNRS-Université de Provence, Marseille, France

Formation, consolidation and retention of episodic memories require an interaction between the hippocampus and the neocortex. The entorhinal cortex (EC) is at the interface of the hippocampus and neocortex and therefore may play an important role in memory processes. Supporting this hypothesis, previous data have shown that rats with EC lesions display a delay-dependent retention deficit in spatial and non spatial learning tasks. In this study, we tested the effects of EC lesions in an object exploration task involving habituation, spatial and non spatial memory. Habituation to a configuration of 4 objects was examined during 6 successive sessions separated by a 4 min or a 10 min inter session interval. Following habituation, a spatial change was made by displacing one object and the reaction to change was measured after a 30 sec, 4 min or 10 min interval. Lastly, a non-spatial change was made by substituting a familiar object by a novel object and the reaction to change was measured after a 4 min or a 10 min interval. The results showed that EC-lesioned rats exhibited a deficit of habituation at the 10 min but not the 4- min interval. They exhibited a deficit in the reaction to spatial change at all intervals and were not impaired in the reaction to non spatial change. The results suggest that the EC 1) plays a role in non associative memory processes, 2) is differentially involved in habituation and spatial memory processes, 3) is not involved in non spatial memory processes.

# Abstract: STRUCTURAL REARRANGEMENTS WITHIN HIPPOCAMPAL-CORTICAL NETWORKS MEDIATE REMOTE MEMORY FORMATION

**Authors:** G. Vetere, L. Restivo, B. Bontempi, M. Ammassari-Teule

**Affiliations:** ^1^IRCCS, S. Lucia Fondation, Italy; ^2^CNR, Istitute for Neuroscience, Rome, Italy; ^3^Laboratoire de Neurosciences Cognitives, CNRS UMR 5807, Universite Bordeaux I, Talence, France

Although hippocampal-cortical interactions are crucial for the formation of enduring memories, the temporal dynamics of plasticity mechanisms that govern long-term memory storage remains unclear. We present evidence that the formation of remote, but not recent, fear memory requires structural remodelling of synapses on anterior cingulate cortex neurons while a time-inverted pattern of structural changes involving spine formation/elimination occurs on hippocampal neurons. Cortical rewiring is achieved before retrieval of remote memory. Post-training hippocampal lesions disrupt retrieval and abolish structural rearrangements in cortical networks. These findings suggest that progressive changes in the wiring diagram of cortical networks are involved in the formation and expression of remote memory, and that the hippocampus plays only a time-limited role in driving wiring plasticity in the cortex.

# Abstract: BEHAVIOUR-RELATED CHANGES IN EXTRACELLULAR AMINO-ACID LEVELS IN THE MEDIAL STRIATUM OF THE DOMESTIC CHICK: AN IN VIVO MICRODIALYSIS STUDY

**Authors:** G. Zachar, A. K. Kékesi, G. Juhász, P. Kabai, A. Csillag

**Affiliations:** ^1^Department of Anatomy, Semmelweis University, Budapest, Hungary; ^2^Department of Physiology and Neurobiology, ELTE, Budapest, Hungary; ^3^Szent Istvn University, Faculty of Veterinary Sciences, Budapest, Hungary

The medial striatum (MSt) plays a role in the passive avoidance learning as well as in reinforcement learning of the domestic chick. MSt receives dopaminergic and glutamatergic afferents, and a coincidence between excitatory amino acid related and dopaminergic inputs are necessary for memory consolidation. Daisley et al. (1998) showed that the extracellular glutamate level in the MSt is sensitive to stress. In the present study we used both one trial aversive and serial reinforcement conditioning combined with in vivo microdialysis to monitor the changes of the extracellular levels of different amino acids. Microdialysis probes were stereotaxically implanted into the medial striatum of one-day-old chicks. The birds were then trained either by the one-trial passive avoidance paradigm or by a combination of operant conditioning using water reinforcement, habituation learning and taste aversion learning. As conditional stimuli, coloured beads were presented to the individuals, and the number of pecks was recorded during the experiments. The occasional handling of chicks was also recorded as a possible stress source. Microdialysis samples were collected during the experiment at 15 minute intervals, and analysed later by HPLC. Our results showed that stress elicited a marked elevation of the level of aspartate and glutamate but not those of other amino acids. Such changes were not observed in anesthetised birds. This effect increased the variance of baseline values and masked most of the behaviour related responses. However, a slight but significant decrease of aspartate and glutamate occurred after the presentation of the aversive stimulus. We find it potentially meaningful to detect neurochemical correlates of behaviour by in vivo microdialysis in the MSt of freely moving chicks. Stress could be a potent factor affecting the levels of excitatory amino acids and thereby the NMDA and non-NMDA receptor mediated consolidation of memory.

# Abstract: LEARNING-INDUCED POST-SYNAPTIC ENHANCEMENT OF EXCITATORY AND INHIBITORY SYNAPTIC TRANSMISSION

**Authors:** S. Drorit, B. E. B. Inbar

**Affiliations:** Departments of Biology and Neurobiology, University of Haifa, Haifa, Israel

The brain, much as other biological complex systems, maintains homeostasis for stability and survival. Thus, homeostatic plasticity must occur in neurons and neuronal networks under various physiological processes, such as learning and memory. To fully understand how large neuronal ensembles undergo permanent changes while maintaining their functionality, three components of the total neuronal excitability and the manner by which their action is combined must be fully described: the excitatory synaptic drive mediated mainly by glutamate receptors, the intrinsic neuronal excitability, and synaptic inhibition mediated by GABA receptors. The purpose of the present study was to explore if and how excitatory and inhibitory synaptic inputs onto pyramidal neurons are co-modified after olfactory learning to balance each other's effects.

Rats were trained in four-arm maze to discriminate between odors in pairs. As previously shown, rats require 6–8 consecutive training days to learn to distinguish between a pair of odors, but to learn a second pair of odors only requires 1–2 training days (rule learning). Piriform cortex brain slices were prepared 4–5 days after rulelearning. Whole-cell voltage-clamp recordings were obtained from layer II pyramidal neurons at 300 °C, with Vm held at −80 mV. Intracellular recordings were performed with sharp electrodes at 300 °C.

The averaged amplitude of the minimal (quantal) spontaneous AMPAreceptors mediated events was significantly larger (*P* < 0.01) in neurons from trained rats (8.2 ± 2.9 pA, *n* = 18) compared to neurons frompseudo-trained (6.1 ± 1.0 pA, *n* = 14,) and naïve rats (5.7 ± 1.4, *n* = 15). Thus, the single quantum increases after rulelearning. Accordingly, the averaged amplitude of the spontaneous events was significantly larger in neurons form trained rats. In contrast, the frequency of spontaneous events was similar in both groups, indicating that the probability of release is not modified after learning. To evaluate changes in inhibitory synaptic transmission, intracellular recordings with sharp electrode were made from layer II pyramidal neurons. IPSPs were evoked by electrical stimulations applied in layer III, in the presence of APV and DNQX, to block glutamatergic synaptic transmission. The averaged fast IPSP's reversal potential was significantly lower in neurons from trained rats (−77.1 mV ± 5.3, *n* = 16 for trained vs −70.4 ± 7.2, *n* = 17 for naive and −69.6 ± 5.2, *n* = 16 for pseudo trained, *P* < 0.01), indicating enhancement of inhibitory synaptic efficacy.

Our data support the notion that olfactory learning is accompanied by longlasting post-synaptic modifications of excitatory and inhibitory synaptic transmission onto piriform cortex pyramidal neurons. These co-modifications allow enhancement of excitatory synaptic transmission between pyramidal neurons, while preventing the cortical network from entering into a hyper-excitable state, where in which strengthening of undesired synaptic connections may occur.

# Abstract: Theme C: Cognition (human)  HIGH-LEVEL AFTEREFFECTS IN VISUAL PROCESSING OF COMPLEX IMAGES

**Authors:** V. Daelli, N. van Rijsbergen, A. Treves

**Affiliations:** Cognitive Neuroscience Sector, SISSA, Trieste, Italy

Adaptation aftereffects are an extensively studied phenomenon that produces a biased perception following sensory exposure to an adapting stimulus. This effect has been shown to occur at all levels of visual processing. Recent studies have applied an aftereffects paradigm to the investigation of the perception of facial categories, using morphed images that create a continuum between two extremes, and suggested that aftereffects are unlikely to derive only from adaptation to low-level features of the images. Given the particular status of faces as stimuli, it is not clear whether adaptation aftereffects are present in perception of other classes of items. Even if adaptation to simple geometrical shapes has been described, this paradigm has never been applied to complex natural images. The first aim of this work was to investigate whether the adaptation aftereffects can be extended to perception and categorization of objects. Using a morphing technique, we morphed images of animate (animals) and inanimate objects, creating continua of within category (e.g., cat-cat) and between category (e.g., cat-rabbit) stimuli. In order to test whether the similarity between the extremes of the continua is related to the after effects, we obtained a measure of perceptual similarity for the extremes of each pair, investigating in separate sessions the role of shape, texture and meaning. We found significant aftereffects in perceptual discrimination of the morphed images after adaptation to the extremes of the morph continuum. This effect seems to be modulated by the type of similarity rating that the subjects are requested to perform before the adaptation task, suggesting a role of attention to particular feature of the images in the after effects.

# Abstract: PREDICTING FACIAL EXPRESSIONS: ADAPTATION AFTER EFFECTS OF CHANGING EXPRESSIONS

**Authors:** N. van Rijsbergen, N. Furl, A. Treves

**Affiliations:** ^1^SISSA, Trieste, Italy; ^2^Functional Imaging Laboratory, UCL, UK

Prominent models of face processing posit that facial expressions are perceived via a neural pathway specialized for utilizing rapidly time-varying information. We investigated whether expression perception could be altered via adaptation to dynamic expressions, finding that people can use the direction of change in a subtly moving face to predict future emotional expressions, and this prediction influences perception of a target expression presented immediately afterwards. Experiment 1 found sensitivity to the difference between predictive and non predictive face motion, at a short ISI (50 ms), but less so at (350 ms). Experiment 2 contrasted effects of the adaptation aftereffect that heightens the contrast between adaptor and target, which depends on the average expression in the adapting sequence, F{1, 234} = 12.65, *p* = 0.0005) and the effect of predictive change, which depends on direction and, assimilates the target into the sequence, F{1, 214} = 5.28, *p* = 0.03. Experiment 3 tested to see if predictive direction generalised over changes in the identity of the face stimuli, and the location of the stimuli, as predicted by similar representation momentum effects in non face stimuli, (*N* = 23), finding that the predictive effect survived F{1, 1283} = 6.24, *P* = 0.01, but the adaptation aftereffect was greatly attenuated.

# Abstract: ELECTROPHYSIOLOGICAL MARKERS OF HYPERBOLIC DISCOUNTING IN MONEY AND HEALTH: A HIGH-DENSITY ERP STUDY

**Authors:** C. Rawdon, L. Delaney, K. Denny, R. A. Roche

**Affiliations:** ^1^Department of Psychology, NUI Maynooth; ^2^UCD Geary Institute, Dublin

Economics has increasingly attempted to provide solid empirical micro-foundations based on psychology and neuroscience and this has led to a flourishing body of interdisciplinary work (e.g., Glimcher and Rustichini 2004). Economics typically assumes that when delaying gratification the lower weight given to future benefits, the discount rate, declines exponentially. However there is a substantial body of evidence that it declines more rapidly, that is, hyperbolically. Hyperbolic discounting has been utilised to explain a range of phenomena in the behavioural economics literature, particularly in the areas of procrastination (O'Donoghue and Rabin 1999; Brocas and Carrillo 2001). A further issue that has derived from this literature is whether time preferences are domain specific, with both rates of trade and decision processes differing across the domain of the decision. In terms of the neural bases of such decisions, McClure et al. (2004) reported differential activations in limbic, prefrontal and posterior parietal regions when immediate and delayed monetary rewards were contrasted. Here we present behavioral and high-density EEG/ERP data from a task in which participants were asked to make decisions about financial rewards or their health over short and long time-horizons. Participants (*n* = 15) made a button-press response to their preference for an immediate or delayed gain (financial or health), with either a large or small discrepancy existing between these alternatives. Waveform components elicited during the task differentiated between conditions, while dipole source analysis was employed to identify possible cortical generators of the scalp-recorded potentials. This paper is the first to examine potential neurological mechanisms underpinning domain-specific hyperbolic discounting processes, and elucidates on the behavioral and neurocognitive operations involved in this form of decision making.

# Abstract: HIGH-DENSITY ERPS MAY DIFFERENTIATE CONSOLIDATION FROM RECONSOLIDATION PROCESSES IN HUMANS DURING ASSOCIATIVE LEARNING

**Authors:** J. L. Moore, C. Rawdon, R. A. Roche

**Affiliations:** ^1^Department of Psychology, National University of Ireland Maynooth, Maynooth, Co. Kildare, Ireland; ^2^Trinity College Institute of Neuroscience, Dublin, Ireland

The processes of consolidation and reconsolidation constitute a large proportion of current research into memory formation. The evidence in favour of the Consolidation Theory is widespread, both on a cellular and systems level. Research has indicated that consolidation and reconsolidation employ similar mechanisms; both the consolidation and reconsolidation of memory require protein synthesis and glutaminergic input, and both seem to be associated with the hippocampal formation. Despite this, other data argue that the two concepts are entirely separate and individual processes. A task was devised specifically to compare reconsolidation of an existing memory trace and the new consolidation of additional information. An initial study block involved the presentation of sixteen visual pairedassociates. In a subsequent study block, half of the study pairs were combined with an additional stimulus to form a triplet, while the other eight pairs were not associated with a supplementary stimulus. In a final test block, paired versus non-paired stimuli were presented for yes/no recognition. The presentation of the original pair (i.e., reconsolidated memory) was contrasted with presentation of the “new” associates with either element of the original pair (i.e., newly consolidated memory). 128-channel EEG was recorded for each stimulus type and BESA source localisation was employed to identify neural generators associated with the task. Analysis of electrophysiological components elicited for reconsolidated versus newly-consolidated pairs revealed modulation of the memory-related posterior components P3 (250–400 ms), N2 (150–250 ms), and P1 (100–150 ms). These findings help to elucidate the nature of consolidation and reconsolidation processes and address the question of whether they constitute similar or distinct processes.

# Abstract: A STUDY OF THE NEURAL CORRELATES OF SOURCE MEMORY RETRIEVAL IN YOUNG AND OLD PARTICIPANTS USING A HIGH-DENSITY ERPS ARRAY

**Authors:** P. Scanlon

**Affiliations:** NUI Maynooth, Ireland

Failures of source memory (the ability to recall the specific context in which events took place) have been associated with cognitive decline in the elderly, and appear to be more indicative of age-related memory impairment than disruption of item memory. An Opposition Procedure developed by Jacoby and colleagues (Jennings & Jacoby, 1997) tests this source memory capacity by drawing on repetition errors in a word recall task in which novel words are presented repeatedly at 3 differing lags (0, 4 and 16 trials). This task is sensitive to source memory dysfunction, with older adults being found to produce significantly more errors in repetition than healthy younger adults. In this study, we recorded 128-channel EEG from normal healthy participants (*N* = 18; age 20–30) and healthy older adults (*N* = 18; aged 60–70) while they executed the Opposition Task. Behavioral results showed that accuracy decreased significantly (*p* < 0.05) for lag 4 and 16 trials compared to lag 0 trials, with an associated significant increase in reaction times (*p* < 0.05) for the longer lag conditions, replicating the results found by Jennings & Jacoby (1997) across age groups. Older adults showed significantly lower scores and longer RTs than the young adult group. ERP results showed differences in component amplitudes between the age groups and between conditions, with greater frontal scalp positivity in young adults for correct responses to targets over other conditions and greater parietal scalp positivity for all correct responses over incorrect responses. Source analysis showed dipoles near prefrontal lobe, cingulate gyrus and postcentral gyrus.

# Abstract: CORTICAL & SUBCORTICAL BASIS OF SPATIAL MEMORY: IMPLICIT CODING OF LOCATION AND PERCEPTUAL RECOGNITION OF VIEWPOINT

**Authors:** J. Murphy, C. Wynne, R. Roche, S. Commins

**Affiliations:** National University of Ireland, Maynooth, Ireland

Spatial memory is paramount to human existence and fundamental to other types of knowledge. The current study consisted of two experiments designed to examine encoding and retrieval of spatial information using high-density electroencephalography. A computer based “spatial grid task” was used to present stimuli in both experiments. Experiment 1 investigated the discriminatory aspects of a parietal P1 and its sensitivity to rotations of an object array. Twenty participants memorized the locations of objects in an environment. During the recognition test the environment was rotated by 90 degrees left or right or 180 degrees, which required participants to reorient to the new viewpoint. The P1 accompanied these rotations, and after rotation to the left or right, an increase in the amplitude of the P1 was seen in the contralateral hemisphere. We demonstrate a human ability to perceive orientation within an environment, as evidenced by this electrophysiological component's hemispherical dissociation for left/right rotations. Experiment 2 examined whether the coding of spatial location occurred spontaneously, without explicit instruction. Eighteen participants volunteered for an object recognition task in which old/new judgements were required at the test phase. “Old” objects appeared in either their studied location or elsewhere on the spatial grid, resulting in significant differences in the ERP. Behavioural and electrophysiological differences are reported with N2 and P3 component latency negatively affected by relocation. Componentry in the waveform for correctly located objects were ∼100 ms earlier than for incorrectly located objects and distractors. Dipole source models are provided for both experiments.

# TOWARDS A PHYSIOLOGICAL THEORY OF ABSTRACT LEARNING

**Authors:** H. Rey, D. A. Gutnisky, B. S. Zanutto

**Affiliations:** ^1^Institute of Biomedical Engineering, University of Buenos Aires, Argentina; ^2^Department of Neurobiology and Anatomy, University of Texas—Houston Medical School, USA

The capacity to conceptualize contingencies abstractly is an efficient way to save memory resources. In the past, Same/Different (SD) discrimination, the simplest form of abstract learning, was thought to have language training as a necessary condition. However, experimental evidence have shown that this task cannot only be solved by humans, but also by chimpanzees, rhesus and capuchin monkeys, and pigeons. In addition, recent neurophysiological data suggests that neurons in prefrontal cortex of rhesus monkey code abstract rules. Despite these evidence, how neural networks are able to learn abstract rules is largely unknown.

We propose a minimal model that is able to learn the SD task. The model is composed of an input layer, working memory cells, SD cells and response neurons. Working memory cells are connected to SD cells through depressing synapses. Each different stimulus activates a defined subset of working memory cells. The synapses impinging on the SD cells that correspond to activated working memory cells become depressed. A further presentation of the same stimulus will elicit a lower response from these cells indicating that the same stimulus was presented, irrespective to its particular features. In contrast when the stimulus are different, the synapses are not depressed, and the SD cells response are not attenuated. Response neurons pool these neurons to provide the correct response. The model is minimal in the sense that all its components are already described in the literature, although without explicit mention to its relevance for this task. The model provides also a basic building block upon more complicated abstract tasks can be accomplished, such as conceptual matching to sample, learning sequences of stimuli, etc.

# Abstract: A SENSE-LINKAGE FOR METRIC AND LANDMARK INFORMATION IN ANIMALS' SPATIAL REORIENTATION

**Authors:** G. Vallortigara, V. A. Sovrano

**Affiliations:** ^1^Department of Psychology and BRAIN Centre for Neuroscience, University of Triest, Triest, Italy; ^2^Department of General Psychology, University of Padua, Padua, Italy

Disoriented children could use geometric information in combination with featural information to reorient themselves in large but not in small spaces; somewhat similar effects have been found in non-human animals, all on demand for an explanation. We trained young chicks to reorient to find food in a corner of a rectangular room with a distinctive featural cue (a blue wall)—a task similar to that used with children. Then we tested chicks after displacement of the feature on another adjacent wall. In the large enclosure chicks chose the corner that maintained the correct arrangement of the featural cue with respect to sense, whereas in the small enclosure they chose the corner that maintained the correct metric arrangement of the walls with respect to sense. Based on these findings, we propose a simple model that can explain size effects on spatial reorientation.

# Abstract: A MODEL OF THE STORAGE OF CORRELATED MEMORIES AND ITS BEHAVIORAL SIDE-EFFECTS

**Authors:** E. Kropff, A. Treves

**Affiliations:** ^1^SISSA, Trieste, Italy; ^2^CBM, NTNU, Trondheim, Norway

The storage collapse in the presence of correlations has been long last pointed as a major weakness of attractor memory models. We present, however, a simple modification to the standard hebbian learning rule that overcomes this shortage, enabling the storage of any set of correlated representations under the single assumption of independence across neurons, which we further discuss. In comparison with previous studies, the rule uses local information rather than global measures of activity, increasing its biological plausibility. The secondary effects of this rule condense into a new and remarkable property of associative networks: the robustness of a given memory is inverse to the information it carries. This theoretical discovery can be extrapolated to explain functional and architectural properties of the cortex. In first place, we use the robustness rule to derive an explanation of category-specific impairments in the semantic memory system as a consequence of the information distribution across categories, in line with previous qualitative proposals [1, 2]. In second place, we show that correlated memories cannot be acquired on the basis of a one-shot learning paradigm, which meets the proposal [3] and posterior experimental finding [4] that the quintessence of one-shot learning in cortex—the episodic memory loop—is equipped with an un-correlating mechanism (Dentate Gyrus acting on the CA3 memory-layer of hippocampus), possibly the mammalian strategy to avoid such an incompatibility.

## References

[1] Devlin JT, Russell RP, Davis MH (2002). Is there an anatomical basis for category-specificity? Semantic memory studies in PET and fMRI. *Neuropsychologia*.

[2] McRae K, de Sa VR, Seidenberg MS (1997). On the nature and scope of featural representations of word meaning. *Journal of Experimental Psychology: General*.

[3] Treves A, Rolls ET (1992). Computational constraints suggest the need for two distinct input systems to the hippocampal CA3 network. *Hippocampus*.

[4] Leutgeb S, Leutgeb JK, Treves A, Moser M-B, Moser EI (2004). Distinct ensemble codes in hippocampal areas CA3 and CA1. *Science*.

# Abstract: HOW DOES THE CORTEX DO ALL OF THE THINGS THAT IT DOES? TOWARDS A UNIFYING MODEL OF CORTEX

**Authors:** S. Forwood, L. Saksida, T. Bussey

**Affiliations:** Department of Experimental Psychology, University of Cambridge, UK

Current computational ideas of cortical function can have little relation to biological reality. Where models are explicitly based on biology, they often take a modular approach to the functions of the cortex, addressing the functional role of only one cortical structure at a time. The premise for the work presented in this paper is that a useful understanding of the contribution of cortex to cognition can be gained from taking a unitary view of cortical function, regarding all cortex as computationally alike. This paper presents a model of cortex that was designed to include biologically observed properties common to all neocortex. The model was then applied to a variety of tasks which are known to engage the neocortex. These include the development of representations in V1, perceptual learning tasks and stimuli identification and recognition. The model proposes a new means of understanding cortical function that ties learning and behaviour to synptic placticity and neuronal activity.

# Abstract: GUESSING IN THE BRAIN

**Authors:** C. Giorgio, N. Rosemarie

**Affiliations:** Institut des Sciences Congnitives, Bron, France

We used fMRI for investigating human mental processes related to reasoning about others' behavior. We implemented an experimental task called “Guessing game” (Nagel, 1995, American Economic Review). In this game subjects have to guess a number in a specified interval, for example, [0, 100] which is closest to the mean of the numbers announced by others players multiplied by a fixed and known parameter *p*. If 0 ≤ *p* < 1, then the theoretical solution (Nash equilibrium) of the game is for everybody to guess “0”. This is the optimal solution if everybody is rational and think that everybody is rational etc. This particular task might induce different levels (orders) of beliefs reasoning, that is, reasoning about others' reasoning. A first-order belief corresponds to think that other players guess (uniform) randomly and thus best response (e.g., if *p* = 2/3) is 50*2/3 = 33.333; a second-order corresponds to think that other players guess using a first-order beliefs (33.333*2/3 = 22.222). fMRI data indicate enhanced activity in paracingulate and orbitofrontal cortex during choice related with higher level of reasoning, thus with increasing social complexity.

# Abstract: THE ROLE OF THE P1 AND ALPHA OSCILLATIONS FOR PICTURE PERCEPTION

**Authors:** R. Freunberger, Y. Höller, B. Griesmayr, P. Sauseng, W. Gruber, W. Klimesch

**Affiliations:** ^1^Department of Physiological Psychology, University of Salzburg; ^2^Institute of Medical Psychology, University of Tübingen, Germany

It was tested whether the P1 component of the event-related potential (ERP) in the human EEG is an indicator of early visual categorization processes. Subjects were instructed to judge visual stimuli (photographs and distorted images) as “living” or “non-living”. P1 was analyzed with respect to its frequency characteristic. Results revealed that crystallized images showed pronounced P1 amplitudes when compared with living and non-living objects. In the time-window of the P1 strong evoked alpha oscillations (at around 10 Hz) were found showing similar effects as the ERP's. We concluded that P1 is an early correlate of categorization processes and is in part generated by alpha oscillations. There is a relationship between early ERP components and oscillatory behaviour.

# Abstract: EXPLAINING THE EFFECTS OF ATTENTION ON LEXICAL PROCESSES USING A SINGLE HEBBIAN NEURONAL MODEL OF THE LANGUAGE CORTEX

**Authors:** M. Garagnani, T. Wennekers, F. Pulvermüller

**Affiliations:** ^1^MRC Cognition & Brain Sciences Unit Cambridge, UK; ^2^Centre for Theoretical and Computational Neuroscience, University of Plymouth Plymouth, UK

Meaningful familiar stimuli and senseless unknown materials lead to different patterns of brain activation. The major neurophysiological response indexing “sense” is the N400, a late event-related brain response larger for senseless materials (e.g., meaningless pseudowords) than for matched meaningful words. More recently, however, early differences have also been recorded, using Magneto- and Electro-Encephalography (MEG and EEG)—for example, in the Mismatch Negativity (MMN, latency 100–250 ms). The MMN is elicited even when subjects are heavily distracted and, in this case, is larger for words than for pseudowords, thus exhibiting the reverse pattern seen for the N400. So far, no single account has been able to explain these seemingly contradictory results.

We implemented a neuroanatomically grounded neural-network model of the left-perisylvian language cortex and used it to simulate brain processes of early language acquisition. The network was repeatedly confronted with activation patterns and allowed to adapt by means of Hebbian (longtermpotentiation and depression)mechanisms: we observed the formation of input-specific neuronal circuits, i.e., sets of strongly interconnected neurons distributed over a range of areas which responded only to known patterns (“words”). The trained model was then used to simulate the neurophysiological response of the language cortex to words and senseless pseudowords stimuli. We found that variation of the amount of global inhibition of the network (which we interpret as the model correlate of attention) modulated the simulated brain response to words and pseudowords, producing either an N400- or an MMN-like response depending on the amount of global inhibition (or available attentional resources).

Our model (1) demonstrates the viability of purely Hebbian, associative learning in a multi-layered network architecture, (2) offers a unifying explanatory account for seemingly inconsistent experimental observations, and (3) makes clear predictions on the effects of attention on latency and magnitude of ERPs to lexical items. Such predictions have been confirmed by recent experimental evidence.

# Abstract: MEMORY TRACE STABILIZATION LEADS TO LARGE-SCALE CHANGES IN THE RETRIEVAL NETWORK: A FUNCTIONAL MRI STUDY ON ASSOCIATIVE MEMORY

**Authors:** T. Atsuko, Ingrid L. C. Nieuwenhuis, R. Mark, M. P. Karl, J. Ole, F. Guillén

**Affiliations:** ^1^FC Donders Centre for Cognitive Neuroimaging, Radboud University Nijmegen, Nijmegen, The Netherlands; ^2^Department of Neurology, Radboud University Nijmegen, Nijmegen, The Netherlands

Spaced learning with time to consolidate is known as an effective learning strategy resulting in stabilization of the memory traces. However, little is known about the neural correlates of trace stabilization, especially in humans. In the present fMRI study, we trained subjects on associating a face (40 faces in each condition) to a given location (one of the eight locations on the screen). The task of the subjects at test was to retrieve the associated location when a face cue was presented. We contrasted the retrieval activity of two memory conditions, one learned in a massed style and tested on the same day of learning (i.e., the labile condition) and another learned in a spaced scheme over the course of one week (i.e., the stabilized condition). In accord with our expectation, both sets of associations were retrieved equally well; however, the retrieval of stabilized association was faster (stabilized: reaction time = 936 ± 26.1 ms; labile: reaction time = 1029 ± 35.0 ms; *n* = 22, *t* = 4.520, *P* < 0.001). Cuedrecall of stabilized as compared to labile associations was accompanied by increased activity in the precuneus, the ventromedial prefrontal cortex, the bilateral temporal pole and left temporo-parietal junction. Conversely, memory representational areas such as the fusiform gyrus for faces and the posterior parietal cortex for locations did not change their activity with stabilization. Thus stabilization is expressed as changes in regions linking the representational areas. The activation changes in the precuneus, which also showed increase in connectivity with the fusiform area with stabilization, are likely to reflect a more direct retrieval of the spatial components of the task. As for the activation increase in the ventromedial prefrontal cortex, the change might reflect the succession of the hippocampal role in linking distributed neocortical representations.

## Figures and Tables

**Figure 1:** 

# Abstract: MEMORY TRACE STABILIZATION IS REFLECTED BY CHANGES IN THETA AND GAMMA ACTIVITY: AN MEG STUDY ON ASSOCIATIVE MEMORY

**Authors:** I. L. Nieuwenhuis, A. Takashima, G. Fernández, O. Jensen

**Affiliations:** ^1^FC Donders Centre for Cognitive Neuroimaging, Radboud University Nijmegen, Nijmegen, The Netherlands; ^2^Department of Neurology, Radboud University Nijmegen, Nijmegen, The Netherlands

From animal and human physiological research it is known that oscillatory activity in the theta (5–8 Hz) and gamma (30–120 Hz) band reflects successful memory retrieval. Little is known about how this oscillatory activity changes when memories stabilizes with time and training. We used magnetoencephalography (MEG) to investigate the changes in neocortical activity with memory stabilization. 22 subjects were trained on two sets of face-place associations. The first set was learned distributed over the week prior to the MEG measurements (‘stabilized’) and the second set was intensively learned just prior to the measurement (‘labile’). In the retrieval sessions the subjects had to recall the place associated with a given face. Retrieval was faster for stabilized than labile associations but equally accurate. The eventrelated fields (ERFs) showed a faster onset for stabilized associations in frontal sensors. Additionally sustained ERFs were larger for labile than stabilized associations over frontal, temporal and parietal areas. Time-frequency representations of power revealed an increase in gamma band (60–120 Hz) activity around the parieto-occipital sulcus during recall. This increase was higher for labile than stabilized associations. Theta power (∼6 Hz) also increased during recall spreading from posterior to anterior regions. The increase was higher for stabilized than labile associations, first over right temporal and later over right parieto-central areas. The faster recall and smaller in ERFs for stabilized associations point to neuronal processes for recall becoming more efficient with memory stabilization. Our data suggests that the recall of labile face-space associations relies on a strategy engaging visual areas (e.g., ‘mind's eye’) as reflect by the gamma activity. Retrieval of stabilized memories relies to a higher degree on the classical association areas in temporal and parietal areas as reflected by the theta activity.

# Abstract: EFFECTS OF AGING ON ATTENTIONAL MODULATION OF CORTICAL PROCESSING

**Authors:** D. R. Eve, W. S. Taylor, D. Matthew, C. Frederick

**Affiliations:** Department of Psychology University of Toronto, Canada

Selective attention enables us to process behaviourally relevant information from the environment while simultaneously filtering out the irrelevant. Given that the efficiency of selective attention is presumed to decline in the course of normal aging, the present study examined how the processing of irrelevant information differs between younger and older adults. Participants performed a gender judgment task comprised of superimposed face (target) and house (ignored) stimuli. It was hypothesized that older adults would unintentionally encode more task irrelevant (house) information. Some of the stimuli were presented multiple times non-consecutively, whereas others were presented only once. On a subsequent implicit recognition test, participants matched the previously seen faces with either the correct (previously seen) house or a novel house. Performance accuracy on the implicit recognition test provided a measure of unintentional encoding of irrelevant (house) information during the gender judgment task. Older adults exhibited significantly better accuracy on the implicit recognition test compared to the younger group for the repeated irrelevant stimuli from the gender judgment task. However, there was no difference detected in explicit recognition for the faces or houses between groups. The results suggest that older adults enhanced implicit knowledge of irrelevant information reflect impaired selective attentional control. We are currently using functional magnetic resonance imaging (fMRI) to examine potential age-related changes in attentional modulation of cortical processing in the parahippocampal place area (PPA). We also hypothesize that these results will reveal attentional network changes that predict the behavioural correlates of impaired selective attention in older adults. Supported by: Canadian Institutes of Health Research grant to E.D.R.

# Abstract: VOLUNTARY ORIENTING INFLUENCES CONFLICT RESOLUTION: AN ERP AND SOURCE RECONSTRUCTION STUDY

**Authors:** V. Rossi, A. M. Proverbio, A. Zani

**Affiliations:** ^1^Department of Psychology, University of Milano-Bicocca, Milano, Italy; ^2^Institute of Molecular Bioimaging and Physiology, CNR, Segrate-MI, Italy

It is renown that visual orienting and executive control systems are based on functional separate but partly overlapping circuits. A simple task has been proposed to test the independence of these sub-systems: the Attention Network Test (ANT), in which targets falling above or below fixation are presented under different cueing conditions (Spatially valid Cue, SC, Central neutral Cue, CC, No Cue, NC), surrounded by Congruent or Incongruent Flankers (CF, IF). Previous behavioral studies using ANT found interactions between voluntary orienting and executive control systems (during orienting, SC, the flankers effect was smaller than when non-orienting), although the anatomical counterpart of this interaction has not been clearly indicated. To investigate the spatial and temporal links between orienting and executive control we administered the ANT to 19 adults while recording their EEG from 128 channels. Offline grandaverage ERP and Difference Waves (DW) were computed as a function of task (SC, CC, NC) and congruency (CF, IF). We also carried out source reconstruction by means of LORETA. In accordance with literature, task and congruency exerted an interactive effect. LORETA computed for the SCCC DW showed the activation of both the areas considered responsible for attentional orienting and of Anterior Cingulate Cortex (ACC, BA 32), currently thought to be involved in conflict resolution and monitoring processes. Particularly, the ACC activation already arose during C1 latency range (60–100 ms after target presentation) and maximized during frontal N2 and P300 time range (750–1100 ms). We did not observe this pattern in the CC-NC DW LORETA. Taken together, these results suggest a plausible neural correlate of the lower cost for incongruency in SC, in which the conflict monitoring system is plausibly pre-activated by the orienting network.

# Abstract: HOW TO GIVE ELMAN BP SOME REINFORCEMENT FLAVOUR

**Authors:** A. Grüning

**Affiliations:** Cognitive Neuroscience Sector - SISSA, Italy

The back-propagation (BP) training scheme is widely used for training network models besides its well known technical and biological short-comings. We present a method to make the BP training scheme more acceptable from a biological and cognitive point of view in cognitively motivated prediction tasks overcoming one of its major drawbacks. Traditionally, recurrent neural networks in symbolic time series prediction (e.g., language) are trained with gradient decent based learning algorithms, notably with backpropagation (BP) through time. A major drawback for the biological plausibility of BP is that it is a supervised scheme in which a teacher has to provide a fully specified target answer. Yet, agents in natural environments often receive a summary feed-back about the degree of success or failure only, a view adopted in reinforcement learning schemes. We show that for simple recurrent networks in prediction tasks for which there is a probability interpretation of the network's output vector, Elman BP can be reimplemented as a reinforcement learning scheme for which the expected weight updates agree with the ones from traditional Elman BP.

# Abstract: TYPES OF APHASIA IN CHILDREN WITH TRAUMATIC BRAIN INJURY

**Authors:** M. G. Vukovic, J. R. Vuksanovic, I. Z. Vukovic

**Affiliations:** ^1^Faculty of Special Education and Rehabilitation, University of Belgrade, Belgrade, Serbia; ^2^Helth Centre of Zemun, Belgrade, Serbia; ^3^University of Belgrade, Belgrade, Serbia

Brain injury is the most common cause of acquired aphasia in children: traumatic aphasia includes 30% of all cases of childhood aphasia. In this paper we present eight righthanded patients suffering from traumatic aphasia: 4 boys and 4 girls aged 9–11. CT brain scan revealed a diffuse lesion in 4 cases, while 4 cases had focal brain lesion (3 patients in the frontoparietal region, one patient had a lesion in the temporoparietal region, and one in the area of the parietal region). In the assessment of language functions The Boston Diagnostic Aphasia Examination and the Boston naming test were used. The results have shown nonfluent aphasia in six cases, while fluent aphasia was found in two children. The correlation of brain lesion localization and the type of aphasia are discussed in this paper, as well as the recovery of language functions.

# Abstract: EPISODIC-LIKE MEMORY IMPAIRMENT IN SUBTYPES OF MILD COGNITIVE IMPAIRMENT

**Authors:** K. Vlcek, J. Laczo, O. Vajnerova, M. Ort, M. Vyhnalek, J. Hort

**Affiliations:** ^1^Department of Neurophysiology of Memory, Academy of Sciences, Czech Republic; ^2^Charles University, 2nd School of Medicine, Prague, Czech Republic

Alzheimer's disease (AD) is associated with a loss of episodic memory. It is preceded by a stage of cognitive impairment without dementia, referred to as mild cognitive impairment (MCI). Episodic-like memory concept was developed by Clayton and Dickinson (1998) as the memory for information about ‘where’ a unique event or episode took place, ‘what’ occurred during the episode, and ‘when’ the episode happened. We developed non-verbal test of episodic-like memory for human presented on computer. The test consists of a presentation and a testing phase. In the presentation phase, the subject is shown a computer screen with several abstract pictures on predefined places on the right part of the screen and an empty open chest on the left. S/he is instructed to drag, using the computer mouse pointer, the pictures from the predefined places in a given order slowly into the chest. The subjects should memorize both the order and the position of each picture. After about 10 minutes break, the subject should drag the pictures in the same order to the correct position. Successively, memory for position and order of three, five and seven pictures was tested. We evaluated separately the errors in giving order of the pictures, position of the pictures and order of the predefined positions. Comparison was made among groups diagnosed with non-amnestic, amnestic single domain and amnestic multidomain subtypes of MCI, early stage of AD and a control group. The nonamnestic MCI subjects were impaired in the order of the object, while the amnestic multidomain MCI subjects were impaired in the object position. The amnestic multidomain and AD patients were impaired in both. These results can be useful in predicting the outcome of different MCI subtypes.

# Abstract: THE ROLE OF POSTERIOR THALAMUS FOR OUR CONTROL OF UPRIGHT BODY POSTURE

**Authors:** L. F. Ticini, M. Fruhmann Berger, L. Johannsen, D. Broetz, U. Klose, H.-O. Karnath

**Affiliations:** ^1^Section Neuropsychology, Center of Neurology, Hertie-Institute for Clinical Brain Research, University of Tübingen, Tübingen, Germany; ^2^Section ExperimentalMR of the CNS, Department of Neuroradiology, University of Tübingen, Tübingen, Germany

Pusher syndrome is a specific disorder of postural orientation induced by lesions centering on the posterior thalamus. The patients have a disturbed perception of their own body orientation. They experience their upright body posture tilted about 20° to the ipsilesional side. The aim of the present study was to investigate whether posterior thalamic strokes in patients with pusher syndrome cause abnormal perfusion in sub/cortical areas remote from the site of the lesion. In a group of four patients with pusher syndrome and six controls, we studied the pattern of (structurally intact but) dysfunctional sub/cortical tissue induced by these strokes using perfusion-weighted MR imaging (PWI). The group analyses revealed that patients with pusher syndrome did not show distinctive perfusion abnormalities in addition to their lesions in the posterior thalamus. We conclude that pusher syndrome is not caused by dysfunction of structurally intact sub/cortical areas but rather is caused by the neuronal loss in the posterior thalamus itself. The finding is consistent with recent anatomical studies that suggested a fundamental role of the posterior thalamus for our control of upright body posture.

# Abstract: A COMPARISON OF COGNITIVE FUNCTIONS BETWEEN NON-DEMENTED PARKINSON'S DISEASE PATIENTS OF DIFFERENT MOTOR SUBTYPES

**Authors:** E. Lyros, L. Messinis, P. Papathanasopoulos

**Affiliations:** ^1^Department of Neurology, University of Patras Medical School, Greece; ^2^Clinical Neuropsychological Unit, Department of Neurology, University of Patras Medical School, Greece; ^3^Department of Neurology, University of Patras Medical School, Greece

Parkinson's disease (PD) is characterized by great variety in terms of both motor and cognitive symptoms. Two major clinical subtypes of PD have been identified; the postural instability and gait disorder (PIGD) - dominant subtype and the tremor-dominant (TD) subtype based on the relative predominance of these symptoms, with the remaining PD patients classified as “indeterminate” subtype. The PIGD subtype has been shown to be associated with worse clinical prognosis and higher occurrence of dementia compared to the non-PIGD subtypes. In the present study, two groups of nondemented PD patients, one composed of 15 PIGD patients and one composed of 15 non-PIGD ones, as well as a group of 15 healthy controls performed a set of tasks to evaluate different aspects of cognition previously found to be affected in PD. All groups were matched regarding potential confounders of neuropsychological performance. Patients were assessed on treatment with levodopa, therefore the scores obtained are thought to represent residual nondopaminergic deficiency. Statistical analysis revealed no significant differences between the two groups of patients in the performances in any of the administered tests, indicating against the presence of major differences in cognitive functions between non-demented PIGD-dominant PD patients and their counterparts to account for the higher incidence of dementia in the former group. However, there was a tendency towards a differential pattern of cognitive dysfunction when each patient group was compared to controls. The PIGD group had slower performance in a test of psychomotor speed and cognitive flexibility, while the non-PIGD group provided worse scores in measures of verbal learning and visuospatial perception. Pedunculopontine nucleus (PPN) lesions for the PIGD group and cerebello-thalamocortical activation for the tremor-dominant variant that mainly compounds the non-PIGD group are primarily considered.

# Abstract: TASK-INDEPENDENT NEGLECT IN NEAR AND FAR SPACE–A COMPARISON OF LINE BISECTION AND CANCELLATION PERFORMANCE IN RIGHT BRAIN DAMAGED PATIENTS

**Authors:** L. Aimola, I. Schindler, A. Venneri

**Affiliations:** Department of Psychology, University of Hull, Uk

Case studies of patients with neglect and neurophysiological findings suggest a distinct organization of attentional mechanisms for near (peripersonal) and far (extrapersonal) space (Vuilleumier, 1999; Halligan, 1991; Rizzolatti et al. 1983). To date, the effect of distance on the severity of neglect has been shown mainly with line bisection paradigms only (Halligan, 1991; Keller, 2005) and it is still unclear whether neglect for near and far space represents a task independent phenomenon. In the present study, 35 right hemisphere damaged patients with and without neglect were recruited. All the patients performed a cancellation and a line bisection task in near (40 cm) and far (350 cm) space respectively. The stimuli were presented in front of each subject and aligned with the body midline. The tasks were performed by using a pen in near space and a laser pointer in far space. The cancellation task (bell test) required to detect 35 targets along with 280 distractors whereas in the bisection task patients were asked to mark the middle of three lines placed respectively on the right, on the centre and on the left of the display. The results showed that in both tasks, the performance of the neglect patients was significantly worse compared to patients without neglect and that space distance affected only the performance of the neglect group. In fact, the performance of the latter improved significantly in far space compared to near space irrespective of the type of task. The results suggest a dissociation between neglect for near and far space. Thus it is possible that different neuronal networks may be recruited for allocating attention to objects within and beyond grasping distance. Moreover, the similar pattern of reduced neglect in far as compared to near space in both tasks indicates task-independent attention processing within near and far space.

# Abstract: GROUP ANALYSIS OF POLARITY DIFFERENCES IN PATTERN-ONSET VEPS

**Authors:** M. Del Zotto, A. Zani, A. M. Proverbio

**Affiliations:** ^1^Department of Psychology, University Milano-Bicocca, Milan, Italy; ^2^Institute of Molecular Bioimaging and Physiology, CNR, Milan-Segrate, Italy

It is know that C1 component of VEPs is strongly affected by stimulus location, as indicated by a polarity reversal for upper vs lower field. While it is supposed that C1 reflects the sensory processing of stimulus features at the primary visual cortex, not much is known about the timing of modulatory attention effects at an early sensory stage, both for spatialand object-based stimulus features. The goal of the research was to investigate early attention effects by recording patternonset VEPs to familiar objects in a conjoined space-based. Possible differences in the polarity of attention effects as a function of individual differences in the morphology of C1 and P1 component were monitored. EEG was recorded from 30 scalp sites in 18 right-handed individuals. The task consisted in paying attention and responding to a conjunction of space and object features. Stimuli were black and white drawings of familiar objects and animals, presented slightly lateralized in the right or left hemi-field. Independent of task factors, participants were subdivided in two groups, on the base of their VEP morphology. A group exhibited a prominent N80 at mesial occipital sites, while, in the same latency range, the other group exhibited a prominent P80. ERP results showed that the amplitude and polarity of spatial and nonspatial attention effects were affected by VEP morphology. In fact, attention effects always resulted in an increased positivity for the P80 group (both at C1 and P1 level), while shape relevance was associated with an enhanced N80 and P1 responses in the N80 group. RTs were comparable across the 2 groups, thus suggesting an independence of morphology from task related factors. These data provide evidence of an inversion of attention effects, besides inversion of C1 polarity, in people exhibiting a negative C1 at mesial occipital sites.

# Abstract: DIFFERENT LANGUAGES IN ONE BRAIN: AN ERP STUDY ON SIMULTANEOUS INTERPRETERS

**Authors:** R. Adorni, A. Zani, A. M. Proverbio

**Affiliations:** ^1^Department of Psychology, University of Milano-Bicocca, Milan, Italy; ^2^Institute of Molecular Bioimaging and Physiology, CNR, Milan-Segrate, Italy

The aim of the present study was to investigate the neurofunctional mechanisms of orthographic analysis and lexical access in multilingual people. Professional simultaneous interpreters took part to the experiment. In order to distinguish the role played by the age of acquisition and proficiency of a certain language, we selected fifteen Italian native speakers with an excellent proficiency in English (L2) and an intermediate knowledge of German (L3). 780 linguistic stimuli were created. They consisted of words (balanced in terms of imageability and frequency of use) and legal pseudowords in 3 different languages (Italian, English and German). All stimuli were balanced in terms of length and position of target letter (beginning, middle or end of stimulus). The task consisted in responding to the appearance of given target letter. EEG was recorded from 30 scalp sites at a sampling rate of 512 Hz. ERPs were time-locked to stimulus onset. Both reaction times (RTs) and electrophysiological data indicated that the difference between words and legal pseudo-words (the so called “word superiority effect”) was modulated by language age of acquisition and proficiency. Words were discriminated from pseudo-words as early as 150 ms with larger N1 responses to words over the left inferior posterior occipital area, possibly indexing the activity of the so-called VWFA, only in the native language (L1). At later processing stages (since 250 ms), corresponding to deeper linguistic processing, we observed a clear difference in the brain response to languages mastered with a different degree of proficiency (namely English vs. German), both at posterior (N2 and N3 component) and anterior (LPN component) electrode sites areas. These data show how ERPs can dissociate age of acquisition from proficiency, and be predictive of a person linguistic competence.

# Abstract: THE INFLUENCE OF IMMEDIATE FEEDBACK ON ASSOCIATIVE LEARNING IN CHILDREN: COMBINING EDUCATIONAL AND NEUROSCIENCE RESEARCH

**Authors:** J. Özyurt, A. Anschuetz, S. Puschmann, B. Moschner, C. M. Thiel

**Affiliations:** ^1^Cognitive Neurobiology, IBU, University of Oldenburg, 26111 Oldenburg, Germany; ^2^Department of Education, University of Oldenburg, 26111 Oldenburg, Germany

The role of feedback on learning is one of the major topics in educational research. Little is known about the neural and behavioral effects of feedback on subsequent information processing and the role individual differences may play. To investigate these questions, the present project aims to combine educational and neuroscience research. An associative learning paradigm was used both in an fMRI study (Siemens Magnetom MRI scanner, 1.5T, *N* = 40) and in a parallel behavioural study with a much larger sample of children (*N* = 138). Each trial consisted of four object-name associations, one of which had to be retrieved after a variable delay period. Children (10 to 12 yrs) were randomly assigned either to a group receiving corrective feedback after retrieval or to a group receiving no feedback. In the behavioural study children underwent additional testing assessing intelligence, memory capacity, self-concept and other psychological variables.

Preliminary data of the fMRI study with 12 children receiving corrective feedback indicate that trials following negative feedback elicited stronger encoding-related activity in occipital regions as compared to trials following positive feedback. Retrieval eliciting positive as compared to negative feedback was associated with stronger activity in occipital brain regions and the caudate nucleus while negative feedback as compared to positive feedback increased neural activity in the anterior cingulate and inferior operculum. These results in children are consistent with prior evidence in adults that suggest that the anterior cingulate cortex responds to negative feedback while the ventral striatum shows increased activity with positive feedback. Further, the increased neural activity in occipital brain regions after trials with negative feedback might reflect heightened attention to the following object-name association in order to improve performance. First exploratory analysis of the behavioural data (*N*=131) revealed no significant differences in error rates between the two feedback groups. Overall, a positive self-concept concerning physical attractiveness and school achievement in German language (but not in mathematics) correlated positively with success in task performance.

# Abstract: EVALUATING PEOPLE: ROLE OF AMYGDALA, STRIATUM, AND PREFRONTAL CORTEX

**Authors:** J. Freeman, J. Mitchell, J. Uleman, E. Phelps, D. Schiller

**Affiliations:** ^1^Psychology Department, New York University, NY, USA; ^2^Psychology Department, Harvard University, UK; ^3^Center for Neural Science, New York University, NY, USA

Social information processing is regarded as a highly complex cognitive effort engaging a variety of brain areas. Specifically, the process of impression formation has been isolated primarily to the medial prefrontal cortex (PFC), but it is still unknown what particular mechanisms underlie this. To address this question, we tested which brain regions were employed while human subjects used different types of information to form impressions of other people. We were particularly interested in whether impression formation might also recruit areas implicated in emotional learning and value encoding. During fMRI scanning, subjects were presented with novel target persons. In each presentation, a face was displayed while 3 negative and 3 positive person-descriptive sentences were introduced successively. Following these, subjects were instructed to form an impression of targets by indicating their evaluation using an 8-point valence scale. These responses were used to determine which information was impression-relevant (for (for example negative sentences for a negativelye-valuated target) and impression-irrelevant (for example positive sentences for a negatively-evaluated target). We then looked for brain areas that distinguished impression-relevant from impression-irrelevant information. Extending prior research, we found that the dorsomedial PFC was engaged during the presentation of social information but did not respond as a function of impression relevance. Interestingly, the amygdala, striatum and ventromedial PFC showed such dissociation, exhibiting stronger responding to impression-relevant information. While the amygdala has long been associated with the learning of aversive outcomes and the striatum largely implicated in reward learning, our results suggest that these and PFC areas work in tandem to ascribe subjective value to relevant social information. This integrated neural activity may provide a manner by which impressions are adaptively formed in a social context.

# Abstract: CEREBRAL CORTEX GREY MATTER CHANGES IN PATIENTS WITH FOCAL CEREBELLAR LESION: A VOXEL BASED MORPHOMETRY STUDY

**Authors:** S. Clausi, M. Bozzali, M. G. Leggio, G. Hagberg, M. Di Paola, M. Molinari

**Affiliations:** ^1^IRCCS Santa Lucia Foundation, Rome, Italy; ^2^Neuroimaging Laboratory, IRCCS Santa Lucia Foundation, Rome, Italy; ^3^Department of Psychology, University of Rome “La Sapienza”, Rome, Italy

Previous behavioural studies indicate specific cognitive dysfunctions in patients with cerebellar lesions. Consistently, several functional imaging studies in healthy subjects demonstrate direct involvement of the cerebellum in different cognitive tasks. However, interactions between the cerebellum and cerebral areas still remains largely debated. Aim of this study was to assess, using MRI and voxel based morphometry (VBM), whether chronic focal cerebellar lesion may result in regional changes of specific cortical areas. Eighteen patients with a stabilized cerebellar lesion (11 on the right and 7 on the left side) and 18 sex and age matched healthy controls were recruited. All subjects underwent an extensive neuropsychological assessment and an MRI study. T2-weighted scans and T1-weighted volumes were collected from each subject. With the exception of the cerebellar damage, none of the patients had any additional macroscopic abnormality on T2- weighted scans. After masking the cerebellar lesions, T1-volumes were post-processed according to an optimized VBM protocol using SPM2. Gray matter (GM) maps were produced for each subject and group comparisons were performed, on a voxel-by-voxel basis, to assess regional differences in GM density between patients and controls. Compared with controls, patients with right cerebellar damage showed a bilateral pattern of reduced GM density, mainly involving the left frontal and temporal lobe. Present study indicates that specific GM abnormalities of the cerebral cortex may be detected in patients with focal cerebellar damage. These results must be analyzed in light of the known anatomical organization of the cerebro-cerebellar loops as well as referring to the pattern of the cognitive impairments observed after focal cerebellar lesions.

# Abstract: PATTERNS OF EXECUTIVE DISORDER IN OBSESSIVE-COMPULSIVE DISORDER

**Authors:** G. Y. Demeter, M. Racsmány, K. Csigó, A. Harsányi

**Affiliations:** ^1^Department of Cognitive Science, Budapest University of Technology and Economics, Hungary; ^2^Research Group on Cognitive Science, Hungarian Academy of Sciences—Budapest University of Technology and Economics, Hungary; ^3^Department of Psychiatry, Gyula Nyírö Hospital, Budapest, Hungary

Disorder of the executive system is generally thought to be the main underlying cognitive factor of symptoms in OCD. However, the results are contradictory as many studies have found less impaired or intact performance on traditional executive neuropsychological tasks by OCD patients. The aim of this study was to find specific patterns of impairment in the executive system in a properly diagnosed clinical sample of OCD patients. To fulfil this aim we used a recent psychometric model proposed by Miyake, et al. (2000) as a theoretical framework which states that traditional executive tasks loading different executive subcomponents. According to Miyake et al.'s (2000) analysis there are three main executive functions: inhibition, modality specific updating and shifting-monitoring, and traditional neuropsychological tasks differently tapping these functions. We applied four widely used neuropsychological tasks: Corsi blocks tapping task, digit span forward task, the Stroop task, and the Wisconsin Card Sorting Task (WCST), as they thought to be load more or less independently one these executive functions. In our study 35 OCD patients diagnosed according to DSM-IV criteria and matched healthy control subjects were involved. The OCD group performed significantly worst in all of the executive tasks. In a second set of analysis OCD patients were categorized on the basis of their symptoms and labelled these subgroups as obsessive, compulsive and mixed. The results show that comparing to healthy controls only the so called compulsive subgroup performed significantly lower on the Stroop Task and produced significantly more preservative errors on the WCST. In our view this result show that compulsive subgroup is the only one produced impaired performance on tasks of inhibition and shifting components of the executive system, while the updating component is injured in all three subgroups.

# Abstract: GROWTH HORMONE SECRETION IS ASSOCIATED WITH COGNITIVE PERFORMANCE IN OLDER MEN WITH MILD COGNITIVE DEFECTS

**Authors:** E. H. Quik, E. B. Conemans, G. D. Valk, L. J. Kenemans, H. P. Koppeschaar, S. P. van Dam

**Affiliations:** ^1^Department of Psychopharmacology, Utrecht University, Utrecht, The Netherlands; ^2^Department of Clinical Endocrinology, University Medical Center Utrecht, Utrecht, The Netherlands; ^3^University Medical Center Utrecht, Utrecht, The Netherlands; ^4^Department Of Internal Medicine, Onze Lieve Vrouwe Gasthuis, Amsterdam, The Netherlands

Background and Aim: Reduced growth hormone (GH) secretion and circulating insulin-like growth factor-I (IGFI) levels are associated with deteriorated cognitive performance. As GH secretion decreases with age, this may contribute to cognitive changes associated with ageing. We evaluated the relation between GH secretion and cognition in older men. We focused on correlations between GH secretion and performance on cognitive tests. Furthermore, we recorded cortical brain activity using event-related potentials (ERPs) in order to assess underlying neurophysiological mechanisms. Subjects and Methods: We studied 17 healthy male subjects (age 50–78 years). Cognitive function was assessed by standardized neuropsychological tests, in particular specific tests either sensitive or insensitive to ageing. ERPs were recorded during a go/ no go selective attention task. GH secretion was assessed by a GHRH-GHRP-6 test and by measurement of plasma IGF-I. Results: GH peak and GH area under the curve (AUC) were significantly correlated with the number of targets detected in the selective attention task. Speed of responding (mean reaction time) correlated with IGF-1, with GH peak, and with GH AUC. GH peak significantly correlated with the performance on the Trailmaking task A. Furthermore, the Number-digit span item scores correlated significantly with the GH AUC. No correlation was observed between GH secretion and ERP data, in particular N2b, which reflects attention related cortical activity. Conclusion: Cognitive performance in elderly men, as far as it concerns target detection, speed of responding, short-term memory, working memory, attention, processing speed, and planning behavior, is associated with GH secretion. GH secretion was not significantly associated with attention-related brain potentials, which we previously found to be compromised in childhood onset GH deficient patients

# Abstract: LEXICAL LEARNING: DISSIMILAR ROLES OF CONSONANTS AND VOWELS

**Authors:** E. Vinnik, M. Nespor

**Affiliations:** ^1^SISSA, Triestra, Italy; ^2^University of Ferrara, Ferrara, Italy

Existing models of lexicon acquisition usually include phonemes, as units of speech perception. Traditionally no distinction between consonants and vowels is made, despite a substantial body of evidence that they serve different roles in language: consonants contribute more to the identification of lexical items (Nespor et al., 2003 and literature cited therein; Bonatti et al., 2005), whereas vowels aremore important for rule learning, specifically syntactic rules (Toro et al., submitted). The aim of our study is to test the possibility that consonants receive special attention (compared to vowels) during lexical learning. 3 groups of subjects performed an oddball detection task, where the oddball was a change of the first phoneme in trisyllabic pseudowords either a consonant or a vowel. The groups differed in an additional task: (1) memorization of the association between words and pictures (word learning group), (2) memorization of pictures (attention control) or (3) passive looking at a checkerboard (control group). Reaction times were measured starting from the onset of the oddball. We found a significant interaction between phonemic category either vocalic or consonantal-and condition, namely in the word learning task a change of consonant was more easily identified. Our results provide new evidence that different roles of consonants and vowels can be seen already at the level of speech perception. We propose to integrate the functional distinction between consonants and vowels both into models of speech perception and models of lexical learning.

# Abstract: FORGETTING RATE OF TOPOGRAPHICAL KNOWLEDGE IN HUMANS IN ABSENCE OF RE-CONSOLIDATION

**Authors:** L. Latini Corazzini, C. Thinus-Blanc, M. P. Nesa, G. C. Geminiani, P. Péruch

**Affiliations:** ^1^Departmento di Psicologia, Università degli Studi di Torino, Torino, Italy; ^2^INSERM U-751 Epilepsie & Cognition, Faculté de Médecine de la Timone, Marseille, France

Topographical knowledge is necessary for efficient navigation toward unseen goals. Route knowledge corresponds to the memory trace of the sequence of landmarks encountered along a specific route, while survey knowledge allows direct access to the global layout of an environment. In normal humans, the few available data on the retention of topographical memory shown no systematic decline over months or years. In the present experiment, two groups of participants elaborated route and survey knowledge during navigation in the same complex virtual environment before performing two route tasks (estimating the number of turns and the route distance between landmarks) and two survey tasks (pointing to unseen landmark sand estimating the straight-line distance between landmarks). Both groups were tested immediately after learning and three months later, while one group was also tested one week and one month later (repeated testing). Following the first testing session, performance was similar in both groups. On subsequent sessions, while performance of the repeated tested group remained stable, it decreased significantly in the non-repeated tested group, especially on route tasks. Survey knowledge was more resistant to decay probably because of its abstract structure. These data are the first to reveal a substantial and selective decline of topographical memory in humans, occurring only if there is no possibility to reactivate knowledge along successive testing phases, that is, in the absence of re-consolidation.

# Abstract: COGNITIVE IMPAIRMENT CORRELATES TO DECLINE IN N1 COMPONENT OF THE AUDITORY EVENT-RELATED POTENTIALS IN PATIENTS WITH TYPE 1 DIABETES

**Authors:** G. C. Cooray, L. Maurex, T. Brismar

**Affiliations:** Karolinska Institutet, Department of Clinical Neuroscience, Sweden

**Background**: A decline in mental speed and memory function has been reported in diabetes, and there is concern that this might be caused by frequent hypoglycemic events (with relation to intensive glucose control), and/or to bad glucose control.

**Aims**: To understand how cognitive defects are caused by type 1 diabetes and how they might be prevented. Methods. Patients with type 1 diabetes (*n* = 150) were studied with EEG, auditive evoked potentials in odd-ball paradigms, cognitive tests, neurography and sensory tests. Normal data of the neurophysiological parameters were obtained from 116 healthy controls (age and gender matched). Data were zscored in order to compensate for age and gender effects. Mean age of patients and controls was 43 years.

**Results**: The most significant neurophysiological abnormality was a widespread decline in the auditory evoked potential (N1) with nadirs (*p* < 10 − 4) in the temporo-parietal regions. Test for correlation between the cognitive scores and the N1 component showed the highest correlation (*p* < 10 − 4) for the psychomotor speed. Of the relevant clinical variables old age, long diabetes duration and high BMI were the strongest predictors of cognitive decline, whereas frequency of hypoglycemic events had no effect. A pronounced decline of EEG beta power was found in both temporo-occipital regions (*p* < 0.001), which was the most significant abnormality in the resting EEG power spectrum. This agrees with two previous studies from our group. However, this abnormality had only weak correlation with the outcome in the cognitive tests.

**Conclusion**: The present data indicate that age and time dependent factors are important for the cognitive decline in type 1 diabetes. The N1 component of the auditory eventrelated potential is a sensitive test for brain dysfunction in type 1 diabetes, and its applicability to other disease conditions with cognitive impairment deserves to be evaluated.

# Abstract: “OFF-LINE” PREFRONTAL REPETITIVE TRANSCRANIAL MAGNETIC STIMULATION EFFECTS ON REGIONAL CEREBRAL BLOOD FLOW CHANGES: TIMECOURSE AND SPATIAL EXTENT

**Authors:** C. Eisenegger, V. Treyer, D. Knoch, E. Fehr

**Affiliations:** ^1^Center for Social Neuroscience and Neuroeconomics, Institute for Empirical Research in Economics, University of Zurich Switzerland; ^2^PET Center, Nuclear Medicine, Department of Radiology, University Hospital Zurich, Switzerland

Low frequency “off-line” repetitive transcranial magnetic stimulation (rTMS) over the course of several minutes has gained a lot of interest as a research tool in cognitive neuroscience due to its ability to induce functional disruptions of brain areas. This disruptive TMS effect is highly valuable for revealing a causal relationship between brain and behaviour. However, the effect on remote interconnected areas and, in particular, the duration of the induced neurophysiological effect remains unknown and are critical in designing a cognitive study. In order to investigate these issues, 12 healthy subjects underwent 8H_2_O[15] PET scans after application of long-train low-frequency rTMS to the right dorsolateral prefrontal cortex. Immediately after the stimulation train, rCBF increases were present under the stimulation site as well as in other prefrontal cortical areas, including the lateral orbitofrontal cortex ipsilateral to the stimulation site. The mean increases in rCBF returned to baseline within nine minutes. These results might be of particular interest for those who aim at modulating behaviour in cognitive paradigms by means of rTMS.

# Abstract: COGNITIVE IMPAIRMENTS IN DEPRESSION: ARE THEY CLEAR-CUT AND PREDICTIVE FOR THE FURTHER COURSE?

**Authors:** S. Reppermund, M. Ising, S. Lucae, J. Zihl

**Affiliations:** Max Planck Institute of Psychiatry, Munich, Germany

Cognitive performance is often impaired in depression, and may even persist after remission of psychopathological symptoms. Moreover, some authors found that cognitive performance, in particular, executive dysfunctions, have predictive value for the further course of depression. However, very few longitudinal studies exist on possible associations between cognitive function and depression. The objective of this study was to examine cognitive performance in depressed patients in the acute phase, after remission, and six months after remission, to determine the specificity of cognitive (dys-)functions and the relevance for the further course of depression. Assessment of cognitive function and psychopathological symptoms were carried out in 62 depressed inpatients on admission and prior to discharge. Twenty-three patients were retested six months after discharge. To control for practice effects, 13 healthy subjects were assessed twice with the same cognitive tests. In the acute stage of depression, patients were impaired in memory, attention, and executive functions. After remission, cognitive performance was improved, but still impaired in a high number of patients. As the controls also improved in some tests, improvements in the patient group in these tests may rather reflect practice and not treatment effects. We found no differences in cognitive performance between remitters and non-remitters, and severity of depression was not correlated with cognitive performance, indicating that psychopathological and neuropsychological symptoms are dissociable. In addition, no cognitive function could be ascertained as a predictor for remission or relapse. Our results support the hypothesis that cognitive impairments are not clear-cut, are trait-dependent, and are therefore not merely an epiphenomenon of depression. For clinical implications, it is thus important to consider rehabilitation of cognition as complementary to pharmaceutical and other treatment measures.

# Abstract: HOW DO WE LEARN TO NAVIGATE: COMPETITION OR COEXISTENCE OF NAVIGATION STRATEGIES?

**Authors:** K. Igloi, M. Zaoui, A. Berthoz, L. Rondi-Reig

**Affiliations:** CNRS UMR 7152- LPPA Collge de France, France

Navigation in a complex environment can rely on the use of different spatial strategies. However, traditional questions on temporal arousal and hierarchy between strategies are still debated. To address this issue we investigated spontaneous choice and switches between two spatial strategies: the mapbased or allocentric and the route-based or sequential egocentric strategies during the process of navigation. Hitherto we developed the virtual version of the Starmaze, adapted from rodent studies [1, 2]. Subjects had to learn to navigate to a hidden location using an allocentric and/or a sequential egocentric strategy known to depend on different memory systems. Through a probe test simultaneously arising and equally performing strategies were characterized. Our results showed bi-directional strategy switches, suggesting no temporal or performance-related hierarchy between strategies. Thus, we argue that albeit spontaneously performed distinct navigation strategies, all sensorial information are encoded in parallel during learning, leading to mentally coexisting strategies that are used, separately or conjunctly, according to their accuracy to solve the task. Spontaneous decision of the strategy used does not seem to depend on the practice of the task but rather on the degree of awareness of the environment.

## References

[1] Burguière E, Arleo A, Hojjati MR (2005). Spatial navigation impairment in mice lacking cerebellar LTD: a motor adaptation deficit?. *Nature Neuroscience*.

[2] Rondi-Reig L, Petit GH, Tobin C, Tonegawa S, Mariani J, Berthoz A (2006). Impaired sequential egocentric and allocentric memories in forebrain-specific-NMDA receptor
knock-out mice during a new task dissociating strategies of navigation. *Journal of Neuroscience*.

# Abstract: DO THE MATH: ROOTS OF NUMERICAL COMPETENCE IN FISH

**Authors:** C. Agrillo, M. Dadda, G. Serena, A. Bisazza

**Affiliations:** Department of General Psychology, Via Venezia 8, 35131 Padova, Italy

Comparative studies have demonstrated that human infants, apes, monkeys, dolphins, dogs and birds have a rudimentary numerical system that enables them to discriminate between two groups on the basis of the numerosity of the items, allowing survival and successful reproduction in many situations. At present few attention has focus on the numerical abilities in lower vertebrates. We investigated the ability to discriminate among different numerosities in a poeciliid fish, Gambusia holbrooki, by using the spontaneous social choice for shoals differing in numerosity in two different ecological contests: when placed in a potentially dangerous environment and when facing a harassing male. In both context, we found that females were able to discriminate between two shoals that differed by one element when the paired numbers were 1 vs 2, 2 vs 3 and 3 vs 4, but not 4 vs 5 or larger. Using large numerosities (>4), in agreement with the numerical distance effect, the ability to discriminate between two numbers improved as the distance between them increased, and a significant discrimination was found with ratios of 1 : 2 or smaller (4 vs 8, 8 vs 16 and 4 vs 10). Control experiments of non-numerical variables evidenced the role played by the total area occupied by the stimuli in the selection of the larger group. The importance of total movements of the stimuli is less clear, since it may affect quantity discrimination only in comparisons between large numerosities. In summary our study indicates that spontaneous numerical abilities in fish are comparable with those of other non-verbal creatures studied; results are in agreement with the hypothesis of the existence of two distinct systems for quantity discrimination in Vertebrates.

# Abstract: TIME COURSE OF SCENE PROCESSING IN HUMANS

**Authors:** C. Thinus-Blanc, E. Barbeau, C. Liegeois-Chauvel, P. Marquis, P. Chauvel, J. Regis, P. Peruch

**Affiliations:** ^1^INSERM U-751 Epilepsie & Cognition, Faculté de Médecine de la Timone, Marseille, France; ^2^CNRS UMR 5549 Cerveau & Cognition, Faculté de Médecine de Rangueil, Toulouse, France

Whereas many fMRI studies have investigated the brain structures involved in visual scene encoding and recognition (for example, Epstein & Kanwisher, 1998; Maguire, Frith, & Cipolotti, 2001), the time course of activation of these structures is relatively unexplored. A fine-grained spatiotemporal analysis would provide information about the dynamics of the brain network involved in such complex processing. In the present study, intra-cerebral activity was recorded in epileptic patients while they performed a series of tests of encoding/recognition of visual scenes and of other types of visual stimuli (objects, faces, abstract structures and configurations,. . .). Thus, it was possible to combine both high temporal and spatial resolution of the signal. Preliminary results suggest that information related to “natural” scenes flows from the primary visual cortex (with a latency of about 110 ms) to the posterior parahipoccampal area (PPA), and later to the posterior and anterior hippocampus. No marked difference of activity was found between encoding and recognition. Abstract structures did not involve as strongly the parahippocampal cortex. Finally, visual object processing activated a network where the rhinal cortices were predominantly involved. Further analyses are currently conducted in order to determine to what extent this apparently serial processing would take place in parallel. The present data confirm the function of the PPA in scene encoding and recognition as evidenced by fMRI studies, and bring additional information as to the time course of scene processing and to its functional significance.

# Abstract: Theme D: Disorders of the nervous system  MEMORY IMPAIRMENTS IN HEPATIC ENCEPHALOPATHY

**Authors:** M. Méndez, M. Méndez-López, L. López, M. A. Aller, J. Arias, J. L. Arias

**Affiliations:** ^1^Laboratory of Psychobiology. School of Psychology. University of Oviedo, Oviedo, Spain; ^2^School of Medicine, Complutense University of Madrid, Madrid, Spain

Hepatic encephalopathy is a CNS disorder associated with hepatic dysfunction, characterized by certain changes in personality and cognitive deficit such a spatial disorientation and memory impairment. Our objective is to evaluate spatial reference and working memory in different rat models of hepatic dysfunction. Spatial memory, assessed with Morris water maze, is studied in 90 Wistar rats that are divided in five groups: rats with portocaval anastomosis (PCA), rats with triple partial portal vein ligation (TPVL), rats that received thioacetamide in the drinking water (0.03%) (TAA), rats pseudo-operated (PSO) and control rats (CTRL). These five groups are then distributed for the different experiments: spatial reference memory, PCA (*n* = 8), TPVL (*n* = 10), TAA (*n* = 10), PSO (*n* = 8) and CTRL (*n* = 10), and spatial working memory, PCA (*n* = 8), TPVL (*n* = 8), TAA (*n* = 10), PSO (*n* = 9) and CTRL (*n* = 9). There are not differences between groups in anxiety and activity tasks.The spatial reference memory task shows no differences between groups in escape latencies (F4/41 = 1.469, *p* = 0.229). No-platform probe tests show that CTRL and PSO groups have learned the location of platform since the firts and second days. TAA group is unable to learn the task. PCA rats displays learning only on day 5. TPVL group acquires the task on day 3 but fails on 5. For the spatial working memory task, the groups TPVL and TAA are incapable to acquire the task with no differences between sample and retention trials (*p* = 0.342 and *p* = 0.382). Cirrhosis induced by TAA produces spatial reference and working memory impairment, TPVL produces impairment in working memory and difficulties with reference memory. PCA is able to acquire working memory task but shows delay with reference. Therefore, models of hepatic dysfunction show different memory impairment and TAA model seems to be the most serious one.

# Abstract: AN INVESTIGATION OF MOTIVATIONAL PROCESSES IN AN ANIMAL MODEL OF ALZHEIMER'S DISEASE

**Authors:** M. J. Lelos, M. A. Good

**Affiliations:** School of Psychology, Cardiff University, Cardiff, UK

The production of *β*-amyloid is thought to be one of the cardinal pathological events in the development of cognitive deficits in Alzheimer's disease. Amyloid has been shown to accumulate in a variety of brain regions implicated in memory, including the hippocampal formation. Consistent with the view that *β*-amyloid production impairs neuronal mechanisms underlying memory, mice expressing human amyloid precursor protein mutations linked to a familial form of AD (e.g., Tg2576 mice) show deficits in several hippocampus-dependent spatial memory tasks. However, there is less information concerning the effects of this mutation on cognitive processes supported by other neural systems that also display amyloid pathology. In the present study we examined the effects of the APPswe mutation on appetitive learning processes associated with basolateral amygdala, a region that shows age-dependent deposition of *β*-amyloid. More specifically, we examined the effects of the APPswe mutation on appetitive incentive learning processes using both instrumental and Pavlovian outcome-specific devaluation tasks. Our results indicated that the APPswe mutation did not affect the animals' ability to form a representation of an action-outcome association or to change their instrumental behaviour following incentive learning. We did, however, find a deficit in the animals' ability to use context cues to access information about the current incentive value of an outcome. These findings have implications for our understanding of the effects of the mutation on the neural systems supporting learning and memory.

# Abstract: SPATIAL MEMORY IMPAIRMENTS ASSOCIATED WITH LPA1 RECEPTOR NULL MUTATION

**Authors:** D. López-Barroso, L. Santín, C. Pedraza, E. Castilla-Ortega, J. Sánchez-López, E. Matas-Ricom, J. Chun, F. Rodriguez De Fonseca, G. Estivill-Torrús

**Affiliations:** ^1^Departamento de Psicobiología, Universidad de Malaga, Spain; ^2^Unidad de Investigación, Fundación IMABIS, Spain; ^3^Department of Molecular Biology, The Scripps Research Institute, CA, USA

The lysophosphatidic acid (LPA) is a bioactive phospholipid acting as an intercelullar messenger through specific protein G-coupled receptors (LPA1–4). LPA1 receptor is expressed in the adult brain in the hippocampus. Few experiments have shown a LPA1 receptor involvement in the normal function of the adult hippocampus but no direct evidence has been shown about the LPA1 receptor in learning and memory. In this study, we used a variant of formerly characterized LPA1- null mutant, that spontaneously arose during colony expansion (maLPA1-null). Spatial learning and memory was studied in the maLPA1-null mice using the Morris Water Maze (MWM). In addition, it was assessed the exploratory and locomotor behaviors in the open field and several neurologic functions. maLPA1-null mice showed an impairment in several neurologic tests: somesthesis, grasping reflex, equilibrium and olfaction tests. Also, a general locomotor impairment was demonstrated in the open field and MWM tasks, revealed by the reduced velocity in both tasks. Thus, maLPA1-null mice exhibited an exploratory impairment in the open field, with a reduced general exploration of the open field. In the MWM, it was observed that the maLPA1-null mice were able to learn a spatial memory task with reference memory demands. However, the transfer task analysis showed that the maLPA1-null mice spent less time in the target quadrant in the MWM, suggesting an alteration in the extinction. Complementary, maLPA1-null mice mainly used non-spatial searching strategies in this task than the wild type mice, that used mainly spatial strategies. The results suggest the LPA1 receptor involvement in several cortical and hippocampal functions.

# Abstract: EXOGENOUS NGF PREVENTS AGE-ASSOCIATED CHOLINERGIC NEURONS ATROPHY BUT HAS LIMITED EFFECT ON COGNITIVE DEFICIT IN RATS

**Authors:** G. Niewiadomska, M. Mazurkiewicz, A. Mietelska, E. Nosecka, M. Baksalerska-Pazera

**Affiliations:** Department of Neurophysiology, Nencki Institute of Experimental Biology, Warsaw, Poland

The decrease of trophic support for the cholinergic neurons in aging brain may lead to neuronal atrophy and appearance of neurodegenerative diseases such as Alzheimer's disease. In rats, object discrimination depends on the integrity of the cholinergic system, thus it could be expected that nerve growth factor (NGF) can improve the behavior in aged subjects. The interactive effect of age and cholinergic improvement was assessed behaviorally in young and aged rats injected intraventriculary with NGF. In order to check an effect of NGF infusion on different type of memory we examined rats performance in three different behavioral tests: short-lasting training in object-recognition task (recognition memory) in object-location task (spatial memory), and long-lasting training in acquisition and reversal of brightness discrimination test (long-term procedural memory). In aged rats, NGF positively effected cognitive processes related to spatial memory, but was ineffective on memory processes involved in the formation of associations established in recognition memory tasks. In acquisition of discrimination learning the choice accuracy was the same for young and aged, both control and NGF-treated rats. However, in reversal learning NGF-treated aged rats mastered the test much longer and made significantly more errors than all other groups. These indicate that in aged animals exogenous NGF deteriorates storage and recall of memory. Together, the contrasting findings show that NGF administration modulates spatial, recognition and visual memory in different not always beneficial ways.

# Abstract: PLACE-FIELD DYNAMICS OF HIPPOCAMPAL CA1 NEURONS DURING TRAVERSAL OF SPIRAL PATHS

**Authors:** D. A. Nitz

**Affiliations:** The Neurosciences Institute, San Diego, CA, USA

For a single position in allocentric space, behavioral context can strongly influence the place-specific discharge of CA1 hippocampal neurons. Here, it was determined whether the same behavioral context could drive similar activity patterns in CA1 hippocampal neurons despite differences in allocentric position. To this end, three rats were trained to traverse, from outside to inside, leftward-moving and rightward-moving spiral paths. Each spiral path was defined by 5 cm. walls permitting visualization of distal cues and was comprised of 5 full “loops” leading to a food reward site in the center. Under these conditions, three features of the spatial firing patterns of CA1 principal neurons were remarkable. First, CA1 neurons exhibited analogous spatial firing patterns across individual loops of the full spiral track. Specifically, many neurons (66%) exhibited multiple, robust firing fields approximately localized to the same positions of 3 or more individual loops. Second, for such neurons, firing fields adapted to the size of individual loops. Spatial firing fields on outer, longer loops were proportionally larger in length than those for inner, shorter loops. Finally, relative to a radial line drawn through each loop of a given spiral, the centers of mass of firing fields progressively shifted forward. The former results indicate that hippocampal activity can generalize across similar experiences irrespective of differences in their duration and relation to allocentric space. The latter result suggests that distance and directional components of path integration exert dissociable influences on CA1 neurons.

# Abstract: NMDA RECEPTOR SUBUNIT 1 HYPOMORPHIC MICE SHOW IMPAIRED HIPPOCAMPAL LTP, STEREOTYPICAL BEHAVIOUR AND DEFICITS IN SPATIAL LEARNING TASKS

**Authors:** S. Bickel, A. Sieber, D. Vyssotski, D. Umbricht, D. Balschun, H. P. Lipp, D. P. Wolfer

**Affiliations:** ^1^Institute of Anatomy, University of Zurich, Switherland; ^2^Translational Medicine, Exploratory Clinical Development, Novartis Pharma AG, Basel, Switzerland; ^3^Department of Psychology, Katholieke Universiteit Leuven, Leuven, Belgium

The constitutive mouse mutant with 90% reduced expression of the NMDA receptor subunit 1 (NR1) has been widely investigated as a putative mouse model of impaired glutamatergic signaling in schizophrenia. Phenotyping of these mutants mainly focused on specific behavioral and neurophysiological tests that are thought to be of relevance as putative endophenotypes of this disorder. To date, data is missing regarding the phenotype of the NR1 mutants in hippocampal long-term potentiation (LTP) and many traditional behavioral tests. The NR1 mutants showed reduced LTP due to highfrequency tetanic (*p* < 0.001), as well as theta-burst stimulation (*p* < 0.001) in the CA1 region compared to controls. The mutants (*N* = 23, controls *N* = 23) showed increased open sector activity on the O-maze, increased indexes of stereotypy and impaired habituation in the emergence and open field test, in the novel object test object exploration was disinhibited. Escape time in the water-maze was massively prolonged in the mutants (*p* < 0.001). Indeed, their performance was the second worse ever tested in a database covering 107 different mutations. Indexes of working memory in the 8-arm radial maze were significantly impaired in themutant group (*N* = 12, controls *N* = 14). However, in both memory tests NR1 mutants showed increased measures of stereotypical behavior and altered movement patterns, representing a major confounding factor for analyzing specific memory impairments. The present data imply impairments in the NR1 mutants in multiple brain systems. However, no conclusive statement can be made regarding memory deficits in spatial learning tasks, due to stereotyped movement patterns in these mutants.

# Abstract: VOLUNTARY WHEEL-RUNNING ATTENUATES THE MILD COGNITIVE DEFICITS, BUT NOT THE ENHANCED ACOUSTIC STARTLE RESPONSE IN A TRIPLE TRANSGENIC MOUSE MODEL OF ALZHEIMER'S DISEASE

**Authors:** S. Pietropaolo, F. M. Laferla, I. Knuesel, J. Feldon, B. K. Yee

**Affiliations:** ^1^Laboratory of Behavioral Neurobiology, ETH, Zurich, Switzerland; ^2^Department of Neurobiology and Behavior, University of California, Irvine; CA, USA; ^3^Institute of Pharmacology and Toxicology, University of Zurich, Switzerland

Converging lines of evidence from human and animal studies suggest that voluntary physical activity exerts cognitiveenhancing and neuroprotective effects. In the present study, we evaluated the efficacy of voluntary running exercise in ameliorating the behavioral phenotypes of a triple transgenic mouse model of Alzheimer disease (3×Tg-AD). This genetic model of human AD harbors two transgenes (encoding APP_Swe_, and tau_P301L_) in a homozygous PS1_M146V_ knockin background [1]. These 3×Tg-AD mice progressively develop A*β* and tau pathology, with a temporal-and regionspecific profile that closely mimics the neuropathology of human AD. Here, male and female mutant and wild-type (129/C57BL6 hybrids) mice were housed at adulthood (3 months of age) with either a running or locked wheel and left in the corresponding housing conditions for the entire experimental period. At 7 months of age, that is at the early stages of AD pathology, mice were tested for anxiety, attentional gating and spatial memory. The 3×Tg-AD mice showed normal anxiety levels, but enhanced acoustic startle reactivity in comparison to controls. Abnormal startle response was observed in mice of both sexes and it was not attenuated by running activity. The 3×Tg-AD mice showed a mild deficit in spatial learning as evaluated on the water maze reference memory test and in spontaneous alternation in the Y-maze. Thesemild cognitive impairments were more pronounced in female animals and were largely ameliorated by the availability of wheel-running. Our data demonstrated the contribution of epigenetic and genetic factors in the ethiopathology of AD, underlining the efficacy of voluntary physical exercise in selectively attenuating some, but not all AD-like symptoms.

## References

[1] Oddo S, Caccamo A, Shepherd JD (2003). Triple-transgenic model of Alzheimer's disease with plaques and tangles: intracellular A*β* and synaptic dysfunction. *Neuron*.

# Abstract: NEUROBEHAVIORAL ASSESSMENT IN PRE-CLINICAL SPECIES: RODENTS, DOGS, MINIPIGS AND MONKEYS

**Authors:** E. Moscardo, A. Giarola, N. Fasdelli, M. Tontodonati, R. Dorigatti

**Affiliations:** Safety Pharmacology Group, Dept. Safety Assessment, GlaxoSmithKline R&D Centre, Verona, Italy

Most of the common unwanted side-effects on the nervous system in humans are not easily identifiable in pre-clinical species. This area of safety is therefore of particular importance in the assessment of new potential pharmaceutical compounds and it has been included as part of the “core battery” assessment of vital organ functions in the ICH S7A safety pharmacology guideline. A behavioral test was first introduced by Irwin as a rapid psychotropic screening procedure adapted for use in mice. Following modifications Functional Observational Batteries (FOBs) were developed for rats and more recently adapted for use in the most common non-rodent species. It is of particular importance when assessing neurobehaviour to carefully control and to standardise all factors involved in the test (the animal, the observer, the observation battery and environmental conditions) to make the data collected objective, reproducible, reliable and predictive of safety liabilities. In our research's experience, detailed neurobehavioural observation batteries were developed for use in pre-clinical species: rodents, dogs, mini-pigs and nonhuman primates. The rodent FOB assesses home cage and open field activities, stimulus reactivity and neuromuscular functions. The dog and mini-pig FOBs have been developed from rodent one and are, the first focused on the interaction with humans and emphasises evaluation of gait, postural reactions and reflex functions, while in the second battery, due to the remissive behaviour ofmini-pigs, the evaluations with handling are limited to a minimum. The monkey FOB is considered to be the most suitable assessment when effects on higherlevel cognitive and behavioural processes are expected with a direct relevance for humans. In conclusion, it is possible to perform a reliable neurobehavioual assessment in all the major pre-clinical species, using a standard observation battery, but the procedure has to be carefully adapted and optimised to the species taking into account social variables, ethical issues and costs-benefits ratio.

# Abstract: MOTOR ACTIVITY IS MODULATED VIA DIFFERENT NEURONAL CIRCUITS IN RATS WITH CHRONIC LIVER FAILURE THAN IN NORMAL RATS

**Authors:** O. Cauli, N. ElMlili, M. Llansola, V. Felipo

**Affiliations:** Laboratory of Neurobiology, Centro de Investigacion Príncipe Felipe, Valencia, Spain

The mechanisms by which liver failure alters motor function remain unclear. It has been suggested that liver disease alters the neuronal circuits between basal ganglia and cortex that modulates motor function. Activation of group I metabotropic glutamate receptors in nucleus accumbens by injecting DHPG activates one circuit that induces locomotion. We analyzed by in vivo brain microdialysis the function of the circuits that modulate motor function in rats with liver failure due to portacaval shunt (PCS). We inserted cannulae in nucleus accumbens and microdialysis probes in nucleus accumbens, ventral pallidum, substantia nigra pars reticulata, medio-dorsal thalamus, ventro-medial thalamus or prefrontal cortex. We injected DHPG in nucleus accumbens and analyzed extracellular neurotransmitters concentration in these areas. The results indicate that in control rats DHPG induces locomotion by activating the “normal” neuronal circuit: nucleus accumbens < ventral pallidum < medio-dorsal thalamus < prefrontal cortex. In PCS rats this circuit is not activated. In PCS rats DHPG injection activates an “alternative” circuit: nucleus accumbens < substantia nigra pars reticulata < ventromedial thalamus < prefrontal cortex. This circuit is not activated in control rats. DHPG injection increases dopamine in NAcc of control but not of PCS rats and glutamate in PCS but not in control rats. DHPG-induced increase in dopamine would activate the “normal” neuronal circuit while increase in glutamate would activate the “alternative” circuit. The identification of the mechanisms responsible for altered motor function and coordination in liver disease would allow designing treatments to improve motor function in patients with hepatic encephalopathy.

# Abstract: INCREASED SENSITIVITY TO PENTYLENETETRAZOLEINDUCED BUT NOT AUDIOGENIC SEIZURES IN AN ANIMAL MODEL OF AUTISM CREATED BY PRENATAL EXPOSURE TO VALPROIC ACID

**Authors:** T. Schneider, R. Przewlocki

**Affiliations:** Department of Molecular Neuropharmacology, Institute of Pharmacology, Krakow, Poland

Many studies have shown that there is an increased frequency of seizures in autism, and conversely, autism and milder pervasive developmental disorders are more common in epileptic children than in the general population. Recently a new rodent model of autism induced by prenatal exposure to valproic acid on 12.5 day of gestation has been created (VPA rats). The model has striking etiological, anatomical, and behavioral similarities to human data. VPA rats' susceptibility to seizures has not been studied yet. The purpose of the present experiments was to evaluate male Wistar VPA rats' susceptibility to seizures induced by pentylenetetrazole (PTZ) or audiogenic stimulation. To elicit audiogenic seizures, rats were placed in a Plexiglas cylindrical chamber within soundproof cabinet and exposed to 110 dB sound lasting up to 90 sec, once daily for 14 days. Rats were scored for their reactions according to the widely used Jobe's scale. Another group of rats was observed for 30 min after PTZ (65 mg/kg) intraperitoneal injection. The reaction phases included: unresponsiveness, mild contractions, clonic seizures (with and without loss of righting reflex), and tonic seizures. Both latency to and number of animals achieving any particular phase were scored. There was no difference between VPA and control animals in severity and time parameters of audiogenic seizures. There was also no difference in a first-trial (i.e., non-priming induced) audiogenic seizure. However, VPA rats showed significantly shorter latency to the mild contractions and clonic PTZ-induced seizures, as well as a higher number of animals expressing clonic seizures.Our data suggest that VPA rats might bemore susceptible to PTZ-induced seizures. This observation corresponds to the increased frequency of epilepsy in autism and suggests that prenatal exposure to substances enhancing GABA-ergic tone (e.g., VPA) might increase sensitivity to epileptogenic substances in subsequent developmental stages.

# Abstract: INFLUENCE OF SOME PHARMACOLOGICAL DRUGS ON THE HIPPOCAMPAL EPILEPTIFORM DISCHARGES

**Authors:** E. Saralidze, L. Khuchua, T. Ioseliani

**Affiliations:** ^1^Department of Biology, Shota Rustaveli state University, Batumi, Adjara, Georgia; ^2^Department of Biology, Tbilisi state University, Tbilisi, Georgia

Alterations of the hippocampal epileptogenic thresholds following piracetam and diazepam administration were investigated in the albino rat. The rats were stereotaxically implanted with hippocampal electrodes in order to record epileptic activity. EEG recording was made in the hippocampus as well as in the neocortex. Epileptiform discharges (ED) were induced by electrical stimulation of dorsal hippocampus. To this end study was made of: the effect of piracetam intraperitoneal injection on hippocampal epileptiform discharges and the effect of diazepam intraperitoneal injection on hippocampal epileptiform discharges. Analysis of the data obtained may lend support to the following conclusions: Experiments have shown that intraperitoneal administration of piracetam (20–25 mg/kg) decreased epileptogenic threshold in the hippocampus, whereas diazepam injection (0.5–1 mg/kg) increased epileptogenic threshold. These results might be because piracetam activates cortical neurons, which in turn decrease their tonic inhibitory influence on the hippocampus. Contrariwise, diazepam elevated influences of the collateral inhibition within the hippocampus.

# Abstract: TOWARDS A MODEL OF NEUROLEPTIC-RESISTANT COGNITIVE IMPAIRMENTS IN SCHIZOPHRENIA: THE ANTIMUSCARINIC LATENT INHIBITION MODEL

**Authors:** S. Barak, I. Weiner

**Affiliations:** Department of Psychology, Tel-Aviv University, Israel

Schizophrenia symptoms segregate into positive, negative and cognitive, which exhibit different sensitivity to drugs. Muscarinic antagonists (e.g., scopolamine) produce an “antimuscarinic syndrome”, characterized by psychosis and cognitive impairments. We suggest modeling both symptom classes as contrasting effects of low and high doses of scopolamine on latent inhibition (LI). LI indexes the capacity to ignore irrelevant stimuli as reflected in poorer conditioning to a stimulus that had been repeatedly preexposed without consequences. Amphetamine-induced disrupted LI and its reversal by typical and atypical neuroleptics is a well established model of positive symptoms. Conversely, abnormally persistent LI produced by NMDA receptor antagonists and its reversal by glycinergic drugs and atypical but not typical neuroleptics has been proposed to model negative/cognitive symptoms. Recently, we reported that low but not high doses of scopolamine led to LI disruption, which was reversed by typical and atypical neuroleptics and the acetylcholinesterase inhibitor physostigmine. Here, we tested the capacity of a higher dose of scopolamine to produce persistent LI, and the sensitivity of the latter to neuroleptics and cognitive enhancers. We found that a high dose of scopolamine produced abnormal persistent LI, which was resistant to both typical and atypical neuroleptics (haloperidol and clozapine), but was reversed by cholinergic and glycinergic cognitive enhancers (physostigmine and glycine). This contrasts with LI disruption induced by low scopolamine doses, which is reversed by both neuroleptics and physostigmine, and which we have proposed to model the positive spectrum of antimuscarinic psychosis. As cognitive impairments in schizophrenia are not improved, or even exacerbated, by neuroleptics, but can be alleviated by cognitive enhancers, we suggest that scopolamine-induced abnormally persistent LI can model neuroleptic-resistant cognitive impairments in schizophrenia.

# Abstract: NEURO DEVELOPMENT AND BEHAVIORAL CHARACTERIZATION OF EHMT1 MOUSE PUPS WITH SYNDROMIC MENTAL RETARDATION?

**Authors:** V. D. Z. CEEM, K. Arthur, V. B. JHLM

**Affiliations:** ^1^Department of Cell Biology, Nijmegen Centre for Molecular Life Science, Nijmegen, The Netherlands; ^2^Department of Human Genetics, Radboud University, Nijmegen Medical Centre, Nijmegen, The Netherlands

With an 8% health-care expenditure for mental handicaps and a high burden for society and families, Mental Retardation (MR) is one of most important unsolved problems in medicine. Elucidation of the genetic causes is important, but MR is extremely heterogeneous, both clinically and genetically.

The characterization of chromosomal microdeletions has allowed us and others to define a common clinically recognizable syndrome, consisting of severe MR, hypotonia, microcephaly, typical facial dysmorphism and heart defects in patients. We have recently established that this MR syndrome is caused by haplo-insufficiency of the Euchromatin Histone Methyl Transferase 1 (*EHMT1*) gene. In addition, we have demonstrated by mouse *in situ* hybridization studies that this gene is highly expressed during embryonic brain development. Selective *EHMT1* expression was subsequently observed in areas of the adult brain that contain actively dividing neurons. These data indicate that *EHMT1* may act as a cell cycle regulator in neuronal progenitors cell populations and most likely will affect brain development. In this study the comparison of morphological and neurological development in newborn wildtype and Ehmt1+/− mice will add valuable information to studies that only analyzed behavior in adult animals (like the Fragile X mental retardation syndrome mouse model). Neurological development and possible behavioral abnormalities in the newborn and postnatal mouse pups will be measured using the following behavioral tests: Surface righting (labyrinth and postural response), walking (coordination andmuscular strength), cliff drop aversion test (somato sensory response), geotaxis test (labyrinth and postural reaction), grasping test (freeing reflex) and bar holding test (muscular strength). Morphological development, such as body weight, the age of onset of incisor eruption, nipple development, eye-opening, earelevation and opening, and hair growth, will be determined by daily observation of the individual pups.

# Abstract: DEEP BRAIN STIMULATION IN THE NUCLEUS ACCUMBENS FOR OBSESSIVE COMPULSIVE DISORDER

**Authors:** R. Muffatti, R. Cordella, O. Gambini, G. Messina, S. Scarone, A. Franzini, M. Savino, G. Broggi

**Affiliations:** ^1^Department of Psychiatry, University of Milan Medical School and San Paolo Hospital, Milan, Italy; ^2^Department of Neurosurgery, Fondazione Istituto Besta, Milan, Italy; ^3^Department of Psychiatry, Neurobiology, Pharmacology and Biotechnology, University of Pisa, Pisa, Italy

Nucleus accumbens (NA) has been recognized to play an important role in reward, pleasure and addiction. Several neuroimaging studies of patients affected by Obsessive Compulsive Disorder (OCD) have pointed to basal ganglia and orbitofrontal cortex being relevant for pathophysiology of this disorder. As a central relay structure between amygdala, basal ganglia, mesolimbic dopaminergic areas, mediodorsal thalamus and prefrontal cortex, NA has been proposed by Sturm et al. (2003) as a target for deep brain stimulation (DBS) in OCD. Here we report two cases of OCD patients treated by DBS of NA. Technical details of electrode implantation and neurophysiological parameters of stimulation, as well as clinical description of both patients at baseline and follow up are given. Finally a neurobiological model of OCD is proposed.

# Abstract: EARLY POSTNATAL HANDLING IMPROVES COGNITIVE DEFICITS AND EMOTIONAL-BEHAVIOUR ALTERATIONS IN 3XTGAD MICE FOR ALZHEIMER DISEASE

**Authors:** T. Cañete, G. Blázquez, F. LaFerla, A. Tobeña, A. Fernández-Teruel, L. Gimenez-Llort

**Affiliations:** ^1^Institute of Neuroscience, University of Alabama at Birmingham, Birmingham, Albama; ^2^Department of Neurobiology and behaviour, University of California, CA, USA

The 3xTg-AD mice harboring PS1M146V, APPSwe, tauP30- 1L transgenes and mimicking many critical hallmarks of Alzheimer's disease, show cognitive deficits but also other neuropsychiatric-like behavioural alterations (i.e., anxiety, reduced exploratory behaviour) from early stages when overt BA and tau neuropathologies are not yet observed, but when intraneuronal accumulation of Abeta, synaptic and cholinergic deficits have already been described. More recently, emerging evidence indicates that intraneuronal Abeta may also contribute to the cascade of neurodegenerative events and strongly suggest that it is an early, pathological biomarker for the onset of Alzheimer's disease and associated cognitive and other behavioral deficits. On the other hand, postnatal handling (PH, tactile stimulation administered from postnatal days 1 to 21) is an early-life treatment known to produce profound and long-lasting behavioural and neurobiological effects. Emotionality, reactivity to stressors and exploratory behaviour as well as the functionality of several neurotransmitter systems can be enduringly altered by this procedure. The present study was aimed at describing the effects of early postnatal handling on the behavioural profile of male and female 3xTgAD mice at early stages of the disease (4 month-old). The results show that early postnatal handling treatment increased the activity levels in the open-field, reduced anxiety in this and other tests (elevated plus-maze and dark-light box) and improved the acquisition of place learning in the Morris water maze. Moreover, impulsive/disinhibitory behaviours observed in the 3xTgAD groups were also reduced by this treatment. Therefore, the results suggest that early-life treatments such as postnatal handling may exert a preventive effect on both cognitive deficits and emotional alterations characteristic of Alzheimer's disease.

# Abstract: THERAPEUTICAL EFFECTS OF ENVIRONMENTAL ENRICHMENT ON COGNITIVE AND NEUROPSYCHIATRIC-LIKE SYMPTOMS AT ADVANCED STAGES OF ALZHEIMER'S DISEASE IN 3XTGAD MICE

**Authors:** G. Blázquez, T. Cañete, B. Johansson, A. Tobeña, F. M. LaFerla, A. Fernández-Teruel, L. Gimeénez-Llort

**Affiliations:** ^1^Department of Psychiatry & Forensic Medicine, Institute of Neuroscience, Autonomous University of Barcelona, Bellaterra, Spain; ^2^Department of Neuroscience & Clinical Neuroscience, Karolinska Institutet, Sweden; ^3^Department of Neurobiology & Behaviour, University of California, Irvine, USA

Alzheimer's disease is a neurodegenerative disorder characterized not only by the progressive loss of cognitive functions but also the appearance of neuropsychiatric symptoms such as anxiety, emotional disturbances, hallucinations, aggressiveness, etc. Triple transgenic mouse model of Alzheimer's disease (3xTgAD) harbouring human APP_Swe_, Tau_P301L_ and PS1_M146V_ develops A*β* plaques and tau-tangle pathologies in an age-dependent and region-specific manner, mimicking the progression of human disease. In this model, cognitive deficits and neuropsychiatric. like symptoms are observed from early stages of the disease (6 month of age) when only intraneuronal A*β* is observed and they increase with age and development of extracellular A*β* plaques and neurofibrilary tangles. In the present work we study the putative therapeutic effects of environmental enrichment (groups of 8 mice, diverse objects of different shapes and colours changed every two days, permanent mouse house and wheel) on cognitive performance and behavioural alterations of male and female 3xTgADmice as compared to animals under standard housing conditions (2–3 animals per cage). The treatment began on the adulthood, at early stages of the disease (6 months of age) and lasted for 5.5 months. Thereafter, its effects were evaluated in a longitudinal study during the advanced stages of the disease. The battery of behavioural tests consisted of corner test, sensorymotor tasks, open field, dark-light box and hole-board tests as well as several Morris water maze paradigms. Our results show that environmental enrichment induced an improvement of cognitive function and attenuates neuropsychiatric-like symptoms, mainly observed as a reduction of anxiety. In accordance with 86/609/EEC regarding the care and use of animals for experimental procedures.

# Abstract: SYNAPTIC AND MORPHOLOGICAL CHANGES IN STRIATAL NEURON SUBTYPES UNDERLYING ENHANCED PROCEDURAL LEARNING IN BSN KO MICE

**Authors:** C. A. Sgobio, V. Ghiglieri, B. Picconi, V. Bagetta, V. Paillé, M. Ammassari-Teule, P. Calabresi

**Affiliations:** ^1^Fondazione Santa Lucia, Roma, Italy; ^2^Università di Perugia, Perugia, Italy; ^3^CNR Institute for Neuroscience, Roma, Italy

Bassoon (Bsn) is a presynaptic protein essential for docking and fusion of glutamatergic synaptic vesicles. However, the functional consequences of Bsn loss at striatal synapses remains unclear. Bsn KO mice (BSN) mice lacking the central region of the presynaptic active zone protein Bsn show intense epileptic seizures. In experimental models of epilepsy, striatal medium spiny (MS) neurons appear more susceptible to the paroxysmal cortical activity compared to fast-spike (FS) interneurons. Given the modulatory function of FS interneurons on striatal activity, it could be that the continuous spreading of epileptic seizures throughout the cortex in BSN mice might selectively alter the short- and long-termexcitability of striatal neuron subtypes To investigate this possibility, we performed in vitro intracellular recordings to compare the membrane and synaptic properties of MS and FS neurons. Successively, we examined the morphology of MS and FS neurons using Golgi and Parvalbumin staining. Then, we characterized the behavioral phenotype of BSN mice in a striatal-dependent learning task. We first observed that the intrinsic membrane properties of the two neuronal subtypes were similar in BSN and WT mice. Long-term depression (LTD) and long-term potentiation (LTP) in MS neurons also developed normally in both genotypes although the amplitude of LTP was smaller in the mutants. Surprisingly, BNS mice showed a potentiation of FS neurons absent in WT mice, more parvalbumin-positive FS neurons, and increased dendrite branching on MS neurons. Consistent with this apparent enhancement in striatal function, BSN mice exhibited higher active avoidance scores. Our results suggest that striatal neuron subtypes are differently sensitive to continuous seizures occurring in BSN mutants. In particular, the emergence of NMDAdependent plasticity in FS could represent an adaptive mechanism underlying enhanced performance in the striatal-dependent active avoidance task.

# Abstract: THE INFLUENCE OF SEROTONINERGIC DRUGS ON THYROID STATUS AND TURNOVER OF BIOGENIC AMINES IN YOUNG AND OLD RATS WITH IMBALANCE OF THYROID HORMONES

**Authors:** O. O. Masalova

**Affiliations:** Department of Neuropharmacology, Institute for Experimental Medicine of the RAMS, St. Petersburg, Russia

Triiodothyronine (T3) augments and accelerates the effects of tricyclic antidepressants. The influence of T3 on serotonin (5-HT) turnover and 5-HT_1_, 5-HT_2_ receptor activity is supposed to underlie such an action of thyroid hormones.

The aim of the present work was to study the effects of chronic administration of serotoninergic drugs on thyroid status and concentrations of biogenic amines in brain structures of old and young thyroidectomized (Tx) rats.

All experiments were carried out on young (aged 3 months) and old (aged 22–24 months) male rats. The following drugs were used: citalopram (10 days, 4 mg/kg i.p.), DL-p-chlorophenylalanine (7 days, 300 mg/kg i.p), T3 (14 days, 70 g/kg/day i.p). Serum concentrations of T3, T4, TSH were determined using enzyme immunoassay; levels of norepinephrine, dopamine, 5-HT and its metabolites were studied in hippocampus and amygdala using HPLC. Statistical processing of data was carried out by one-way ANOVA at *p* < 0.05. Citalopram decreased serum T4 concentration in young and old rats. Citalopram reduced serum TSH level in young and old rats and old Tx rats. p-Chlorophenylalanine increased blood levels of T4 and TSH in young and old rats and increased TSH concentration in Tx rats treated with T3. Citalopram decreased 5-HT concentration in hippocampus of old Tx rats. 5-HT concentration increased in hippocampus of old Tx rats treated with T3 and citalopram as compared to Tx rats receiving only citalopram. p-Chlorophenylalanine decreased the concentration and turnover of 5- HT in hippocampus and amygdala of intact rats, Tx rats and Tx rats, treated with T3. p-Chlorophenylalanine decreased dopamine concentration in hippocampus and amygdala of intact rats and had no influence on dopamine levels in hippocampus and amygdala of old Tx rats. The obtained data provide evidence of close relationship between hypothalamus-pituitarythyroid axis and serotoninergic system in young and old rats.

# Abstract: ANATOMICAL ORGANIZATION AND DOPAMINE TRANSPORTER EXPRESSION IN THE DOPAMINERGIC GROUPS OF THE MACAQUE MONKEY BRAIN

**Authors:** M. A. Sánchez-González, C. Cavada

**Affiliations:** Departamento de Anatomía, Histología y Neurociencia, Universidad Autónoma de Madrid, Spain

The current schema of dopaminergic (DAergic) neuronal populations includes 10 groups (A8 to A17) based on the nomenclature introduced by Dahlström and Fuxe in 1964 for the rat brain. After re-examining the anatomy of the DAergic populations in the macaque monkey brain using tyrosine hydroxylase (TH) immunohistochemistry, we performed dopamine transporter (DAT) and DAT/TH immunohistochemistry. Some differences were found between the monkey and rat DAergic groups. Group A11 holds abundant DAergic neurons along the whole periaqueductal gray; for this reason we do not consider A11 a diencephalic group, and we call it periaqueductal group. Group A13 (called in the rat dorsal hypothalamic group) is formed in the monkey by neurons located more laterally, ventrally and rostrally than in the rat. Because of its wide extension and because it is the only hypothalamic group not bordering the 3rd ventricle, we call A13 lateral hypothalamic group. In addition, we subdivide it into A13r (reaching rostrally the preoptic region) and A13dm-dha (for dorsomedial nucleus and dorsal hypothalamic area). TH-positive neurons, not included in none of the accepted DAergic groups, are found in the lateral parabrachial nucleus, the basal telencephalon and the striatum. DAT expression is intense in the mesencephalic groups A8–A10, as reported for the rat and the human. In the dorsal tier (A8 dorsal, A9 dorsal, A10) DAT expression shows micro-regional heterogeneity, in which neurons with different levels of DAT are intermingled, including some with no detectable immunoreactivity. DAT also appears outside the mesencephalon. Some DATpositive neurons appear in the caudal half of A11; thus we subdivide A11 into A11r (rostral part with no DAT) and A11c. In addition, some slightly DAT-positive neurons are found in the basal telencephalon. No DAT-positive neuronal somatas appear in the lateral parabrachial nucleus, the hypothalamus or the striatum.

# Abstract: IS IT POSSIBLE TO INFLUENCE THE EFFECTS OF A FOREBRAIN CHOLINERGIC DEPLETION? AMORPHOLOGICAL STUDY OF III-LAYER PYRAMIDAL PARIETAL NEURONS

**Authors:** P. De Bartolo, F. Gelfo, L. Petrosini, M. G. Leggio

**Affiliations:** ^1^Istituto de Ricovero e Cura a Carattere Scintifico, Santa Lucia, Rome, Italy; ^2^Department of Psychology, University of Rome “La Sapienza”, Rome, Italy; ^3^Faculty of Motor Sciences, University of Naples “Parthenope”, Naples, Italy

One of the most intriguing challenge of the contemporary neurosciences is to identify instruments for contrasting the cognitive decay inescapably present in patients affected by Alzheimer Disease (AD). A large body of data indicates that the cognitive deficits in AD are due to the loss of cholinergic input from basal forebrain structures to the cortex and hippocampus. Various animal paradigms have been developed tomodel these cholinergic dysfunctions and, at present, the immunotoxin 192 IgG-saporin is considered one of the most effective methods for selectively destroying cholinergic neurons of the basal forebrain. Aim of the present study was to analyse the influence of environmental (rearing in enriched environment) and pharmacological (acetylcholinesterase inhibitors, Donepezil) factors on the effects of 192 IgG-saporin-induced cholinergic depletion in the dendritic morphology of III-layer pyramidal neurons of the rat parietal cortex. The neuronal analysis was performed by taking into account dendritic branching and spine density. These indices allow inferring changes in the synapse activity, given the dendrites receive more than 95% of the synapses of the neuron. In lesioned animals reared in standard conditions, the parietal neurons exhibited increased synaptic spine number and reduced dendritic arborisation. In enriched lesioned animals, the parietal neurons maintained the higher level of spine density provoked by the enriched rearing although they presented a reduced dendritic arborisation. Preliminary data suggest that in the III-layer parietal neurons the morphological modifications induced by cholinergic depletion are not significantly influenced by the administration of an acetylcholinesterase inhibitor. These findings indicate that exogenous factors, as the environmental enrichment, are able to affect the neuronal modifications related to the cortical cholinergic depletion and suggest a possible anatomical background for the “brain reserve” hypothesis.

# Abstract: SYSTEMIC BIOCHEMICAL ALTERATIONS IN BRAIN IN SCHIZOPHRENIA

**Authors:** I. S. Boksha

**Affiliations:** Mental Health Research Center RAMS, Moscow, Russia

Systemic approach is used to establish a relationship between amounts of enzymes of glial glutamate and energy metabolism (glutamine synthetase and glutamine synthetase-like protein, glutamate dehydrogenase isoenzymes, brain isoform creatine phosphokinase) and two major glial proteins (glial fibrillary acidic protein and myelin basic protein) in autopsied brain samples taken from patients with schizophrenia and mentally healthy subjects. These biochemical parameters were measured in tissue extracts in three brain areas (prefrontal cortex, caudate nucleus, and cerebellum). Significant differences in the level of at least one of the glutamate metabolizing enzymes were observed between two studied groups in all studied brain areas. Different patterns of correlative links between the biochemical parameters were found in healthy and schizophrenic brains. These findings give a new perspective to our understanding of the impaired regulation of enzyme levels in the brain in schizophrenia.

# Abstract: Theme E: Emotion, stress EARLY LIFE EXPERIENCES ENTAILING SUBTLE CHANGES IN THE EARLY SOCIAL ENVIRONMENT FAVOUR BEHAVIORAL AND NEURAL PLASTICITY IN MICE

**Authors:** L. T. Bonsignore, F. Capone, L. Aloe, E. Alleva, F. Cirulli

**Affiliations:** ^1^Section of Behavioral Neuroscience, Department of Cell Biology and Neurosciences, The Istituto Superiore di Sanità, Viale Regina Elena 299, 00161 Rome, Italy; ^2^Institute of Neurobiology and Molecular Medicine, CNR, European Brain Research Institute, Via Fosso di Fiorano 64/65, 00143 Rome, Italy

Early life experiences, such as early handling, can impact on neural development of rodents leading to changes in physiological and behavioral reactivity to stress. These effects are likely to be mediated by changes in maternal behaviour. This study analysed the effects of different manipulations of the rearing environment on maternal behaviour and the behavioral and physiological response to mild challenges in CD-1 mouse pups early during development. Litters underwent either 15 min of neonatal handling (H) or were exposed briefly to an unfamiliar male intruder from postnatal (PND) day 2–14 (MI). Both groups were compared with litters which were not manipulated (NH). Compared to NH subjects, maternal behaviour in the MI group was increased only on the first day of introduction of the male intruder, while the H group showed an increase in maternal behaviour also on PND 10. As for aggressive behaviour expressed by the MI dams, the number of attacks towards the male intruder decreased significantly, while social interactions and non-social activities increased over subsequent encounters. On PND 8, pups ultrasonic vocalizations were recorded upon treatment with an axiolytic drug (chlordiazepoxide 0, 2, or 7.5 mg/kg). Results indicate that, in the absence of basal changes, compared to the NH and MI groups, handled subjects did not reduce their calling rate following drug administration. Following maternal separation and novelty exposure on PND 9, levels of hippocampal NGF increased significantly only in the H group. These data suggest that active pup manipulations in the form of handling favour behavioral and neural plasticity resulting in the maintenance of an adequate level of arousal and in increased neurotrophin levels in response to an acute manipulation. Changes in hippocampal levels of NGF might be involved in the appraisal of subtle changes in the early social environment.

# Abstract: EARLY ADVERSITY AFFECTS NERVE GROWTH FACTOR AND BRAIN-DERIVED NEUROTROPHIC FACTOR AND DISRUPTS NEUROBEHAVIORAL DEVELOPMENT IN MACAQUES

**Authors:** F. Cirulli, N. Francia, F. Capone, L. Aloe, S. J. Suomi, E. Alleva

**Affiliations:** ^1^Behavioral Neuroscience Section, Department of Cell Biology and Neurosciences, The Istituto Superiore di Sanità, Rome, Italy; ^2^Institute of Neurobiology and Molecular Medicine, (CNR-EBRI), Rome, Italy; ^3^Laboratory of Comparative Ethology, NICHD, Poolesville, MD, USA

In humans, both genetic and experiential factors can shape individual vulnerability to psychiatric illness. However, the quality and quantity of experience predisposing an individual towards psychopathology and the specific neural substrates affected are still open questions. Among those factors involved in brain development and function, neurotrophins appear as good candidates for mediating long-term effects of experience on brain function. We have taken a comparative approach using both rodents and primates (rhesus macaques) to test the hypothesis that changes in the levels of neurotrophins (i.e., NGF and BDNF) during critical periods of brain development might be considered as markers of neuroplasticity. We have shown that, in rodents, brain levels of neurotrophins are sensitive to manipulations of the mother-infant relationship. We have recently measured peripheral levels of NGF and BDNF in rhesus macaques exposed to early stress (reared in the presence of peers, rather than by the mother). Results indicate that plasma levels of neurotrophins are increased by peer rearing, suggesting that these markers of brain plasticity might also be peripheral measures of early stress in primates, possibly underlying the disfunctional social behavior characterizing these subjects.

# Abstract: SIMPLE DEVICES FOR MULTIPLE UNIT ACTIVITY RECORDINGS IN FREELY MOVING RATS

**Authors:** A. Tóth, Z. Petykó, I. Szabó, K. Máthé, M. Katona, A. Czurkó, R. Gálosi, L. Lénárd

**Affiliations:** ^1^Analogical and Neural Computing Systems Laboratory, Computer and Automation Researdh Institute of the HAS, Hungary; ^2^Institute of Behavioral Sciences; ^3^Neurophysiology Research Group of the HAS and Institute of Physiology, Pécs University, Medical School, Hungary; ^4^Faculty of Engineering, Institute of Electronics, Pécs University, Hungary

Multi-channel multi-unit recording techniques require specific skills and sophisticated tools. In order to make the recordings easier we designed and tested several devices such as

- PCB microdrive
- headstage preamplifier with
- galvanically isolated power supply
- different tetrode selectors.

We re-designed low-cost and light-weight multi-channel

- **PCB microdrive**. This new design led to improve stability and applicability for recording field potentials, EEG and extracellular multiple unit discharges up to 64/128 channels. We assembled a 32-channel unity gain
- **headstage preamplifier** from low noise CMOS current opamps and developed a
- **galvanically isolated power supply board**. The advantage of this board is that there is no need for using rechargeable batteries. Applying the microdrive and the preamplifier it is advisable to implant 8 tetrodes and to select 2 or 4 of them providing the successful spike sorting. For this purpose our
- **tetrode selector devices are** really useful for the laboratories having 8 or 16-channel main amplifier set and ADC card.

These devices were successfully tested in rats in different behavioral paradigms such as tread-mill and T-maze experiments and during startle reaction.

# Abstract: FIRING RATE PATTERNS IN MEDIAL PREFRONTAL CORTEX NEURONS DURING GLUCOSE SOLUTION INTAKE IN FREELY MOVING RATS

**Authors:** Z. Petykó, A. Tóth, I. Szabó, R. Gálosi, L. Lénárd

**Affiliations:** ^1^Neurophysyology Research Group of the Hungarian Academy of Sciences, Institute of Physiology, Pécs University Medical School, Hungary; ^2^Institute of Physiology, Pécs University Medical School, Hungary; ^3^Institute of Behavioral Sciences, Pécs University Medical School, Hungary; ^4^Analogical and Neural Computing Systems Laboratory, Computer and Automation Rearch Institute of the Hungarian Academy of Sciences, Hungary

The prefrontal cortex is associated with the central control of feeding, acquisition of conditioned taste aversion, rewarding mechanisms, hedonic evaluation of nutrients and glucose preference. There are only a few data, however, about the single neuron activity in the medial prefrontal cortex (mPFC) related to feeding and taste preference. In the present experiments multiple unit recordings were made in the mPFC of freely moving wistar rats during consumption of sugar solution. Eight tetrodes with multichannel PCB microdrive were chronically implanted and a tetrode selector was applied. During the 2 hour test period animals had free access to 5% glucose solution or water. Multiple unit activity and behavioral actions (drinking periods and other behavioral patterns such as sniffing, rearing, grooming) were continuously recorded. Two tetrodes were selected for data sampling. Single unit activity was separated offline with principal component analysis. mPFC neurons exhibited the following response patterns: groups of neurons responded with an increased firing rate during glucose consumption, while other neurons responded with inhibition. A distinct group of neurons responded 2–3 seconds before the rats began to drink glucose solution and these responses decreased for the end of the test period. Our results suggest that the mPFC is involved in the hedonic evaluation of glucose consumption in a complex manner.

# Abstract: NEURAL MECHANISM FOR SUPPRESSION OF AGGRESSION IN THE MOUSE

**Authors:** H. Shin, C. Kim, D. Jeon, Y. Kim, C. Lee, U. Oh, H. Kim

**Affiliations:** ^1^Center for Neural Science, Korea Institute of Science & Technology, Seoul, South Korea; ^2^Depatment of Anatomy, Korea University, College of Medicine, Seoul, South Korea; ^3^Institue of Neuroscience, Medical School of Inje University, Pusan, South Korea; ^4^Sensory Research Center, CRI, Seoul National University, College of Pharmacy, Seoul, South Korea

Aggression is normally under control in mammals, but how it is controlled is not well understood. Mice lacking the alpha1B subunit (Cav2.2) of N-type Ca^2+^ channels showed enhanced aggressive behaviors, an elevated level of arginine vasopressin (AVP) in the cerebrospinal fluid and plasma, and an increased amount of serotonin (5-HT) in the hypothalamus. A ventricular infusion of an AVP receptor antagonist or a microinjection of a 5-HT neurotoxin into the anterior hypothalamus reduced the aggressiveness of Cav2.2−/− mice to a wild-type level, indicating that an enhancement of the 5-HTAVP system underlies the aggression phenotype of Cav2.2−/− mice. 5-HT neurons in Cav2.2−/− dorsal raphe nucleus (DRN) exhibited higher spontaneous firing rate than wildtype controls, accompanied by the reduced inhibitory transmission. These results suggest that N-type Ca^2+^ channels in the inhibitory synapses in the DRN as a critical component in the control of aggression by down-regulating the 5-HT-AVP system. Our results reveal potential targets for suppression of aggression in mammals.

# Abstract: EXPRESSION OF MATERNAL BEHAVIOR IN THE RAT IS ASSOCIATED WITH ALTERATIONS IN DOPAMINE AND SEROTONIN RECEPTORS IN THE BRAIN OF THE MOTHER

**Authors:** A. Tzanou, A. Stamatakis, F. Stylianopoulou

**Affiliations:** Laboratory of Biology-Biochemistry, University of Athens, Athens, Greece

Dopamine and serotonin play important roles in triggering and regulating maternal behavior through their cellular receptors, within behaviorally relevant brain areas. Dopamine receptor antagonists disrupt ongoing maternal behavior for example pup-retrieval and nest building. Moreover, variations in nucleus accumbens dopamine are correlated with individual differences in maternal behavior. On the other hand, serotonin system function is increased during lactation and its activation induces various forms of maternal behavior. Expression of maternal behavior towards the offspring is increased following “neonatal handling”. We employed this experimental paradigm in order to determine the effect of maternal behavior on the levels of D1 and serotonin 1A (5-HT1A) receptors (by in vitro autoradiographic ligand binding) in areas of the limbic system (hippocampus and prefrontal cortex) and in the basal ganglia of lactating mothers. In the brain of lactating mothers, levels of D1 receptors were increased in the striatum, nucleus accumbens and tuberum olfactorius, while levels of 5-HT1A receptors were decreased in the prefrontal cortex and in the CA1, CA4 and DG. Moreover, the reduction in 5-HT1A receptors was more pronounced in the DG of mothers whose pups were subjected to “handling”. These localized alterations in D1 and 5-HT1A receptors during lactation support their role in the regulation of distinct aspects of maternal behavior: D1 receptors are probably involved in the regulation of motivational and motor components of maternal behavior by the basal ganglia, while 5-HT1A receptors in the modulation of emotional reactions by the limbic system during the post-partum period. Moreover, alterations in the neurochemistry of the limbic system could possibly explain the modifications of maternal behavior following handling of the pups.

# Abstract: EFFECTS OF AN EARLY LIFE EXPERIENCE ON DOPAMINE AND OPIOID RECEPTORS IN THE RAT BRAIN

**Authors:** A. Stamatakis, G. Kiosterakis, M. Vlassi, A. Tzanou, F. Stylianopoulou

**Affiliations:** Laboratory of Biology-Biochemistry, University of Athens, Athens, Greece

Early life events, particularly the mother-infant interactions, have been recognized as major factors in determining adult behavior. An animal model altering maternal behavior is “neonatal handling”, which has been shown to affect the programming of the hypothalamic-pituitary-adrenal axis and thus the responsiveness to stressful stimuli. Moreover, neonatally handled animals are known to exhibit as adults addictive-like behaviors, for example, increased consumption of palatable, sweet food and increased initial preference for cocaine. Since the dopaminergic and opioid systems have been implicated in the etiopathogenesis of addictive-like behaviors, we investigated the effects of “neonatal handling” on the distribution of D1 and D2 receptors (by in vitro autoradiographic ligand binding) as well as of mu-opioid receptors (by immunohistochemistry and in vitro autoradiographic ligand binding) in the cortex, hippocampus and basal ganglia of adult animals of both sexes. “Neonatal handling” resulted in increased levels of D1 receptors in the prefrontal cortex and nucleus accumbens only of male animals. In the striatum handled males had higher numbers of D1 receptors compared to the respective non-handled, while the opposite held true for the females. On the other hand, D2 receptors were not affected by “neonatal handling”. Mu-opioid receptors were increased in the nucleus accumbens and in the CA3 and CA4 hippocampal areas of handled animals of both sexes, compared to the respective non-handled. These data indicate that “neonatal handling” has long-term effects on the dopaminergic and opioid systems of the brain, specifically in areas involved in the control of addictive-like and emotional behaviors.

# Abstract: RECURRENT INHIBITION OF CONTACT OF THE RAT PUP WITH ITS MOTHER HAS SHORT AND LONG-TERM EFFECTS ONMU-OPIOID RECEPTOR LEVELS IN THE BRAIN OF THE OFFSPRING

**Authors:** G. Kiosterakis, A. Stamatakis, F. Stylianopoulou

**Affiliations:** Lab of Biology-Biochemistry, University of Athens, Athens, Greece

The opioid system plays an important role in the formation of the mother-infant attachment bond. Mu opioid receptors have been shown to mediate in the pups the reward inherent in maternal contact. Based on the above we employed an experimental model of perturbed mother-pup contact and determined immunohistochemically the levels of mu-opioid receptors in the brain of the offspring as neonates as well as adults. In our model rats were exposed during post-natal days 10 to 13 to a T-maze containing their mother at the end of its right arm. Two experimental groups were used: non frustrated rats which, upon finding the entrance of the mother-cage, were allowed to enter through a sliding door so that animals were retrieved by their mother and frustrated rats which, upon finding the cage, were not allowed to enter, since the sliding door remained closed, thus inhibiting tactile contact between the mother and the infant. On P13, two hours after the last exposure to the T-maze, frustrated rats had significantly increased numbers of mu-opioid receptor immunopositive cells in the basolateral and central amygdaloid nuclei as well as in nucleus accumbens, whereas at 3 months of age frustrated animals showed higher levels of mu-opioid receptors in nucleus accumbens and in the CA4 hippocampal region, compared to the non-frustrated and naïve rats. This increase could reflect a compensatory mechanism elicited by the inhibition of the mother-pup tactile contact. Interestingly, this increase is localized in the amygdala, nucleus accumbens, and the hippocampus, brain areas known to play a cardinal role in detecting and responding to motivationally and emotionally significant stimuli. The alterations induced by the perturbed mother-infant interaction are long-lasting, extending into the adulthood and possibly influencing the behavior of the adult organism.

# Abstract: ANXIETY IN INTELLICAGE: A SIMPLE TEST PROCEDURE FOR EVALUATION OF ANXIOLYTIC DRUG ACTION

**Authors:** S. Kamran, N.-W. Frieder, W. Hans, L. Hans-Peter

**Affiliations:** ^1^NewBehavior AG, Hardturmstrasse 76, 8005 Zürich, Switherland; ^2^Anatomisches Institut, Universität Zürich, Winterthurerstrasse 190, 8057 Zürich, Switherland

Anxiety disorders are the most common mental illness in the U.S. with 19.1 million (13.3%) of the adult U.S. population affected. Animal models have a fundamental contribution to make in the area of anxiety research both at the clinical and industrial level as well as at the scientific level. We used a modified Vogel water lick conflict for mice in IntelliCage. The anxiogenic effect of our treatment in IntelliCage was tested with Diazepam as a well-known anxiolytic drug. After a deprivation period of 18 hours 12 mice were observed simultaneously in three different conditions: punished drinking, punished drinking after injection of Diazepam and finally punished drinking with injected saline. For each individual IntelliCage allowed us to observe and quantify automatically many different behavioral measures. None of these measures differed between the punished drinking and punished drinking with saline injection treatments. Animals treated with Diazepam, however, showed in several aspects significant differences compared with the two other treatments indicating an increased resistance against the aversive stimulus. The results were robust and remained unchanged after correction for individual differences and differences in activity due to the medication. Our study shows that IntelliCage can be used to assess anxiolytic drug action and recommends it for fast and efficient anxiety research.

# Abstract: AN AUTOMATED ARENA WHICH CONTINUOUSLY RECORDS ACTIVITY AND COMPLEX SOCIAL INTERACTIONS IN GROUPS OF MALE MICE

**Authors:** M. J. Galsworthy, A. E. Rau, F. Neuhaeusser-Wespy, A. L. Vyssotski, C. D. Blanchard, R. Blanchard, H. Lipp

**Affiliations:** ^1^Institute of Anatomy, University of Zurich, Switherland; ^2^NewBehavior AG, Zurich, Switzerland; ^3^Department of Psychology, University of Honolulu, HI, USA

Here we report the successful use of an automated version of the visible burrow system (VBS), in which mice are continuously monitored as social hierarchies form and experimental manipulations are implemented. By utilizing the combined technologies of implanted micro-transponders on each mouse and in-cage antennae, individual mice are continually monitored in real-time. The resultant data are capable of identifying the dominant mouse by his patrolling behaviour and chasing of other mice through the tubes. In pilot groups of 7 mice/group, the dominant male (as identified by initial observation and external indices such as tail bites) regularly shows substantially higher activity than all other mice, and will be the “chaser” in 74% to 94% of chase events. These chase events are coded as two mice passing through the same 50 cm long tube in the same direction, in less than 2 seconds (ie running), and the two mice being less than 2 seconds apart in their completion of the event (ie chasing). These chase events are then broken down into who chases whom, and when and where they happen. Further to monitoring such data, we also demonstrate that in groups with lower stress levels (as measured by weight loss and tail bites), it appears as though the stress/aggression level can be augmented by introducing a female mouse into the arena, but inaccessible through a wire grid. Future potential manipulations include limiting or presenting food or water access in different parts of the arena or at different times in order to apply or reduce stress generally, or to specific animals, or study rank-ordering in resource access. The general aim is to utilize the automatic recording to develop social scenarios for study and to identify early indicators of stress and depression and explore methods of early intervention.

# Abstract: EFFECTS OF INTRA-AMYGDALOID NEUROTENSIN ON SPATIAL LEARNING AND PASSIVE AVOIDANCE

**Authors:** K. Lászlo, K. Tóth, E. Kertes, K. Oláh-Várady, R. Bárdosi, L. Lénárd

**Affiliations:** ^1^Institute of Physiology and Neurophysiology Research Group of the HAS, Hungary; ^2^Pécs University Medical School, Pécs, Hungary

Tridecapeptide Neurotensin (NT) was first isolated from bovine hypothalamus. In the central nervous system NT acts as a neurotransmitter and neuromodulator. It was indicated that microinfusions of NT antagonist into the nucleus accumbens core impair spatial learning. The central nucleus of amygdala (ACE), part of the limbic system, plays an important role in learning, memory, regulation of anxiety and emotional behaviour. It has been demonstrated that the amygdaloid body is rich in NT immunreactive elements and NT-1 receptors.

The aim of our study was to examine in the ACE the possible effects of NT on spatial learning in Morris water maze (MWM) paradigm and on passive avoidance learning (PAV) in two-compartment passive avoidance paradigm. In MWM test male wistar rats were microinjected bilaterally with 100 ng NT or 250 ng NT (Sigma: N 3010, dissolved in sterile saline, injected in volume of 0.4 *μ*l) or NT-1 receptor antagonist SR 48692 100 ng (Sanofi-Synthelabo) alone, or NT-1 receptor antagonist 15 min before 250 ng NT treatment or vehicle solution into the ACE. Application of 100 ng NT or 250 ng NT significantly reduced latency to find the safe platform located in one of the quadrants of the maze. Prior treatment with the non-peptid NT-1 receptor antagonist (SR 48692), equimolar to NT treatment (0.18 nM and 0.18 nM, respectively) blocked the effects of NT. Antagonist in itself did not influence the spatial learning. In PAV animals were shocked with 0.4 mA and subsequently were microinjected bilaterally with 100 ng NT or 250 ng NT or vehicle solution into the ACE. Both doses of NT significantly increased the latency time. Our results show that in the rat ACE NT facilitates place learning, memory and passive avoidance.

# Abstract: YOU SHAN'T STRESS THE ANXIOUS OR WHEN LEARNING THE HARD: EXPERIMENTS AND MODELLING IN STUDYING STRESS EFFECTS ON BEHAVIORAL LEARNING

**Authors:** G. Luksys, J. Delile, D. Sheynikhovich, C. Sandi, W. Gerstner

**Affiliations:** Ecole Polytechnique Fédérale de Lausanne (EPFL), Lausanne, Switzerland

Acute stress regulates different aspects of behavioral learning through the action of stress hormones and neuromodulators. Stress effects depend on its type, intensity, timing, and the learning paradigm. In addition, the animals genetic background might be an important factor in determining how stress influences their performance. In our conditioning and spatial learning experiments, we expose 2 different mouse strains (C57BL/6 and DBA/2) to extrinsic and motivational stressors. We also study how learning is affected by stress in the standard Morris water maze task vs. a more difficult task with variable platform location. Our results suggest that stress affects learning in a strain dependent fashion, and that its effects on learning and performance may be opposite depending on task difficulty. We then describe how mouse behaviour can be formalized and studied using reinforcement learning (RL) models. In this framework, effects of stress and genetic background on learning and memory can be attributed to differential dynamics of model metaparameters (such as learning rate or exploitation-exploration factor), which are thought to be related to activity of certain neuromodulators in the brain. We provide the main results of RL meta-parameter studies and discuss them in relation to standard behavioral analysis' approaches.

# Abstract: NEONATAL ODOR-SHOCK CONDITIONING ALTERS THE NEURAL NETWORK INVOLVED IN ODOR FEAR LEARNING AT ADULTHOOD

**Authors:** Y. Sevelinges, P. J. Holman, B. Messaoudi, R. M. Sullivan, A. M. Mouly

**Affiliations:** ^1^Neurosciences Sensorielles, Comportement, Cognition, CNRS-Université Lyon 1, France; ^2^Institut des Sciences Cognitives, CNRS-Université Lyon 1, France; ^3^Zoology, University of Oklahoma, Norman, OK 73019, USA

Neonatal olfactory fear conditioning paradoxically causes odor preferences in rat pups during a temporally defined sensitive period. With emergence of amygdala participation in fear conditioning at 10-days-old, pups readily learn to avoid odors paired with shock (0.5 mA). We assessed the effects of odor-shock pairing during the sensitive period, -leading to an odor preference-, on later adult olfactory fear conditioning.

Infant rats were trained daily from 8–12 days old in a fear conditioning paradigm and assigned to different experimental groups: Paired (peppermint odor-0.5 mA shock),Unpaired, Odor-only and Naive. In adulthood, animals from each of the 4 infant conditioning groups were trained with paired odor (peppermint)-shock (0.5 mA) associations. Animals were then tested for freezing to the presentation of odor alone. In parallel, amygdala (basolateral complex and cortical nucleus) and piriform cortex (anterior and posterior parts) functioning was assessed using 2-DG autoradiographic mapping and field potential recordings following electrical stimulation of the olfactory bulb.

Infant Naïve/Adult Paired animals readily learned to freeze to the odor paired with shock in adulthood, as well as Infant Unpaired/Adult Paired and Infant Odor-only/Adult Paired animals, suggesting that infant pre-exposure to either shock or odor, including unpaired presentations, did not alter the adult conditioning. In contrast, animals that received both infant and adult odor-shock pairing (Infant Paired/Adult Paired) showed significantly lower levels of freezing compared to animals in the other groups.

The attenuated fear learning observed in Infant Paired/Adult Paired animals was accompanied by a lower level of 2DG uptake in the amygdala (basolateral complex and cortical nucleus) and in the posterior piriform cortex. In the same structures, a decrease in paired-pulse inhibition was also observed in the recorded field potential signals.

The present data suggest that odor-shock learning during the sensitive period attenuates adult odor fear learning. This deficit is associated with lasting neurobiological alterations at the level of the amygdala and posterior piriform cortex.

# Abstract: ENHANCED SPATIAL MEMORY AND REDUCED EMOTIONALITY IN THE P66SHC−/− MOUSE, A MODEL OF REDUCED OXIDATIVE STRESS

**Authors:** A. Berry, M. Giorgio, P. Pelicci, E. de Kloet, E. Alleva, L. Minghetti, F. Cirulli

**Affiliations:** ^1^Section of Behavioral Neurosciences, Department of Cell Biology and Neurosciences, Istituto Superiore di Sanitd, Rome, Italy; ^2^LACDR Division of Medical Pharmacology, Leiden University, Leiden, The Netherlands; ^3^Division of Experimental Oncology, Istituto Europeo di Oncologia, Milan, Italy; ^4^Section of Degenerative and Inflammatory Neurological Diseases, Department of Cell Biology and Neurosciences, Istituto Superiore di Sanitd, Rome, Italy

The p66Shc mammalian gene mediates oxidative stressinduced apoptosis. P66Shc−/− mice (KO) show reduced levels of oxidative stress and live longer than the wild-type (WT) phenotype. We hypothesis that in KO mice reduced exposure to reactive oxygen species throughout life may slow down brain ageing, leading to better cognitive performance. Adult (4-months-old) and old (24-months-old) WT and KO mice were assayed for spatial memory in a Morris water maze (MWM). Since emotionality is a modulator of behavioral responses, adult mice underwent an open field test to assess emotional reactivity and a tail flick to assay nociception. To further characterise the KO phenotype basal levels of brainderived neurotrophic factor (BDNF: a neurotrophin involved in several aspects of memory retention, mood disorders and pain sensitivity) were assessed in the hippocampus and cortex of adult and old mice. KO adult subjects were characterised by significantly better memory performance in the MWM, lower emotionality and a higher pain threshold when compared to WT mice. Moreover, basal levels of BDNF were significantly higher in the hippocampus of KO adult than in the WT subjects. Old subjects were unable to learn the MWM task, although KO mice showed overall a better physical performance. These results overall show that oxidative stress not only affects physical aspects of ageing, as seen in old subjects but, more interestingly, can modulate responses to painful or arousing stimuli and lead to better cognitive performance (adult mice) pointing to this gene as a novel target for studying the mechanisms underlying emotionality.

# Abstract: NEUROCHEMICAL CHANGES IN AMYGDALA AND PIRIFORM CORTEX REVEALED BY MICRODIALYSIS DURING OLFACTORY FEAR CONDITIONING

**Authors:** S. Parrot, Sevelinges, R. Gervais, A. Mouly

**Affiliations:** ^1^NeuroChem Department, Université Lyon 1, France; ^2^Neurosciences Sensorielles, Comportement, Cognition, CNRS Université Lyon 1, France; ^3^Institut des Sciences Cognitives, CNRS-Université Lyon 1, France

In a previous study using multisite electrophysiological recordings, we showed that olfactory fear conditioning induces synaptic changes in the basolateral amygdala (BLA) and posterior piriform cortex (PPC) suggesting an involvement of these two structures in the learning. In order to further characterize the learning-induced modifications in these sites, the present work was aimed at monitoring the variations of neurotransmitter concentrations in BLA and PPC during odor fear acquisition. For that purpose, high temporal resolution (1-min sampling rate) microdialysis experiments were carried out on freely-moving Long-Evans rats receiving either six odor(CS)-shock(US) pairings (Trained group) or six presentations of odor alone (Control group). Simultaneous monitoring of GABA and Glutamate was performed in both PPC and BLA during the conditioning session in both groups. In trained animals, a transient increase in Glutamate was observed in BLA for the two first CS-US pairings as compared to control rats, whereas a significant increase in GABA levels was shown for the second pairing only. The most significant variations were observed in PPC: transient increases in both GABA and Glutamate levels were found after each odor-shock pairing, the amplitude of enhancement being greater for GABA (+25–50%) than for Glutamate (+10–25%). In addition in trained animals, for both neurotransmitters, the increase observed in BLA occurred earlier (around 1–2 min) than in PPC. The present data are in agreement with our previous electrophysiological data, which confirms the involvement of BLA and PPC in odor fear learning. In addition high temporal resolution microdialysis allowed us to show a temporal dynamic of activation of these structures during the course of successive pairings, with the involvement of BLA during the first odor-shock associations preceding neurotransmitter release in PPC, after which PPC alone shows learning-induced modifications.

# Abstract: NORADRENERGIC SYSTEM IN THE BASOLATERAL AMYGDALA DURING CONDITIONED ODOR AVERSION LEARNING IN THE RAT: ALPHA1-ADRENERGIC RECEPTORS IN THE GAME

**Authors:** B. Ferry, L. Estrade, R. Gervais

**Affiliations:** LNSCC, UMR 5020 Centre Nantionale de la Recherche Scientifique, 50 avenue Tony Garnier, 69366 Lyon, France

Conditioned odor aversion (COA) corresponds to the avoidance of an odorizedtasteless solution (conditioned stimulus, CS) previously paired with toxicosis. COA occurs only when the interstimulus interval (ISI) is kept short, suggesting that the memory trace of the odor is subject to rapid decay. Previous experiments have shown that the basolateral amygdala (BLA), is involved in the processes that control the olfactory memory trace during acquisition of COA. More precisely, some recent results have shown that catecholamine depletion, as well as infusion of propranolol in the BLA impaired COA. Since *β*- but also *α*1-adrenoceptors are present in the BLA, the present experiment investigated what part played the *α*1-adrenoceptors in the effect induced by the catecholamine depletion on COA. Male Long-Evans rats bilaterally implanted with cannulae aimed at the BLA were exposed to odor-toxicosis pairing using a 15 min ISI. Three groups of rats received infusions of 0.1 *μ*g of the selective *α*1-antagonist prazosin either before (pre-CS) or after the CS presentation (post- CS) during the acquisition, or before the test (pre-test) of COA. Results showed that neither post-CS or pre-test infusions of Prazosin impaired COA. In contrast, pre-CS infusion of Prazosin induced a strong and clear deficit of COA. Since pre-test group diplayed a COA, the deficit observed in Pre-CS group was not due to a potential side effect of prazosin on an attentional, motivational or sensory process. More likely, it is suggested that pre-CS infusion of prazosin affected a process related to the to be conditioned olfactory memory trace duration. Therefore, and in accordance with our previously reported selective pre-CS effect of propranolol on COA, our result confirm that the noradrenergic system in the BLA is involved in the control of the olfactory memory trace during acquisition of COA, and suggest more precisely that the *α*1- adrenoceptors are involved, at least in part, in the mediation of this effect.

# Abstract: THE SENSITIZATION OF ACOUSTIC STARTLE REFLEX BY FOOTSHOCK IS PROLONGED IN RATS TREATED CHRONIC VARIABLE STRESS

**Authors:** S. Kanai, M. Nakano, D. Tanaka, A. Misonoo, K. Osada, M. Asakura

**Affiliations:** Department of Neuropsychiatry, St. Marianna University School of Medicine, 2-16-1 Suago, Miyamae-ku, Kawasakicity, Kanagawa, 216-8511, Japan

Corticotropin releasing hormone (CRH)- and norepinephrine (NE)-containing neurons in brain are activated during stress. We previously reported a decrease in the basal level of CRH immunoreactivity in the central nucleus of amygdala and the tyrosine hydroxylase immunoreactivity in the locus coeruleus after chronic variable stress (CVS) whereas both response was augmented by a novel stress. Since the acoustic startle reflex (ASR) can be enhanced by the CRH neuronal activity in the central nucleus of amygdala, we examined the influence of footshock on ASR in rats exposed to CVS. The footshock after CVS caused a significant augmentation of ASR compared with the acute footshock. Moreover, The enhanced startle to acute footshock was maximally increased at 6 min and was absent after 40 min, whereas the maximal change of the enhanced startle to footshock after CVS was delayed to 14 min and the significant enhanced startle was found until 180 min. The footshock-enhanced startle after CVS may be related to the augmentation of CRHNE activity, leading to the possibility that a prolonged CRH hyperactivity to stress might generate a pathophysiology of the major depression with a vulnerability to stress.

# Abstract: STUDY OF STRESS ON THE HIPPOCAMPUS FORMATION WITH ETHANOL ADMINISTRATION IN FEMELLES RATS AND THERE OFFSPRING

**Authors:** D. Harrouz, Z. Z. Birem, B. Asselah

**Affiliations:** Neurosciences of behavior and cognition, Algers, Algeria

The association between spatial learning and the hippocampus provides such a model. Studies of humans and animals with hippocampal lesions strongly suggest that spatial learning ability is reliant upon an intact hippocampus. This correlation between hippocampal cell loss and impaired spatial learning ability has been strengthened by similar evidence provided by animal models of FAS. Spatial learning deficits are one of the most commonly reported outcomes in animal models of FAS. These deficits have been observed following both prenatal neonatal and combined prenatal and neonatal alcohol exposure. The CA1 region of the hippocampus has also proven highly susceptible to cell loss following prenatal, neonatal, or combined prenatal and neonatal alcohol exposure.

In this study, we aimed to investigate whether exposure of femelle rats to chronic administration of ethanol prenataly could impaired cognitive decline during early age; such an effect could be dependent on the behavioral trait of reactivity to novelty displayed during young adulthood (4 months). Moreover, reducing cognitive learning in the Morris water maze was found to prevent loss of hippocampal neurons. Furthermore, we also aimed to identify neurobiological substrates during prenancy in rats that could correlate with their differential vulnerability to showcognitive impairments. We focused on the hippocampus for its role in learning and memory processes and its particular sensitivity to stress and brain aging; corticosteroid receptors for their critical involvement on the regulation of the hypothalamuspituitaryadrenal (HPA) axis and cognitive function.

**Conclusion**: These studies suggest that the effects of ethanol on hippocampus formation may have a role in the pathophysiology of Alcohol-Related Neurodevelopmental Disorders and Fetal Alcohol Syndrome.

**Keywords**: Stress; Ethanol; Hippocampus; HPA axis; Rat; Learning; Memory; Morris water maze.

# Abstract: BOUNDARY CONDITIONS OF CORTICOSTERONE-DEPENDENT ENHANCEMENT OR ALTERATION OF MEMORY CONSOLIDATION

**Authors:** N. Kaouane, L. Calandreau, F. Esclassan, S. Tronel, N. Mons, R. Jaffard, D. N. Abrous, P. V. Piazza, A. Desmedt

**Affiliations:** ^1^CNIC, CNRS UMR 5228, Université Bordeaux 1, Talence, France; ^2^INSERM U588, Université Bordeaux 2, France

Extensive evidence indicates that administration of the adrenocortical hormone corticosterone (CORT) facilitates the consolidation of emotional experience. Nevertheless, high release of CORT, which can occur in extreme stressful situation, can result in memory deficits. This suggests that beyond a certain level of stress, the influence of CORT on memory consolidation may switch from a facilitating effect to a deleterious effect, leading to maladaptive emotional responses with regards to the aversive learning experience. Nevertheless, the boundary conditions under which such a switch can be observed are still elusive.

In order to specify these conditions, we used in mice two fear conditioning procedures known to result in a preferential conditioned fear response either

- to a discrete tone (i.e., tone-shock pairing: the tone is predictive of a mild footshock occurrence) or
- to contextual cues (i.e., tone-shock unpairing: tone not predictive).

First, we show that increasing the intensity of footshock from a very low (0.3 mA) to a relatively high (1 mA) level gradually enhances conditioned fear responses in an adaptive manner, that is, as a function of the tone-shock contingency. However, beyond the critical footshock intensity of 1 mA, fear responses become maladaptive, that is, independent on the conditioning procedure.

Second, preliminary results indicate that under mild footshock intensity (0.5 mA), posttraining intrahippocampal infusions of CORT dose-dependently enhance adaptive conditioned fear responses. However, beyond a critical dose maladaptive conditioned fear responses are again observed. Finally, we also assessed whether the CORTdependent switch from adaptive to maladaptive fear responses was associated with changes in ERK1/2 activationrelated recruitment of the hippocampal-amygdalar circuit. Altogether, these results throw light on boundary conditions of CORT-dependent normal and pathological fear learning.

# Abstract: BEHAVIORAL ABNORMALITIES IN ADA-DEFICIENT MOUSE MODEL

**Authors:** R. Jofra Hernandez, P. D'Adamo, A. Aiuti

**Affiliations:** ^1^San Raffaele Telethon Institute for Gene Therapy (HSR-TIGET), Milan, Italy; ^2^Molecular genetics of mental retardation (DTI-DIBIT), Milan, Italy

Severe combined immunodeficiency due to the lack of adenosine deaminase (ADA-SCID) causes an accumulation of ADA substrates in plasma and cells leading to impaired immune functions, recurrent infections, and autoimmune manifestations in milder forms. In addition, neurological and behavioral alterations have been observed in a significant proportion of patients surviving after bone marrow transplant or enzyme replacement therapy. To test if these alterations are part of the disease phenotype we performed a detailed assessment of the learning and behavioral features in the ADA-deficient mouse model. ADA ko and wt mice littermates were subjected to the Fox battery test to assess their sensory and motor developmental abilities as well as to the RotaRod test to evaluate the motor coordination and neurological deficits. We found no significant differences between neither ADA ko nor wt mice nor female and male in their ability to perform FOX battery and RotaRod tests. We then investigated emotional, motivational and exploratory behavior using the open field and lightdark box test in mice 15 and 20 days old. We found that ADA ko mice were significantly less active in the open field as compared to ADA wt mice as assessed by different parameters. However, the speed of movement was not different between the groups. Remarkably, the time spent in the light compartment of the light-dark box was significantly decreased in ADA ko mice compared to control mice. Together these results suggest that the ADA ko mice are not physically unable to move but prefer to remain in safer zones, probably for anxiety-related reasons. As part of our future plans we will test the ability of different treatments (enzyme replacement therapy, bone marrow transplantation and gene therapy with hematopoietic stem cells) to rescue the observed phenotypes. These findings will contribute to improve our knowledge on the pathogenesis of the ADA-SCID as well as to the design of better therapeutic approaches.

# Abstract: SOCIAL DEPRIVATION IMPROVES PROCESSES OF FORMATION OF THE PASSIVE AVOIDANCE RESPONSE

**Authors:** I. Labadze, T. Domianidze, T. Matitaishvili

**Affiliations:** Department of Behavior and Cognitive Processes, Ins. of Physiology, Tbilisi, Georgia

In our previous experiments, deprivation of the social contacts at early stage of the postnatal development elicits the stable alterations in the emotion, learning and memory processes, in the mechanisms of adaptation to stressogenic environment. The purpose of present stude was investigation of an influence of the intraspecies deprivation on the passive avoidance response. Behavior of animals were investigated in the rats, were isolated since 14th day (for 8 weeks) and control animals. In order to elaborate the passive avoidance response, special two-compartment chamber was used. Experiments were carried out in three stages: I-Exploration, II-Learning, III- Retrieval. Thus has shown that in the postlearning period (Stage III) the time spent in the dark compartment decreased in both control and isolated animals that point at acquisition in the animals of the passive avoidance behavior. However the isolated animals when tested 24 hours after the learning, did not enter at all into the dark compartment unlike the control animals. Formation of the passive avoidance response depends on the competitive relation between the inner aversive (fear of open, well-lit space) reaction, on the one hand, and acquired avoidance response on the other. It was shown also that activation of the cholinergic system promotes acquisition and retrieval processes in the passive avoidance. Meanwhile, in the rats raised in conditions of the social isolation, a hypersensitivity of the brain acetylcholine-receptors does occur. Therefore, increase of the latent period of entrance into the dark compartment, found in the isolated rats, the authors explain as improved retention of the avoidance response in the memory. According to the above-said, we think that in the isolated rats, as compared to the control ones, dominates the avoidance reaction and deprivation of the social relations improves processes of formation of the passive avoidance response and its retention in the long-term memory.

# Abstract: ANATOMICAL EVIDENCES AND NEUROCHEMICAL CHARACTERIZATION OF A PONTO-SEPTAL PATHWAY VIA THE NUCLEUS INCERTUS UNDERLYING THETA RHYTHM IN THE RAT

**Authors:** A. Cervera-Ferri, A. Ruiz-Torner, A. A. Valverde-Navarro, A. Nuñez, F. Olucha-Bordonau, V. Teruel

**Affiliations:** ^1^Department Anat. y Embriol. Hum. Fac. Medic. Univ. Valencia, Valencia, Spain; ^2^Department Morfol. Fac. Medic. Univ. Aut. Madrid, Madrid, Spain

Hippocampal theta activity appears during memory encoding and retrieval. This oscillation appears during REM sleep and also in the awake animal during information-seeking behaviours and voluntary movements. The complete circuitry involved in its generation, maintenance or regulation remains unclear. Medial septum-diagonal band (MSDB) is considered the pacemaker of the rhythm. Pontine key structures activate (reticularis pontis oralis, RPO) or desynchronize the oscillation (median raphe, MR). The supramammillary nucleus (SuM) modulate theta frequency. Since the efferences of the nucleus incertus (NI) include MSDB, MR, SuM and HPC, this tegmental nucleus-belonging to the arousal system-could be part of theta circuitry. Recently we have verified in urethane-anaesthetized rats that NI neurons increased their activity under theta predominance induced by RPO stimulation or sensory stimuli. Moreover, NI stimulation elicited theta and NI lesion or inhibition abolished the synchronization induced by RPO. Our electrophysiological data suggested a pathway RPO-NI-MSDB. In order to verify this pathway, simultaneous injection of anterograde tracer in RPO and retrograde tracer in MSDB were performed, revealing double labelled neurons in NI. Subsequent inmunochemical detection of choline acetyl transferase (ChAT), parvalbumin (PV), calbindin (CB), calretinin (CR), orexin (OX) and glutamate vesicular transporter 2 (VGLUT2) was performed. The preliminary results of this study support the ideas that NI has to be considered as part of the subcortical theta rhythm circuitry and that its effect over theta is mediated at least by GABAergic transmission, with orexinergic influence. A possible circuit is proposed on the basis of the anatomical, neurochemical and electrophysiological data.

# Abstract: MODULATION OF ADULT BEHAVIOR AND BASAL CORTICOSTERONE SECRETION BY NEONATAL NOVELTY EXPOSURE IN RATS

**Authors:** N. Daskalakis, K. Anastasopoulos, S. Bezati, M. Kaperoni, E. Kitraki

**Affiliations:** ^1^Lab of Histology & Embryology, Athens Medical School, Athens, Greece; ^2^Division of Medical Pharmacology, Leiden/Amsterdam Center for Drug Research, Leiden, the Netherlands

Exposure of rodents to early life experiences can permanently affect adult brain functions concerning emotionality, stress response and synaptic plasticity. We have applied a “novelty exposure” protocol in rats of both sexes, frompostnatal day 2 to 21, to investigate effects on their exploratory behavior, spatial memory and HPA axis activity. This protocol differs from the widely used models of early handling or maternal separation, in that the only additional stimulus experienced by the pups is the environment of a novel cage (split litter design). In adulthood, animals were behaviorally tested in a Y-maze task and 1 week later they were blood sampled and killed in basal conditions. The task consisted of a 15 min-acquisition trial where only 2 arms were accessible and a 5 min-retrieval trial, 4 h later, where all 3 arms were accessible. In terms of general locomotion activity and motivation for exploration, female rats exposed to novelty made more entries in the accessible Y-maze arms compared to all other groups, when first exposed to the maze. In the retrieval trial, general locomotion was similar among all groups. Novelty exposure improved spatial memory and novel arm discrimination in both sexes, in a sexually dimorphic manner. On one hand, “novel” males visited the novel arm more often in the beginning of the second trial, but then splat their entries in known and start arms as well. On the other hand, “novel” females started trial 2 with a preference for novel armcombined with often visits to the known armand ended it with a clear preference for the novel arm. By the end of the retrieval, all animal groups progressively lost their interest for exploration, entering more the start arm. Novelty exposure increased basal corticosterone levels in adult females, but not males, and did not affect adrenal weight or histology in either sex.

# Abstract: SPATIAL LEARNING AND MEMORY ABILITIES ARE PREDICTED BY INDIVIDUAL DIFFERENCES IN BEHAVIORAL TRAITS

**Authors:** B. Salehi, M. Cordero, C. Sandi

**Affiliations:** Laboratory of Behavioral Genetics, Brain Mind Institute, Swiss Federal Institute of Technology (EPFL), Lausanne, Switzerland

Learning and memory may be influenced by different factors such as age, environment, behavioral styles, stress, or a combination of them. This work aims to study whether different behavioral traits in rodents (frequently stable in time and, therefore, regarded as a sort of “personality” traits) can predict performance in spatial learning and memory tasks in different individuals. Adult male Wistar rats were first characterized for their anxiety level, reactivity to explore novel environments and objects, social hierarchy in the homecage, and locomotor responses to novelty. For this purpose, we used a variety of behavioral tasks, including the Elevated Plus Maze, Open field, Novel object reactivity, Open field “emergency” test, Light and dark test, circular corridor, 16-hole board, social interaction and water competition tests. Subsequently, all rats were trained in the hippocampus-dependent Radial Arm Water Maze (RAWM) task to explore their spatial learning, long-term memory and reversal learning capabilities. Our results indicate that both exploration and social hierarchy are highly related to performance in the spatial learning and memory tasks. High explorative animals are better learners in the RAWM task than low explorative ones. Moreover, and surprisingly, alpha-rats (dominants) are clearly the worst learners among the three animals living together in the homecage. These results suggest that certain behavioral styles or “traits” (such as exploration, frequently related in the rodent literature to novelty-seeking behavior) and behavioral states (such as social hierarchy, which to a great extent develops with social experience) in rat are strong predictors of individuals spatial learning and memory abilities.

# Abstract: DIFFERENTIAL LIMBIC SYSTEM RE-ACTIVATION FOLLOWING SPATIAL MEMORY UNDER HIGHER VERSUS LOWER LEVELS OF STRESS

**Authors:** I. Kogan, G. Richter-Levin

**Affiliations:** The Brain and Behavior Research Center, University of Haifa, Haifa, Israel

Limbic system activity underlies emotional memory formation; however, the specific interactions among limbic elements (i.g., CA1, basolateral amygdala [BLA] and the entorhinal cortex [EC]) generating affective memory are obscure. Previously, we investigated ERK1/2 and CREB actvation pattern in these areas following spatial memory under different stress conditions. Learning under lower level of stress was found to involve predominantly CA1 while learning the same task under higher levels of stress involved also the BLA. In the present study we examined the pattern of activation of these areas following spatial memory re-activation under different levels of stress. Five groups of animals were tested: “learning” animals were trained either in the cold (higher stress) or in the warm (lower stress) water; “no platform” animals were subjected to the maze without a platform either in cold (higher stress) or in warm (lower stress) water; “naives” were not exposed to the maze. The “learning” and “no platform” animals were introduced to a water maze under cold or warm water for one trial after 24 h, and taken for biochemical analysis 10 min following the water maze procedure. In the present study increased ERK1/2 activation was detected in CA1, BLA and EC in both the learning and the no platform groups under higher stress conditions only. Whereas, CREB activation significantly marked a lower stress condition (i.e., warm water) in all areas examined. The data suggests diverse molecular pathways involvement in reactivation of the spatial memory generated under different stress conditions, thus implying on memory creation of a different quality under higher versus lower levels of stress. Moreover, the results further emphasize the role of the limbic system areas in emotional memory reactivation and indicate that the ERK1/2 and CREB are activated during memory reconsolidation in a different pattern than during memory formation.

# Abstract: CRITICAL ROLE OF THE 65KD ISOFORM OF GLUTAMIC ACID DECARBOXYLASE IN CONSOLIDATION AND GENERALIZATION OF PAVLOVIAN FEAR MEMORY

**Authors:** J. R. Bergado Acosta, K. Obata, H. Pape, O. Stork

**Affiliations:** ^1^Institute für Physiologie, Otto-von-Guericke Universität Magdeburg, Leipziger Strasse 44, D-39120 Magdeburg, Germany; ^2^Neural Circuit Mechanisms Research Group, RIKEN Brain Science Institute, 2-1 Hirosawa, Wako, Saitama 351-0198, Japan; ^3^Institute für Physiologie I, Westfälische-Wilhelms Universität Münster, Robert-Koch Strasse 27a, D-49149 Münster, Germany

Evidence suggests that plasticity of the amygdalar GABAergic system is critical for fear memory formation. Moverover, the key enzyme in GABA synthesis, glutamic acid decarboxylase 67 (GAD67) has been identified as a genetic factor in mood and anxiety disorders. In this study we investigated in wild type and genetically manipulated mice the role of a second, activity dependent GAD isozyme, GAD65, in the consolidation and generalization of conditioned fear. Firstly, we demonstrate a transient reduction of GAD65 gene expression in the basolateral amygdala (BLA), 24 h after conditioning. Secondly, we show that targeted ablation of the GAD65 gene in GAD65−/− mice results in a pronounced context independent, intramodal generalization of fear memory during long-term memory retrieval. The lack of a similar generalization during short-term memory retrieval and failure of GAD65−/− mice to enhance the stimulus-specific response over time suggest a disturbance of fear memory consolidation processes in the mutant mice. An analysis of neural activity patterns in freely behaving mutant mice, finally, revealed a reduction of theta frequency synchronization between LA and area cornu ammonis (CA1) and deficits in γ-frequency oscillations in the LA of GAD65−/− mice. Interestingly, the latter effect was also observed as a result of overtraining in wild type mice, indicating an involvement in both genetically dispositioned and experience-dependent fear. Together our data strongly suggest that GAD65 plays a key role in the consolidation of stimulus-specific fear memory and represents a genetic disposition factor for fear memory generalization.

# Abstract: EFFECT OF RESTRAINT ON THE EXTINCTION OF INHIBITORY AVOIDANCE IN CD1 MICE

**Authors:** A. Valls-Rios, C. I. Navarro-Francés, A. Puigcerver, M. C. Arenas

**Affiliations:** ^1^Department of Psychobiology, University of Valencia, Valencia, Spain; ^2^Department of Psychobiology, University of Malaga, Malaga, Spain

It is well known that stress (acute or chronic) can contribute to the development of anxiety-related disturbances such as phobias and post-traumatic stress disorder (PTSD). Current neurobiological and psychological studies on the pathophysiology of PTSD in humans emphasize the importance of disturbed memory systems, particularly deficits in fear extinction. Thus, the present study was designed to determine the effect of acute stress on the extinction of conditioning with an aversive stimulus. The CD1 male mice were restrained for 2 hours and, 5 days afterwards, were subjected to the training phase of inhibitory avoidance conditioning. After a 90 second adaptation period to the apparatus in the light compartment, the animals received an inescapable footshock of 0.7 mA for 5 seconds when it entered the dark compartment. The test phase was carried out 24 h after training and when the task was finished the animals were placed into the black compartment, but with no shock, to achieve the extinction of conditioning. This phase was repeated another three times. Both stressed and controlmice significantly increased the latencies of crossing in the test phase, showing that the acute stress did not impair the learning of the task. However, control animals extinguished the conditioned avoidance on the fourth test phase, 6 days after the training phase, but the stressed mice did not. This finding indicates that acute stress, such as restraint, impairs the extinction of inhibitory avoidance in male mice. The effect of stress on the extinction of fear memories may constitute a useful animal model to understand the underlying mechanisms of the vulnerability to suffering disorders such as PTSD.

# Abstract: EXPOSURE TO A STRESSFUL EVENT IMPAIRS EXTINCTION IN TWO AVERSIVE PARADIGMS: DIFFERENTIAL INVOLVEMENT OF GLUCOCORTICOID RECEPTORS IN THE AMYGDALA

**Authors:** M. Maroun, I. Akirav

**Affiliations:** ^1^Department of Neurobiology, University of Haifa, Israel; ^2^Department of Psychology, University of Haifa, Haifa, Israel

The ability to extinguish emotional responses in the face of a no-longer relevant conditioned cue is an essential part of a healthy emotional memory system. Despite the efficacy of behavior therapy for anxiety disorders, extinction-like treatments require repeated cue exposures and are vulnerable to reversal by a number of environmental factors, particularly stress. The interaction between stressful experiences and memory has focused mainly on the behavioral and neural mechanisms of memory consolidation, but not on memory extinction. Here we show that exposure to a mild stressor (i.e., placing animals on the elevated platform for 30 min) had a long-term effect of impairing the extinction of contextual fear conditioning and conditioned taste aversion. We further show that microinfusing RU-486, a glucocorticoid receptors (GRs) antagonist, into the basolateral amygdala (BLA) reversed the impairing effects of the stressor in the fear paradigm, but not in the aversion paradigm. Hence, the results suggest that although in both paradigms the stressor increased resistance to extinction, there is a differential involvement of the GRs in the BLA in the fear versus the taste aversion paradigm. The inhibition of fear via extinction receives increasing attention, since it could become an effective intervention for the treatment of fear related disorders. We present first evidence, as far as we know, for the deleterious effects a stressful experience might have on extinction. The effect of stressful experiences is important as it may predispose some individuals to the development of anxiety disorders such as posttraumatic stress disorder or exacerbate its symptomatology.

# Abstract: PATTERNS OF VISUAL SCANNING SHOW GREATER ATTENTION TOWARD THE RIGHT HEMIFIELD THAT IS SPECIFIC TO EMOTIONAL FACES

**Authors:** U. Clark, A. Cronin-Golomb

**Affiliations:** Department of Psychology, Boston University, Boston, MA, USA

Facial expressions, particularly negative emotions, are expressed more intensely by the left than the right hemiface. Though studies have indicated a left visual field (right hemiface) bias in healthy adults (HA) while viewing neutral faces, there has been little examination of such patterns in response to emotional faces and most designs have lacked a non-facial control condition. The present study compared visual scanpaths in response to facial expressions and non-facial images to better inform our understanding of emotion perception in HA. We evaluated the scan pattern of ocular fixations made by 20 non-depressed, cognitively intact HA (age range: 45–72 years) as they identified Facial emotion (2 Happy, 2 Sad) and Landscape (2 Forest, 2 Canyon) images presented for 8 seconds. Facial and Landscape images were judged as equally recognizable by 28 independent observers. Mean fixation duration and number of fixations made to Facial and Landscape images did not differ. Participants fixated longer in the right visual hemifield (RVF) of Facial than of Landscape images. Examining scan patterns by image type showed that more fixations were made in the RVF for Sad than for Happy and Landscape (Canyon) images, and fixation durations were longer in the RVF for Sad than for Landscape (Canyon) images. No correlations with age were found. This study extends previous studies of visual scanpaths in relation to facial expressions and compares these patterns to those elicited by non-facial images. We report a specific RVF bias for facial expressions, especially of negative emotion, compared to non-facial images, reflecting a preference for attending to the more expressive left hemiface. We suggest that enhanced fixation in the RVF increases the amount of information available to the left hemisphere and may help coordinate fine-grained local analyses with holistic processes in the left and right hemispheres respectively.

# Abstract: DEVELOPMENTAL ASPECTS OF RECOGNITION OF SPEECH EMOTIONAL PROSODY VALENCE IN STIMULI OF DIFFERENT TIME STRUCTURE

**Authors:** E. Dmitrieva, V. Gelman, K. Zaitseva, A. Orlov

**Affiliations:** ^1^Inst. of Evol. Physiol. & Biocem. Russ. Ac. Sci.; ^2^Medical Academy for Postgraduate Studies

The theoretical and research contributions have underlined the importance of emotional prosody in speech signals processing for human communication and adjustment to environment. The main aim of the present paper was to further examine the developmental features of cerebral mechanisms of valence evaluation of speech affective component which have not yet received extensive study. The speech material has been obtained by recording of professional actors emotion portrayals. A brief sentence was of neutral semantic content. The simulated emotions were neutral, joy, anger. The neural mechanisms underlying the perception of emotions of different valences were studied by comparing the reaction time (RT) and accuracy of recognition (AR) in 42 adults (20–35, 36–50, 51–65 years old). They were exposed to the stimuli that varied in duration (T) from 0.5 s to 3 s and were presented either on right or left ear of the subject. The results of AR and RT were submitted to analyses of variance. The ANOVA of the data obtained revealed that “age”, “type of emotion”, “stimuli duration” and “gender” factors were highly significant (*p* < 0.0005) in the process of speech emotions recognition in the adult sample and the “emotion valence”-within each age group. The significant factors “type of emotion”*“ear of presentation” interaction (*p* = 0.038) indicated different laterality effects for positive and negative emotional valences recognition. 51–65 years old in the whole showed significantly poorer performance in speech emotional processing as compared to the younger listeners. The positive emotional valence was better recognized in all age groups though in the eldest group the difference was smaller and less significant as compared to the youngest group. *T* = 0.5 s of positive emotional stimuli was shown to be threshold duration for 20–35 years old and *T* = 2 s and longer for 36–65 years old.

# Abstract: WORKING MEMORY PERFORMANCE AFTER ACUTE STRESS IN HEALTHY HUMANS

**Authors:** R. Duncko, B. Cornwell, L. Johnson, K. R. Merikangas, C. Grillon

**Affiliations:** ^1^Section on Developmental Genetic Epidemiology, Intramural Research Program, National Institute of Mental Health, NIH, MD, USA; ^2^Unit of Affective Psychophysiology, Intramural Research Program, National Institute of Mental Health, NIH, MD, USA

Effects of acute stress exposure on learning andmemory have been frequently studied in both animals and humans. However, only a few studies have focused specifically on working memory in humans. We investigated the effect of acute exposure to a Cold Pressor Stress test (CPS; i.e., insertion of the dominant hand into ice water for 60 s) during the Sternberg Working Memory task and the Virtual Morris Water Maze task. The tasks were administered at 20 and 40 minutes after the CPS, respectively. To examine the involvement of sympathetic nervous system and hypothalamicpituitaryadrenocortical (HPA) axis activation during CPS, we measured heart rate, skin conductance and salivary cortisol at selected time points before, during and after the stress procedure. Exposure to the CPS test was associated with a significant increase in heart rate and skin conductance, but no increase in salivary cortisol. Exposure to the stress procedure facilitated learning performance in both learning paradigms. The present results indicate that acute stress can enhance working memory performance in healthy human subjects even in absence of a significant HPA axis activation.

# Abstract: REPEATED EXPOSURE TO A NOVEL ENVIRONMENT; EVALUATING THE ADAPTIVE CAPACITY IN THE BALB/C AND 129/J MOUSE STRAIN

**Authors:** A. R. Salomons, S. S. Arndt, F. Ohl

**Affiliations:** Department of Animal, Science and Society, Faculty of Veterinary Medicine, Utrecht University, The Netherlands

Anxiety refers to a biologically relevant response and is adaptive in its nature. When anxiety responses are in appropriate or prolonged, that is, non-adaptive, they can lead to long lasting (neuro) physiological changes and finally to various forms of pathological anxiety. In animals anxiety might be defined as pathological if it appears to lack adaptive value and severely interferes with normal interaction of the sufferer with its physical and social environment. In this experiment we aimed at evaluating whether the highly anxious phenotype of BALB/c mice represents a pathological, that is, nonadaptive form of anxiety. We investigated 2 inbred mouse strains, the BALB/c and the 129/J male mice and tested them 40 times (4 trials/day) for 5 minutes per trial in the modified hole board test. The results show that the BALB/c mice initially demonstrated significantly more avoidance behaviour as well as risk assessment than 129/J mice. The initially higher anxious BALB/c individuals subsequently habituated to the test situation, and showed increased escaping behaviour. In contrast, the 129/J strain starting at a lower anxious level than the BALB/c mice, showed no habituation in avoidance behaviour throughout the test period. Habituation in BALB/c mice was also seen in other parameters such as exploratory activity and locomotion whereas no apparent changes in these parameters were observed in the 129/J strain during the test period. Notably, stress hormone (corticosterone) responses to the initial test situation revealed higher corticosterone levels in BALB/c mice compared to 129/J mice. This underlines that the BALB/c strain habituates to an initially stressful novel environment while the 129/J strain probably lacks adaptive capacity under the same circumstances. Further investigations are necessary to show whether 129/J mice are either insensitive for the aversive characteristics of a novel environment or might be a model for impaired habituation.

# Abstract: THE COMPARTMENTAL, HORMONAL AND CARDIOVASCULAR RESPONSES TO NOISE STRESS IN THE WISTAR MALE RAT

**Authors:** H. Beldjoud, H. Zerroug, B. Asselah, A. Abdelmalek

**Affiliations:** Laboratoire de Neurosciences comportementales et cognitives, FSB, Université des Sciences et de la Technologie, Houari Boumediene, BP 32, El-Alia, Alger, Algérie

The goal of our researchs is to evaluate the effects of an auditory stress (noise of 100 dB) short-term (CT) and mediumterm (MT), among the Wistar male rat, on a set of behaviour, the plasmatic ACTH and on the CV parameters (MAP, FC). A noise of 15 min causes some variations of behaviour with reduction of the exploring (*p* < 0, 05, Student test). This variation becomes very important (*p* < 0, 001,) after cessation of the noise, whereas the number of freezing increases very meaningful way (*p* < 0, 01) and continuous of the being (*p* < 0, 05). The sleeping/Resting, the rearing as well as the grooming don't present any variation of their basal values. The sniffing that presented no important variation during the 15 min of noise, show a meaningful reduction after cessation of the noise (*p* < 0, 05). The absolute value of the ACTH increases meaningful way among the rats having undergone the noise during 15 min of noise in relation to the rats witnesses non submitted to the noise (*p* < 0, 05). The PAM as well as the FC didn't undergo any meaningful variations When the noise is managed every day (15 min/Day, 7 days), the exploring remains decreased in a meaningful way at the time of the induction of the noise and after end of this one. In the same way, the continuous Sniffing to be reduced after the noise (*p* < 0, 05). The freezing as for him it doesn't present any variation. The ACTH of the rats submitted to the noise during 7 days doesn't present meaningful variation compared to the non stressed rats anymore. This absence of answer would be associated to the adaptation likely of the HPA axis to the repeated homotypic stress. The CV parameters didn't undergo any variation meaningful. The variations caused by the noise (15 min) don't meet when this one is managed during 7 days let suppose that he got settled a phenomenon of habituation to the managed noise which doesn't seem to represent a danger as well as an adaptability of the homeostatic systemto the auditory stress.

# Abstract: THE INTERPLAY BETWEEN JUVENILE STRESS AND ENRICHED ENVIRONMENT ON BEHAVIOR AND L1-CAM

**Authors:** Y. Ilin, G. Richter-Levin

**Affiliations:** The Brain and Behavior Research Center, University of Haifa, Haifa, Israel

Epidemiological studies indicate that childhood trauma is predominantly associated with higher rates of both mood and anxiety disorders, which were associated with altered limbic system functioning. Exposure of rats to stress during juvenility (27–29 days of age) has comparable effects and was suggested as a model of induced predisposition for these disorders (Avital and Richter-Levin, 2005; Tsoory et al., 2006). The importance of the environment in the regulation of brain, behavior and physiology has long been recognized in biological, social and medical sciences. Animals maintained under enriched conditions have clearly been shown to have better learning abilities than those maintained under standard conditions. We set out to investigate the long-term effects of Juvenile stress and effects of enriched environment (EE) on the ability of animals to cope with subsequent learning tasks and on the expression of the cell adhesion molecule L1, suggested to be involved in environment-induced neuronal re-organization. Three groups were tested: 1) Juvenile Stress subjected to Juvenile stress; 2) Enriched Environmentsubjected to Juvenile stress and then, from day 30 on to EE; and 3) Naïves. In adulthood, coping and stress responses were examined using the elevated plusmaze, open field, exploration and avoidance learning. In adulthood, “juvenile” stressed rats exhibited reduced exploration in a novel setting, and poor avoidance learning, with learned helplessness-like behaviors, while exposure to EE impede these long lasting effects of juvenile stress. Preliminary findings suggest that these where associated also with lasting alterations in the expression of L1.

## Figures and Tables

**Figure 1:** DLPFC activation during the memory suppression and retrieval phases in patients (blue) and healthy controls (red)

# Abstract: MECHANISM OF MEMORY SUPPRESSION IN STRESS-RELATED PSYCHIATRIC DISORDERS: PRELIMINARY RESULTS

**Authors:** M. Baiano, M. Balestrieri, M. Maieron, L. Perini, R. Zuliani, C. Rossana, C. Giuseppe, C. Zuiani, M. Bazzocchi, B. Francesco, S. Michela, P. Brambilla

**Affiliations:** ^1^University of Udine, Udine, Italy; ^2^DPMSC, Section of Psychiatry; ^3^DSTB, Section of Physiology, Udine, Italy; ^4^DRMM, Section of Radiology, Pavia, Italy; ^2^University of Pavia, Section of Psychiatry, Pavia, Italy

Recurrent or chronic stressful events, such as early dysfunctional relationships, sexual/physical abuse, or mobbing may play a key role in the pathophysiology of stress-related disorders, such as Major Depression (MD) or Borderline Personality Disorder (BPD). Structural and biochemistry abnormalities of dorsolateral prefrontal cortex (DLPFC) and hippocampus were shown both in MD and BPD patients undergoing MRI investigations [1]. Interestingly, it has been demonstrated that the DLPFC-hippocampus circuitry is crucial for memory suppression in humans [2]. In this study we aim to explore with functional MRI whether the suppression mechanism is impaired in patients suffering from MD and BPD.

**Methods**: Four DSM-IV patients withMD, four DSM-IV patients with BPD and 11 healthy controls underwent a 1.5T MRI session by performing a specific go/no-go test [3]. A qualitative cluster analysis, based on Tailarach parameters, has been performed by visually detecting the activation of DLPFC and hippocampus.

**Preliminary Results**: Healthy control subjects showed a greater DLPFC activation during memory suppression than retrieval phase compared to patients Figure 1.

**Conclusions**: These preliminary data suggest the presence of dysfunctional DLPFC-hippocampus circuitry during memory suppression in patients with either MD or BPD. This may be due to impaired suppression of hippocampus by the DLPFC. Hypothetically, alterations of such neuronal network may impair the mechanism of repression of unwanted memories in MD and BPD, playing a major role for the psychopathology of these disorders.

## References

[1] Sala M, Perez J, Soloff P (2004). Stress and hippocampal abnormalities in psychiatric disorders. *European Neuropsychopharmacology*.

[2] Anderson MC, Ochsner KN, Kuhl B (2004). Neural systems underlying the suppression of unwanted memories. *Science*.

[3] Anderson MC, Green C (2001). Suppressing unwanted memories by executive control. *Nature*.

# Abstract: THE EFFECTS OF SEX AND STRESS ON AVIAN COGNITION

**Authors:** H. Olivia, M. Simone, C. Nicola, H. Sue

**Affiliations:** ^1^Edinburgh University, UK; ^2^Cambridge University, UK

Recent research findings indicate that the sex of an individual may influence behaviour, memory and the brain's response to stress hormones. Females have a much greater incidence of some mental disorders, especially those that are stressrelated such as generalized anxiety. Despite the above, female animals are rarely tested in experiments and little is known about the interplay between sex and stress hormones in cognition. My overall aim is to investigate this relationship in the zebra finch, using a spatial and non-spatial version of a one-trial associative memory task. Birds will be trained and tested to find food hidden under flaps, using either the location or colour of the flap. Initially the role of sex steroids is being determined, and performance on this task following artificially increased and decreased hormone levels is being examined. Pharmacological manipulations will be used to determine if testosterone itself or its oestrogen metabolites are producing effects. The neural basis of those effects will be investigated through examination of hippocampal slices. Performance on this task will then be compared with that following ingestion of the stress hormone corticosterone, injected into meal worms. Differential effects of sex and stress raise the possibility that it might be necessary to develop sex specific treatments for conditions such as depression, addiction, schizophrenia, and post-traumatic stress disorder.

# Abstract: A COMPARATIVE STUDY OF THE EFFECTS OF INJECTIONS OF SUBSTANCE P INTO THE DORSAL AND VENTRAL HIPPOCAMPUS ON THE EXTRACELLULAR CONCENTRATION OF SEROTONIN AND THE EXPLORATORY BEHAVIOR OF RATS

**Authors:** M. C. Carvalho, S. Masson, M. A. De Souza Silva, M. L. Brandão

**Affiliations:** ^1^Laboratory of Psychobiology, FFCLRP-USP, São Paulo, Brazil; ^2^Institute of Physiological Psychology, University of Düsseldorf, Düsseldorf, Germany

Substance P (SP) is found in brain regions associated with fear/anxiety reactions such as the amygdala, hypothalamus, periaqueductal gray and hippocampus. The dorsal (DH) and ventral (VH) hippocampus has been related with cognitive and emotional processes. Considering that the hippocampus also receives serotoninergic terminals, and both SP/ 5- HT mechanisms have been implicated in the expression of fear/anxiety-like processes, the aim of the present study was to investigate the effects of administration of SP (10–1000 ng/ 0.5 *μ*L) on the serotoninergic activity of the DH and VH through microdialysis technique. Besides, it was investigated the role of SP-VH on exploratory behavior of rats submitted to the elevated plus maze (EPM). The results showed that only SP-100 ng increased the extracellular level of serotonin in the VH. Behaviorally, SP injections into the DH, but not in the VH, caused significant anxiolytic effects on the exploratory behavior of rats in the EPM. Therefore, it appears that the observed interaction of SP and 5-HT mechanisms in the VH is not implicated in the expression of fear responses to the height and openness of the EPM. On the other hand, these aversive states may be mediated by SP-mediated mechanisms in the DH. FAPESP.

# Abstract: EFFECTS OF DIFFERENT STRESSORS ON DEPRESSION IN FEMALE RATS: MODIFICATIONS BY OVARIECTOMY AND ESTRADIOL TREATMENT

**Authors:** J. Fedotova, S. Barabanova

**Affiliations:** Department of Neuropharmacology, Institute Experimental Medicine, Russian Academy of Medical Sciences, 12 Acad. Pavlov Street, 197376 St. Petersburg, Russia

It is widely accepted that stressmay be involved in the clinical manifestation of depression (Stout, Nemeroff, 1994; Mazure, 1995). However, it is not known whether and in which extent stressors of different strengthmight influence the response of female animals with imbalance of estrogen in experimental models of depression.

The aim of present work was to evaluate the mild and severe stressors effects on behavioral despair in forced swim test using intact cycling female rats during the estrous cycle, ovariectomized (OVX) rats and OVX-estrogen treated female rats.

The intact cycling, OVX and OVX-estrogen treated females were received electric shocks of different intensity and duration (mild, moderate and severe) 24 h and 1 h before being subjected to forced swim test. Statistical processing of the data was carried out using two-way ANOVA test and posthoc Dunnett's test for multiple comparisons at *p* < 0.05.

In the forced swim test, immobility time appeared to be generally higher when mild (0.1 mA, 5 ms), moderate (1 mA, 5 ms) or severe shocks were applied prior to behavioral testing in proestrus and estrus animals, while the behavioral response of diestrus and metestrus animals did not differ from that of nonshocked female rats. Application of severe shock significantly decreased immobility time in OVX rats as compared to non-shocked OVX rats. On the contrary, application of mild shock had no effect on immobility time in OVX rats as compared to non-shocked OVX rats. Application of mild shock significantly reduced immobility time in E2-treated OVX rats as compared to E2-treated non-shocked OVX rats. Stress-induced plasma corticosterone levels surge correlated with intensity and duration of shocks in female rats.

Thus, these results suggest that duration and intensity of stressors profoundly affect the behavioral response of female rats with imbalance of estrogen in forced swim test.

# Abstract: A CHRONIC BY GAVAGE APPLICATION PROCEDURE PER SE INDUCES A DEPRESSION-LIKE PHENOTYPE IN MICE

**Authors:** M. S. H. Henniger, M. C. Schweizer, C. Storch, I. Sillaber

**Affiliations:** Affectis Pharmaceuticals, Munich, Germany

Chronic oral drug application by gavage is a common procedure in many animal experiments. Objective of the present study was to assess the behavioural impact of this chronic treatment scheme per se in paradigms of anxiety-like behaviour, stress coping and hedonic behaviour.

Male DBA/2JIco mice were assigned to two groups: One group (gavage-treatment group) was given tap water by gavage for a total period of 7 weeks. The control group was left undisturbed in the home cage. After 6 weeks of gavagetreatment, allmice were subsequently tested in the open field, dark/light box and forced swim test paradigms. In the latter two paradigms part of the mice was acutely given paroxetine before the test. In an independent batch of male DBA/2Ola mice, the influence of 6 weeks gavage-treatment on voluntary saccharin intake in a two bottle-free choice restricted access paradigm was tested.

Compared to control, mice of the gavage-treatment group were significantly less active in the open field and displayed significantly more anxiety-like behaviour in the dark/light box. The latter was reversed by acute paroxetine application. Behaviour in the forced swim test was not changed by the chronic treatment procedure, however, the antidepressant properties of paroxetine were more pronounced in mice that experienced chronic gavage-treatment than in the control group. In saccharine consumption, a significant and stable decrease was seen after two weeks of the water gavaging procedure.

In conclusion, a chronic enforced oral application procedure induces a phenotype that resembles several features of depression, i.e., psychomotor retardation, anxiety symptoms and anhedonia. Thus, the chronic application procedure might per se act as a chronic mild stress model and hence facilitate the screening of novel antidepressants.

# Abstract: ROLE OF THE AMYGDALA ON ACUTE AND CHRONIC ANTIDEPRESSANT ACTIONS IN BEHAVIOR AND HIPPOCAMPAL NEUROGENESIS

**Authors:** J. Castro, E. Varea, M. I. Cordero, C. Sandi

**Affiliations:** Laboratory of Behavioral Genetics, Brain Mind Institute, Swiss Federal Institute of Technology (EPFL), Lausanne, Switzerland

Depression has been associated with a decrease in hippocampal neurogenesis, pointing to a role of the lack of new cells and new connections in the cognitive and emotional symptoms of mood disorders, and to neurogenesis as a possible target/mechanism for antidepressants actions. Selective serotonin reuptake inhibitors (SSRI), although induce an immediate increase in the extracellular levels of serotonin and noradrenalin, need 3–4 weeks to develop both therapeutic effects and increased neurogenesis. This evidence highlights the existence of other relevant mediating mechanisms in the course of depression and antidepressant actions. The amygdala has been related to the both the etiology and the recovery of depression which has also been linked to increased expression of corticotrophin releasing factor (CRF). Here, we hypothesized that the amygdala might play a key role in both therapeutic effects and neurogenic action of antidepressants. In order to test this hypothesis, amygdala or sham lesions were conducted in Sprague-Dawley rats and after recovery, animals were behaviorally characterized in a variety of tasks selected to evaluate anxiety- and depression-like behaviors, social interactions, and memory function. They were then submitted either to acute or chronic treatment with the SSRI fluoxetine and subsequently tested for a variety of behaviors. The results show an interaction between amygdala lesions and fluoxetine treatments over both behavioral outcomes and hippocampal neurogenesis. Particularly, chronic fluoxetine treatment enhanced neurogenesis and reduced anxietyand depression-like behaviors whereas acute treatment reduced neurogenesis and increased anxiety-like behaviors; interestingly, in both cases acute and chronic fluoxetine treatment, amygdala lesions suppressed these neurogenic and behavioral effects.

# Abstract: WHAT'S SO FUNNY—RAT 50-KHZ VOCALIZATIONS AS AN ANCESTRAL FORM OF HUMAN LAUGHTER?

**Authors:** R. K. W. Schwarting, C. Natusch, M. Wöhr

**Affiliations:** Experimental and Physiological Psychology, Philipps-University of Marburg, Gutenbergstr. 18, 35032 Marburg, Germany

Panksepp and coworkers have provided a wealth of experimental evidence showing that rats, if tickled in a playful way, emit 50-kHz chirps. Even more, these rats became socially bonded to the tickling experimenter, could be conditioned to seek tickles, and preferred to spend more time with other animals that chirped a lot rather than with those that did not. Based on such evidence, Panksepp suggested that “rat laughter” might serve as an index of the animal's subjective state, and that it might provide a new measure for analyzing natural reward/desire circuits in the brain [1].

We have recently shown that 50-kHz calls cannot only be induced by tickling rats, but also by shortly isolating them from their mates in a housing cage [2]. Calling under such conditions did not habituate with repeated testing, and occurred in the animal's own, or in a fresh housing cage. Furthermore, the propensity to call differed substantially and reliably between individual rats. We assume that such calls are social signals (for example, to reestablish contact), which are more likely if the animal is in a positive emotional and appetitive state. New evidence in favor of this hypothesis will be presented.

## References

[1] Panksepp J (2005). Psychology: beyond a joke: from animal laughter to human joy?. *Science*.

[2] Schwarting RKW, Jegan N, Wöhr M (2007). Situational factors, conditions and individual variables which can determine ultrasonic
vocalizations in male adult Wistar rats. *Behavioural Brain Research*.

# Abstract: THE EFFECT OF RAT TURNING SPEED UPON RAT TURNING BEHAVIOUR AND HIPPOCAMPAL NORADRENALIN AND CORTICOSTERONE LEVELS

**Authors:** A. Van Hemelrijck, C. Nolten, A. Shand-Davies, T. Steckler, W. H. I. M. Drinkenburg

**Affiliations:** Johnson & Johnson Pharmaceutical Research & Development, a Division of Janssen Pharmaceutica N.V. Turnhoutseweg 30, 2340 Beerse, Belgium

Measuring neurotransmitter levels in the brain extracellular fluid using microdialysis on freely moving, conscious rats can be used to directly correlate stressors with neuronal response. Catecholamines and noradrenalin release in particular is involved in a wide array of neurological pathways and is widely accepted as having a direct correlation to stressful stimuli as part of the hypothalamus-pituitary axis and the locus coeruleus-norepinephrine systems. The aim of this study is to determine the effect of rat turning and turning speed upon rat turning behaviour and hippocampal levels of noradrenaline and corticosterone, in order to minimize stress, limitation of movement and other confounding factors in microdialysis studies. Three groups of rat setups were examined: fast to slow turning, slow to fast turning and a liquid swivel. Rat turning behaviour was recorded digitally over a period of forty-five hours. Brain samples were taken twice for two hours in twenty-four hours and analysed for corticosterone and noradrenalin with radio immuno assay and liquid chromatography coupled to electrochemical detection respectively. The results show a significant difference in total turning time between the first day/night and the second day/night in both turning setups, meaning that it takes et least twelve hours before the rats are adapted to the turning apparatus. These results are confirmed by a decrease of hippocampal corticosterone and noradrenalin levels during the second day compared to the first day in both turning setups. The slow to fast turning setup showed enhanced slow movement and therefore a higher total turning compared to the fast to slow turning setup. Therefore, these results reveal that the adaptation period to the turning apparatus and the turning speed in microdialysis studies require careful consideration when interpreting data.

# Abstract: ACTIVITY CHANGES IN MICE SUBJECTED TO CHRONIC MILD STRESS: A PIVOTAL POINT FOR STRESS RESPONSE AND MEASURES REFLECTING ANHEDONIA?

**Authors:** M. C. Schweizer, M. S. H. Henniger, F. Schleicher, T. Pohl, M. B. Müller, I. Sillaber

**Affiliations:** ^1^Affectis Pharmaceuticals AG, Munich, Germany; ^2^Max-Planck-Institute of Psychiatry, Munich, Germany

Chronic mild stress (CMS) as an animal model of depression enjoys a certain popularity in psychiatric research - not least due to its face validity and comprehensive readout. However, the results are difficult to replicate in different labs. The goal of the present study was to examine the influence of stressinduced changes in general activity on the behavioural readout. We particularly focussed on the influence of light as a widely used stressor in CMS protocols on subsequent measures. A weekly CMS schedule consisting of common mild stressors was applied for at least 4 weeks to different strains of mice. During this stress period saccharin intake and preference over water was acquired twice a week, each time during the first 2 hours of the dark phase. To exclude interventions with a putatively high impact on consuming behaviour per se, food and water deprivation was omitted. Parameters were assessed using tests like open field, modified holeboard and long-termhome cage observation. Independent of the illumination conditions in the behavioural tests, an apparently paradox decrease in anxiety-related behaviour after CMS was observed. This could be explained by a generally increased stress-induced activity, which in turn appeared as reduced risk assessment behaviour. Preceding application of a single footshock normalised the latter in CMS mice. While no enduring decrease in saccharin intake (‘anhedonia’) due to CMS was observed, over-night illumination as particular stressor of the weekly paradigm turned out to be associated with a significant decrease in saccharin intake during the measurement period the day after. Taken together, these results suggest that in mice the CMS regimen as a whole causes hyperreactivity to a novel environment represented by the test situation and therefore requires careful interpretation of behaviour. Shifts in circadian rhythms due to light cycle changes may mimic an ‘anhedonic’ effect of CMS.

# Abstract: NON-CONSCIOUS EMOTIONAL CONTAGION IN BLINDSIGHT

**Authors:** M. Tamietto, L. Castelli, S. Vighetti, L. Latini Corazzini, G. Geminiani, L. Weiskrantz, B. de Gelder

**Affiliations:** ^1^Depatment of Psychology, University of Torino, Italy; ^2^University of Oxford, UK; ^3^University of Tilburg and MGH Harvard Medical School, The Netherlands

Observing facial expressions prompts imitation as can be typically observed with facial electromyography (EMG). Here we explored whether this automatic reaction occurs even in the absence of visual awareness for the stimulus, and whether this can be elicited also by bodily expressions. Facial and bodily expressions of happiness and fear were presented either in the intact visual field or in the blind field of two well-known hemianope patients (DB and GY) with striate cortex lesions but residual vision (blindsight). The patients were required to judge the emotional expression of the pictures presented in their intact visual field, and “to guess” the expression of the unseen pictures shown in their blind field. During the task we recorded emotion-specific facial muscle activity (zygomaticus major for happy, corrugator supercilii for fear). Despite both patients reported no visual awareness for stimuli projected in the blind field and commented their performance as “at chance”, their evaluation of the emotional expressions was significantly above chance-level for faces and bodies alike. Most notably, unseen facial as well as bodily expressions produced a congruent emotional reaction in patients' face, comparable to that observed in response to consciously perceived pictures. Our findings provide evidence that facial expressions in the observer may unfold as an automatic reaction that results from emotional contagion. This expressive response seems insensitive to visual awareness and to the specific perceptual features of the stimuli. Rather, it appears to be modulated by the emotional valence of external events.

# Abstract: SUBDIVISIONS OF THE ARCOPALLIUM ARE DIFFERENTIALLY INVOLVED IN THE CONTROL OF FEAR BEHAVIOUR IN THE JAPANESE QUAIL

**Authors:** H. Saint-Dizier, C. Leterrier, F. Lévy, S. Richard

**Affiliations:** Equipe Comportement, Neurobiologie et Adaptation, Unitè de Physiologie de la Reproduction et des Comportements, UMR85 INRA – CNRS – Université de Tours Haras Nationaux, F-37380 Nouzilly, France

Growing interest in the phylogeny of emotions within vertebrates has motivated research on the neurobiology of fear reactions in birds. In the avian brain, the arcopallium has been suspected to play a major role in the control of fear reactions. This structure is considered as a partial homologue of the mammalian amygdale, on the basis of developmental and anatomical data. Moreover, lesions or stimulations of the arcopallium induce respectively a decrease or an increase in fear reactions. However, the arcopallium is a large and heterogeneous structure and the specific roles of its subdivisions are unknown. The present study aimed at investigating the respective implications of different subdivisions of the arcopallium in the control of fear behaviour. Adult Japanese quail were given bilateral electrolytic lesions of the arcopallium or sham-operation, and were subsequently placed in several tests of fear: open-field, hole-in the-wall box, tonic immobility and novel object tests. Quail with lesions of the anterior part of the arcopallium exhibited reduced fear behaviour when compared to sham-operated quail. By contrast, quail with lesions in the caudal part of the arcopallium tended to show more pronounced fear behaviour than sham-operated quail. The behaviour of quail with combined lesions of the anterior and caudal parts of the arcopallium was not significantly different from that of shamoperated quail. Those results are the first to show a differential involvement of subdivisions of the arcopallium in the control of fear behaviour in birds. The results will be discussed in the light of current knowledge regarding the neuroanatomical characteristics of the arcopallium.

# Abstract: EFFECTS OF HOUSING ENVIRONMENT ON ACTIVITY AND ANXIETY LEVEL IN MALE AND FEMALE MICE

**Authors:** A. Puigcerver, A. Valls-Rios, C. I. Navarro-Frances, M. C. Arenas

**Affiliations:** ^1^Department of Psychobiology, University of Valencia, Valencia, Spain; ^2^Department of Psychobiology, University of Malaga, Malaga, Spain

The present study was designed to determine the effect of individual or social housing on various tests of anxiety in mice of both sexes. After an 18-day isolation period or grouphousing, general activity of each mouse in the actimeter and in the open-field test was recorded for 5 min. Afterwards, the animals were individually placed onto the central square of the elevated plus-maze and video recorded for 5 min. The number of counts in the actimeter and number of crossings from one square to another in the openfield were registered as measures of activity, and also the number of closed arm entries in the elevated plus-maze. The percentage of time on the central square of the openfield, together with the percentage of time spent on the open arms and the percentage of open arm entries were scored as measures of anxiety level. Individual housing increased the activity on both open-field and actimeter, this increase being really due to the effect of isolation on the females, because when sexes were analysed separately, individually housed females showed higher activity than those socially housed, however, housing conditions had no effect on males. Furthermore, isolated females displayed higher activity in the actimeter than isolated males. Individual housing also increased the general activity ofmice on the elevated plus-maze, but equally in both sexes. The females displayed more anxiety than males, spending less time on the central square of the open-field. This effect was not due to sex differences in general locomotion, because it was precisely the females which presented more activity in this behavioural test. Nevertheless, neither the individual housing condition nor the sex produced significant differences in anxiety on the plus-maze. These results indicate that social housing can reduce the hyperactive response to novelty in females, whereas in males, the housing environment did not have a significant effect on activity or performance in the anxiety test.

# Abstract: WHAT CAN NAPLES HIGH EXCITABILITY AND SPONTANEOUSLY HYPERTENSIVE RATS TELL US ABOUT DIFFERENT VARIANTS OF ATTENTION-DEFICIT HYPERACTIVITY DISORDER?

**Authors:** L. A. Ruocco, D. Vallone, D. Viggiano, U. A. Gironi Carnevale, A. G. Sadile

**Affiliations:** ^1^Lab Neurophysiol. Behavior & Neural Networks, Department Exptl. Med, Second Univ. Naples, Naples, Italy; ^2^Institut für Toxikologie und Genetik, Forschungszentrum Karlsruhe, Germany; ^3^Department Neurosci., University of Naples “Federico II”, Naples, Italy

Attention Deficit Hyperactivity Disorder (ADHD) is a neurodevelopmental problem affecting 1–3%of school children, mainly boys (4 : 1 ratio). It is characterized by inattention, hyperactivity and impulsivity. An altered mesocorticolimbic dopamine (DA) system is thought to be associated to different variants of ADHD. The Naples High Excitability (NHE) and the Spontaneously Hypertensive (SHR) rats model the variant with altered executive functions and response inhibition respectively. The NHE show hyperactive mesocortical DA branch by hypertrophic DA neurons, high expression of tyrosine hidroxylase (TH), high DA D2 receptor density and overexpression of DA-related phosphoprotein (DARP32) in the mesencephalon. Conversely they show in the prefrontal cortex (PFC) more axonal varicosities, high DA transporter (DAT) density and lower DA D1 and D2 receptors. Moreover the mesotriatal branch is not altered as shown by TH, DAT, DA D1 and D2 receptors and DARP32. Treatment with methylphenidate (MPH; 3 mg/Kg i.p. for 14 days) reverses the basal profile. In contrast, the SHR show altered mesolimbic and mesocortical branches associated with no main changes in the mesencephalon but with a high responsiveness for TH expression and no responsiveness for DA D2 autoreceptors to MPH treatment. Conversely in the PFC a higher basal DA tone (Carboni et al. 2003, 2004) is associated with high DAT density (Watanabe et al. 1997) and higher DA D1 and D2 receptors (Carey et al., 1998). In addition in the striatum DA D1 and D2 receptors show high density due to altered pruning that is reversed by MPH treatment. Moreover DA reuptake system is defective as shown in synaptosome preparations (Leo et al., 2003). Thus, both high and low functional state of mesocorticolimbic DA branches may be associated with impaired response inhibition, attention and executive functions.

# Abstract: DO HOUSING CONDITIONS AFFECT THE BEHAVIOUR OF RESTRAINT-STRESSED MALE AND FEMALE MICE ON THE ELEVATED PLUS-MAZE?

**Authors:** C. I. Navarro-Frances, A. Valls-Rios, A. Puigcerver, M. C. Arenas

**Affiliations:** ^1^Department of Psychobiology, University of Valencia, Valencia, Spain; ^2^Department of Psychobiology, University of Malaga, Malaga, Spain

In humans, social deprivation is linked to an increase in the vulnerability to develop severe mental illnesses such as depression and anxiety disorders. Previous clinical studies have associated the development of these psychological disturbances with stressful situations. Thus, the present study was designed to determine the impact of medium stress (restraint) on activity and anxiety levels of male and female CD1 mice which were previously housed in different conditions, isolated or social, for 18 days. After this period, while the control animals continued in their home cages, the experimental mice were immobilized for 2 hours. Afterwards, the animals were tested in the elevated plus-maze for 5 min. The number of entries into open and closed arms, as a measure of activity, the percentage of time spent in the open arms and the percentage of open arm entries, as measures of anxiety, were scored by a trained observer, blind with respect to the housing and treatment conditions. The activity was higher in individually than socially housed mice, and the restraint produced a decrease in the locomotion of all animals. However, when the effect of stress on mice of the two housing conditions was analysed separately, this reduction of activity was only observed in socially housed subjects. Similarly, socially housed mice presented a higher level of anxiety than those individually housed, but restraint only increased anxiety in socially housed mice, females showing more anxiety than males. In conclusion, individual housing annulled the effects of acute stress on animal behaviour and sex differences observed. This research may help to understand the biological basis of sex differences in the vulnerability to stress diseases and their interaction with social isolation.

# Abstract: EMOTIONAL MEMORY AND MIGRAINE

**Authors:** A. Gasbarri, B. Arnone, A. Pompili, P. Di Fabrizio, C. Marini, A. Cifariello, C. M. Tavares, C. Tomaz

**Affiliations:** ^1^Department of Biomedical Sciences and Technologies, University of L'Aquila, Italy; ^2^Department of Internal Medicine, University of L'Aquila, Italy; ^3^Laboratory of Neurosciences and Behavior, University of Brasilia, CEP 70910-900 Brasilia, DF, Brazil

It is very well known that emotional events are better memorized than neutral events. In the present study we evaluated the effects of emotional content on explicit memory in cephalalgic patients, suffering from migraine headache. We utilized an adaptation of two versions of the same story, with different arousing properties (neutral or emotional), which have been already employed in several studies focused on the enhancing effects of emotions on memory retention. Subjects of this study were healthy subjects and cephalalgic patients, which included untreated migraineurs and migraineurs treated with the antidepressant amitriptyline. Our findings suggested that chronic migraine is related to memory impairment. Taking into account that migraine is associated with major depression, in the present research the effect of the antidepressant amitriptyline was also evaluated. Our results showed that amitriptyline has an impairment effect on memory. In fact, the untreated migraineurs recalled the most emotional phase of the arousal story significantly better compared to migraineurs treated with amitriptyline. Then, our data suggest that amitriptyline prevents the enhancing effects of emotional content on memory processes. Moreover, in agreement with the results we reported in previous papers, the present study confirms the existence of gender differences in the processing of emotional stimuli and underscores the view that the gender influences should be considered in future studies on neural correlates of emotion, and on the relation of emotion to memory.

# Abstract: TRANSGENERATIONAL TRANSMISSION OF ENVIRONMENTALLY-INDUCED BEHAVIORAL TRAITS

**Authors:** T. B. Franklin, H. Russig, J. Graeff, I. M. Mansuy

**Affiliations:** Brain Research Institute, University of Zurich; Swiss Federal Institute of Technology (EPEL), Lausanne, Switzerland

Early life trauma, such as chronic exposure to physical or mental stress, is a contributing factor to the development of behavioral and emotional disorders. These disorders not only persist throughout life, but are often heritable. To investigate their potential mechanisms of occurrence and transmission, we modeled early trauma in the mouse using chronic and unpredictablematernal separation, and examined the impact on adult behavior in two consecutive generations. Mice subjected to maternal separation behaved abnormally when exposed to novel or stressful conditions in adulthood and displayed depressive-like behaviors. Strikingly, their offspring exhibited similar impairments, despite being reared in normal conditions, suggesting that behavioural impairments induced by early trauma can be transmitted through epigenetic mechanisms. Preliminary evidence indicates that alteration in DNA methylation of genes associated with stress pathways may be involved. This mouse model provides a unique means to investigate the factors involved in the epidemiology of stress-related affective disorders.

# Abstract: THE EFFECTS OF ADULT AND MATERNAL CALORIE RESTRICTION ON SOCIAL, SEXUAL AND FEAR BEHAVIOUR IN RATS

**Authors:** A. Govic, E. A. Levay, J. Penman, S. Kent, A. G. Paolini

**Affiliations:** School of Psychological Science, La Trobe University, Victoria, VI, Australia

Despite an abundance of research on calorie restriction (CR) and health, the consequences of CR on social and sexual behaviour remain to be examined systematically. This study compared the social behaviour, social recognition memory (SRM), sexual behaviour, and partner preference for male adult rats administered a 3 week CR (either 25%, 50%, or an acute episode) with ad libitum fed controls. The CR25% and CR50% both demonstrated greater social interaction than controls. Although the CR25% group failed to exhibit SRM, their pattern of behaviour appeared to reflect anxiety more than a deficit in memory. Greater partner preference was observed for the control animals only when compared to the CR50% group. Sexual performance was altered in the CR50% group, which demonstrated fewer mounts, intromissions, and a longer latency to the first intromission than controls. Serum testosterone was also reduced in both CR groups compared to controls. The social and sexual behaviour of male offspring from dams that were administered 25% CR at one of four times in the perinatal period: a brief period preconception, during gestation, during lactation, or lifelong (gestation, lactation, & continuing postweaning) was also investigated. Social interaction was increased by a lifelong CR, as was the exhibition of more dominant type behaviours. Typically, CR during preconception or lactation resulted in offspring that displayed an enhanced and more efficient copulatory pattern compared controls. Furthermore, serum testosterone was significantly higher in the preconception and lifelong groups than controls. The fear responses to auditory fear conditioning were also assessed in these animals. The lactation group was found to spend more time freezing during extinction and maintained the fear response longer than the other groups. These findings indicate that CR, initiated in adulthood or in specific periods of development, can alter the behavioural phenotype of animals.

# Abstract: TRANSGENIC MICE OVEREXPRESSING THE NEUROTROPHIN RECEPTOR, TRKC SHOW HYPOTHALAMIC-PITUITARY-ADRENAL (HPA) AXIS DYSREGULATION AFTER DIFFERENT STRESS CONDITIONS

**Authors:** A. Amador-Arjona, X. Gallego, X. Belda, R. Delgado, M. E. Keck, A. Armario, M. Dierssen

**Affiliations:** ^1^Genes and Disease Program, Genomic Regulation Center, Barcelona, Spain; ^2^Institute of Neuroscience and Department Cellular Biology, Physiology and Immunology, Autonomous University of Barcelona, Bellaterra, Barcelona, Spain; ^3^Max Planck Institute of Psychiatry, Munich, Germany

We have used transgenic mice overexpressing TrkC, the high-affinity receptor of NT-3, (TgNTRK3), that has been validated as a mouse model for anxiety/panic disorder, to study the neuroendocrine and behavioral consequences of stress. We have used differential lightening conditions, novel environment, restraint, and morphine abstinence as stressors with physical and/or psychological components. The analysis of corticosterone levels revealed no apparent alterations in hypothalamus-pituitaryadrenal (HPA) axis in TgNTRK3 under basal conditions or after exposure to a mild stressor, such as the open field. However, TgNTRK3 mice showed a higher increase in corticosterone levels after a more intense stressful stimulus, as is restraint in tube. Restraint also caused a lower increase of CRH mRNA in TgNTRK3 paraventricular nucleus of the hypothalamus, an effect that may be explained due to an enhanced negative-feedback response in CRH synthesis by a higher increase in corticosterone secretion. Anxiety-like behavior was tested after chronically increased levels of diurnal illumination intensity. In the lightdark box, stressed TgNTRK3 mice showed a tendency to spend less time in the light compartment and a similar activityexploratory behavior than the non-stressed TgNTRK3 group. On the other hand, immobilization in a wooden board in combination with social isolation showed a different HPA axis response in TgNTRK3 mice. Finally, after morphine withdrawal TgNTRK3 showed a higher increase of corticosterone levels as compared to wild types. These results may reflect different stress responses and coping strategies in TgNTRK3 mice that suggest a role of this neurotrophin receptor in the habituation process to chronic stress situations and in theHPA axis response after drug dependence.

# Abstract: SHORT- AND LONG-TERM CONSEQUENCES OF EXPOSING RATS TO STRESSORS DURING JUVENILITY

**Authors:** S. Jacobson-Pick, K. Rosenblum, G. Richter-Levin

**Affiliations:** The Brain and Behavior Research Center, University of Haifa, Haifa, Israel. 31905

Background: epidemiological studies indicate that childhood trauma is predominantly associated with higher rates of both mood and anxiety disorders, which were associated with altered limbic system functioning. Exposing rats to stressors during juvenility (27–29 days of age) has comparable effects and was suggested as a model of induced predisposition for these disorders (Avital and Richter-Levin, 2005; Tsoory and Richter-Levin, 2006; Tsoory et al., 2007).

Objectives: the current study utilized the ‘juvenile stress’ model to examine its effects on anxiety indices and exploratory behavior in the open field and elevated plus maze, as well as on circulating corticosterone levels (CORT). These effects were evaluated both soon after the exposure and in adulthood.

Results: when tested soon after the exposure to ‘juvenile stress’ (1 hr), juvenile stressed rats exhibited increased levels of activity and spent more time in the ‘unsafe’ regions of the arenas compared with unexposed rats. A reverse pattern of effects was evident when tested in adulthood (60 days of age). Adult juvenile stressed rats exhibited decreased levels of activity and spent less time in the ‘unsafe’ regions of the arenas compared with unexposed rats.

Nevertheless, a uniformed pattern of effects was found for CORT levels. In comparison with naïve rats, rats which were exposed to ‘juvenile stress’ and challenged in the open field and elevated plusmaze exhibited increased CORT levels both soon after the exposure and in adulthood.

Conclusions: Opposite pattern of effects were found in juvenility and in adulthood, but increased levels of CORT seems to be involved in both. Taken together, this differential outcome may correspond with the different short and long term effects of childhood trauma.

# Abstract: EFFECTS OF ALCOHOL STRESS ON BEHAVIOR AND HISTOLOGY OF ADRENAL IN RAT (*RATTUS NORVEGICUS*)

**Authors:** Z. Birem, Harouz, B. Asselahi

**Affiliations:** Laboratory of Behavior and Cognitive Neuroscience, Algres, Algeria

Background: Alcoholis considered stressful factor.The present study was designed to investigate the probable effects of alcohol stress on behavior, histology of adrenal gland of rat. Methods: Seventeen adulte male Wistar rat were divided into two series: controls rats (*n* = 8) receive i.p injection of saline solution (0.9%), and treated rat (*n* = 9) receive i.p injection of alcohol (1–2.25 g/kg/day) over a periode of 21 days. Locomotor activity data were collected in 5 min and recordede in vedeo camera. The left adrenal gland were quickly esccised, feed of fat tissue (4c), weighed and fixed in Bouin solution for histological study. Results: The results showed that ethanol-treated rat expressed a significant behavioral sensitization on day 21 compared with day 1. There were no changes in the response to saline on day 21 compared with day 1 in the saline treated rat. Absolute and relative adrenal weigh of ethanol treated rat were significantly increased. Histology of adrenal gland in stressed rat, stained with Masson Trichrome: zona fasciculata, reticularis and medulla showing a significant changes. Conclusion: Acohol stress can increase the locomotor activity, increasing the absolute and relative adrenal weigh that may be related to the effect of alcohol stress on the hypothalamus-pituitaryadrenal axis. Alcohol stress produce a significant changes on histology of adrenal gland.

# Abstract: CONTROL OF TYROSINE HYDROXYLASE BY GAD AND CRH AFTER CHRONIC VARIABLE STRESS

**Authors:** Daisuke Tanaka, Mikio Asakura, Shigeto Kanai, Sachiko Fujii

**Affiliations:** Department of Neuropsychiatry, St. Marianna University School of Medicine, 2-16-1 Sugao, Miyamae-ku, Kawasaki, 216-8511, Japan

Locus coeruleus(LC) is the major component of noradrenergic neurons in the brain. The corticotropin-releasing hormone(CRH) and norepinephrine(NE) are suggested to play a role in modulating the central stress response. In previous study we observed a decrease of the basal level of tyrosine hydroxylase(TH) immunoreactivity(-ir) in the LC of rats treated with chronic variable stress(CVS) for 14 days. Furtheremore a novel stressor produced an enhanced response of the TH-ir after CVS. In the present study we examined the effect of CVS on the glutamic acid decarboxylase(GAD)- ir activity of periaqueductal gray(PAG), prepositus hypoglossi( PrH) and peri-LC. The GAD-ir was significantly increased in PrH and peri-LC after CVS. The footshockinduced reactivity in the GAD-ir was decreased in both regions after CVS. Moreover, we investigated the influence of the CRH receptor antagonist, *α*h-CRH(i.c.v.) on the CVS-induced activation of the TH-ir in the LC. The *α*h-CRH i.c.v. diminished the enhanced-TH reactivity by novel stressor after CVS. Our results suggest that the GABA activity in peri-LC and PrH might regulate the LC-TH response, and also the CRH input from central nucleus of amygdala(CeA) and/or the bed nucleus of stria terminalis(BNST) might regulate the TH reactivity.

# Abstract: EFFECT OF ADULT-AND MATERNAL-ONSET CALORIE RESTRICTION ON ANXIETY-LIKE BEHAVIOUR

**Authors:** E. A. Levay, A. Govic, A. Hazi, J. Penman, A. G. Paolini, S. Kent

**Affiliations:** School of Psychological Science, La Trobe University, Bundoora VIC 3086, Australia

Calorie restriction (CR) has consistently been shown to increase lifespan and ameliorate disease outcomes; effects on behaviour are less clear, though an anxiolyticlike effect has been observed. Rats subjected to 1 of 4 dietary regimens: control, CR25 %, CR50% and, an acute episode of CR were tested in 3 tests of anxiety: open field test (OF), elevated plus maze (EPM), modified open field test (MOF). In the OF, the CR25% and CR50% groups made more central zone entries, and the CR50% spent more time there, than the control and Acute groups. The Acute group also exhibited longer latencies to leave the central zone at the onset of the test than the control and CR50% groups. In the EPM, the Acute group displayed longer latencies to enter the open arms than control and CR50 % groups and showed lower ratios of open:total armentries than all other groups. CR did not alter any variables of the MOF. Serum leptin was reduced in the CR50% only. Male offspring of dams subjected to CR25% for a brief period preconception, during gestation only, during lactation only, or lifelong (gestation, lactation, continuing post-weaning) were also tested. No differences were observed in the OF, however, in the EPM, the lifelong group engaged in greater open arm exploration than all other groups except the gestation group. For the MOF, the preconception group exhibited longer latencies to and spent less time engaged in full body emergence than controls. Serumleptin was higher in the preconception group than all other groups. Our results indicate that CR initiated in adulthood or during developmental periods leads to an altered behavioural phenotype.

# Abstract: Theme F: Pharmacology & behavior SEXUAL PHEROMONES VERSUS SWEET TASTE: OPIOID MODULATION OF TWO CHEMOSENSORY REWARDS

**Authors:** C. Agustin-Pavon, J. Martínez-Ricós, F. Martinez-García, E. Lanuza

**Affiliations:** ^1^Department of Cell Biology, Faculty of Biology, Universitat de València, Spain; ^2^Department of Functional Biology, Faculty of Biology, University of València, Spain

Although endogenous opioids are apparently involved in reward-related processes of both natural (food, sweet taste) and artificial reinforcers (opioidergic drugs such morphine), sexual behaviour is generally inhibited by opioids. In order to better understand the role of opioids in sex-related reward, we analyse the effects of opioid agonists and antagonists on the rewarding properties of male sexual pheromones for female mice [1]. We compare the effects of these drugs on sexual pheromones reward with those on a ‘classical’ nonsexual natural reinforcer, namely sucrose sweetness. To do so we have analysed the effects i.p. injections of the general opioid antagonist naloxone (1–10 mg/kg) and of the *μ*-agonist fentanyl (0.1–0.5 mg/kg) on a number of reward-related behaviours involving attraction to male chemosignals and sucrose consumption.Naloxone treatment did not affect the innate preference displayed by female mice for male chemosignals in two-choice tests against female chemosignals. In contrast, even low doses of fentanyl strongly inhibited that preference. On the other hand, the drugs had differential effects on the preference for consuming a 5% sucrose solution confronted with tap water. Thus, whereas naloxone inhibited sucrose preference, fentanyl had no effect. These results suggest that opioidergic inhibition of sexual behaviour might be due to impaired processing of pheromonal cues, at least in macrosmatic rodents, and that hedonic value of tasty solutions and sexual pheromones are under different opioid modulation.

## References

[1] Martínez-Ricós J, Agustín-Pavón C, Lanuza E, Martínez-García F (2007). Intraspecific communication through chemical signals in female mice:
reinforcing properties of involatile male sexual pheromones. *Chemical Senses*.

# Abstract: BENEFICIAL ANTIPSYCHOTIC EFFECTS OF ASCORBIC ACID ADD-ON THERAPY COMPARED TO HALOPERIDOL ALONE IN SHIZOPHRENIA, A RANDOMIZED, DOUBLE-BLIND

**Authors:** I. H. Khani

**Affiliations:** General Practices (M.D.), Research Expert of Research Center of Psychiatry and Behavioral Sciences, Mazandaran University of Medical Sciences, Mazandaran, Iran

**Background and Purpose**: Ascorbate, the endogenous form of vitamin C, is found in high concentration in the nigrostriatal system, where it appears to function as an extracellular neuromodulator. Several studies have observed that patients with several symptoms of schizophrenia have lower plasma or cerebrospinal fluid ascorbic acid concentrations. The authors conducted a trial of ascorbic acid in schizophrenic patients to evaluate its therapeutic effects.

**Materials and Methods**: In a randomized, double-blind, placebo-controlled clinical trial 40 patients with schizophrenia were assigned to haloperidol plus ascorbic acid or haloperidol plus placebo. After a washout period, 20 patients received 20 mg/day of haloperidol plus placebo and 20 received haloperidol plus 1500 mg/day of ascorbic acid for 6 weeks. Analysis of data was done by repeated measure analysis of variance (ANOVA), Newman-Keuls and Spearmans Coefficient Rank Correlation and Chi square method.

**Results**: Over 6 weeks, the ascorbic acid group showed significantly greater improvement in scores on total Brief Psychiatric Rating Scale and on positive, negative and general symptoms subscales. The total symptoms score decreased at the 1st, 2nd, 3rd, 4th, 5th, and 6th week, positive symptoms score decreased at the 1st, 2nd, 3rd, 4th, 5th, and 6th week, negative symptoms score decreased at the 2nd, 3rd, 4th, 5th, and 6th week, and general symptoms score decreased at the 3rd and 6th week.

**Conclusion**: Oral supplementation of vitamin C with conventional antipsychotic drugs can be used in the treatment of schizophrenia.

# Abstract: IMPAIRED FEAR EXTINCTION AND HIPPOCAMPAL LTP IN VGLUT1^+/−^ BUT NOT IN VGLUT2^+/−^ MICE

**Authors:** C.-V. Zsuzsanna, M. Diederik, B. Detlef, D. H. Rudi

**Affiliations:** ^1^Laboratory of Biological Psychology, University of Leuven, Belgium; ^2^Department of Functional Genomics, Johnson&Johnson Pharmaceutical Research and Development, Beerse, Belgium

Vesicular glutamate transporter (VGLUT) 1 and 2 show largely complementary brain distribution suggesting different functional roles for these transporters in brain and behavioural physiology. VGLUT1 is highly expressed in cerebellar cortex and telencephalic structures, whereas VGLUT2 expression is detected predominantly in subcortical structures. In the present study, examination of VGLUT1 and VGLUT2 heterozygote mice showed differences between these mice in extinction of conditioned fear and in hippocampal synaptic plasticity. A conditioned emotional response procedure was used to study fear-induced response suppression and extinction. In the scheduled training phase, all mice learned to poke for food reward and developed high response rates (>500 nosepokes/30 min), but VGLUT1^+/−^ poked consistently less than VGLUT2^+/−^ and littermate control mice. During 8 CER acquisition trials, tone-shock presentations suppressed nosepoke responding to a similar degree in all genotypes. During subsequent extinction trials (tones presented without footshocks), all mice gradually resumed nosepoking, but rateindependent suppression ratio calculations indicated that VGLUT1^+/−^ mice were slower to extinguish this conditioned response suppression than control and VGLUT2^+/−^ mice. In hippocampal slices prepared from these mice, VGLUT1^+/−^ but not VGLUT2^+/−^ mice showed decreased long-term potentiation (LTP) in the CA1-region. Reduced LTP in VGLUT1^+/−^ mice could decrease signal-tonoise ratio and bandwidth of information processing, and might have contributed to the defect in fear extinction in these mice.

# Abstract: AMPA ANTAGONISTS PRODUCE STRAIN-DEPENDENT NEUROPROTECTION AFTER GLOBAL BRAIN ISCHEMIA IN RATS

**Authors:** M. Ágoston, K. Móricz, G. Gigler, A. Simó, G. Szénási, L. G. Hársing

**Affiliations:** Division of Preclinical Research, EGIS Pharmaceuticals Plc, Budapest, Hungary

A short ischemia to the whole brain can cause delayed necrotic injury in few vulnerable areas, the best known of which is the CA1 field of the hippocampus. Neuroprotective compounds can prevent pyramidal cell death in the CA1 area, and robust effect in a global ischemia test is an important criterion for selecting drug candidates for the treatment of stroke. Reliability of the results is crucial for the prediction of human usefulness, which can be supported by results demonstrating lack of differences in efficacy across rat strains. For this reason we evaluated and compared the neuroprotective activity of GYKI 53405, a non-competitive AMPA receptor antagonist, in transient global cerebral ischemia produced by clamping both carotid arteries for 10 min in male Wistar and SPRD rats, in which both vertebral arteries had been occluded by electro-coagulation (4VO). Neuronal degeneration in the CA1 field was quantified with the Gallyas silver staining technique 4 days after 4VO. GYKI 53405, injected 30 min after reperfusion at 30 mg/kg i.p., reduced pyramidal cell loss in the CA1 area by 49% (*p* < 0.05) in Sprague Dawley (SPRD) rats. Surprisingly, GYKI 53405 produced no effect in Wistar rats. Administration of a large dose of NBQX, a competitive AMPA receptor antagonist, at 60, 75 and 85 min after reperfusion (30 mg/kg i.p. all) decreased neuronal cell necrosis less in Wistar (47%; *p* < 0.01) than in SPRD rats (91%; *p* < 0.01). Treatment with 7- nitroindazole, a neuronal nitric monoxide inhibitor, at the start of occlusion and 50 min after reperfusion at 25 mg/kg i.p. (both) rescued 40% and 31% of CA1 neurons in Wistar and SPRD rats, respectively. In conclusion, the neuroprotective effects of AMPA receptor antagonists were quantitatively different in Wistar and Sprague Dawley rats after global cerebral ischemia, and these findings can have significant implications for further development of AMPA antagonists for the treatment of stroke.

# Abstract: OCTOPRESSIN INFLUENCES LONG-TERM RETENTION OF A PASSIVE AVOIDANCE TASK IN THE CUTTLEFISH, SEPIA OFFICINALIS

**Authors:** I. Bardou, J. Leprince, R. Chichery, E. Maubert, H. Vaudry, V. Agin

**Affiliations:** ^1^LPCC, EA 3211, UCBN, Caen, France; ^2^INSERM U413, Université de Rouen, Rouen, France; ^3^INSERM-Avenir, EA 4102, GIP Cyceron/UCBN, Caen, France

Neurohypophysial hormones of the oxytocin/vasopressin (OT/VP) superfamily are closely related peptides that are widely distributed in vertebrate species. Apart from cyclostomes, vertebrate species possess at least one OT and one VP related peptide. Besides the classical peripheral effects of OT related peptides on reproduction, and of VP and its homologues on osmoregulation, OT and VP appear to play a key role in behavioral regulation including learning and memory. Several related peptides have been identified from diverse invertebrate species (viz., annelids, arthropods, molluscs). Moreover, it is interesting to note that two members of the OT/VP superfamily, cephalotocin and octopressin (OP), were characterized in Octopus vulgaris, an octopod cephalopod. This is a unique demonstration for the cooccurrence of two members of the OT/VP superfamily in an invertebrate species. Some of these OT-like and VP-like peptides seem to be involved in peripheral processes similar to that noted in mammals. However, there is no data to date, concerning their central effect in invertebrate species. By means of immunohistochemistry techniques, we investigated the distribution of OT/VP superfamily peptides in the central nervous system of the cuttlefish, Sepia officinalis, a decapod cephalopod. We reported the presence of at least two closely related peptides in cerebral structures known to be involved in feeding behavior, intra- and inter-specific communications and learning and memory systems. Thus, we investigated the effects of OP on the long-term retention of a passive avoidance task. OP was injected intravenously 1 h after the training phase; retention was tested 24 h post-training. Injections of 3 and 60 *μ*g/kg of OP induced a significant increase and decrease, respectively, in long-term memory performance. This study shows, for the first time in an invertebrate, the implication of an OT/VP-like peptide in learning and memory.

# Abstract: CENTRAL NERVOUS SYSTEM ACTIVITY OF LOTUS CORNICULATUS VAR. ALBINUS

**Authors:** Ö. D. Can, Ü. Demir, F. Mor, Y. Öztürk

**Affiliations:** ^1^Department of Pharmacology, Anadolu University, Eskisehir, Turkey; ^2^Department of Pharmacology and Clinical Toxicology, Akdeniz University, Burdur, Turkey

Lotus corniculatus var. albinus (Leguminosae) is a common flowering plant native to grassland temperate Eurasia and North Africa. The plant is generally known as Birdsfoot Trefoil. Traditionally, Lotus corniculatus has been used in folk medicine for its sedative effects in several countries including Turkey. To the best of our knowledge, there has been any reports about the central nervous system (CNS) activity of this plant. So, the aim of our study was to investigate the possible CNS activity of Lotus corniculatus plant extract. The effects of the extract (100 mg/kg) on explatory behaviour, depression level, spontaneous motor activity and motor coordination were investigated in mice. Intraperitonal (i.p) administration of the extract induced a significant decrease in exploratory behaviour in hole-board tests and spontaneous motor activity in activity cage measurements and increase in immobilitiy time in tail suspension tests. On the other hand, the extract failed to inhibit motor coordination in Rota-Rod tests. The decrease in vertical and horizantal motor activity and exploratory behaviour indicates the reduce in CNS excitability which might be closely related to sedation based on depression of the CNS. In spite of decreased spontaneous motor activity in the activity cage, changes on motor coordination in Rota-Rod test was not significant suggesting an effect unrelated with neuromuscular blockage. Increase in the immobility time in tail suspension test pointed out the antidepressant like activity of the plant extract. Further studies are neccesary to explain the mechanisms of this antidepressant like activity.

# Abstract: Behavioral deficits in the cuprizone-induced murine model of demyelination/remyelination

**Authors:** N. Franco-Ponsa, M. Torrente, M. T. Colomina, J. Tomas, E. Vilella

**Affiliations:** ^1^Unitat de Psiquiatria i Psicologia Mèdica, Facultat de Medicina i Ciències de la Salut, Universitat Rovira i Virgili, Reus, Spain; ^2^Unitat de Psicobiologia, Departament de Psicologia, Facultat de Ciències de l'Educació i Psicologia, Tarragona, Spain

The neurotoxicant cuprizone has been used extensively to create a mouse model of demyelination. However, the effects on behavior of cuprizone-treatment have not been previously reported. We have analyzed the behavioral changes of mice given a diet containing 0.2% cuprizone for 6 weeks followed by 6 weeks of recovery. Behavior was assessed using a range of tests: the functional observation battery, the open-field test, and the rota-rod test. Concurrent with the start of demyelination, at 3 and 4 weeks of 0.2% cuprizone treatment, the animals exhibited an increase in central nervous system activity and an inhibited anxiogenic response to the novelty challenge test. At 5 weeks of treatment (the period of maximal demyelination) equilibrium was altered and sensorimotor reactivity was also affected. Further, rotarod analysis demonstrated that the treated group had poorer motor coordination than control animals. This effect was not reversed 6 weeks after cuprizone withdrawal. The animals in the recovery period also exhibited difficulties in the rota-rod progressive learning task. Our results indicate that behavioral deficits follow the course of demyelination-remyelination induced by administration of 0.2% cuprizone, and that some of the changes persist even after 6 weeks on normal diet.

# Abstract: HIPPOCAMPAL THETA RHYTHM AFTER BICUCULLINE MICROINJECTION INTO THE ROSTRAL PART OF NUCLEUS RETICULARIS PONTIS ORALIS IN RATS

**Authors:** M. Kroplewski, E. Jurkowlaniec

**Affiliations:** Department of Animal Physiology, University of Gdansk, Poland

It has been well established that the nucleus reticularis pontis oralis (RPO) is one of the crucial structures for the regulation of hippocampal theta rhythm, but involvement of rostral and caudal parts of this structure is different. The caudal part is effective in inducing theta rhythm while the rostral part of the RPO may contain neurons which inhibit the theta via unknown transmitter. At least a part of these inhibitory neurons could be glycinergic, because strychnine (a glycine receptor antagonist) microinjections released the theta activity as we found in our previous studies; now we tested the GABA transmission involvement. In the present experiment the effect of GABAA receptor blockade in the rostral part of the RPO on hippocampal theta rhythm was examined in urethaneanesthetized rats. Male Wistar rats (*n* = 10) were implanted with recording electrodes in the dorsal hippocampus and received microinjections of GABAA receptor antagonist, bicuculline (50 ng/0.5 *μ*L, over 3–6 min) into the rostral part of RPO. Theta rhythm was induced by sensory stimulation (60 s tail pinch) in the control condition and every 10 min after bicuculline. The effect of GABA_A_ receptor blockade was observed for 1 hour. Unilateral bicuculline injections induced spontaneous theta episodes without sensory stimulation in both hippocampi (ipsi- and contralateral to the injection site), with a mean latency of 207.8 ± 11.4 s. Mean duration time of spontaneous theta episodes was 940.3 ± 70.2 s. The hippocampal EEG signal and the fast Fourier transform (FFT) power spectra during control theta and theta rhythm after bicuculline were similar. The results obtained indicate that inhibition of GABA-ergic or GABA-sensitive neurons in the rostral part of the RPO could elicit the hippocampal theta activity, the bicuculline effect on theta elicitation we found even more evident than strychnine.

# Abstract: THE EFFECT OF 5-HT RECEPTORS ACTIVATION IN THE PEDUNCULOPONTINE TEGMENTAL NUCLEUS ON THE THETA RHYTHM IN ANESTHETIZED RATS

**Authors:** P. Matulewicz, E. Jurkowlaniec

**Affiliations:** Department of Animal Physiology, University of Gdansk, Poland

The pedunculopontine tegmental nucleus (PPN) is thought to play an important role in the generation and regulation of hippocampal theta activity. In the present study the effect of 5-carboxyamidotryptamine (5-CT), a selective 5-HT_1A_, 5-HT_1B_, 5-HT_1D_, 5-HT_5_ and 5-HT_7_ receptors agonist injected into the PPN on hippocampal EEG activity was examined in urethanized rats. The experiments were performed on 7 male Wistar rats implanted with bilateral recording hippocampal electrodes and unilateral injection cannula into the PPN. The animals were maintained at the level of anesthesia at which spontaneous theta rhythm was not present in hippocampal EEG, but could be elicited by sensory stimulation (tail pinch lasting 1 min). The hippocampal EEG was recorded during the sensory stimulation in the preinjection conditions and after unilateral injections of 5-CT into the PPN. Microinjection of 5-CT (1 *μ*g/0.5 *μ*L, lasted at least 3 min) induced hippocampal regular spontaneous theta activity in both hippocampi, with a mean latency of 16.7 ± 2.1 min and with mean duration of 31 ± 2.9 min. The hippocampal EEG signal and fast Fourier transform (FFT) power spectra during control theta and theta rhythm after 5-CT infusion were similar. Clear synchronization of the hippocampal EEG associated with an increase in FFT power in theta band (3–6 Hz) and power reduction in delta band (0.1–3 Hz) were observed. It is thought that serotonergic system inhibits the mesopontine cholinergic neurons (the PPN is a part of cholinergic theta activation system), which activation is crucial for the induction of theta rhythm in the hippocampus. Presently available evidence indicates that activation of 5-HT receptors on PPN neurons generally induce their hyperpolarization. The results obtained suggest that local inhibition of presumably cholinergic neurons in the PPN could elicit hippocampal theta rhythm without sensory stimulation.

# Abstract: TESTING OF SELECTIVE AND NONSELECTIVE GABA_(A)_ MODULATORS IN THE FOUR-PLATE TEST USING PUNISHED AND UNPUNISHED CROSSINGS

**Authors:** A. N. Nielsen, J. P. Redrobe

**Affiliations:** Neurosearch A/S, Pederstrupvej 93, 2750 Ballerup, Denmark

Evidence suggests that GABA_(A)_ receptors containing an alpha1 subunit mediate the sedative effect of unselective benzodiazepines like diazepam and chlordiazepoxide, whereas receptors with an alpha2 or 3 subunit mediate the anxiolytic effect of these compounds. It is important to be able to detect the separation between potential sedative and anxiolytic effects when testing novel compounds with subtype selective properties for GABA_A_, pre-clinically.

In this study, we attempted to distinguish between the effects of non-selective and selective GABA_A_ agonists, using a punished and unpunished crossings paradigm in the fourplate test. In addition, we examined whether subtype selective or partial agonists at the GABA_A_ receptor would show less sedative effects. Female NMRI mice were used for testing of the following compounds: diazepam (1, 3, 10 mg/kg) and chlordiazepoxide (5, 10, 20 mg/kg, both unselective GABA_A_ full agonists), zolpidem (1, 3, 10 mg/kg, selective GABA_A_-*α*_1_ agonist), SL651498 (1, 3, 10 mg/kg, GABA_A_-*α*_2_ > GABA_A_- *α*_3_), NS2710 (1, 3, 10 mg/kg, nonselective partial agonist), and the centrally stimulating agent methamphetamine (0.3, 1, 3 kg/kg, dopamine releaser). All compounds were administered intraperitonally with a pretreatment time of 30 min. Diazepam (1&3 mg/kg), chlordiazepoxide (10&20 mg/kg), and NS2710 (3&10 mg/kg) displayed anxiolytic-like effects as evidenced by an increase in punished crossings. SL651498 did not display anxiolytic-like activity at the doses tested. Zolpidem reduced the number of unpunished crossings (1&10 mg/kg), whereas methamphetamine showed central stimulating effects under unpunished conditions, translating to a false-positive anxiolytic-like effect under punished conditions.

In conclusion, the data suggest that activity at all 3 GABA_(A)_ receptor subunits may be necessary to elicit anxiolytic-like activity in the four-plate test, and thus may not be suitable for the identification of subtype selective compounds. The unpunished crossings component may be useful to identify potential false-positives.

# Abstract: CENTRAL NERVOUS SYSTEM DEPRESSANT ACTIVITY OF OLEA EUROPAEA L. LEAVES EXTRACT

**Authors:** Y. Öztürk, D. Ö. Can, Ü. Demir, N. Bektas

**Affiliations:** Department of Pharmacology, Faculty Pharmacy, Anadolu University, Eskisehir, Turkey

Olea europaea L. (Oleaceae) is an evergreen tree naturally grown in the Mediterranean and South Europe. Olive fruits and oil have been recognized as important components of a healthy diet because of their polyphenol contents. Olive leaves have been shown to have similar polyphenolics composition to olive fruits. Tablets of olive fruit and olive leaf extracts have been marketed as a dietetic product. The plant has been shown to have antioxidant, antihypertensive, antiatherogenic, vasodilatory, antiarythmic, cardioprotective, antiobesity, hypoglycemic, antiinflammatory, antitumoral, antimicrobial, antiviral activities.Historically, the olive leaves have been used for their antimalarial, antipyretic, antiseptic, astringent, febrifuge and sedative effects. Because of the lack of information about the central nervous system (CNS) effects of olive leaves extracts and the historical usage of the leaves for their sedative effects, we aimed to investigate the CNS activities of this extract. Olive leaves extracts (125– 250 mg/kg) was used for this study. Elevated plus maze and hole board tests for anxiety, activity cage measurements for spontaneous motor activity, Rota-Rod tests for motor coordination were studied. Both doses of extracts decreased the number of head-dips in hole-board tests. Mice spent more time in closed arms of maze. Significant decreases in the horizontal and vertical locomotor activities were observed. The decrease in spontaneous motor activity is an indicator for decreased excitability of the CNS. This decrease might be closely related to sedation due to the CNS depression. Decrease in the exploratory behaviour are in general agreement with these reports. The decreased spotaneous motor activity seems to be not related to a neuromuscular blockade, as Rota-Rod performance was not changed. Rather, the effects might comprise the neurons that modulate CNS depressant activity.

# Abstract: EARLY LIFE SEIZURES INDUCE BEHAVIORAL AND NEUROBIOLOGICAL ALTERATIONS IN ADULT RAT

**Authors:** E. Papalexi, M. Dosi, O. Chouliara, R. Liasko, M. Marselos, C. Psarropoulou, K. Antonio

**Affiliations:** ^1^Department of Pharmacology, Faculty of Medicine, University of Ioannina, Ioannina, Greece; ^2^Laboratory of Physiology, Department of Biological Applications and Technologies, Faculty of Science and Technology, University of Ioannina, Ioannina, Greece

Recurrent seizures in infants are highly associated with cognitive deficits in adulthood and most epileptic syndromes are first manifested in infancy or childhood. A variety of experimental animal models has been developed in order to clarify the impact of early life seizures in the CNS function. However, relatively little is known about the longterm behavioral and neurobiological consequences of early seizures. Pentylenetetrazol (PTZ), a GABA_A_ receptor antagonist causes convulsions that model primary generalized epilepsy. In the present study the long-termeffects of PTZ administration (90 mg/kg, i.p.), in early life of male (9 controls, 12 PTZ-treated) and female (19 controls, 15 PTZ-treated) rats (P18–20), were examined. In particular, behavioral responses such as open field activity, object recognition and water maze performance were assessed in adult rats of both sexes. BDNF and neurofilament hippocampal protein levels were also measured. Adult Female, but not Male, PTZ-treated animals presented a reduced motor activity. Adult Male, but not Female, PTZ-treated animals displayed an impaired ability to recognize a novel object and exhibited learning but not memory impairment in the Morris water maze test. Moreover, BDNF hippocampal protein levels were significantly reduced in Male but not Female PTZ-treated animals. These findings suggest that a sustained early life convulsion induced sex dependent behavioral changes linked to reaction to novelty, spatial and non-spatial memory performance along with alterations in neurobiological parameters indicating neuroplasticity. These changes are important for animal survival, although not directly linked to adult life epilepsy manifestations (convulsion threshold).

# Abstract: EARLY MATERNAL DEPRIVATION INDUCES LONG TERM EFFECTS ON BEHAVIORAL AND NEUROBIOLOGICAL PARAMETERS IN THE ADULT RATS

**Authors:** G. Rentesi, K. Antoniou, M. Marselos, M. Syrrou, M. Konstanti

**Affiliations:** ^1^Department of Pharmacology, Faculty of Medicine, University of Ioannina; ^2^Department of Biology, Faculty of Medicine, University of Ioannina, Greece

Early maternal deprivation (MD) has been considered as an animal model of early life stress. A single 24 hour period of MD in rats has been associated with a number of abnormalities in brain and behavior in adulthood. These alterations seem to be relevant to the neurobiological substrate of depression or schizophrenia. The present study investigated specific behavioral and neurobiological parameters in MD as compared to control rats. Behavioral responses such as the spontaneous activity in a novel open field apparatus, the habituation to learning using an open field chamber, the behavior response to d-amphetamine (d-amp,0.5 and 1.5 mg/kg) and the susceptibility to apomorphine (1.5 mg/kg), were examined. Additionally, DARPP-32 striatal protein levels were also measured. Plasma corticosterone levels were estimated in both groups of rats. The findings showed that MD rats exhibited a marginal increase in spontaneous motor activity as compared to controls. During repeated exposure to the open field chamber the MD rats exhibited an impaired habituation to learning in comparison with control rats. MD rats displayed an exaggerated motor activity following the low dose of d-amp, compared with control rats. Similar increase was also observed in MD rats following the high dose of damp but it did not reach statistical significance. Apomorphine induced gnawing was markedly increased in MD as compared to controls. It is noteworthy that striatal DARPP-32 striatal protein levels were increased in MD rats. Compared to controls, MD rats displayed elevated corticosterone levels. These findings clearly showed that early maternal deprivation stress produce long term consequences in distinct behavioral and neurobiological parameters. These behavioral modifications are associated with motor activity and cognitive abilities while they might be linked to alterations in the dopaminergic function and in the hypothalamic-pituitaryadrenal axis status.

# Abstract: DEVELOPMENT A NEW MOUSE MODEL TO STUDY LEARNING BEHAVIOUR AND IMPULSIVITY: INTELLICAGE SYSTEM

**Authors:** O. Yu Vekovischeva, E. V. Verbitskaya, S. Mansikkamaki, E. R. Korpi

**Affiliations:** ^1^Institute of Biomedicine/Pharmacology, University of Helsinki, Helsinki, Finland; ^2^Laboratory of Biomedical Statistics, Pavlov State Medical University, St. Petersburg, Russia

The personal characteristic such as impulsivity plays an important role in normal behavior and in such pathological forms as mania and personality, substance abuse and attention deficit/hyperactivity disorders. The lack of a unitary mouse model of impulsivity suggests developing new methods for better mouse phenotyping. Intellicage system (New- Behavior Lab, Zürich, Switzerland) that monitors automatically mouse drinking and nose-poking (NP) behaviors of the four corners with the help subcutaneous personal transponders was used to study mouse learning behavior and impulsivity in three different forced drinking regimes. All these regimes were programmed so that each animal was entitled to drink clockwise from each corner. The first regime, Radial (Rd), allowed drinking at large during the visit. Time Limited (TLm) regime limited the time of one drink for 3 sec but animals could continue to drink more by NP if they stayed in the corner. Fixed (Fx) regime limited the time of the drink for 5 sec and allowed to drink from one corner only once. C57Bl/J6 males learned successfully on the first day under all regimes but Fx regime appeared more powerful. The learning was evaluated as decreased number of incorrect visits. The impulsivity was evaluated as number of incorrect visits in each corner as well as number of mistakes between correct visits. The persistence was calculated as ratio of average daily incorrect NPs to all NPs in each corner. The learning behavior was also studied in situation when some water bottles were replaced by sucrose and quinine solutions that increased incorrect visits and NPs according to context. Phencyclidine injections (s.c., 0.93 mg/kg, daily for 7 days) increased impulsivity and persistence in the replacement situation. Fx forced drinking regime might be used as a model to analyze mouse learning behavior and impulsivity and its pharmacological control.

# Abstract: DOPAMINERGIC ACTIVITY FACILITATES RETENTION AND RETRIEVAL OF AUDITORY CORTEX-DEPENDENT MEMORY

**Authors:** H. Schicknick, K. H. Smalla, H. Scheich, E. D. Gundelfinger, W. Tischmeyer

**Affiliations:** Leibniz Institute for Neurobiology, Magdeburg, Germany

In the Mongolian gerbil, the auditory cortex is critical for discriminating the modulation direction of linearly frequencymodulated tones (FMs). Using a pharmacological approach, we elucidated the functional significance of the dopamine (DA) system for FM discrimination learning. When compared with vehicle-treated controls, bilateral injections of a DA agonist into the auditory cortex 24 h before or shortly after the first session of differential conditioning to FMs facilitated learning of the discrimination reaction. This effect was sensitive to a D1-like DA receptor antagonist and to protein synthesis inhibitors. The agonist-induced improvement was confined to auditory cortex-dependent aspects of the task, that is, discriminating the conditioned stimuli and/or associating them with their respective meanings. Acquisition performance was normal, implying that D1-like DA receptor activation supports encoding of long-term memory. To assess effects on mechanisms required for retrieval of the learned behaviour, the DA agonist was applied to well-trained gerbils shortly after they repeated the established FM discrimination reaction in a retraining session. On subsequent days, the accuracy of FM discrimination monitored at the beginning of retraining progressively increased in agonisttreated gerbils. In contrast, the mean discrimination scores per session were not significantly affected by this treatment. Accordingly, pharmacological interference with the DA system shortly after the first session of differential conditioning caused deficits in the discriminative behaviour that were evident predominantly during the initial trials of retraining 24 h later. Together, our findings suggest that DA activity in the gerbil auditory cortex facilitates consolidation-relevant processes required for both retention and retrieval of FM discrimination memory.

# Abstract: OXYTOCIN ENHANCES STRESS-INDUCED GROOMING BEHAVIOR IN WISTAR RATS; COMPARISON WITH THE EFFECT OF CARBETOCIN

**Authors:** V. Klenerova, S. Hynie

**Affiliations:** Institute of Medical Biochemistry, Laboratory of Neuropharmacology, 1st Medical Faculty, Charles University in Prague, Prague, Czech Republic

Oxytocin which has primarily the peripheral effects was shown to have some central actions even when administered peripherally. The aim of this study was to test the central effects of oxytocin and its partial agonist/antagonist carbetocin on rat behavior in the open-field device during the stress. Male Wistar rats were used and immobilization was applied for 60 minutes. Tested drugs were administered i.p. immediately after stress termination, and after 60 min lasting pause the behavioral test in the open-field device was performed. We used a circular arena with diameter 150 cm, and the behavioral parameters including grooming were videorecorded and evaluated with the Any Maze software (Stoelting Co, USA). When compared to controls oxytocin and carbetocin (in doses 1 mg/kg b.w.) increased slightly grooming while other parameters in the open-field device differed according to the measured parameter. The effect of both drugs substantially differed in the experiment using stress. Exposure to stress alone increased grooming and oxytocin potently enhanced this behavioral pattern. Carbetocin had only insignificant increasing effect on grooming in stressed animals. Restraint stress produced typical decrease of certain behavioral parameters, like total movement distance or rearing. Carbetocin very effectively antagonized these stress effects. Grooming seems to be an important behavioral parameter, which is useful for evaluation of brain effects of peptides previously believed not to penetrate blood-brain barrier.

# Abstract: NEUROPROTECTIVE EFFECTS OF EGIS-9637, A NON-COMPETITIVE AMPA RECEPTOR ANTAGONIST

**Authors:** K. Móricz, G. Gigler, M. Ágoston, A. Simó, G. Kapus, M. Végh, S. Kertész, G. Lévay, G. Szénási, I. Gacsályi

**Affiliations:** Division of Preclinical Research, EGIS Pharmaceuticals Plc.

Blockade of the AMPA type ionotropic glutamate receptors has been shown to prevent neuronal loss in ischemia/ reperfusion, trauma and slowly progressing neurodegenerative disorders by reducing excitotoxic damage due to excessive glutamate release. EGIS-9637 is a noncompetitive AMPA receptor antagonist compound with a 2,3-benzodiazepine chemical structure. The present study was undertaken in order to compare the in vitro efficacy and in vivo neuroprotective effects of EGIS-9637 to those of GYKI 53405. In patch clamp measurements, EGIS-9637 inhibited the kainate-evoked whole cell currents in cultured rat telencephalon neurones (IC50 = 2.5 microM), population spikes in rat hippocampal slices (IC50 = 2.1 microM) and the AMPA-induced spreading depression in chicken retina (IC50 = 1.1 microM); these in vitro effects of EGIS-9637 were stronger than those of GYKI 53405 (IC50 values were 7.6, 17.8 and 7.0 microM, respectively). In global cerebral ischemia induced by 3-min bilateral carotid occlusion in gerbils, EGIS-9637 or GYKI 53405 (4 × 15 mg/kg i.p.) administered at 30, 45, 60 and 75 min after reperfusion, decreased neuronal death at day 4 in the CA1 area of the hippocampus by 35% and 61%, attenuated hypermotility by 79% and 72%, respectively. In global cerebral ischemia induced by 10- min four vessel occlusion in rats, EGIS-9637 and GYKI 53405 (30 mg/kg i.p.) administered at 30 min after reperfusion, produced similar reductions of the ischemic damage (75% and 78%, respectively). In focal cerebreal ischemia, both compounds dose-dependently reduced cerebral infarct size after permanent middle cerebral artery occlusion in mice and rats. In both species, EGIS-9637 showed stronger neuroprotective activity (minimal effective dose, MED = 0.3 mg/kg i.p. in mice and 0.03 mg/kg i.p. in rats) than GYKI 53405 (MED = 3 mg/kg in mice, MED = 10 mg/kg i.p. in rats). The favourable neuroprotective effect of EGIS-9637 indicates good clinical perspectives in the treatment of human ischemic stroke.

# Abstract: NEUROKININ-2 ANTAGONISM INMEDIAL SEPTUM INFLUENCES ACH NEUROTRANSMISSION AND OBJECT MEMORY FOR RECENCY AND DISPLACEMENT

**Authors:** S. Schäble, E. Dere, J. P. Huston, M. A. De Souza Silva

**Affiliations:** Institute of Physiological Psychology, Heinrich Heine University of Düsseldorf, Germany

Three neurokinin receptors, namely neurokinin-1 (NK1), NK2 and NK3 receptors, have been identified in the brain on cholinergic neurons. NK2 receptors are predominant and functionally identified in the hippocampus, thalamus, septum and frontal cortex. Previously it was shown that in the medial septum NK2 receptor antagonists blocked the increase of acetylcholine release in the hippocampus induced by septal NK2-agonist (NKA) administration. Therefore, it is possible that NK2 receptors in the medial septum can influence memory and that selective NK2 receptor antagonists would affect learning and memory by influencing the activity of the cholinergic neurons of the basal forebrain. The intent of this study was to investigate the action of local application of the peptidic NK2 receptor antagonist Bz-Ala-Ala- D-Trp-Phe-D-Pro-Pro-Nle-NH (1, 10 and 100 pmol) into the medial septum on ACh levels in projection areas of the cholinergic neurons of the basal forebrain, namely frontal cortex, amygdala and hippocampus by in vivo microdialysis and HPLC. Furthermore, we investigated the pharmacological effect of the NK2 receptor antagonism on object memory for recency (“what” and “when”) and spatial displacement (“what” and “where”). Local injection of the NK2 antagonist into the medial septum decreased ACh levels in the frontal cortex, amygdala and hippocampus 30 minutes after pharmacological treatment in a dose dependent manner. The strongest decrease was observed with 10 pmol treatment. We also found that vehicle injection into the medial septum impaired object memory for recency and displacement, which could partially be reversed by local application of the NK2 receptor antagonist into the medial septum. The results suggest a possible role of the NK2 receptor in learning and memory without influencing the activity of the cholinergic neurons of the basal forebrain in a direct manner.

# Abstract: A RAT VERSION OF THE IOWA GAMBLING TASK REVEALS GOOD AND POOR DECISION-MAKERS AS IN HUMANS

**Authors:** M. Rivalan, T. Guegan, S. Ahmed, F. Dellu-Hagedorn

**Affiliations:** CNRS, Université Bordeaux 2, Université Bordeaux 1, UMR 5227, Bordeaux, France

Deficits in decision-making are a key component in several psychiatric disorders like conduct disorders or drug addiction, in which immediate gratification is chosen at the expense of long-term goals. Such deficits can be revealed in the Iowa Gambling Task, designed to mimic complex reallife decision-making. This task involves conflicting options in which greatest gains are associated with higher unpredictable penalties. We devised a rat gambling task based on the same principle. Food-restricted rats had to choose between four holes, each associated with the delivery of different amounts of reward followed by unpredictable penalties of variable duration. Despite the difficulty of the task, most rats rapidly developed a preference for the more favorable choices within one session. However, about a quarter of the rats kept choosing unfavorably options and a minority was undecided. These reproducible behavioral differences were stable over time and were not related to a lower motivation to perform the task or to anxiety levels. Bad decision-makers were not less sensitive to punishment but tended to take more risk in a plus-maze and had a higher motivation to collect food reward in a runway. These results suggest thatmost rats exhibit capacities of making the best decision in such a complex task whereas others fail to do so. These findings strikingly parallel those obtained in humans. Our data support the hypothesis that poor decision-making in normal individuals is related to less risk assessment and a higher motivation for food reinforcement. This task that allows a quick one-trial measure may facilitate the search for the psychobiological bases underlying spontaneous or experimental deficits in decision making.

# Abstract: CORRELATION BETWEEN *IN VLTRO* AND *IN VLVO* TESTS IN THE PHARMACOLOGICAL SCREENING OF NEWPOTENTIAL SEDATIVE-HYPNOTIC MOLECULES

**Authors:** A. M. Alcántara, M. Princep, C. Pérez, J. Terencio, E. Méndez, I. Buira, L. Anglada, A. Palomer, A. Guglietta

**Affiliations:** R+D Center Ferrer International S.A., Barcelona-Spain

**Rationale**: Most used compounds for insomnia treatment are non-benzodiazepines drugs with GABA_A_ *α*-1 selectivity. The aim of this study was to present the screening data of new chemical entities (NCEs) of a novel 7-substituted-3-nitropyrazolo[1.5-a]pyrimidines serie, comparatively to some marketed compounds and to validate the screening performed.

**Results**: 83% of tested NCEs presented affinity higher than 25% in the in vitro binding assay for GABA_A_ *α*-1 at 10^−7^ M and sedative-hypnotic effect in mice higher than 60% in inhibition of the spontaneous motor activity test (i-SMA) at 97.6 *μ*mol/Kg ip. NCEs that showed affinity lower than 25% at 10^−7^ M, elicit low sedation induction (i-SMA below 60%). Zolpidem (*α*-1 affinity: 73.6%; i-SMA: 91.7%) and zaleplon (*α*-1 affinity: 23.4%; i-SMA: 47.2%) results fit within this range of activity in both assays. SMA was recorded during light phase although is the rest phase of rodents. In 4 representative NCEs, selectivity for GABA_A_ receptor was tested against a battery of other central nervous system receptors at 10^−6^ M and negligible affinity was found, confirming that sedation induction can be attributed only to the GABA_A_ modulation. Dose-response curves in GABA binding and i- SMA were performed in two selected NCEs, GF-001952-00 (Ki = 88.6 nM; ED_50_ = 7.8 *μ*mol/Kg ip) and GF-003956-00 (Ki = 11.1 nm; ED_50_ = 1.9 *μ*mol/kg ip). These data fit in the same or higher activity than zaleplon (Ki = 38.2 nM; ED_50_ = 4.4 *μ*mol/Kg ip) and zaleplon (Ki182.6 nM; ED_50_ = 6.2 *μ*mol/Kg ip). Conclusions: In this screening, direct relationship between in vitro and *in vivo* data was established. Despite the in vivo test was performed during light phase (rodents rest phase), enough sensitivity to discriminate and select sedative-hypnotic NCEs was demonstrated. Finally GF- 001952-00 and GF-003956-00, displayed equivalent preclinical efficacy than marketed compounds. Further assays should to be performed to confirm the potential use as sedativehypnotics of these promising compounds.

# Abstract: AM251 REDUCES FOOD INTAKE AND BODY WEIGHT IN FREE-FEEDING MICE RECORDED IN THE “PHENOTYPER”

**Authors:** L. Robinson, S. McKillop-Smith, G. Riedel

**Affiliations:** School Medical Science, University of Aberdeen, Aberdeen, UK

The endocannabinoid system and particularly the CB1 receptor plays an important role in the modulation of food intake in both humans and animals. Previous studies have revealed that cannabinoid agonists including D9- tetrahydrocannabinol increase food intake and that this action can be reversed by CB1 antagonists whereas treatment with the CB1 antagonists SR141716A or AM251 alone reduces food intake. The aim of this study was to determine the effect of AM251 on feeding and exploratory behaviour of free feeding mice. C57BL6 mice were individually housed in Phenotyper cages and subjected to a 12/12 hour light/dark cycle with free access to food and water. The Phenotyper is a video-based observation system that allows longterm continuous monitoring of behavioral activity. Animals were given 2 days of habituation prior to being matched for body weight and allocated to drug groups of AM251 (10 mg/kg) or vehicle (Tween 80) (administered before dark cycle). The body weight of the mice, weights of food hoppers and water bottles were recorded each morning. Motor activity and time spent in predefined areas of the arena associated with food and water were also monitored 24 hours per day. An acute injection of AM251 induced a reduction in body weight and food intake independent of gender. AM251 treated animals were observed as spending less time in the area associated with food. In addition to the effects of AM251 on feeding behaviour the mice revealed a decrease in activity compared to controls in the hours following drug treatment. Repeated dosing with AM251 reduced body weight, food intake and time spent in areas associated with food and water during 4 days of testing. These results corroborate previous findings which have observed that AM251 produces a reduction in food intake and in turn support a possible therapeutic use of AM251 as an anorexic drug and treatment for obesity.

# Abstract: ANTIDEPRESSANT-LIKE EFFECTS OF RIMONABANT IN THE MOUSE FORCED SWIM TEST OCCUR INDEPENDENTLY OF MONOAMINERGIC SIGNALING

**Authors:** M. A. Steiner, G. Astarita, V. Vialou, A. Graham, F. Holsboer, D. Piomelli, B. Lutz, C. T. Wotjak, E. J. Nestler

**Affiliations:** ^1^Max Planck Institute of Psychiatry, Munich, Germany; ^2^Department of Physiological Chemistry, Johannes Gutenberg University, Mainz, Germany; ^3^Department of Psychiatry, UT Southwestern, Dallas, TX, USA; ^4^Department of Pharmacology, University of California, Irvine, CA, USA

The forced swim test (FST) is a behavioral assay in rodents that predicts the clinical efficacy of many types of antidepressants. It was the aim of the current study to systematically evaluate the effects of endocannabinoid modulating drugs in the mouse FST and to further delineate the underlying mechanisms. We show that acute treatment with the cannabinoid type 1 (CB1) receptor antagonists SR141716 (rimonabant) and AM251 dose-dependently reduce immobility in the FST in male C57BL/6N mice without affecting locomotor activity. This antidepressant-like effect is specific for CB1 receptors as SR141716 exerts its action only in CB1 wt, but not in CB1 ko mice. The evaluation of the endocannabinoid level enhancing drugs URB597, AM404 and UCM707 did not reveal any influence on immobility in the FST. Depletion of serotonin or catecholamines via inhibition of tryptophan hydroxylase and tyrosine hydroxylase, respectively, did not attenuate antidepressant-like behavioral or neuroendocrine effects of SR141716. Finally, FST exposure broadly reduced anandamide levels in the ventromedial prefrontal cortex (VPC), the nucleus accumbens (NAC), the basolateral amygdala (BLA), the dorsal hippocampus (DH) and the raphe nuclei, but not in the caudate putamen (CPU). 2-arachidonyl glycerol levels were downregulated in the DH, but upregulated in the BLA and CPU. In conclusion, we illustrate the complexity of the endocannabinoid signaling response to FST stress and propose that the acute pharmacological blockade of CB1 receptors can lead to antidepressantlike effects in the mouse FST, which are likely not mediated via increased monoaminergic neurotransmission.

# Abstract: EEG POWER SPECTRUM AND HEART RATE EFFECTS OF RIMONABANT IN THE ANAESTHETISED RAT

**Authors:** E. A. Shanks, A. Kulla, M. Tricklebank

**Affiliations:** Eli Lilly and Co Ltd, Hampshire, UK

The hippocampus and cortex express appreciable densities of CB1 receptors [1] which are possible targets for the CB1 antagonist rimonabant (SR141716A). The aim of the current study was to investigate the effects of rimonabant on hippocampal and cortical EEG power spectrum and cardiovascular parameters in the anaesthetised rat. Experiments were performed in accordance with the Animal (Scientific Procedures) Act 1986. Adult male Sprague-Dawley rats were terminally anaesthetised with urethane (1.2 g/kg i.p.). EEG was recorded from two sites using a stainless steel electrode in CA3 and cortical skull electrodes. A femoral artery was cannulated to allow blood pressure and heart rate to be monitored. EEG and physiological parameters were recorded prior to and for 90 minutes after rimonabant (10 mg/kg) or vehicle administration (1 ml/kg i.p., *n* = 9 per treatment). Power spectrum analysis was conducted in 5 minute bins at 30, 60 and 75 min post-drug. Rimonabant produced no change in power over the frequency range of 1–40 Hz recorded from either the CA3 or cortical electrodes. However, there was a significant increase in CA3 total power compared to baseline at 60 and 75 minutes post-drug (*p* < 0.005 ANOVA with post-hoc Tukey Kramer). No increase was observed in cortical total power. Blood pressure andHR showed no significant change during the experiment. Previous studies have shown that the cannabinoid agonist CP55940 decreased EEG power and that this can be antagonized by rimonabant [2]. The current study is the first to show an effect of rimonabant alone on hippocampal EEG power. However, we did not observe the decrease in cortical power reported by [3] at doses similar to that used here. Heart rate was not altered by rimonabant in the current study, which agrees with data from awake monkeys [4].

## References

[1] Herkenham M, Lynn AB, Johnson MR, Melvin LS, de Costa BR, Rice KC (1991). Characterization and localization of cannabinoid receptors in rat brain: a quantitative in vitro autoradiographic study. *Journal of Neuroscience*.

[2] Robbe D, Montgomery SM, Thome A, Rueda-Orozco PE, McNaughton BL, Buzsaki G (2006). Cannabinoids reveal importance of spike timing coordination in hippocampal function. *Nature Neuroscience*.

[3] Santucci V, Storme J-J, Soubrié P, Le Fur G (1996). Arousal-enhancing properties of the CB1 cannabinoid receptor antagonist SR 141716A in
rats as assessed by electroencephalographic spectral and sleep-waking cycle analysis. *Life Sciences*.

[4] Vivian JA, Kishioka S, Butelman ER, Broadbear J, Lee KO, Woods JH (1998). Analgesic, respiratory and heart rate effects of cannabinoid and opioid agonists in rhesus monkeys:
antagonist effects of SR 141716A^1,2^. *Journal of Pharmacology and Experimental Therapeutics*.

# Abstract: NOVEL PEPTIDES PARTICIPATE IN THE ORGANIZATION OF FEEDING BEHAVIOR

**Authors:** D. V. Boguslavsky

**Affiliations:** Institute of Higher Nervous Activity and Neurophysiology, Moscow, Russia

Novel gene named preHelixSFamid is expressed in the identified group of serotonin-containing cells of pedal ganglia of Helix lucorum, involved modulation of neural network. HelixSFamid have similarity with known peptides LymnaDFamide and Tritonia pedal peptide. Preprotein preHelixSFamid contains at the N-terminus a hydrophobic leader and ten putative amidated neuropeptides. In situ hybridization and immunocytochemical localization demonstrated that preHelixSFamid gene is selectively expressed in several identified neurons of pedal, cerebral and pleural ganglions. This expression corresponded to appearance of changes in feeding behavior. Was shown increase in number of the preHelixSFamid gene-expressing cells in hungry snails relative the number in sated animals. Also was shown increase in number of the preHelixSFamid gene-expressing neurons in juvenile snails prior to the beginning of an active feed. Ontogenetic dynamics of gene expression do not correspond to dynamics of expression of serotonin in the neural system of Helix lucorum. Apparently, peptides preHelixSFamid participate in the organization of feeding behavior of terrestrial snail. Physiological action of synthetic peptide corresponding to the predicted mimics the described action of serotonin containing interneurons.

# Abstract: EFFECTS OF LESIONS OF CATECHOLAMINE TERMINALS IN THE MEDIAL PREFRONTAL CORTEX ON DOPAMINE AND NORADRENALINE LEVELS AND ON THE REGULATION OF BODY WEIGHT

**Authors:** R. Gálosi, G. Hartmann, Z. Petykó, A. Tóth, L. Lénárd

**Affiliations:** ^1^Institute of Physiology, Pécs University Medical School, Pécs, Hungary; ^2^Neurophysiology Research Group of the Hungarian Academy of Sciences, Pécs, Hungary; ^3^Computer and Automation Research Institute of the Hungarian Academy of Sciences, Budapest, Hungary

The medial prefrontal cortex (mPFC) is innervated by dopamine (DA) and noradrenaline (NA) terminals. The aim of the present experiments was to study the effects of lesions of catecholamine (CA) terminals in the mPFC on the regulation of body weight. 6-hydroxydopamine (6-OHDA) was injected bilaterally into the mPFC to destroy CA terminals. To increase the selectivity of neurotoxin on DA or NA terminals, 6-OHDA was applied after desipramine or GBR12935 pretreatment, respectively. 6-hydroxydopa (6-OHDOPA) lesions were used to compare the effects of lesions on NA terminals. The control groups were injected with vehicle or GBR12935. Daily food and water intake and body weight were measured to the nearest g and ml, respectively. Regulatory deficits after 24 h food and water deprivation were also examined. DA and NA levels were determined in vitro from micro punched samples of the mPFC. Only transient body weight decrease was found in all groups with neurochemical lesions, but the effect of 6-OHDA with desipramine pretreatment was severe and the most pronounced. Only this group had an enhanced food intake following 24 h deprivation. 6- OHDA lesion caused 79.7% decrease in NA and 52.4% decrease in DA level compared to those of controls. After desipramine pretreatment 42.5% decrease in NA and 56.5% decrease in DA level were measured. In the groups of 6- OHDOPA and GBR12935+6-OHDA the NA levels decreased by 50% but DA levels increased by +180% and +268.8%, respectively. Our results show that DA terminals of the mPFC are involved in the regulation of body weight and that selective lesions of NA terminals in the mPFC are followed by an increase of DA level. Supported by ETT 317/2006, RET-008 MEDIPOLIS and the Hungarian Academy of Sciences.

# Abstract: LONG-TIME EFFECT OF BUSPIRONE INJECTIONS ON NEUROGENESIS AND EXPLORATORY BEHAVIOR OF THE LABORATORY OPOSSUM

**Authors:** K. Turlejski, M. Grabiec, R. Djavadian

**Affiliations:** Nencki Institute of Experimental Biology, Warsaw, Poland

Signaling through the serotoninergic receptor 5-HT1A is known to influence i.a. the rate of neurogenesis in the dentate gyrus and spatial memory. We investigated the influence of injections of the partial 5-HT1A agonist, buspirone, one month prior to the behavioral tests on the exploratory behavior and the number of neurons generated at the time of injections in the adult and aged opossums (Monodelphis domestica). Exploratory behavior was evaluated in the spatial novelty test according to Husnaker (2005). Seven six-minute sessions were performed in one day. In session 1 the exploration field was empty. In session 2–4 five objects were present there, always in the same places. In session 5-6 placement of two objects was exchanged. In session 7 one old object was replaced with a new one. All animals intensely explored new objects and then habituated. Aged opossums explored more than adults. Control opossums approached all new objects, while the buspirone-pretreated explored more but selectively. Object displacement increased exploration only in the adults injected with buspirone, while introduction of a new object increased its exploration in both adult and aged buspirone-treated groups. Injections of buspirone raised numbers of newly incorporated neurons in the dentate DG and OB. These results show that injections of buspirone may have long-time effects on both adult neurogenesis and selectivity of the exploratory behavior in the opossum.

# Abstract: DISSOCIABLE EFFECTS OF SELECTIVE 5-HT_2A_ AND 5-HT_2C_ RECEPTOR ANTAGONISTS ON SERIAL SPATIAL REVERSAL LEARNING IN RATS

**Authors:** V. Boulougouris, T. W. Robbins

**Affiliations:** Department of Experimental Psychology, University of Cambridge, Cambridge, UK

**Rationale**: Serotonin (5-HT) is strongly implicated in the ability to shift behaviour in response to changing stimulusreward contingencies. However, there is little information on the role of different 5-HT receptors in reversal learning.

**Objectives**: We investigate the effects of the selective 5-HT_2A_ and 5-HT_2C_ receptor antagonists M100907 and SB 242084 on performance of a spatial discrimination and serial reversal learning task in rats.

**Methods**: The effects of systemic administration of M100907 (0, 0.01, 0.03, 0.1 mg/kg IP) and SB 242084 (0, 0.1, 0.3, 1 mg/kg IP) were investigated on an instrumental two-lever spatial discrimination and serial reversal learning task, where both levers were presented and only one was reinforced. The rat was required to respond on the reinforced lever under a fixed ratio 3 schedule of reinforcement. Following attainment of criterion, a series of within session reversals was presented.

**Results**: Neither M100907 nor SB 242084 altered performance during spatial discrimination and retention of the previously reinforced contingencies. M100907 significantly increased both trials to criterion and incorrect responses to criterion in Reversal 1, a pattern of behaviour manifested as increased perseverative responding on the previously correct lever. In contrast, SB 242084 improved reversal learning by decreasing trials and incorrect responses to criterion in Reversal 1, with significantly fewer perseverative responses.

**Conclusions**: These data support the view that 5-HT_2A_ and 5-HT_2C_ receptors have distinct roles in cognitive flexibility and response inhibition. The improved performance in reversal learning observed following 5-HT_2C_ receptor antagonism suggests these receptors may offer the potential for therapeutic advances in a number of neuropsychiatric disorders where cognitive deficits are a feature, including obsessivecompulsive disorder (OCD).

# Abstract: EXPLORING THE EXTENT OF HIPPOCAMPAL INVOLVEMENT IN OBJECT-LOCATION RECOGNITION MEMORY IN THE RAT

**Authors:** R. F. Langston, E. R. Wood

**Affiliations:** Centre for Cognitive and Neural Systems, University of Edinburgh, Edinburgh, Scotland, UK

Rats and other animals show an innate preference to explore novel aspects of their environment (Ennaceur and Delacour, 1988). This has been exploited in tests of recognition memory for not only objects, but the locations and contexts in which they appear. Previous studies have frequently shown a role for the hippocampus in object-location memory in rats (Save, Poucet et al. 1992; Mumby et al. 2002; Good et al. 2007). However, we have recently found that memory for objects and the locations in which they are presented is not dependent on the hippocampus in one version of this task (Langston & Wood, FENS Abstracts 2006), and the same task is not affected by lesions of the fornix (Eacott & Norman, 2004). We aim to explore the differences between the paradigms used in these studies to determine which manipulations invoke the involvement of the hippocampus. Rats received complete bilateral lesions of the hippocampus with ibotenic acid or sham control operations at the start of the experiment. Lesioned rats preferentially explored objects whose locations had been swapped with other objects seen in the exposure phase over those that had not been swapped. This was dependent on the rats entering the test box at the same location on the exposure and test phases and therefore being able to use an egocentric strategy. When placed into the test box at a location that differed from that used in the exposure phase, hippocampal lesioned rats no longer preferentially explored objects in swapped locations, suggesting that an allocentric strategy may have been necessary. These data are consistent with a role for the hippocampus in allocentric, but not egocentric, spatial processing, and may provide a framework for understanding the circumstances under which the hippocampus is required for spontaneous objectlocation recognition memory.

# Abstract: MONOCULAR DEPRIVATION AND SLEEP IN THE DOMESTIC CHICK (*GALLUS GALLUS*)

**Authors:** D. Bobbo, C. Nelini, A. Quercia, G. G. Mascetti

**Affiliations:** Department of General Psychology, University of Padova, Via Venezia 8, Padova, Italy

During the development of the nervous system there are some time windows, called critical periods, in which the sensory activity induces neural modifications in the brain. Such functional and behavioral changes are aspects of a more general function known as neural plasticity. In neurons of the cats primary visual cortex, the closure of one eye (monocular deprivation) during the critical period causes a shift in eye dominance toward the nondeprived eye and a reduction of vision and visual acuity of the closed eye (Hubel e Wiesel, 1970). In domestic chicks (Gallus gallus), brain functions are remarkably lateralized (Andrew, 2002) but there are no indications on the presence of critical periods. In this species the pattern of lateralization changes during the first two weeks after hatching, shifting from an overall left hemisphere dominance during the first week to the right hemisphere one in the second week. Chicks also exhibit monocular/unihemispheric sleep (MoUn sleep), a unique behavioral and electrophysiological state in which, during sleep, one hemisphere is awakened while the other remains sleeping. We used 18 young female chicks of two days posthatching that underwent to 12 hours monocular deprivation by placing an eyepatch made of canvas. The right eye was deprived (covered) in a group of chicks (*n* = 6; RE), the left eye was covered in a second group (*n* = 6; LE) and a third group (*n* = 6; CONTROL) was maintained with both eyes uncovered. Sleep behaviour was then recorded for 6 consecutive hours, immediately after the end of monocular deprivation. Results revealed that monocular deprivation of 12 hours affects the pattern of sleep. RE chicks showed a significantly lower percentage of binocular sleep, a greater time spent in MoUn sleep and a significant higher number of episodes of left MoUn sleep compared to CONTROL chicks. No significant differences were found between CONTROL and LE chicks in all the measures considered.

# Abstract: CELLULAR AND MOLECULAR EFFECTS OF ENRICHED ENVIRONMENT ON MOUSE HIPPOCAMPUS

**Authors:** A. Trabalza, C. A. Sgobio, M. Ammassari-Teule, A. Bevilacqua

**Affiliations:** ^1^Section of Neuroscience, Department of Psychology, “Sapienza” University, Rome, Italy; ^2^Institute for Neuroscience, CNR, and Santa Lucia Foundation, Rome, Italy

Ephrins and their receptors (Eph) are among the largest families of tyrosine kinases. In the nervous system, they are involved in the patterning of axonal connections during brain development, but until now a role for these molecules in the mature brain has not been clearly described. Environmental enrichment increases adult hippocampal neurogenesis and alters hippocampal-dependent behaviors in rodents. We housed juvenile and adult C57/BL6 mice in an enriched environment condition to examine the possibility that experience-dependent stimulation induces modifications in the expression of EphrinA5 and its receptor in the hippocampus. After an eight-week training, animals were challenged by the context-dependent fear conditioning test, a behavioral paradigm sensitive to hippocampal modifications, and by the cue-dependent fear conditioning test, which relies on amygdala functions, but is not affected by hippocampal plasticity. We found that enriched mice performance was better than that of non-enriched mice in the context-dependent fear conditioning test but not in the cue-dependent fear conditioning test. Both dendritic arborization and dendritic spine density of hippocampal neurons of enriched mice were increased with respect to their control countermates, as revealed by Golgi-Cox staining. We used western blot and immunofluorescence analysis to assay the expression of Ephrin- A5 and its receptor Eph-A5 in different areas of the brain, hippocampus, cortex and striatum, of enriched and nonenriched animals. Expression of both Ephrin-A5 and its receptor in the hippocampi of enriched animals appeared to correlate with their increased neuronal plasticity. Effects of these results on spine cytoskeletal structure and on behavioral performances will be discussed.

# Abstract: AROMATASE INHIBITION IMPAIRS SPATIAL WORKING MEMORY IN MALE AND FEMALE RATS ASSOCIATED WITH CHANGES IN HIPPOCAMPAL GFAPIR ASTROCYTE POPULATION

**Authors:** N. M. Conejo, H. González-Pardo, G. Lana, J. I. Arias, J. L. Arias

**Affiliations:** ^1^Laboratory of Neuroscience, Department of Psychology, University of Oviedo, Spain; ^2^Monte Naranco's Hospital, Oviedo, Spain

Aromatase is an enzyme responsible for the transformation of the androgens like testosterone into estrogens like 17-betaestradiol in the brain, gonads and adipose tissue. Although aromatase is expressed mainly in neurons during adulthood, it can be expressed by astrocytes pre- and perinatally and also under stress conditions. Glial cells have been traditionally neglected in the study of behavior, but its crucial role in brain function is nowadays beginning to be recognized. In particular, astrocytes modulate synaptic transmission influencing neurotransmitter levels and neuronal excitability. Since astrocytes are the main cellular targets for the action of gonadal steroids, it is reasonable to hypothesize that these cells would be especially sensible to the organizational effects of sex steroids, thus influencing behavior. For this purpose, anastrozole, a selective aromatase inhibitor, was administered daily to pregnant rats (0.01 mg/kg, i.p) from day 17 of gestation until parturition. After birth, all pups were injected daily with anastrozole (0.1 mg/kg, i.p) until postnatal day 19. A different group of animals (males and females) received anastrozole only postnatally. Animals receiving vehicle during the same period were used as controls. Spatial working memory evaluated in a water maze during puberty (30 days), was similarly impaired in both males and females treated pre- and postnatally with anastrozole. Males treated postnatally with anastrozole showed a poorer performance than females in this memory task. Stereological quantification of GFAP-immunoreactive astrocyte numbers in CA1 and CA3 hippocampal subfields showed significant differences for sex and different treatments with anastrozole. Our results suggest that organizational effects of gonadal steroids on glial cells would contribute to the development of sex differences in spatial memory.

# Abstract: DIFFERENTIAL EFFECT OF CANNABINOIDS IN CORTEX ON MEMORY PHASES IN CONDITIONED TASTE AVERSION IN THE RAT CORTEX

**Authors:** T. Kobilo, S. Hazvi, Y. Dudai

**Affiliations:** Department of Neurobiology, The Weizmann Institute of Science, Rehovot, Israel

Ligands of the cannabinoid-1 receptor (CB1) have been reported to affect memory in some memory paradigms but not in others. We set out to test the role of CB1 agonist and antagonist in the rat insular cortex (IC) that subserves conditioned taste aversion (CTA) memory. CTA is a convenient model to analyze molecular and pharmacological mechanisms of aversive learning and memory. When microinfused into the IC immediately after experiencing the taste conditioned stimulus (CS) in CTA training, a CB1 agonist, but not antagonist, reduced subsequent memory. Microinfusion of the agonist immediately after the first retrieval session following single CTA training had no effect on extinction. In contrast, the antagonist blocked the extinction. When rats were trained using an intensified CTA protocol, which renders the memory highly resistant to extinction, microinfusion of the agonist after the first retrieval test led to amnesia of CTA memory. The antagonist had no effect under these conditions. Our data support a model in which endogenous occupancy of the CB1 receptor in IC modulate the hedonic valence of the CS. These data also promote further investigation of the potential use of cannabinoids in attempts to mitigate long-term aversive memories.

# Abstract: SELECTIVE DOPAMINERGIC LESIONS OF THE NUCLEUS ACCUMBENS DO NOT IMPAIR PREFERENCE FOR SEXUAL MALE PHEROMONES, BUT DELAY SUCROSE PREFERENCE AND NEOPHOBIA

**Authors:** J. Martinez-Hernandez, E. Lanuza, F. Martinez-García

**Affiliations:** Department of Functional Biology, Faculty of Biology, University of València, Spain

Male sexual pheromones are rewarding to female mice, as they are able to induce place preference, but the centralmechanisms for reward signalling of this novel natural reinforcer is ignored. In this work, we compare the effects of specific lesions of the dopaminergic innervation of the ventral striatum on two different appetitive behaviours, “pheromone seeking” and sucrose intake. Female mice with no previous experience with either adult male chemical stimuli or sucrose received injections of 6-hydroxydopamine in the accumbens (lesioned group: *n* = 8) or injections of the vehicle (sham group: *n* = 9). Then, we analysed the preference of these females for male soiled-bedding in five-minute two-choice tests (vs clean bedding), and the dynamics of the preferential intake of sucrose (vs water) during two daily 60-minute two-choice tests. For the latter tests, animals were 24-h deprived of water. Lesions did not impair locomotor activity. A repeated-measures ANOVA indicated that sham and lesioned animals showed similar preference for male sexual pheromones, a phenomenon that followed linear dynamics. In contrast, during the first sucrose-preference test, lesioned and sham animals displayed significant differences on the initial (15 minutes) dynamics of sucrose preference. Sham animals showed an initial sucrose preference followed by a preference for water, interpreted as sucrose neophobia. Lesioned animals started showing no preference, and both sucrose preference and neophobia appeared with a small but significant delay. When the 60-min data were analysed, neither the sham nor the lesioned group showed preference for sucrose (sucrose minus water), and there was no effect of the lesion on total liquid intake (sucrose plus water). During the second sucrose-preference test, both groups displayed comparable sustained preference for sucrose throughout the test.

# Abstract: PERSISTENCE OF INTERFERON-ALPHA INDUCED DEFICITS IN TEMPORAL-ORDER MEMORY IN WISTAR RATS AFTER CESSATION OF TREATMENT

**Authors:** S. Barlow, B. Fahey, D. P. Kelleher, A. M. O'Dwyer, S. M. O'Mara

**Affiliations:** ^1^Trinity College Institute of Neuroscience, Trinity College, Dublin, Ireland; ^2^St James's Hospital, Dublin, Ireland

Prefrontal cortex (PFC) has been implicated as the substrate of higher cognitive function. The validation of a behavioural task that relies purely on the integrity of the PFC has been troublesome to date. Temporal order memory is a form of working memory vital for keeping information on-line in an ordered format for example in recency discrimination. We examine the effect of interferon-alpha (IFN*α*) on this form of working memory in order to provide insights into the control of goal-directed behaviour. We used a temporalorder memory object discrimination task where rats are required to discriminate between two objects on the basis of recency [2]. Previous research has indicated that the medial PFC (mPFC) is involved in temporal-order memory, contributing to the serial organisation of behaviour. It has been documented that patients treated with IFN*α* therapy suffer deficits in working memory [1]. The utilisation of this task to assess deficits in rats treated with IFN*α* may provide a platformfor further insights into the molecular mechanisms that may be at play. Rats treated IFN*α* performed worse overall on the temporal-order memory task (*P* = 0.014). After 4 weeks of IFN*α* treatment rats performed significantly worse than saline controls displaying an inability to discriminate recency (*P* = 0.007). Three months after cessation of treatment IFN*α* animals remain deficient in this temporal order memory task (*P* = 0.031). The persistence of deficits long after the cessation of treatment is another sinister neurotoxic consequence of IFN*α* treatment. Ameliorative strategies for deficits that remain will be discussed.

## References

[1] Capuron L, Miller AH (2004). Cytokines and psychopathology: lessons from interferon-*α*. *Biological Psychiatry*.

[2] Mitchell JB, Laiacona J (1998). The medial frontal cortex and temporal memory: tests using spontaneous exploratory behaviour in the rat. *Behavioural Brain Research*.

# Abstract: ROLE OF NUCLEUS BASALIS MAGNOCELLULARIS IN RAT'S FEAR CONDITIONING CONSOLIDATION

**Authors:** Chiara Mariottini, Elisabetta Baldi, Corrado Bucherelli

**Affiliations:** Dipartimento di Scienze Fisiologiche, Università di Firenze, Italy

The nucleus basalis magnocellularis (NBM) is known to be involved in the memorization of several conditioned responses. To investigate its role in fear conditioning consolidation the neural site was subjected to fully reversible tetrodotoxin (TTX) inactivation during consolidation in adult male Wistar rats which had undergone fear training to acoustic CS and context. TTX was stereotaxically administered to different groups of rats at increasing intervals after the acquisition session. Memory was assessed as conditioned freezing duration measured during retention testing, always performed 72 and 96 hours after TTX administration. Thus there was no interference with normal NBM function during either acquisition or retrieval phases. Therefore any amnesic effect could be due only to consolidation disruption. The results show that NBM functional integrity is necessary for contextual fear response mnemonic consolidation up to the 24-hour after-acquisition delay. On the other hand, NBM functional integrity was shown to be necessary for memory consolidation of acoustic CS fear responses only immediately after acquisition but not at the 24-hour post-acquisition delay. The present findings help to elucidate the NBM role in memory consolidation and better define the neural circuits involved in fear memories.

# Abstract: PRELIMINARY CHARACTERISATION OF A SIMPLE TWO-PHASE ATTENTIONAL/WORKING MEMORY TASK IN MICE: EFFECTS OF ALPHA7 NICOTINIC RECEPTOR AGONISTS

**Authors:** J. P. Redrobe, D. Peters, G. Olsen

**Affiliations:** NeuroSearch A/S, Ballerup, Denmark

Here we present a quick and simple paradigmthat is sensitive to compounds thought to interfere with learning/attentional processing, and to those expected to enhance such functions, for example alpha7 nicotinic receptor agonists. Mice were forced to choose one of two visually distinct arms of a V-maze, and were confined to this arm for 5 min (phase 1) before being allowed to explore both arms for a 2 min test session (phase 2), immediately thereafter. The time spent in each arm, entries and total distance travelled was recorded using an automated system. Firstly, mice were treated with: scopolamine (0.25–1 mg/kg), chlordiazepoxide (10 & 20 mg/kg), caffeine (5 & 10 mg/kg) or zolpidem (1 & 3 mg/kg), 30 min before phase 1 testing. In a second set of experiments, mice were pre-treated with: ABT-582941 (0.01- 0.1 mg/kg), SSR-180711 (0.3-3 mg/kg) or PNU-282987 (1- 10 mg/kg), 15 min before scopolamine (1 mg/kg), administered 30 minprior to phase 1 testing. Control mice displayed a clear preference for the unfamiliar arm, a preference that was abolished by scopolamine (1 mg/kg). Chlordiazepoxide (20 mg/kg) also attenuated novel arm exploration, but also entries and distance travelled. Caffeine (10 mg/kg) increased the number of arm entries and total distance travelled, but did not affect preference for novel arm exploration. Zolpidem (1 & 3 mg/kg) decreased entries and distance travelled, but only attenuated novel armexploration at the higher dose. Scopolamine was chosen for use in combination studies with alpha7 nicotinic receptor agonists. Results from these experiments showed that all three agonists restored preference for novel armexploration at doses largely corresponding to their respective ex-vivo [^3^H]-bungarotoxin binding ED_50_ values. In conclusion, the test described here may represent a quick and simple measure of learning/attentional processing that avoids the need for extensive training and the use of conventional reinforcers. The present data also support the idea that alpha7 nicotinic receptors may represent an interesting target for the treatment of conditions associated with learning/ attentional dysfunction.

# Abstract: PROTEASOME-DEPENDENT PROTEIN DEGRADATION IS NECESSARY IN THE CA3 REGION OF THE HIPPOCAMPUS DURING THE CONSOLIDATION OF SPATIAL MEMORIES

**Authors:** A. T. McGauran, L. Mouledous, B. Frances, P. Roullet

**Affiliations:** ^1^Centre de Recherches sur la Cognition Animale, CNRS, Toulouse, France; ^2^Institut de Pharmacologie et de Biologie Structurale, CNRS, Toulouse, France

The synthesis of proteins has long been recognised as one of the core mechanisms required for the formation of long-term memories (LTM). In addition, some studies have reported that, along with the synthesis of new proteins, the degradation of proteins is necessary in learning and memory mechanisms in aplysia (Kandel, 2001)and also in the rat during passive-avoidance learning, in research that focussed on the CA1 region of the hippocampus (Lopez-Salon et al., 2001). In our laboratory, recent studies have reported that another region of the hippocampus, i.e., the CA3, is crucial for longterm memory formation, especially for spatial memory (Florian & Roullet, 2004)and that, during consolidation and reconsolidation, protein synthesis is necessary in this region (Artinian et al., 2007). Thus, we investigated the role of protein degradation in the CA3 region of the hippocampus during spatial memory consolidation. Animals were trained to locate a hidden platform in the Morris water maze and proteasome activity was blocked during consolidation. Bilateral intra-CA3injectionsoflactacystin (50 pmol), an inhibitor of the proteasome pathway, were infused (0.25 *μ*L) immediately post-acquisition, and retention was tested 24 hours later. Our results demonstrate that, following lactacystin treatment, animals were impaired in their ability to retain the precise location of the platform. Thus, proteasome-dependent protein degradation is necessary in this region for the consolidation of spatial memories. The results will also be discussed in relation to spatial memory reconsolidation.

# Abstract: SCOPOLAMINE-INDUCED COGNITIVE DEFICIT IN WATER MAZE IN MICE: DOSE-DEPENDENT REVERSAL OF RIVASTIGMINE

**Authors:** S. Deiana, C. Harrington, G. Riedel

**Affiliations:** School of Medical Science, University of Aberdeen, Aberdeen, Scotland (UK)

Degeneration of basal forebrain cortical cholinergic neurones occurs in Alzheimer's disease (AD) and is correlated with the degree of cognitive impairment in AD patients. Anticholinergic drugs, like scopolamine, can disrupt short-term or working memory in humans and animals and are widely used as a pharmacological model to mimic AD in rodents. Cholinesterase inhibitors (ChE-Is) improve cognitive functions in AD; several of these are approved for treatment of mild to moderate AD. Rivastigmine, a ChE-I, inhibits acetyl-cholinesterase activity selectively in cortex and hippocampus. Nevertheless, no investigations have been conducted in mice regarding the effect of different doses of rivastigmine on scopolamine-induced learning deficit in the reference memory (RM) water maze paradigm. In the present study, female NMRI mice were tested for 4 days (6 trials/day) in the RM test and received 2 probes (with no platform in the swimming pool) at 1 and 24 hours post training to assess short- and longterm memory, respectively. Mice were intra-peritoneally injected with scopolamine (0.5 mg/kg) or saline 35 minutes prior test and received rivastigmine (0.1, 0.125, 0.25, 0.5, 1 and 4 mg/kg) 5 minutes after the first injection. Reversal of the scopolamine-induced learning deficit was dosedependent, being the highest dose (0.5 mg/kg) more efficacious. Rivastigmine reversed the short-term memory impairment at the lower doses (0.1 and 0.125 mg/kg), whereas the long-term memory deficit was reversed at the higher doses (0.25 and partially at 0.5 mg/kg). Therefore, the dose range of rivastigmine effective in restoring the learning or the memory deficit does not necessarily overlap. Our study provides a more detailed account of the functional efficacy of rivastigmine in terms of different memory mechanism.

# Abstract: THE NMDA ANTAGONISTS MEMANTINE, SDZ 220,581, PCP AND MK801 PRODUCE DISSOCIABLE PATTERNS OF PERFORMANCE ON A COGNITIVE TEST BATTERY IN THE RAT

**Authors:** P. Slavinka, C. Laura, T. Mark, K. Eric, D. Sophie, L. Eli, W. M. Co Erl, R. Sunninghill, S. Windlesham

**Affiliations:** GU20 6PH, UK

There are very few treatments available for patients suffering from cognitive dysfunction related to Alzheimer's and other neurodegenerative diseases. In order for new drug entities to be developed, robust in vivo assays that assess different cognitive domains are required.

In this study, theNMDA receptor antagonists memantine (Nameda^TM^) (uncompetitive), SDZ 220, 581 (competitive), PCP (non-competitive) and MK-801 (Dizocilpine^TM^) (noncompetitive) were profiled through a within-subject operant cognitive test battery in the rat. The battery was comprised of five stages: (1) discrimination acquisition (reference memory) in which nave animals learned a simple visuo-auditory discrimination in a discrete-trial procedure; (2) delayed discrimination (working memory) in which a delay was imposed between presentation of the stimulus and the levers; (3) reversal learning (executive function) in which the leversignal contingency was reversed (4) extinction in which only one lever was now rewarded and (5) extinction consolidation (drug-free) in which the extinction test was repeated the following day.

SDZ 220, 581, MK-801 and PCP but not memantine produced a dosedependent disruption of acquisition in stage 1. SDZ 220, 581 and PCP also disrupted performance of the delay task while all four compounds disrupted the reversal test. Only MK-801 and PCP disrupted the first extinction test. In contrast, during the consolidation test all four compounds disrupted performance. SDZ 220, 581, MK-801 and memantine produced a dose-dependent increase in head entries in all phases of the test battery, while PCP increased the response only in the acquisition phase. These data show that the battery of cognitive tests used are differentially sensitive to pharmacological agents and that different NMDA receptor antagonists produce distinct and dissociable effects on cognitive processes.

# Abstract: EFFECT OF ACYLATED GHRELIN ON LEARNING AND MEMORY PROCESSES IN THE AMYGDALA

**Authors:** K. Tóth, K. László, E. Lukács, L. Lénárd

**Affiliations:** Institute of Physiology and Neurophysiology Research Group of the HAS, Pécs University Medical School, Pécs, Hungary

Acylated ghrelin(aGHR) known as a brain-gut peptide affects several physiological processes. I.c.v. or intracerebral injection of aGHR enhances memory and learning. Amygdaloid body (AMY) plays an important role in the central mechanisms of the memory and learning processes. Projections of ghrelinergic neurons were identified in the AMY, and i.c.v. applied aGHR caused c-Fos overexpression in the AMY. The aim of the present experiments was to examine potential effect of intraamygdaloid aGHR on learning. Male wistar rats were microinjected bilaterally with 50 or 100 ng aGhr, 15 ng GHR receptor antagonist D-Lys3- GHRP-6 (ANT), ANT + 50 ng aGhr (dissolved in 0.15 M sterile NaCl /0.4 microlitre) or vehicle into the AMY. In two-compartment passive avoidance paradigm animals were shocked with 0.4 mA and subsequently were infused with the solutions. Latency to enter the dark box was measured. Fifthy ng aGhr significantly increased the latency time, the 100 ng and the ANT alone were ineffective. The effect of 50 ng aGHR was eliminated by ANT pretreatment. In separate experiment effects of 50 ng aGHR, ANT alone and ANT + 50 ng aGHR were investigated in Morris water maze paradigm after their bilateral intraamygdalar applications. Injection of 50 ng aGHR significantly reduced latency to find the safe platform located in one of the quadrants of the maze. Effect of 50 ng aGHR was inhibited by ANT pretreatment. ANT alone had no effect. Our results show that the place learning and the aversive situation linked memory processes are facilitated by aGHR in the rat AMY. This effect is specific and mediated by GHR receptors, supported by the resultes of ANT treatment, but other receptor's role can not be excluded.

# Abstract: DRUG—INDUCED DEFICITS IN ACTIVE PLACE AVOIDANCE TASK: EFFECTS ON COGNITION AND LOCOMOTOR ACTIVITY

**Authors:** A. Stuchlik, D. Cinkova, L. Rezacova, L. Rehakova, V. Helesic, V. Valesova, J. Bures, K. Vales

**Affiliations:** ^1^Department of Neurophysiology of Memory, Institute of Physiology, Academy of Sciences, Prague, Czech Republic; ^2^Prague Psychiatric Center, Prague, Czech Republic

Orientation of animals in their space has long been investigated as a model of human higher cognitive functions. Animal navigation is thought to involve representations of environments, so called “cognitive maps”. Some time ago, a novel spatial cognition task named active allothetic place avoidance (AAPA) was designed, which requires allothetic mapping and cognitive coordination. We studied effect of several receptor ligands on the locomotor and spatial behavior in the AAPA. D1-like receptor antagonist SCH23390, D2-like antagonist sulpiride, muscarinic antagonist scopolamine, and NMDA receptor antagonist MK-801 were injected 20 min prior to testing in the AAPA. All substances disrupted AAPA learning; in most instances, hyper- or hypolocomotion elicited by drugs contributed to the overall behavioral impairment. For future exploitation of the AAPA in testing cognitive abilities of animals, we need to develop control avoidance conditions similarly to hidden vs visible platforms versions of the Morris water maze. This condition would allow dissociating cognitive impairments from the sensory, motor and motivational deficits.

# Abstract: ROLE OF NMDA-RECEPTORS IN BASELINE AND TASK-RELATED FIRING OF ORBITOFRONTAL CORTEX NEURONS DURING A 2-ODOUR DISCRIMINATION TASK

**Authors:** E. J. M. van Wingerden, I. Rebelo Ferreira, R. N. J. M. A. Joosten, C. M. A. Pennartz

**Affiliations:** Animal Physiology & Cognitive Neuroscience, SILS Centre for Neuroscience (CNS), University of Amsterdam (UvA), The Netherlands

The orbitofrontal cortex (OFC) is thought to guide behaviour on the basis of the cues predicting reinforcers. There is evidence to suggest that firing patterns of OFC neurons reflect the value of a stimulus predicting reward. However, the transmitter mechanisms underlying the formation of these predictive firing patterns remain unknown. One candidate is the glutamatergic neurotransmission system. We recorded activity from OFC neurons in 2 rats in a familiar environment using an electrode array holding 14 independently moveable tetrodes. Additionally, we modified the array to hold amicrodialysis probe.Used in reversed operation mode, we unilaterally perfused the OFC during recordings with solutions of N-methyl-D-aspartic acid (NMDA), D-2-Amino- 5-phosphonovalerate (D-APV), lidocaine or aCSF control. A total of 67 cells was recorded in 4 sessions. Of these, 58 cells exhibited stable firing patterns during the course of the session and thus could be considered for analysis. To assess the effect of the drugs on single unit firing in a resting state, we calculated mean firing rates over 30 second bins during perfusion intervals for each cell. Compared to aCSF perfusions, the perfusion of 0.5 mMNMDA induced a cessation of firing in 96% of neurons. Perfusion of 2% lidocaine blocked firing activity in 83% of cells. Finally, perfusion of 0.5 mM D-APV did not markedly modulate firing rates in any of the recorded cells but could block the mentioned NMDA effect. These findings confirm that this array can be used for time-specific local application of drugs during extracellular recordings in awake rats. Currently, the effect of D-APV perfusion on single unit and population activity during a 2- odour discrimination task is assessed. Pilot results confirm that OFC units exhibit differential firing patterns to cues predicting the appetitive and aversive outcome and show reward expectancy correlates. Results from drug perfusion in 2 rats performing this task will be presented.

# Abstract: AN AUTOMATED PAIRED-ASSOCIATE LEARNING (PAL) TASK THAT USES TOUCH-SENSITIVE MONITORS AND IS SENSITIVE TO MANIPULATIONS OF THE HIPPOCAMPUS

**Authors:** J. C. Talpos, B. D. Winters, R. Dias, L. M. Saksida, T. J. Bussey

**Affiliations:** ^1^Department of Experimental Psychology, University of Cambridge, Cambridge, UK; ^2^Behavioural and Clinical Neuroscience Institute, University of Cambridge, Cambridge, UK; ^3^Merck, Sharp, & Dohme, Harlow, UK; ^4^Current address Eli Lilly, Windlesham, UK

At present, Alzheimer's disease (AD) can only be confirmed post-mortem, slowing progress in developing effective treatments. A recent series of studies has shown that a test of object-in-place paired-associate learning (PAL), as part of the Cambridge neurological test battery (CaNTaB), is very effective at predicting whether patients suffering from mild cognitive disorder (MCI) will go on to develop AD. Previous research has shown that operant boxes equipped with touchsensitive computer monitors can be used to study both visual and spatial discriminations in the rat. Using this technology, two different PAL tasks were developed: (1) both the correct and incorrect stimuli are identical objects but located in different locations (SPAL); and (2) where the correct and incorrect stimuli are different objects and located in different locations (DPAL). Rats were trained in each task, and then underwent bilateral cannulation of the dorsal hippocampus. After recovery, the Na+ channel blocker lidocaine, the cholinergic antagonists scopolamine and mecamylamine, and the glutamatergic antagonists CNQX and MK- 801 were administered prior to testing. Impairments were seen in DPAL, but not SPAL, after the administration of lidocaine and the glutamatergic antagonists, but not the cholinergic antagonists. The results presented provide early indication that DPAL has utility as an automated assay for AD research, although additional research will be necessary to confirm this conclusion.

# Abstract: NMDA-RECEPTOR MODULATION BY D-CYCLOSERINE PROMOTES EPISODIC-LIKE MEMORY IN MICE

**Authors:** A. Zlomuzica

**Affiliations:** Institute of Physiological Psychology, Center for Biological and Medical Research, Heinrich-Heine-University of Düsseldorf, Duesseldorf, Germany

NMDA-R (N-methyl-D-Aspartate receptors) have been implicated in synaptic plasticity underlying one-trial learning of event-place associations. In rodents, episodic-like memory (ELM) of personally experienced events can be inferred from behavior that reflects the remembrance of the content (what kind of object was presented), place (where was this object placed) and temporal context (when was the object presented). We have previously shown that that dcycloserine (DCS), an NMDA-R agonist, ameliorates stressinduced deficits in ELM. Here, we used an experimental protocol designed to detect promnestic drug effects and investigated whether DCS, which is known to enhance learning and memory, can induce ELM under conditions where mice normally do not show ELM. Mice which have been treated i.p. with DCS (20-mg/kg) both remembered the temporal order in which two different objects have been encountered during two consecutive sample trials, as well as their spatial position during the sample trials. Most importantly, the test trial performance of these mice is compatible with ELM in terms of an integrated memory for unique experiences comprising “what”, “where” and “when” information. In contrast, mice which have received either a saline-injection or lower doses of DCS (0.2- and 2-mg/kg) did not showed such an integrated ELM. To our knowledge this is the first report showing that DCS can promote ELM in mice.

# Abstract: Theme G: Development MATERNAL SWIMMING DURING PREGNANCY ENHANCE THE ACQUISITION AND RETENTION PHASES OF LEARNING AND MEMORY IN RAT OFFSPRING

**Authors:** M. M. Akhavan, M. Emami-Abarghoie, A. A. Vafaei, B. Sadighi-Moghaddam, K. Abrari, A. Rashidy-Pour

**Affiliations:** ^1^Department of Pharmacology, School of medicine, Semnan university of medical sciences, Semnan, Iran; ^2^Department of Physiology, School of medicine, Semnan university of medical sciences, Semnan, Iran; ^3^Department of Immunology, School of medicine, Semnan university of medical sciences, Semnan, Iran

In this study the effect ofmaternal exercise as swimming during pregnancy on learning and memory in offspring has been evaluated. Female virgin Wistar rats were allowed to mate with the male rats during a 24 hours period. The pregnant rats were randomly assigned into two groups: the sedentary control group or the forced swimming group. The swimming pool was filled with water at 32 degree centigrade and the pregnant rats in the swimming group were forced to swim for 10 min once a day until delivery. Morris water maze and fear conditioning tasks were used to study the learning and memory in 37days old rat pups from swimming and sedentary mothers. A stringent two-trial-per-day, 5-day MWM training protocol and a probe trial two days after the last MWM training day revealed that performing of swimming by pregnant rats during their pregnancy significantly increased both acquisition and retention phases of their pups learning and memory. On the other hand rat pups were trained in a fear conditioning system by receiving three foot shocks with 60 sec intervals and a reminder foot shock 8 days later. The rat pups were returned to the context after the training and reminder shocks twice with 48 hours intervals and seconds of freezing were scored for each rat pup. The rat pups from swimming mothers showed significantly higher level of freezing compared to the control group which reveals enhanced long term memory in the rat pups from exercising mothers. In addition we have found that the rat pups whose mothers were submitted to forced swimming during pregnancy have significantly higher brain, liver, heart, and kidney weights compared to their sedentary counterparts. The present results provide the evidence that maternal exercise during pregnancy may enhance the brain cognitive function in offspring.

# Abstract: MONOCULAR AND BINOCULAR SLEEP IN THE DOMESTIC CHICK (GALLUS GALLUS) AFTER 8 HOURS OF SLEEP DEPRIVATION

**Authors:** C. Nelini, D. Bobbo, G. Mascetti

**Affiliations:** Department of General Psychology, University of Padova, Padova, Italy

Brain functions are strongly lateralized in domestic chicks (Gallus gallus). An important aspect of cerebral lateralization in chicks is the phenomenon of monocular-unihemispheric sleep (Mo-Un sleep). During normal sleep (binocular: both eyes closed), chicks have brief periods in which one eye is open while the other remains shut. The hemisphere contralateral to the open eye shows an EEG pattern typical of wakefulness whilst the hemisphere contra-lateral to the closed eye shows an EEG of slow wave sleep. Sleep deprivation seems to lead to a high need for sleep. We investigated the qualitative (Mo-Un sleep or binocular sleep) and the quantitative (duration of binocular and Mo-Un sleep) effects of 8 hours of sleep deprivation on the pattern of chicks' sleep. Eleven-days old chicks were divided into two groups: nondeprived (N-DEP) and deprived 8 hours (DEP-8H). Deprivation of sleep was obtained by enforced locomotion on a treadmill. After deprivation, behavioural sleep patterns were recorded for 6 hours consecutively. The number and duration of episodes of binocular and Mo-Un sleep were recorded. During the recovery period, DEP-8H chicks showed an increased time spent sleeping and had significantly longer episodes of binocular sleep than N-DEP. Furthermore, NDEP and DEP-8H chicks showed a reverse pattern of Mo-Un sleep: the former group showed a bias toward left Mo-Un sleep which is in agreement with a previous study whilst the latter group showed a bias toward right Mo-Un sleep. These results suggest that, as reported in mammals, moderate sleep-deprivation caused a rebound effect in domestic chicks which seems to be aimed at sleep recovery but also affected the pattern of Mo-Un sleep. The reversed pattern shown by deprived chicks may indicate that there was an extension of sleep recovery in the left hemisphere because it would be more active during deprivation [1].

## References

[1] Bobbo D

# Abstract: NATURAL GEOMETRY: EXPERIENCE WITH ANGULAR GEOMETRIC CUES DOES NOT AFFECT SPATIAL REORIENTATION BASED ON THE SHAPE OF THE ENVIRONMENT IN THE DOMESTIC CHICK

**Authors:** C. Chiandetti, G. Vallortigara

**Affiliations:** Department of Psychology and B.R.A.I.N. Centre for Neuroscience, University of Trieste, Italy

Several species have shown to be able of reorienting in a familiar environment using geometrical information (i.e., metric differences among walls and sense for left-right discrimination). Little is known on the role played by experience on the ability to use geometric information. Here we took advantage of the behaviour of a highly precocial species, the domestic chick (Gallus gallus) to investigate this issue. Soon after hatching chicks were reared in rectangular-shaped or circularshaped home-cages. When trained to reorient for food reinforcement in a rectangular-shaped enclosure, rectangularreared and circular-reared chicks were identically able to learn the task. In a second experiment chicks were trained in a rectangular enclosure with different panels located in correspondence to each corner, thus providing both geometrical (the shape of the enclosure) and non-geometrical (the panels) information. Rectangular-reared and circularreared chicks were identically able to learn the task. Moreover, when tested after removal of the panels, circular- and rectangular-reared chicks were equally capable of using the residual geometric information available to disambiguate the task. In a third experiment, after training with the overall arrangement of panels, chicks were tested after a dislocation of the panels (affine transformation) so as the previously reinforced panel was located in a novel, geometrically incorrect, position. Both rectangular- and circular-reared chicks resorted more to landmark use when tested in a large enclosure, whereas they preferred to use geometry when tested in a small enclosure (see also Chiandetti et al. Anim Cogn 2007); however, no differences were observed between the two groups. Overall, these results suggest that effective use of geometric information for spatial reorientation does not require experience in environments with right angles and metrically-distinct surfaces.

# Abstract: THE TIMING OF COGNITION: BOLD EFFECTS OF INFERIOR FRONTAL AND SUPERIOR TEMPORAL CONTRIBUTIONS TO LANGUAGE COMPREHENSION

**Authors:** J. Brauer, A. D. Friederici

**Affiliations:** Max Planck Institute for Human Cognitive and Brain Sciences, Leipzig, Germany

The perisylvian region of the human cortex is known to play a major role in language processing. Especially the inferior frontal cortex (IFC) and the superior temporal cortex (STC) have been investigated with respect to their particular involvement in language comprehension. In order to get a deeper insight in the timing of recruitment of languagerelated brain activation, we have contrasted functional imaging data of adults and 6-year-old children with a special focus on BOLD response timelines in these brain regions. Adults and children differ in latency of their BOLD activation twofold. First, children show an overall later peak of activation. Second, in adults IFC activation reveals peak latencies similar to STC activation, whereas children's IFC activation peaks much later compared to their STC recruitment. Within the STC, however, activations in both groups show a similar regionally bimodal pattern, with fastest peaks in voxels at the STC's mid-portion around Heschl's gyrus and longer latencies in anterior and posterior directions suggesting similar information flow in adults and children in the temporal region. The observed latency differences in BOLD responses between children and adults in inferior frontal and superior temporal areas during language comprehension are in line with the assumption of a maturational dissociation between frontal and temporal brain regions. They also support the view of developmental changes from higher processing costs in the young brain to faster and more automatic language processing in the mature brain.

# Abstract: NEWBORNS' SENSITIVITY TO BIOLOGICAL MOTION

**Authors:** L. Bardi, E. Di Giorgio, H. Bulf, F. Simion

**Affiliations:** Dipartimento di Psicologia dello Sviluppo e della Socializzazione, University of Padova, Padova, Italy

Many vertebrates species have primitive neural pathways that ensure a bias to attend toward or preferentially process sensory information about member of their specie. Newly hatched chicks attend to pattern that correspond the head region of their caregivers. Similarly, newborns humans preferentially orient toward face. Moreover, recent evidence shows that visually inexperienced chicks possess an inborn predisposition to attend to the pattern of semi-rigid motion shared by all Vertebrates. No conclusive data were up to now available for our species concerning the ontogenetic origin of the well known facilitation observed when biological motion displays are processed by the visual system. In fact, no discrimination of biological motion point-light displays was reported for infants below the age of 3 months leaving unsolved the question of whether sensitivity to biological motion is an innate capacity of the human system or is acquired through experience. Three Experiments were carried out to test whether newborns are able to discriminate biological from non-biological displays, and to exhibit a spontaneous preference for the biological motion display. In addition, we wondered whether the inversion effect is already present at birth. A sample of 44 newborn babies aged 44 ± 4.7 (Mean and SE) hours was tested. Results showed that newborns discriminate (recognition following habituation) biological vs. non biological point-light animations (Exp. 1) and, when first exposed to the displays (spontaneous preference), selectively prefer to look at the biological motion (Exp. 2). The biological motion inversion effect seems to be also present at birth, in fact, babies prefer an upright to an upside-down biological motion animation (Exp. 3). This data are consistent with those obtained in other species (i.e., naïve chicks) and provide the very first evidence that an inborn mechanism for the detection and analysis of biological motion is available in humans.

# Abstract: FORMS OF APHASIA IN CHILDREN WITH LANDAU KLEFFNER SYNDROME

**Authors:** J. Vuksanovic, M. Vukovic

**Affiliations:** ^1^Health Center, Zemun, Belgrade, Serbia; ^2^Faculty of Special Education and Rehabilitation of the University of Belgrade, Serbia

**Aims**: Landau Kleffner syndrome, although rare epileptic encephalopathy, attract great attention of epileptologists and neuropsychologists because of its dramatic presentation, difficult treatment and ambiguous outcome. One of the most prominent characteristics of this syndrome is aphasia. The purpose of this study was to describe characteristics of forms of communication disturbances most frequently seen in those children.

**Method**: Nine children with Landau Kleffner syndrome age 4 and 5 years were assessed. Following tests were used: Reynell Developmental Language Scalesrevised, The Benton Controlled Oral Word Association (COWA) Test, The Boston Naming Test, The Token Test, The Digit Span subtest of the WAIS-R, and The Auditory-Verbal Learning Test.

**Results**: In three children neither expressive nor receptive language was preserved. They expressed global aphasia with no nonverbal communication acts except eye contact. Other six children have characteristics of nonfluent aphasia. Namely, in three children receptive language in a form of concrete situation understanding is preserved, while expressive language consists of a few most frequent words. In two children expressive language is almost completely destroyed, while receptive language is preserved. One child has preserved receptive and expressive language. However, he shows disturbances at syntactic level both in expressive language.

**Conclusion**: The children suffering from Landau Kleffner syndrome display great variations in the degree of nonfluent aphasia.

**Key words**: Landau Kleffner syndrome, language, aphasia

# Abstract: LATERALIZATION OF LEXICAL AND MUSICAL TONE PERCEPTION AND MEMORY DURING DEVELOPMENT

**Authors:** E. Castro-Sierra, C. Arteaga, M. Gomez-Gama, A. Poblano, A. Portugal-Rivera, M. Zenker

**Affiliations:** ^1^Laboratory of Psychoacoustics & Auditory Physiology, HIMFG; Mexico, DF, Mexico; ^2^Laboratory of Cognitive Neurophysiology, INR; Mexico, DF, Mexico; ^3^National School of Music, UNAM; Mexico, DF, Mexico; ^4^Laboratory of Cognitive Neurophysiology, INR; Mexico, DF, Mexico; ^5^Laboratory of Psychoacoustics & Auditory Physiology, HIMFG; Mexico, DF, Mexico; ^6^National School of Music, UNAM; Mexico, DF, Mexico

Introduction and methodology. Children from 7 to 14 years of age undergo normal developmental changes from bilateral perception to right-hemisphere global perception and lefthemisphere local perception of visual images [1].

- Hypothesis I. Among native speakers of Zapotec and Otomi, 2 tone languages of Mexico, grade-school children (G. Zapotec: 6M, 6F, age range: 9 : 0–11 : 3; Otomi: 4M, 5F, age range: 9 : 7–11 : 3) manifest bilateral activation of specific brain areas (EEG: theta, alpha & beta waves) whereas high-school children (H. Zapotec: 4M, 5F, age range: 11 : 11–16 : 8; Otomi: 5M, 4F, age range: 14 : 2–17 : 6) show unilateral activation while analyzing contrasts and intersyllabic movement of lexical tones of bisyllabic words in their own language.
- Hypothesis II. There is a similar developmental lateralization of brain area activities (EEG: theta, alpha & beta waves) while these subjects remember in pairs of 5-note musical tunes which note changed in a second presentation of the tune of each pair.

**Results and conclusions.**

- Perception of lexical tones. Zapotec: (G) bilateral frontal (theta) (negative) → (H) left/right temporal (beta) (positive): fine linguistic analysis. Otomi: (G) bilateral occipito-parietal (alpha & beta) (negative) → (H) left temporal (beta) (positive): fine linguistic analysis.
- Memory of musical tones. Zapotec: (G) bilateral frontal (alpha) and temporal (alpha) (negative) → (H) right frontal superior (alpha) (negative): music memory. Otomi: (G) bilateral frontal (alpha & beta) and temporal (alpha & beta) (negative) → (H) left frontal superior (theta) (negative): music memory.

Brain activation during auditory linguistic analysis of lexical tones and auditory memory of musical tones changes from bilateral to unilateral hemisphere localization during development in 9-to 17:6-year-old Zapotec-and Otomispeakers.

## References

[1] Moses P, Roe K, Buxton RB, Wong EC, Frank LR, Stiles J (2002). Functional MRI of global and local processing in children. *NeuroImage*.

# Abstract: CORTICAL AUDITORY EVENT-RELATED POTENTIALS IN PRETERM NEWBORNS INFANTS: MATURATIONAL AND CLINICAL FACTORS

**Authors:** G. Mento, A. Suppiej, P. S. Bisiacchi

**Affiliations:** ^1^Department of General Psychology, Padua University, Padua, Italy; ^2^Department of Clinical Neurophysiology, Children's Hospital, Padua University, Padua, Italy

Preterm birth is known to be related with both neurological and cognitive deficits, such as phonological and speechrelated disorders. Mismatch Negativity (MMN) is a preattentive change-specific component of Event-Related Potentials (ERPs) occurring in response to infrequent changes in the physical properties of homogeneous series of sounds. It has been reported to underlies sensory memory and phonological competence in newborns and, likewise, to predict cognitive impairment, such as the aforementioned ones. However, it has been claimed to vary in amplitude with maturational and clinical factors like gestational age, but this relationship is still not well understood. The present study aims to confirm the presence of this discriminative response in preterm newborns and to investigate its relationship with maturational and clinic factors. ERPs were recorded in “early” (23–28 gestational weeks, *N* = 24) and “late” (29–34 gestational weeks, *N* = 33) newborns, all tested 35 weeks after conceptional age. An auditory oddball paradigm was used with frequently occurring “standard” tones of 1000 Hz and rarely occurring “deviant” tones of 2000 Hz. Both standardrelated and deviant-related waveforms, recorded at the midfrontal location, were analysed. MMN response was present in all preterm newborns and a Pearsons's correlation showed its relation with maturational indices like Gestational Age, Cranial Circumference, Lengths and Weight at birth. Moreover, a step wise linear regression yielded Gestational Age, Apgar Index and Intraventricular Haemorrhage as specific factors explaining MMN amplitudes. Considering the role of newborns' MMN response in predicting cognitive impairments, as previous studies have demonstrated, current findings suggest to take specific maturational and clinical factors into account in view of using ERPs as a new tool for prognosis of cerebral dysfunction at a very early stage.

# Abstract: DIFFERENTIATION AND MATURATION OF CORTICAL NEURONS IN THE ARCHI- AND NEOCORTEX OF PREMATURELY BORN AND FULL-TERM INFANTS

**Authors:** H. Ábrahám, B. Veszprémi, É. Gömöri, K. Kovács, A. Kravják, L. Seress

**Affiliations:** ^1^Central Electron Microscopic Laboratory, University of Pécs, Pécs, Hungary; ^2^Department of Obstetrics and Gynecology, University of Pécs, Pécs, Hungary; ^3^Department of Pathology, University of Pécs, Pécs, Hungary

Children born prematurely, even without perinatal neurological complications, have a high prevalence of cognitive and behavioral disturbances that were correlated with the reduced size of several brain areas including hippocampal formation and cerebellum. Previously we have shown that morphological and functional alterations of the preterm brain cannot be explained with the arrest of neuronal generation in the dentate gyrus and in the cerebellum (Ábrahám et al., 2004). In the present study we examined the pre-and postnatal differentiation and maturation of neuronal subtypes containing calretinin, calbindin and parvalbumin in the temporal archi-and neocortex. Their distribution and morphology observed in preterm infants were compared to full-term agematched controls. Large calretinin-stained cells in layer I of the cerebral cortex co-expressing reelin and p73 were classified as Cajal-Retzius cells. Calretinin and calbindin both appeared early in the fetal period (18th–20th gestational weeks) in the cerebral cortex and were expressed at birth in an amount and distribution that did not differ in preterms and in full-term controls. Parvalbumin-positive cells were detectable only at term, and their appearance and maturation were also similar in preterms and in full-term infants. However, the number of Cajal-Retzius cells that play an important role in the migration of neurons during development and almost completely disappear from the cortex postnatally, was significantly higher in the preterms than in their fullterm controls. Our results suggest that similarly to neuronal proliferation (Ábrahám et al., 2004), expression of calciumbinding proteins seem to be genetically controlled events and are not influenced by preterm delivery. In contrast, the postnatal disappearance of Cajal-Retzius cells might be regulated by othermechanism that is disturbed following preterm birth.

# Abstract: COMMUNAL NESTING, AN EARLY SOCIAL ENRICHMENT, AFFECTS SOCIAL COMPETENCES AND BRAIN FUNCTION AT ADULTHOOD

**Authors:** I. D'Andrea, E. Alleva, I. Branchi

**Affiliations:** Section of Behavioural Neurosciences, Dipartimento di à Biologia cellulare e Neuroscienze, Istituto Superiore di Sanità, Rome, Italy

In order to study the effects of the early experiences on adult brain function and behaviour, we exposed mouse pups to an early social enrichment: Communal Nest (CN). CN, which consists in a single nest where three mothers keep their pups together and share care-giving behaviour from birth to weaning, mimics the natural ecological niche of the mouse species. At adulthood, mice reared in CN display higher propensity to interact socially, more elaborate social skills and higher NGF and BDNF levels in selected brain areas, when compared to mice reared in standard laboratory conditions. Homecage observations, carried out 24/24h for two consecutive days revealed that, when the hierarchy is established (first day), CN mice display higher levels of social investigation behavior. However, when exposed to cage cleaning (second day), a stimulus challenging social hierarchy, CN mice display higher levels of offensive behavior. The home-cage findings show that CN mice display a wider repertoire of social behaviors and confirm that they have a stronger propensity to interact with same-sex conspecifics. Furthermore, though CN mice aremore aggressive, they have elaborate social competences displaying high levels of aggressive behavior only when needed to set up their own territory. Overall, these findings confirm the crucial role played by early social experiences in shaping adult behavior and suggest a role for neurotrophins as factors mediating the long-term effects of experiences on brain function. Support contributed by EU, project INTELLIMAZE contract n 037965 and by project NIH-ISS Rif. 0F14, both to Enrico Alleva.

# Abstract: BEHAVIORAL ALTERATIONS IN YOUNG ADULT RATS FED A HIGH FAT DIET

**Authors:** G. Boukouvalas, E. Papalexi, G. Soulis, C. Kittas, E. Kitraki

**Affiliations:** Lab of Histology & Embryology, Athens University Medical School, Athens, Greece

Prolonged high fat (HF) feeding has been shown to alter the hypothalamic pituitary adrenal axis of adult male rats. In this study we have examined the behavioral impact of HF feeding on adult (postnatal day 80) rats of both sexes, exposed to the diet during development. The rats were fed the HF diet from weaning to adulthood (8-week protocol) or from puberty to adulthood (4-week protocol). A 1-week protocol, when in adulthood, was also included in the study. The 8-week diet protocol enhanced the exploratory behavior of male rats in the open field, by means of moving, rearing and center crossings, and the vertical locomotion of female rats. One week of HF feeding in adulthood increased sniffing in males, but decreased locomotion in female rats. Notably, the HF protocol applied from puberty to adulthood did not affect the open field behavior in either sex. When forced to swim in a glass cylinder,HF animals of both sexes, in either the 8-week or the 1-week protocol, adopted a less active behavioral response, by reducing swimming and increasing immobility time. Interestingly, when HF diet was applied from puberty to adulthood (4-week protocol), it induced the same as above behavioral response to swimstress inmales, but had no effect on females. These data show that fat diet feeding can differentially affect the behavior of male and female rats. This was particularly evident during adolescence. In the paradigms used, young females appear more resistant to fat diet-induced alterations in behavioral coping with novelty or acute stress.

# Abstract: THE EFFECTS OF NEGLECT ON COGNITIVE FUNCTIONS: A NEUROPSYCHOLOGICAL PERSPECTIVE

**Authors:** P. Nolin

**Affiliations:** Department of Psychology, University of Quebec at Trois-Rivieres

The aim of this study is twofold: First, to investigate whether cognitive functions can contribute to differentiating neglected children with or without physical abuse compared to comparison participants; second, to demonstrate the detrimental impact of children being victimized by a combination of different types of maltreatment. 129 children aged 6 to 12 years and currently receiving Child Protection Services because of one of two types of maltreatment (neglect with physical abuse, *n* = 84; neglect without physical abuse, *n* = 45) were compared with a control group of 89 children matched for age, gender and annual family income. The neuropsychological assessment focused on attention, and frontal/executive functions. Discriminant analysis identified auditory attention (Function 1), and frontal/executive functions (Function 2) as the two sets of variables that most distinguished the groups. Discriminant analysis predicted group membership in 61% of the cases. Children who were neglected with physical abuse showed cognitive deficits in Function 1 and Function 2. Children who were neglected without physical abuse differed from the control group in Function 1. Surprisingly, these same children demonstrated a greater capacity in Function 2 than the physically abused neglected and control children. Those results reproduce those of Nolin & Ethier (2007). The present study underscores the relevance of neuropsychology to maltreatment research. The results support the heterogeneity of cognitive deficits in children based on different types of maltreatment and the fact that neglect with physical abuse ismore harmful than neglect alone.

# Abstract: INTEGRATION OF NEURAL PROGENITORS INTO THE CIRCUITS OF THE NEONATAL BRAIN

**Authors:** P. Muzzi, W. Kilb, S. Kodirov, M. Bossi, M. Mirabelli, A. Gilardini, M. Amedeo, G. Tredici, H. Luhmann, A. Vercelli

**Affiliations:** ^1^DAFML, University of Torino, Torino, Italy; ^2^Institute of Physiology, University of Mainz, Mainz, Germany; ^3^Department of Neurosciences and Biomedical Technologies, Bicocca University of Milan, Monza, Italy

Cells from the ganglionic eminence (GE) and the dorsal telencephalon (DT) of mice of different embryonic ages expressing green fluorescent protein (EGFP) were transplanted into the brain of newborn, P7 and adult mice. At different intervals from transplantation, EGFP+ cells in the host brains were morphologically and immunohistochemically characterized, or studied under Electron Microscopy. In some mice, EGFP+ projection neurons were retrogradely labeled with FluoroRuby fromthe thalamus. In addition, coronal brain slices were prepared from 3-week-old mice which had received EGFP+ cells at P1 and whole-cell voltage- and current-clamp recordings were obtained from EGFP+ neurons. EGFP+ cells could be observed in all animals which underwent a transplantation at P0-P7. Their phenotype was strictly dependent from where progenitors were dissected: GE-derived cells were suggestive of interneurons and oligodendrocytes. DT cells gave a larger variability of phenotypes, some of which suggestive of pyramidal neurons. EGFP+ neurons were integrated into the host neural circuits, since they could be retrogradely labeled from the thalamus, received synaptic boutons and made synapses with the surrounding neuropil. Moreover, EGFP+ cells displayed repetitive action potentials and excitatory synaptic inputs, suggesting they were functional neurons integrated into synaptic networks.

# Abstract: DETERMINANTS OF MATERNAL AND INFANTICIDAL BEHAVIOR IN FEMALE MOUSE F1 HYBRIDS

**Authors:** W. Shi, U. Singh, Y. Yu, F. Jens, A. Orth, K. Broad, E. Keverne, R. Fundele

**Affiliations:** University of Uppsala, Sweden

Mice from one strain of mice and the F1 offspring of different inbred mice are genetically largely identical. However, females obtained by matings between two different mouse species exhibited strikingly different maternal behavior towards alien pups, even when derived from inbred strains. Microarray hybridization on brains of infanticidal and maternal females yielded a set of differentially expressed genes that are involved in behavior, olfaction and epigenetic regulation.
